# Sesquiterpene Coumarin Ethers with Selective Cytotoxic Activities from the Roots of *Ferula huber-morathii* Peşmen (Apiaceae) and Unequivocal Determination of the Absolute Stereochemistry of Samarcandin

**DOI:** 10.3390/ph16060792

**Published:** 2023-05-26

**Authors:** Fatma Memnune Eruçar, Fadıl Kaan Kuran, Gülsüm Altıparmak Ülbegi, Süheyla Özbey, Şule Nur Karavuş, Gülşah Gamze Arcan, Seçil Yazıcı Tütüniş, Nur Tan, Pınar Aksoy Sağırlı, Mahmut Miski

**Affiliations:** 1 Department of Pharmacognosy, Faculty of Pharmacy, İstanbul University, 34116 İstanbul, Türkiye; memnune.erucar@istanbul.edu.tr (F.M.E.); kaankuran@istanbul.edu.tr (F.K.K.); secilyaz@istanbul.edu.tr (S.Y.T.); nurtan@istanbul.edu.tr (N.T.); 2 Department of Biochemistry, Faculty of Pharmacy, İstanbul University, 34116 İstanbul, Türkiye; gulsum.altiparmakulbegi@istanbul.edu.tr (G.A.Ü.); gamze.arcan@istanbul.edu.tr (G.G.A.); aksoyp@istanbul.edu.tr (P.A.S.); 3 Department of Engineering Physics, Faculty of Engineering, Hacettepe University, 06800 Ankara, Türkiye; sozbey@hacettepe.edu.tr; 4 Department of Pharmacognosy, School of Pharmacy, İstanbul Medipol University, 34810 İstanbul, Türkiye; sule.karavus@medipol.edu.tr

**Keywords:** *Ferula huber-morathii*, sesquiterpene coumarin ethers, cytotoxicity, COLO 205, MCF-7, K-562, Bcl-XL, caspase 3, 8, 9, β-catenin, molecular docking

## Abstract

Ancient physicians frequently used the resin of *Ferula* species to treat cancer. Today, some folkloric recipes used for cancer treatment also contain the resin of *Ferula* species. The dichloromethane extract of the roots of *Ferula huber-morathii* exhibited cytotoxic activities against COLO 205 (colon), K-562 (lymphoblast), and MCF-7 (breast) cancer cell lines (IC_50_ = 52 µg/mL, 72 µg/mL, and 20 µg/mL, respectively). Fifteen sesquiterpene coumarin ethers with cytotoxic activity were isolated from the dichloromethane extract of the roots of *F. huber-morathii* using bioactivity-directed isolation studies. Extensive spectroscopic analyses and chemical transformations have elucidated the structures of these sesquiterpene coumarin ethers as conferone (**1**), conferol (**2**), feselol (**3**), badrakemone (**4**), mogoltadone (**5**), farnesiferol A (**6**), farnesiferol A acetate (**7**), gummosin (**8**), ferukrin (**9**), ferukrin acetate (**10**), deacetylkellerin (**11**), kellerin (**12**), samarcandone (**13**), samarcandin (**14**), and samarcandin acetate (**15**). The absolute configuration of samarcandin (**14**) was unequivocally determined by the X-ray crystallographic analysis of the semi-synthetic (*R*)-MTPA ester of samarcandin (**24**). Conferol (**2**) and mogoltadone (**5**) were found to be the most potent cytotoxic compounds against all three cancer cell lines; furthermore, these compounds exhibit low cytotoxic activity against the non-cancerous human umbilical vein epithelial cells (HUVEC) cell line. Investigation of the biological activity mechanisms of mogoltadone (**5**) revealed that while suppressing the levels of Bcl-XL and procaspase-3 in the COLO 205 cancer cell line, it did not have a significant effect on the Bcl-XL, caspase-3, and β-catenin protein levels of the HUVEC cell line, which may explain the cytotoxic selectivity of mogoltadone (**5**) on cancer cell lines.

## 1. Introduction

Cancer is a complex set of diseases originating from the uncontrolled division of abnormal cells. Cancer is a major health problem worldwide and is the second leading cause of death in the United States. According to the most recent statistical estimations, breast, colon, and lymphoma cancers are the major types of new cases and causes of death in the United States [1]. Natural products and natural product-derived molecules represent the major portion of the anticancer drugs approved by the FDA since 1981 [2]; thus, further exploration of the natural sources from historical and/or folkloric knowledge could lead to novel anticancer drug candidates.

Pedanius Dioscorides described five *Ferula* drugs in his “De Materia Medica” and mentioned their use for the treatment of tumors about 2000 years ago [3,4]. İbn-i Sina (i.e., Avicenna) also described the use of certain *Ferula* resins in the Canon of Medicine; he suggested cutting open the tumor and then directly applying the resin for treatment [5]. *Ferula* resins are currently used in traditional medicine recipes such as Thai herbal recipe Benja Amarit [6], which was used to treat liver and colon cancers. In addition, cancer patients from certain geographical areas use *Ferula* resins as a complementary alternative medicine (CAM) [7].

*Ferula* species are rich sources of sesquiterpene esters and coumarins [8,9,10,11]. While certain sesquiterpene esters of *Ferula* species show cytotoxic activity on resistant cancer cell lines [12], sesquiterpene coumarins through the competitive inhibition of the p-glycoprotein transport mechanism reverse the p-glycoprotein-mediated multidrug-resistant cancer cell lines [13,14]. The cytotoxic activity of the sesquiterpenoids of *Ferula* species is the most prominent aspect of the genus *Ferula* L. As part of our continuing investigation of the bioactive compounds of *Ferula* species growing in Anatolia [9,15,16,17,18,19,20,21,22,23,24], *Ferula huber-morathii* Peşmen, a rare endemic species growing in the vicinity of Erzurum providence of Eastern Anatolia from the subgenus *Dorematoides* (Regel & Schmalh.) Korovin of the genus *Ferula* L. [25], has been investigated for its cytotoxic compounds.

## 2. Results and Discussion

### 2.1. Bioactivity-Directed Isolation and Structure Elucidation of Cytotoxic Sesquiterpene Coumarin Ethers

Cytotoxic activity testing of the dichloromethane and methanol extracts of the roots of *Ferula huber-morathii* has shown that the cytotoxic compounds were in the dichloromethane extract; in contrast, the methanol extract of the root did not show any cytotoxic activity at a concentration up to 100 µg/mL (Table 1).

**Figure 1:**
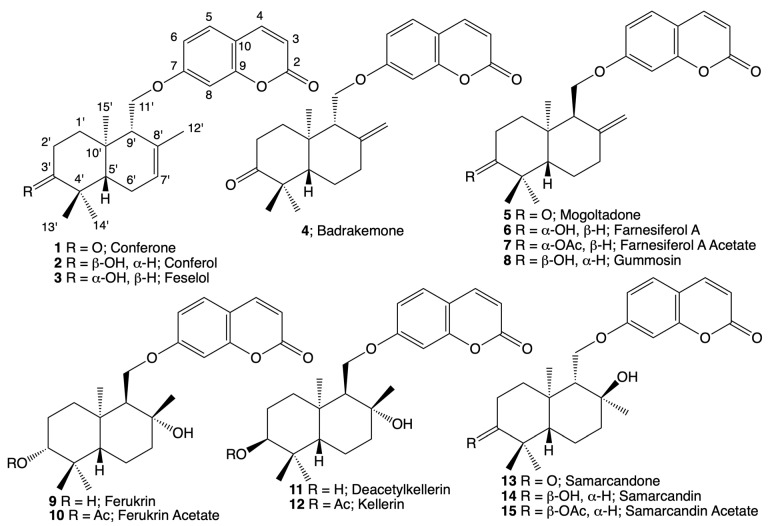
Structures of the cytotoxic sesquiterpene coumarin ethers of *Ferula huber-morathii*.

The cytotoxic dichloromethane extract of the roots of *Ferula huber-morathii* was subjected to the bioactivity-directed fractionation on a Sephadex LH-20 column (Supplementary Materials Figure S1). Initial fractions were combined based on their similar cytotoxic activities and thin layer chromatography (TLC) profiles. Then, each combined fraction group was subjected to further purification using various chromatographic techniques (i.e., RP-18 flash chromatography, prep. TLC on silicagel plates and prep. high-performance liquid chromatography (HPLC)). The following 15 sesquiterpene coumarin ethers were isolated from the aforementioned fractions: conferone (**1**) [26], conferol (**2**) [27], feselol (**3**) [27], badrakemone (**4**) [28], mogoltadone (**5**) [29], farnesiferol A (**6**) [30], farnesiferol A acetate (**7**) [31], gummosin (**8**) [32], ferukrin (**9**) [33], ferukrin acetate (**10**) [33], deacetylkellerin (**11**) [34], kellerin (**12**) [35], samarcandone (**13**) [36], samarcandin (**14**) [37], and samarcandin acetate (**15**) (Figure 1).

Due to the availability of limited NMR data for the isolated cytotoxic coumarins in the literature, extensive 2D NMR spectroscopic techniques, such as 2D ^1^H-^1^H correlation spectroscopy (COSY), nuclear Overhauser effect spectroscopy (NOESY), heteronuclear single-quantum coherence (HSQC), heteronuclear multiple-bond connectivity (HMBC), (Supplementary Materials Figures S2–S91) as well as high-resolution mass spectroscopy (HRMS) and optical rotation measurements (Supplementary Materials Table S1), were used for the confirmation of their structures. However, many of the proton signals of bi-cyclic drimane sesquiterpene nucleus appear as overlapping signals in the upfield region of NMR spectra (Table 2) and create ambiguity for the 2D NOESY stereochemical assignments of some of the sesquiterpene coumarins. Thus, most of the isolated compounds were synthesized by chemical transformations (Supplementary Materials Figure S92), and structures of the parent and daughter compounds were unambiguously confirmed by comparing their spectroscopic data with those of semi-synthetic derivatives. Detailed ^1^H- and ^13^C-NMR data of all 15 cytotoxic coumarins are listed in Table 2.

In order to illustrate the use of chemical transformation for the structure elucidation of *Ferula huber-morathii* sesquiterpene coumarins, the determination of the structure of ferukrin (**9**) will be described. The molecular formula of **9** was identified as C_24_H_32_O_5_ from the ESI-MS peak observed at *m*/*z* 423.15 ([M+Na]^+^, calc. for C_24_H_32_O_5_Na^+^), indicating nine degrees of unsaturation. The ^1^H-NMR, ^13^C-NMR, 2D COSY, HSQC, HMBC, and NOESY (Supplementary Materials Figures S50–S55) indicated that the structure of **9** is a sesquiterpene coumarin derived from umbelliferone and a bicyclic drimane-type sesquiterpene triol by forming an etheric bond between the C-7 hydroxyl group of umbelliferone and C-11′ hydroxyl group of the drimane triol. Since the ^1^H-NMR signals of 10 cyclic aliphatic protons of the drimane sesquiterpene nucleus were clustered between the δ 1.30 and 1.80 ppm region of the ^1^H-NMR spectrum of **9**, the stereochemistry data obtained from the 2D NOESY spectrum was ambiguous. However, the C-3′ hydroxyl group geminal proton (i.e., H-3′) appeared as a double of a doublet at δ 3.12 ppm (*J* = 4.4, 11.5 Hz), indicating the equatorial orientation of the C-3′ hydroxyl group of the drimane triol nucleus. Due to the presence of both equatorially (i.e., compounds **1**-**4**) and axially (i.e., compounds **5**–**8**) oriented C-11′ drimane ethers in the dichloromethane root extract of *F. huber-morathii*, identification of the spatial orientation of C-11′ methylene group of **9** was the key aspect of its structural determination. If the orientation of the C-11′ methylene group of **9** was equatorial (as observed with compounds **1**–**4**), then the structure of **9** should be isosamarcandin (**16**). To explore whether the structure of **9** was isosamarcandin or not, samarcandin (**14**) was transformed to isosamarcandin (**16**) through an oxidoreductive chemical transformation (see Supplementary Materials Figure S92). The ^1^H-NMR data of the semi-synthetic isosamarcandin (**16**) (see Supplementary Materials Figure S93) was not similar to that of **9**. Thus, the structure of **9** was confirmed as ferukrin. 

In a recent publication [38], the presence of 4 daucane aromatic esters: ferutinin (**17**), elaeochytrin A (**18**), teferidin (**19**), and feruhermonin C (**20**), and 6 sesquiterpene coumarin ethers: mogoltavidin (**21**), deacetylkellerin (**11**), kellerin, farnesiferol A (**6**), gummosin (**8**), ferukrin acetate (**10**), and a guaianetriol; teuclatriol (**22**) (Supplementary Materials Figure S94) was reported from the chloroform root extract of *Ferula huber-morathii*. The potential aphrodisiac activity of some of these compounds was tested on male rats in comparison with sildenafil citrate as a positive reference compound, and ferutinin (**17**) was determined as the most potent compound of the root extract, which was present at ca. 19.5% (*w*/*w*) in the chloroform extract of the root.

Ferutinin (**17**) is a strong cytotoxic compound [39]; however, during the bioactivity-directed isolation study, this compound was not found in the dichloromethane extract of the roots of *Ferula huber-morathii*. Nevertheless, the absence of ferutinin (**17**) and elaeochytrin A (**18**) in the dichloromethane extract of the roots of *F. huber-morathii* was further investigated by direct HPLC and TLC comparison of the ferutinin (**17**) and elaeochytrin A (**18**) reference compounds with the dichloromethane extracts of two *F. huber-morathii* root samples collected from two different locations and times (i.e., Muş to Varto, 37 km from Zorabat village in June 1983 and between Erzurum and Varto, roadside in July 2017). The extract prepared from the old root samples was designated as the old extract, and the extract prepared from the root samples collected in 2017 was designated as the new extract; neither extract showed the presence of ferutinin (**17**) and/or elaeochytrin A (**18**) in its TLC and HPLC comparison chromatograms (Supplementary Materials Figures S95 and S96). Moreover, the paper published by Baykan et al. [39] describes the ferutinin content of the root extracts of several *Ferula* species growing in Türkiye, including *F. huber-morathii*, and does not list the presence of substantial quantities of ferutinin in the root extract of *F. huber-morathii*. In addition, it should be noted that *F. huber-morathii* is a member of subgenus *Dorematoides* (Rgl. et Schmalh.) Korovin of the genus *Ferula*, and from the chemotaxonomical point of view, phytochemical studies performed on the members of this subgenus revealed the presence of sesquiterpene coumarins, sesquiterpene chromones, sesquiterpene aryl compounds, phenylpropanoids, and guaiane sesquiterpenes, but daucane aromatic esters were never found in the *Ferula* species of subgenus *Dorematoides* [13,40,41,42,43,44,45,46,47,48,49,50,51,52,53,54]. The biosynthetic pathway of daucane sesquiterpenes is different [19] than those of germacranes, elemanes, eudesmanes, and guaianes, and the aromatic esters of daucane sesquiterpenes are commonly found in the *Ferula* species of subgenus *Euferula* (Boiss.) Korovin [9,16,18,19,20] and subgenus *Peucedanoides* (Boiss.) Korovin [12,15,23]. Thus, the presence of three daucane aromatic esters, ferutinin (**17**), elaeochytrin A (**18**), and teferidin (**19**), in *F. huber-morathii* is highly unlikely. In addition, the figure of structures of sesquiterpene coumarins reported in the same publication [38] displays the structure of mogoltavidin (**21**) as the enantiomer of deacetylkellerin (**11**) (Supplementary Materials Figure S94) that is not chromatographically separable by the analytical procedures described in the publication, and based on the basic organic chemistry rules, the spectroscopic data of enantiomeric compounds should be identical except for their opposite optical rotation values. However, the NMR spectra data for mogoltavidin (**21**) and deacetylkellerin (**11**) were reported as completely different spectra [38]; thus, the proposed structure for mogoltavidin (**21**) is incorrect. Careful examination of the reported ^1^H- and ^13^C-NMR spectra of mogoltavidin (**21**) (i.e., SP 15 and SP 16) in the supplementary data files of the previous publication [38] and comparison with the ^1^H and ^13^C-NMR spectra of samarcandin (**14**) shown in the supplementary files of the current publication (Supplementary Materials Figures S80–S85) confirm the actual structure of mogoltavidin (**21**) as samarcandin (**14**).

### 2.2. Determination of the Absolute Configuration of Samarcandin **14**

Samarcandin (**14**), along with its keto derivative samarcandone (**13**), was isolated from *Ferula samarcandica* Korovin in 1968, but the initial structure proposed for samarcandin (**14**) was incorrect [36]. Several biological activities were attributed to samarcandin (**14**), such as cytotoxic activity against the AGS (human gastric carcinoma) and WEHI-164 (fibrosarcoma) cancer cell lines [37], activity against NCI yeast anticancer drug screen assay [55], and potential antiviral [56,57] and aphrodisiac activities [58]. The structure of samarcandin (**14**) was revised based on NMR decoupling experiments [59] and later by comparative optical rotation measurements of several sesquiterpene coumarin ethers, including various samarcandin derivatives and isomers [60]. Nevertheless, the stereochemistry of samarcandin proposed in these publications was incorrect. The relative stereochemistry of samarcandin (**14**) was established with a single crystal X-ray crystallographic analysis [61]; however, without providing any supporting evidence, the relative stereochemistry was proposed as the absolute stereochemistry of samarcandin in this paper. In a more recent publication, the stereochemistry of samarcandin (**14**) was reinvestigated by computational chemistry methods and X-ray crystallographic analysis. Despite the lack of required specific measurements and calculations for the establishment of absolute stereochemistry of samarcandin (**14**), the enantiomer of samarcandin (i.e., *ent*-samarcandin, **23**) was proposed as the absolute atomic structure of samarcandin [37]. Close examination of the reviews of sesquiterpene coumarins in the literature also revealed the presence of similar confusion about the absolute stereochemistries of other sesquiterpene coumarins [62,63,64,65].

**Figure 2:**
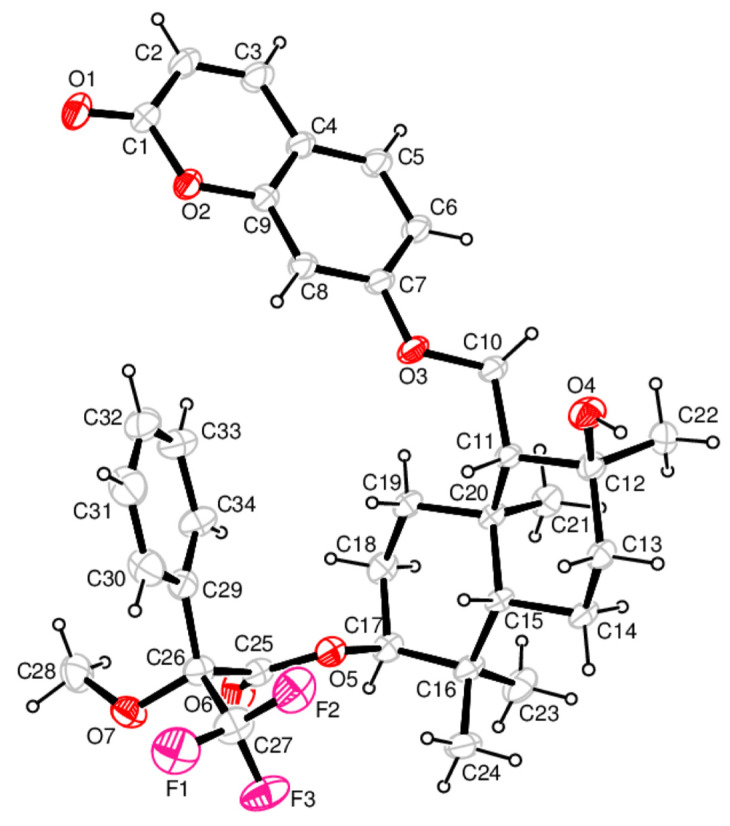
ORTEP drawing of the (*R*)-MPTA ester of samarcandin (**24**) with the atom numbering scheme. The displacement ellipsoids are drawn at a 30% probability level. The H atoms are drawn as small circles of arbitrary radii.

To identify the absolute stereochemistry of samarcandin (**14**), the (*R*)-MTPA ester of samarcandin **(24**) (Supplementary Materials Figure S99) was prepared, and its solid-state packing was investigated using X-ray crystallography. The molecular structure with the atom numbering scheme and the packing arrangement of the molecules are presented in Figure 2 and Supplementary Materials Figure S109. The asymmetric unit consists of one molecule of (*R*)-MTPA ester of samarcandin (**24**), as shown in Figure 2. As expected, the coumarin moiety is nearly planar; the displacements of all ten atoms contained in the ring are less than 0.019(2) Å [for C(4)] from the least-squares plane. Two cyclohexane rings in the sesquiterpene parts of the samarcandin adopt a chair conformation with the spherical polar set values of Q = 0.5915(15) Å, θ = 7.67(15)°, and φ =2 52.4(11)° for the C11/C12/C13/C14/C15/C20 ring; these values are Q = 0.5383(16) Å, θ = 5.11(17)°, φ = 227.0(19)° for the C15/C16/C17/C18/C19/C20 ring. The O1−C1 and O6−C25 bond lengths of 1.213(2) Å and 1.192(2) Å, respectively, match the value for double bond C=O, while the bonds O2–C1 [1.3731(18) Å], O2–C9 [1.3772(18) Å], C7–O3 [1.3552(16) Å], O3–C10 [1.4390(18) Å], O4–C12 [1.4511(17) Å] and O5–C25 [1.3317(19) Å], O5–C17 [1.478(2) Å] correspond to the value for single C–O bonds. The MTPA moiety of the molecule adopts an extended conformation with torsion angle C17−O5−C25−O6 [3.0(2)°] and O5–C25–C26–C29 [68.99(15)°]. The trifluoro and methoxy groups attached to the C26 atom are twisted out of the phenyl ring plane with torsion angles of 142.46(18)° for C34–C29–C26–C27 and −102.92(18)° for C34–C29–C26–O7.

All of the intra- and intermolecular contacts were examined with Platon. Two types of intramolecular interactions, C−H···O and C−H···F, and one intermolecular hydrogen bond, O−H···O, were detected in the structure [O(4)−H(1)···O(1)i 0.87(3)Å 2.17(3)Å 3.028(2)Å 168(3)°] [Symmetry code: (i) −x, −1/2 + y, −z].

### 2.3. Cytotoxic Activities of the Sesquiterpene Coumarin Ethers Isolated from the Dichloromethane Extract of the Roots of Ferula huber-morathii

The cytotoxic activities of individual sesquiterpene coumarin ethers isolated from the roots of *F. huber-morathii* were tested against the COLO 205, K-562, and MCF-7 cancer cell lines, as well as the non-cancerous human umbilical vein epithelial cells (HUVEC) cell line. The results are shown in Table 3, which showed that compounds **4**, **6**, **8**, **9**, **10**, **11**, **13**, **14**, and **15** exhibited little or no cytotoxicity. On the contrary, compounds **1**, **2**, **3**, **5**, and **7** showed moderate or strong cytotoxicity. Moreover, compound **12** showed a moderate cytotoxic effect only for MCF-7 cell lines.

**Figure 3:**
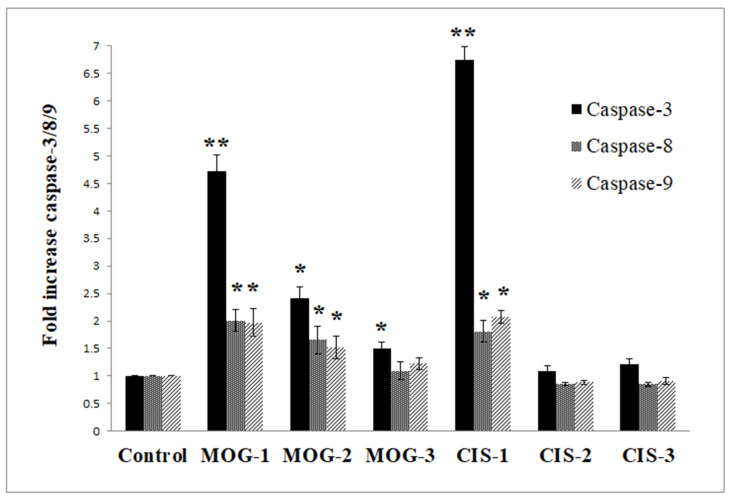
Effect of mogoltadone (MOG) and cisplatin (CIS) on the caspase-3, -8, and -9 activation in the COLO 205 cell line. * *p* < 0.05; ** *p* < 0.005. MOG-1: 131.58 µM; MOG-2: 26.32 µM; MOG-3: 2.63 µM. CIS-1: 131.58 µM; CIS-2: 26.32 µM; CIS-3: 2.63 µM.

Based on the IC_50_ values, conferol (**2**) and mogoltadone (MOG, **5**) are the most potent sesquiterpene coumarins against cancer cell lines. However, the selectivity indexes of mogoltadone (**5**) for the COLO 205, K-562, and MCF-7 cells lines (>8.29; >12.46; >8.64, respectively) were higher than those of conferol (**2**) (5.45; 1.73; 3.82, respectively). Therefore, the caspase-3, 8, and 9 activity analyses for the COLO 205, K-562, and MCF-7 cell lines were performed by mogoltadone (**5**). The data showed that the caspase-3, 8, and 9 activities were the highest in the COLO 205 cell line in comparison with the other cancer cell lines. Concerning the results for the COLO 205 cell line, mogoltadone (**5**) dose-dependently increased the caspase-3, 8, and 9 activities. Furthermore, mogoltadone (**5**) showed an increase in caspase-3 activity by 4.72-fold, in caspase-8 activity by 2-fold, and in caspase-9 activity by 1.97-fold when compared with the control at the highest concentration (131.58 µM) (Figure 3). Cisplatin (CIS) was used as a positive control for the caspase activities. Based on these results, the cisplatin indicated an increase in the caspase-3 activity by 6.73-fold, in caspase-8 activity by 1.8-fold, and in caspase-9 activity by 2.07-fold compared with the control at the highest concentration (Figure 3).

**Figure 4:**
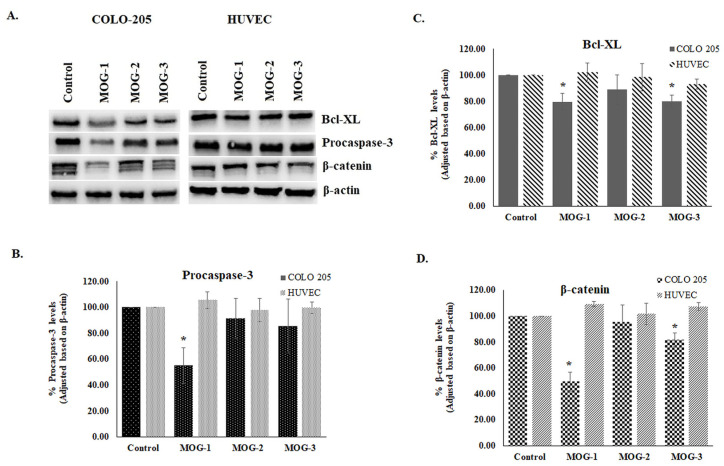
Effect of mogoltadone (MOG) on the Bcl-XL, procaspase-3, and β-catenin immunoreactive protein levels in the COLO 205 cell line. (**A**) Bcl-XL, procaspase-3, and β-catenin immunoreactive protein bands; (**B**) Bcl-XL immunoreactive protein levels in the COLO 205 cell line; (**C**) procaspase-3 immunoreactive protein levels in the COLO 205 cell line; (**D**) β-catenin immunoreactive protein levels in the COLO 205 cell line. * *p* < 0.05. MOG-1: 131.58 µM; MOG-2: 26.32 µM; MOG-3: 2.63 µM.

Western Blot analysis indicated that mogoltadone (**5**) suppressed the level of Bcl-XL, an antiapoptotic protein, and decreased the procaspase-3 level in the COLO 205 cell line. The statistically significant effect for procaspase-3 was observed at a concentration of 131.58 µM. These data supported the results of the caspase-3 activity. Furthermore, the effect of mogoltadone (**5**) on the β-catenin levels was also investigated and the results showed that mogoltadone decreased the β-catenin levels in the COLO 205 cell line. In contrast, mogoltadone (**5**) did not have a statistically significant effect on the Bcl-XL, caspase-3, and β-catenin protein levels of the HUVEC cell line, which may explain the cytotoxic specificity of mogoltadone (**5**) on cancer cell lines (Figure 4).

The cytotoxicity evaluation of 15 cytotoxic sesquiterpene coumarins isolated from the dichloromethane extract of the roots of *Ferula huber-morathii* indicated that conferone (**1**), conferol (**2**), feselol (**3**), mogoltadone (**5**), and farnesiferol A acetate (**7**) show a remarkable cytotoxic effect on some cancer cell lines.

Previous cytotoxic activity studies performed on conferone (**1**) showed that the highest cytotoxic activity of this compound was observed on the ovarian carcinoma cells (CH1) with an IC_50_ of 7.8 µM [29]. Conferone (**1**) also showed moderate cytotoxic activity on colorectal (HT29; 20 µM), breast (MDA-MB-231; 30 µg/mL), colon (HCT116; 32 µM), ovarian (A2780; 32 µM), cervical (HeLa; 38 µM), lung (A549; 38 µM), cisplatin-resistant derivatives of the human ovarian (A2780P A2780/RCIS; 25 µM), melanoma (SK-MEL-28; 64 µM), and chronic myelogenous leukemia (K562; 86.12 µg/mL) cancer cell lines [29,66,67,68,69,70]. In addition, conferone (**1**) showed very low cytotoxic activity (>100 µM) on the MCF-7 breast cancer cell line and no cytotoxic effect on bladder cancer cells (5637) [46,66,69]. Based on the findings, conferone (**1**) displayed a moderate cytotoxic effect on the COLO 205, K-562, and MCF-7 cancer cell lines, as well as on the non-cancerous HUVEC cell line.

In a study conducted by Soltani et al. [67], the cytotoxic activities of feselol (**3**) on the HCT-116, HeLa, A549, and A2780 cancer cell lines were found to be 28, 35, 26, and 20 µM, respectively. Furthermore, some studies reported that feselol (**3**) did not show any cytotoxic activity on the bladder (TCC, 5637), K562, HeLa, and stomach (AGS) cancer cell lines [14,71,72,73,74].

Thus far, there has been no cytotoxic activity reported for farnesiferol A acetate (**7**); however, the findings herein suggest that farnesiferol A acetate has moderate cytotoxic activity against the MCF-7 breast cancer cell line. Nevertheless, its cytotoxic effect was weak against the rest of the cell lines.

**Figure 5:**
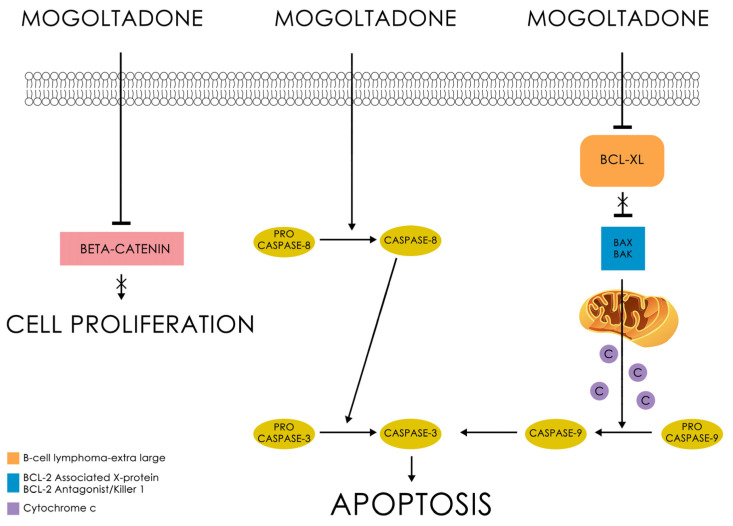
The cytotoxic effect of mogoltadone (**5**).

Few studies have explored the cytotoxic effect of mogoltadone (**5**) on different cell lines [29,66]. A study carried out by Valiahdi et al. [29] did not find any potent cytotoxic activity by mogoltadone (**5**) against the A549 and SK-MEL-28 cell lines. However, against the ovarian carcinoma cell line (CH1), mogoltadone (**5**) showed moderate cytotoxic effects [29]. Similarly, another study suggested that mogoltadone (**5**) had a low cytotoxic effect on the A2780/RCIS (>50) and MCF-7 (>100 µM) cancer cell lines [14,66]. Moreover, the combination of mogoltadone (**5**) with cisplatin significantly enhanced the cisplatin cytotoxicity on A2780/RCIS cells [66]. These results were similar to those of conferol (**2**) [75]. The current investigation indicated that mogoltadone (**5**) showed a moderate cytotoxic effect on the COLO 205, MCF-7, and K-562 cancer cell lines, except the non-cancerous HUVEC cell line, which is the control cell line. As there are no mechanistic studies regarding the selective cytotoxic activity of mogoltadone (**5**) reported in the literature, mogoltadone’s (**5**) cytotoxic effect mechanism was also investigated in the present study. Hence, this is the first study to show that mogoltadone (**5**) triggers the caspase activation and suppresses anti-apoptotic protein, Bcl-XL, and β-catenin. The accumulating evidence indicates that β-catenin has a central role in the malignant transformation of normal cells [76]. Therefore, the inhibition of β-catenin supports the cytotoxic effect of the molecule (Figure 5).

### 2.4. Molecular Docking Studies

In order to investigate the molecular interactions and evaluate the possible binding modes of conferol (**2**) and mogoltadone (**5**), which are the most active cytotoxic sesquiterpene coumarin ethers of *Ferula huber-morathii*, with Bcl-XL (PDB ID: 7LH7) and β-catenin proteins (PDB ID: 7AFW) and compare their active site amino acid interactions with those of established ligands (i.e., A-1293102 and compound **6**) as well as the reference compound (i.e., obatoclax) (Supplemental Materials, Figure S110), docking studies with the 7LH7 and 7AFW proteins were performed using the Schrödinger program suite [77]. The experimental bond conformations of native ligands were predicted by superposition Glide. Glide successfully replicated the experimental binding conformations of compound 6, A-1293102, with an RMSD of 0.0429 and 0.5471 Angstroms at the 7AFW, and 7LH7 binding sites, respectively (Supplementary Material, Figure S111).

#### 2.4.1. Molecular Docking of Conferol (**2**), Mogoltadone (**5**), and Ligands with β-Catenin

**Figure 6:**
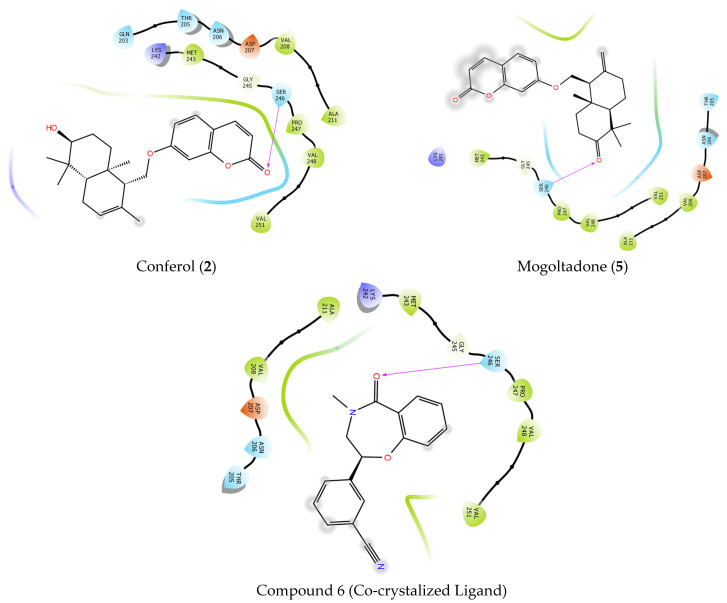
Docking 2D poses of the title compounds, co-crystalized ligand (compound 6), and their interactions with the active site of β-catenin protein crystal structure (PDB ID: 7AFW).

**Figure 7:**
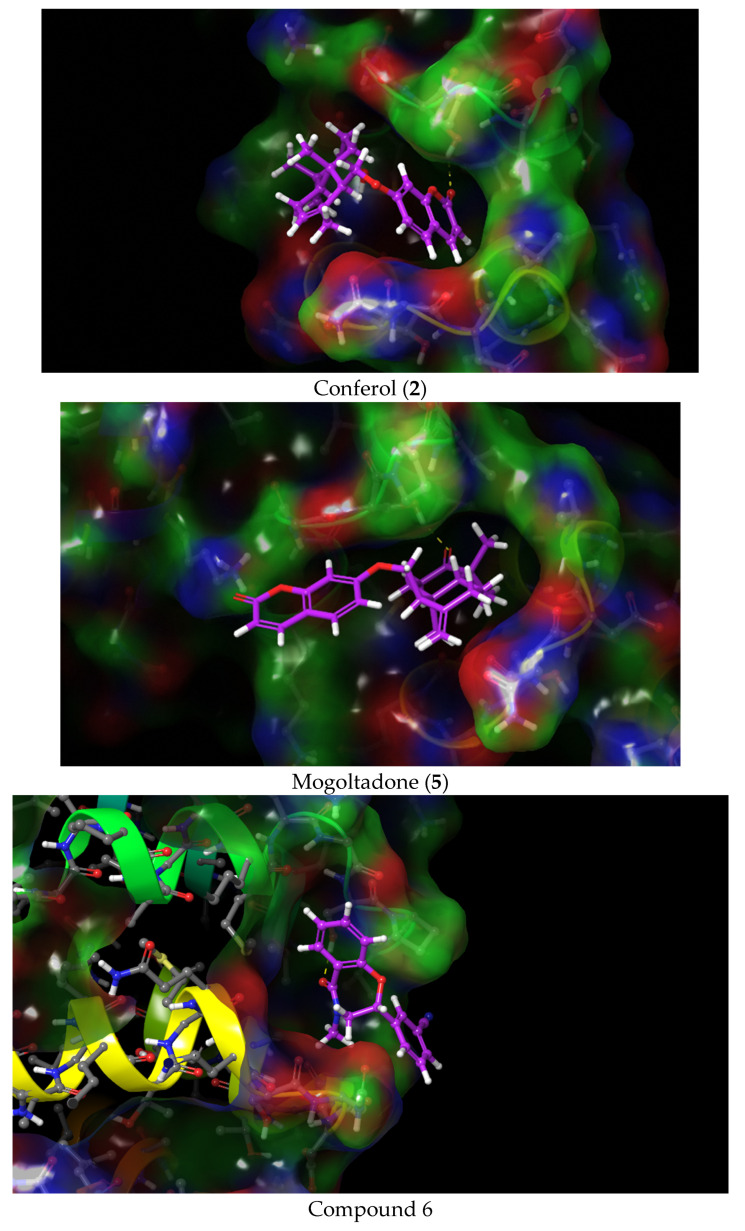
Docking 3D poses of the title compounds, co-crystalized ligand (compound 6), and their interactions with the active site of β-catenin protein crystal structure (PDB ID: 7AFW).

Native ligand compound 6 exhibited hydrophobic interactions and hydrogen bonding in the binding pocket of the β-catenin protein (PDB ID: 7AFW). Compound 6 formed a hydrogen bond with amino acid SER 246; in addition, it displayed hydrophobic interactions with amino acids PRO 247, VAL 248, ALA 239, and PRO 238. Conferol (**2**) and mogoltadone (**5**) formed a hydrogen bond with SER 246 similar to the native ligand. Hydrophobic interactions between mogoltadone (**5**) and the binding pocket of the receptor were not detected. Conferol (**2**) exhibited hydrophobic interactions with MET 243, GLY 245, ALA 211, and VAL 208 (Table 4 and Figure 6 and Figure 7).

#### 2.4.2. Molecular Docking of Conferol (**2**), Mogoltadone (**5**), and Ligands with Bcl-XL

**Figure 8:**
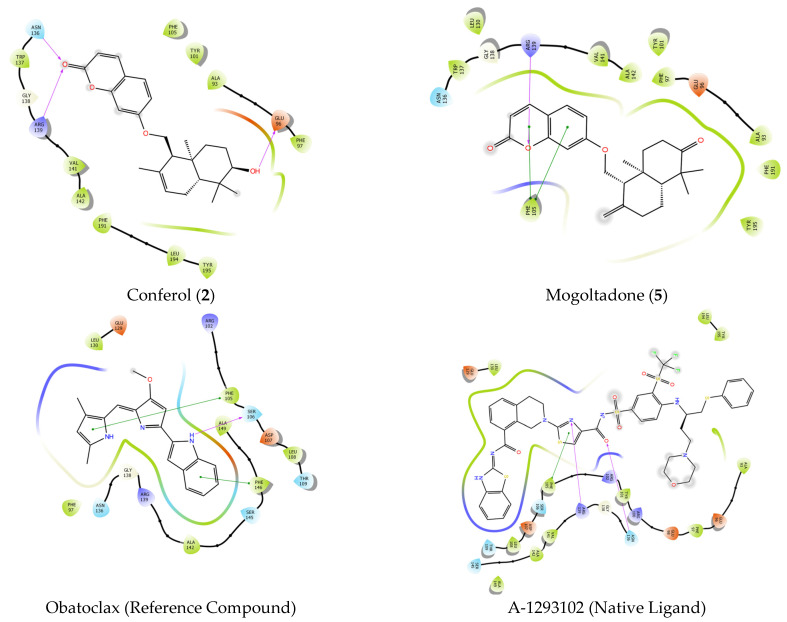
Docking 2D poses of conferol (**2**), mogoltadone (**5**), obatoclax, and co-crystalized ligand (A-1293102) and their interactions with the active site of Bcl-XL protein crystal structure (PDB ID: 7LH7).

**Figure 9:**
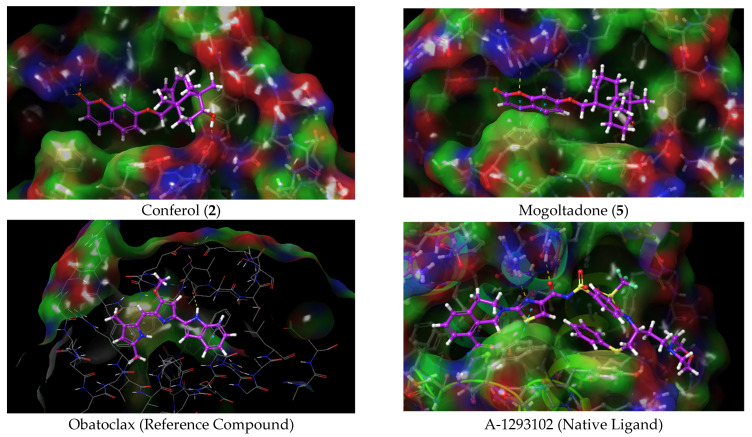
Docking 3D poses of conferol (**2**), mogoltadone (**5**), obatoclax, and co-crystalized ligand (A-1293102) and their interactions with the active site of Bcl-XL enzyme crystal structure (PDB ID: 7LH7).

The native ligand, A-1293102, exhibited hydrophobic interactions, hydrogen bonding, and Pi-Pi stacking in the binding pocket of the Bcl-XL protein. It showed two H-bond interactions with amino acids ASN 136 and ARG 139, Pi-Pi stacking interaction with PHE 105, and hydrophobic interactions with amino acids PHE 105, VAL 141, ALA 142, LEU 130, PHE 131, PHE 146, VAL 126, VAL 127, and ALA 149. The reference compound, obatoclax, showed a hydrogen bond with SER 106 and two Pi-Pi stacking interactions with PHE 105 and PHE 146. It also exhibited hydrophobic interactions with ALA 142, GLY 138, PHE 97, and ALA 149. Conferol (**2**) and mogoltadone (**5**) showed a hydrogen bond with ARG 139 and Pi-Pi stacking with PHE 105, similar to the native ligand. Conferol (**2**) exhibited two more H-bonds with ASN 136 and GLU 96, in addition to ARG 139. Mogoltadone (**5**) showed hydrophobic interactions with amino acid residues PHE 97, GLY 94, ALA 93, ALA 89, PHE 191, and LEU 108 (Table 5 and Figure 8 and Figure 9).

#### 2.4.3. Calculated ADME Properties of Conferol (**2**) and Mogoltadone (**5**)

The absorption, distribution, metabolism, excretion (ADME), and physicochemical properties such as molecular weight (MW), predicted aqueous solubility (QplogS), and predicted percent human oral absorption (PHAO) were calculated by the QikProp module of Schrödinger and are shown in Table 6. The data presented in the table indicate that conferol (**2**) and mogoltadone (**5**) showed no Lipinski’s rule violation.

#### 2.4.4. Evaluation of the Molecular Docking Study Results of Conferol (**2**) and Mogoltadone (**5**)

GX15-070 (obatoclax) is a molecule that triggers apoptosis by inhibiting enzymes, such as Bcl-2, BCL-XL, and MCL-1, among the Bcl-2 family of enzymes [78,79,80]. Obatoclax was used as a reference molecule against the molecules used in the docking studies against the Bcl-XL protein in this study. Both the conferol (**2**) and mogoltadone (**5**) used in the docking studies exhibited similar interactions to obatoclax and showed a docking score close to that of obatoclax. In addition, considering the interactions of the co-crystal ligand in the protein pocket, amino acids ARG 139 and ASN 136 are seen as important amino acids for hydrogen bonding, as is amino acid PHE 105 for Pi-Pi stacking interaction. The importance of these amino acids in inhibiting the Bcl-2 family of enzymes was also stated in another study conducted with the protein′s natural ligand [81]. As anticipated, mogoltadone (**5**) formed hydrogen bonds with amino acid ARG 139, and pi-pi stacking interaction was established with amino acid PHE 105 in both mogoltadone (**5**) and conferol (**2**).

It has been stated that some amino acids, such as SER 246, PRO 247, and MET 243 in the active region, play a critical role in the inhibition of β-catenin protein [82]. The docking studies in this study showed that the hydrogen molecules of the mogoltadone (**5**) and conferol (**2**) bonded with amino acid SER 246, and both molecules formed similar hydrophobic interactions with the co-crystalized ligand in the binding pocket of β-catenin.

As can be seen from the docking simulations, all of the compounds used in the docking studies could fit with the target enzymes by forming hydrogen bonds and hydrophobic and Pi-Pi stacking interactions. Based on the results of the computational studies, both mogoltadone (**5**) and conferol (**2**) complied with Lipinski’s rule of five, which is a sign of the drug-likeness of these molecules.

## 3. Materials and Methods

### 3.1. General Experimental Procedures

#### 3.1.1. Chemical Reagents, Solvents and Chromatographic Adsorbents

Sodium dichromate dihydrate, sodium borohydride (NaBH_4_), sodium bicarbonate (NaHCO_3_), anhydrous sodium sulfate (anhydr. Na_2_SO_4_), *p*-anisaldehyde, ethyl acetate (EtOAc), dichloromethane (DCM), dimethylaminopyridine (DMAP), triethylamine [(C_2_H_5_)_3_N], 3-dimethylaminopropylamine, and (*S*)-MTPA Chloride ((*S*)-(+)-α-methoxy-α-trifluoromethylphenylacetyl chloride) were purchased from Sigma-Aldrich (St. Louis, MO, USA). The hexane (Hxn), methanol (MeOH), ethanol (EtOH), acetonitrile (ACN), benzene, diethylether (Et_2_O), chloroform, cyclohexane, acetic anhydride (Ac_2_O), pyridine, and sulfuric acid (H_2_SO_4_) were purchased from Merck (Darmstadt, Germany). The Milli Q ultrapure water (W) was obtained from Millipore (Billerica, MA, USA). Sephadex LH-20 was purchased from GE Healthcare (Chicago, IL, USA). Silica gel 60 (0.063–0.200 mm) for the column chromatography was purchased from Merck, as was the LiChroprep RP-18 (40–63 µm).

#### 3.1.2. Spectroscopic Analyses

Optical rotations were recorded using an Autopol V Plus polarimeter (Rudolph Research Analytical, Hackettstown, NJ, USA). Infrared spectra were acquired using an Alpha FT-IR Spectrometer (Bruker, MA, USA). The absorbance spectra were obtained on a UV-Vis Spectrophotometer UV-1700 PharmaSpec (Shimadzu, Columbia, MD, USA). NMR spectra were acquired on a Bruker BioSpin spectrometer (Rheinstetten, Germany) operating at 500 MHz for ^1^H and 125 MHz for ^13^C and equipped with a 5-mm probe in CDCl_3_. HRESIMS analyses for the compounds were measured using the Thermo Scientific-Q Exactive LC-HRESIMS spectrometer (Thermo Scientific, Waltham, MA, USA).

#### 3.1.3. Column and Thin Layer Chromatography (TLC)

Analytical HPLC analyses were performed on a Shimadzu 10A model HPLC system (Shimadzu Analytical and Measuring Instruments, Kyoto, Japan) with a pump (LC-10AD), a diode-array detector (DAD) (SPD-M10A), and an autosampler (SIL-10AD). A Luna C18 column (250 × 4.60 mm, 5 micron; Phenomenex, Torrance, CA, USA) was used for the comparison of the dichloromethane extract of *Ferula huber-morathii* with reference compounds ferutinin (**17**) and elaeochytrin A (**18**) (isolated from *Ferula elaeochytris* Korovin) and cytotoxic sesquiterpene coumarin ethers of *F. huber-morathii* (i.e., compounds **1**–**15**) (Supplementary Materials Figure S40).

A Gilson PLC 2050 (Saint-Avé, France) preparative HPLC system with a Luna C18 column (150 × 21.2 mm, 5 micron; Phenomenex, Torrance, CA, USA) was used for the preparative HPLC purifications.

Supelco Silica gel 60 F_254_ PTLC plates (0.5, 1, and 2 mm; Merck, Darmstadt, Germany) were used for the preparative separations. Silica gel 60 F254 TLC plates (0.25 mm; Merck, Darmstadt, Germany) were used for the analytical separations. A UV lamp (Camag, Muttenz, Switzerland) with 254 and 366 nm wavelength detection capabilities was used for the visualization of plates. Compounds without chromophore groups were detected by spraying plates with freshly prepared 10% *p*-anisaldehyde in 10% ethanolic sulfuric acid, followed by heating.

### 3.2. Plant Material

Roots of *Ferula huber-morathii* were collected twice. The first batch was collected near the Zorabat village between Muş to Varto (37 km) in June 1983, identified by Prof. Ömer Saya and an herbarium sample was deposited in the Dicle University Herbarium (DUF 3704). The second batch was collected between Varto and Erzurum in July 2017, identified by Prof. Emine Akalın, and an herbarium sample was deposited in the herbarium of Faculty of Pharmacy, İstanbul University (ISTE 115970). 

### 3.3. Extraction and Isolation

Air-dried and coarsely pulverized root samples (second batch, collected in July 2017) of *Ferula huber-morathii* (707.7 g) were successively extracted with dichloromethane and methanol. However, the plant material was initially subjected to a maceration (2 × 1 L for 1 h) at room temperature with the respective extraction solvent (i.e., dichloromethane and methanol) in a Soxhlet extractor before the initiation of continuous Soxhlet extraction. The extracts obtained by maceration and continuous Soxhlet extraction were concentrated separately in vacuo in a rotary evaporator. Due to the close similarities between the TLC analyses of the extracts obtained by maceration vs. continuous Soxhlet extraction, they were combined and subjected to fractionation by column chromatography. The dichloromethane (DCM) extract was 50.5 g (yield % 7.1), and the methanol (MeOH) extract was 52.3 g (yield % 7.4) [83].

The DCM extract (6.5 g) was subjected to bioactivity-directed fractionation on a Sephadex LH-20 column (100 × 5 cm) using Hxn:DCM:MeOH (7:4:1 to 7:1:4 with 5% polarity increment) solvent system gradiently to afford 281 fractions. The fractions were combined based on their TLC profile and bioactivity test results. 

Fractions 97–107 (148.4 mg) were subjected to a reverse phase (RP-18) flash chromatography column (2 × 20 cm; W:ACN, *v*/*v* = 50:50 >> 0:100) to yield 48 subfractions. Compound (**7**) (farnesiferol A acetate, 8.5 mg) was obtained from subfractions 46–48 and purified by preparative silica gel TLC (0.5 mm; Hxn:EtOAc, 6:4). Reverse phase preparative HPLC was used to fractionate fractions 133–137 (137.5 mg), resulting in 7 subfractions (150 × 21.2 mm, Luna C18 column by a gradient with W:ACN, *v*/*v* = 60:40 >> 0:100). Fractions 2–4 were subjected to Sephadex LH-20 column (70 × 3 cm), eluted with Hxn:DCM:MeOH (*v*/*v* = 7:4.5:0.5 → 7:1:4) and obtained 12 subfractions. Compounds **1** (conferone, 7.2 mg), **4** (badrakemone, 1 mg), and **5** (mogoltadone, 4 mg) were then purified by preparative silica gel TLC (1 mm; Hxn: EtOAc, 7:3). Fraction 151 was crystalized (64.5 mg) with Et_2_O, and purified by preparative TLC (1 mm, Benzene:DCM:EtOAc:ACN, 8:8:2:1,) to yield **8** (gummosin, 13.5 mg). The column chromatography (Sephadex LH-20, 120 × 2.5 cm) of fractions 154–157 (548 mg) (isocratic, Hxn:Chloroform:EtOH, 25:25:1) afforded 45 subfractions. Subfractions 20–26 were combined (164.7 mg) and purified through silica gel (0.063–0.200 mm) column chromatography (70 × 2.5 cm) using gradient elution with the mobile phase Hxn:EtOAc (*v*/*v* = 100:0 >> 0:100) and conferol (**2**) (33.1 mg) was obtained from fractions 46–48. Subfractions 11–13 (11 mg) were also purified by preparative silica gel TLC (1 mm, Cyclohexane: EtOAc, 6.5:3.5), and samarcandin acetate (**15**) (9.3 mg) was isolated. Fractions 180–185 (300 mg) were subjected to RP-18 flash chromatography (2 × 20 cm column) using a W:ACN gradient mobile phase (*v*/*v* = 50:50 >> 0:100) to afford compounds **3** (feselol, 12.7 mg), **6** (farnesiferol A, 9 mg), **10** (ferukrin acetate, 8.6 mg), **12** (kellerin, 12 mg), and **13** (samarcandone, 2 mg), respectively. Fractions 218–222 (330 mg) were chromatographed on a RP-18 column (2 × 20 cm) with a W:ACN gradient mobile phase (*v*/*v* = 50:50 >> 1:100) to yield 30 subfractions. Fractions 5 and 6 were further purified by preparative silica gel TLC (2 mm, Hxn:EtOAc, 1:1) to yield compounds **14** (samarcandin, 100.7 mg) and **11** (deacetylkellerin, 26.2 mg). Fractions 232–240 (40 mg) were purified by preparative silica gel TLC (1mm, benzene: DCM: EtOAc: ACN, 2:2:2:1) and compound **9** (ferukrin, 5.6 mg) was isolated.

### 3.4. Chemical Transformations of Cytotoxic Sesquiterpene Coumarins

In order to corroborate the structures of parent or daughter compounds, some of the sesquiterpene coumarin ethers isolated from the dichloromethane extract of the roots of *Ferula huber-morathii* were subjected to a series of chemical transformations. Descriptions of the typical chemical transformation examples are as follows. Acetylation of samarcandin (**14**): samarcandin (10 mg) was dissolved in pyridine (0.5 mL) and Ac2O (0.5 mL) was added to the samarcandin solution in pyridine and left at room temperature for 16 h. After the work-up, samarcandin acetate (**15**) was recovered as a gum (12 mg). Oxidation of samarcandin (**14**): samarcandin (20 mg) was dissolved in Et_2_O (10 mL) and while stirring the Et_2_O layer, chromic acid solution (1 mL) [84] was added dropwise. The solution was stirred at room temperature for 2 h. The Et_2_O layer was separated and washed with saturated aqueous NaHCO_3_, dried over anhydr. Na_2_SO_4_, filtered, and then evaporated under a N_2_ stream to yield 17 mg samarcandone (**13**) as an amorphous white powder. Reduction of samarcandone (**13**): samarcandone (10 mg) was dissolved in anhydrous MeOH (5 mL); while stirring the solution, NaBH_4_ (15 mg) was added in small portions. The reaction mixture was stirred for 45 min at room temperature, and the excess NaBH_4_ was destroyed by the addition of saturated NaHCO_3_ solution. The reaction mixture was transferred into a separatory funnel and extracted with DCM (3 × 20 mL). The combined DCM layers were dried with anhydr. Na_2_SO4, filtered, and then evaporated to dryness in vacuo using a rotary evaporator to yield 7 mg isosamarcandin (**16**) as a gum.

Preparation of the (*R*)-MTPA ester of the samarcandin (24): samarcandin (14, 50 mg) was dissolved in dry dichloromethane (4.5 mL). (*S*)-MTPA chloride (47 mg), DMAP (65 mg), and triethylamine (30 µL) were added to this solution, and the reaction mixture was kept at room temperature for 16 h. Then, 3-dimethylaminopropylamine (35 µL) was added to the reaction mixture, and the solvent was evaporated to dryness under reduced pressure. The reaction mixture was purified on a PTLC using a hexane-ethyl acetate mixture (6:4) to yield the (*R*)-MTPA ester of the samarcandin (24, 67 mg) (Supplementary Materials Figure S99).

#### 3.4.1. (*R*)-MTPA Ester of Samarcandin **24**

Colorless crystal; T_m_: 173–4 °C, [α]_D_^20^: +5.88 (c 0.34, CH_2_Cl_2_); UV (MeOH) λ_max_ (log ε) nm: 202 (1.20), 221 (sh) (1.79), 294 (sh) (0.94), 324 (1.20) nm (Supplementary Materials Figure S108); IR υ_max_ (NaCl) cm^−1^: 3067, 2947, 2876, 1737, 1709, 1613, 1554, 1509, 1452, 1397, 1352, 1278, 1232,1183, 1166, 1123, 1011, 997, 836, 756, 720 cm^−1^ (Supplementary Materials Figure S107); ^1^H-NMR, ^13^C-NMR, 2D-COSY, HSQC, HMBC, NOESY spectra (Supplementary Materials Figures S100–S105), (+)-HRESIMS *m*/*z* 617.2721 [M + H]^+^ (calcd. for C_34_H_40_F_3_O_7_, 617.2726) (Supplementary Materials Figure S106).

#### 3.4.2. X-ray Crystal Structure Analysis of the (R)-MTPA Ester of the Samarcandin **24**

Single crystal X-ray diffraction experiments were carried out with a RIGAKU Rotating Anode Diffractometer equipped with confocal monochromator Mo Kα radiation. The structure was solved by direct methods (SHELXS97) [85] and refined by least-squares procedures on Fsqd (SHELXL97) [86]. The refinement was made with anisotropic displacement factors for all of the non-hydrogen atoms. All of the hydrogen atoms were calculated to their idealized positions and refined as riding atoms. The geometric calculations were carried out with the program Platon [87]. The crystal data and final parameters of the results of refinement are listed in Supplementary Materials Table S2. Fractional atomic coordinates, anisotropic displacement parameters, molecular dimensions, and geometric data have been deposited with the Cambridge Crystallographic Data Centre (CCDC 2080858). Copies of the data can be obtained, free of charge, on application to CCDC, 12 Union Road, Cambridge CB2 1EZ, UK, (fax: +44-1223-336033 or e-mail: deposit@ccdc.cam.ac.uk). For the crystal data and details of the structure determination of **24**, see Supplementary Materials Table S2.

### 3.5. Cell Culture Conditions

Human colorectal cancer (COLO 205, CCL-222), breast cancer (MCF-7, HTB-22), chronic myelogenous leukemia (K-562, CCL-243), and umbilical vein/vascular endothelium (HUVEC, CRL-1730) cell lines were obtained from the American Type Culture Collection. The COLO 205, MCF-7, K-562, and HUVEC cell lines were maintained in Roswell Park Memorial Institute (RPMI 164, Wisent, Montreal, QC, CANADA), Eagle′s Minimum Essential Medium (EMEM, Wisent, Montreal, QC, CANADA), Iscove’s Modified Dulbecco’s Medium (IMDM, Wisent, Montreal, QC, CANADA), and Dulbecco′s Modified Eagle Medium (DMEM, Wisent, Montreal, QC, CANADA) supplemented with 10% fetal bovine serum (Capricorn, Ebsdorfergrund, Germany), 100 U/mL penicillin, and 100 µg/mL streptomycin (Wisent, Montreal, QC, CANADA), respectively. All of the cell lines were cultured at 37 °C in a humidified atmosphere containing 5% CO_2_.

### 3.6. Cytotoxic Activity

A 3-[4,5-dimethylthiazol-2-yl]-5-[3-carboxymethoxyphenyl]-2-[4-sulfophenyl]-2H-tetrazolium, inner salt (MTS) cell viability assay was performed to evaluate the cytotoxic activities of the sesquiterpene coumarin ethers isolated from *Ferula huber-morathii*. HUVEC (1 × 10^4^), COLO 205 (3 × 10^4^), K-562 (3 × 10^4^), and MCF7 (5 × 10^4^) were plated in 96-well plates. Then, 24 h later, the tested compounds were dissolved in DMSO, and the cells were exposed to the different concentrations (131.58–1.028 µM) of the compounds for 72 h. Cisplatin and doxorubicin were used as a positive control. Next, an MTS/phenazine methosulfate (PMS) (Promega, Madison, WI, USA and Sigma, St. Louis, MO, USA respectively) mixture was added to each well and incubated for 1–4 h at 37 °C. The absorbance was read on a microplate reader (Biotek, Winooski, VT, USA) at 490 nm. The cytotoxic activities of the compounds were expressed as an IC_50_ value. The % inhibition of cell proliferation was calculated using the formula: % Inhibition = [1-(A490test/A490cont)] × 100, where A490test = absorbance of the test sample and A490cont = absorbance of the control sample [88]. The values of the 50% inhibition of the cell proliferation (IC_50_) were calculated by locating the x-axis values corresponding to one-half of the absorbance values of the sample.

#### 3.6.1. Caspase Activities

The caspase-3, -8, and -9 activities were detected by following the protocol of the caspase-3, -8, -9 colorimetric assay kits (Biovision, K106, K113, K119, Mountain View, CA, USA). The cells treated with mogoltadone (**5**) for 24 h were harvested and lysed with a RIPA (50 mM Tris-HCl, pH 8.0, 150 mM NaCl, 1% Nonidet P-40, 0.5% sodium deoxycholate and 0.1% SDS) cell lysis buffer. The lysed cells were centrifuged at 22,000 g for 30 min and the supernatant was collected in a tube. The samples (200 µg protein) were incubated with caspase substrates (final concentration, 200 µM) at 37° C for 2 h and read on a microplate reader at 405 nm. The fold-increase in enzyme activity was detected by comparison with the untreated control.

#### 3.6.2. Western Blot Analysis

To evaluate the effect of mogoltadone (**5**) on the caspase-3, Bcl-XL and β-catenin protein levels, the COLO 205 cell line was treated with mogoltadone (**5**) and incubated for 24 h. At the end of the incubation period, the treated cells were harvested. After that, the harvested cells were washed with cold phosphate buffered saline and lysed in a RIPA buffer. The samples were centrifuged at 24,000 g for 45 min at 4° C, and the supernatants were used to determine the procaspase-3, Bcl-XL, β-catenin, and β-actin protein levels by western blot analysis. The cellular proteins were separated on sodium dodecyl sulfate-polyacrylamide gels (SDS-PAGE, AnyKD mini protean TGX precast protein gel, Bio-Rad, Hercules, CA, USA, 4569033). The separated proteins were transferred to the polyvinylidene difluoride (PVDF) membrane using the Trans-Blot Turbo transfer system (Bio-Rad). The membrane was then blocked with a 5% non-fat milk blocking buffer and consecutively incubated with rabbit monoclonal Bcl-XL antibody (1:20,000, ab17884, Abcam), rabbit monoclonal caspase-3 antibody (1:3000, ab3235, Abcam, Cambridge, MA, USA), rabbit monoclonal beta-actin antibody (1:10,000, M01263, BOSTER, Beijing, China), and rabbit polyclonal β-catenin antibody (1/5,000, ab264263, Abcam, Cambridge, MA, USA) overnight at 4° C. Furthermore, the membrane was washed with Tris-Buffered Saline with Tween (TBST) solution and incubated with goat anti-rabbit Ig-G (H+L) HRP-conjugated antibody (1:5000, BA1054, BOSTER, Beijing, China) for 1 h at room temperature. Finally, the membrane was incubated with a chemiluminescent substrate for 5 min and the protein bands were visualized by the imaging system (Vilber Lourmat, Fusion FX5, Marne-la-Vallée, France).

#### 3.6.3. Statistical Analysis

Statistical data analysis was performed using an independent sample *t*-test with *p* < 0.05 considered statistically significant. Each experiment was performed three times.

### 3.7. Molecular Docking

Protein structures were obtained from the Protein Data Bank (PDB database, www.rcsb.org) to identify interactions between the ligands and the binding pocket of the proteins. Two target proteins were used in this study: compound **6**-encoded ligand-bound beta-catenin protein (PDB ID: 7AFW, 1.81 Å) [82] and Bcl-XL protein A-1293102 bound by a benzothiazole-derived ligand (PDB ID: 7LH7, 1.41 Å) [81]. First, the crystal structures of the enzymes were prepared for docking using the multistep Protein Preparation Wizard module of the Schrödinger Software Suite [77]. Missing hydrogen atoms were added, and water molecules, heteroatoms, and co-factors, except for the native ligand, were removed to optimize the protein structure. To define the ligand binding pocket of the protein, a receptor grid box was generated by the atomic coordinates of the native ligand using the Receptor Grid Generation implemented in Glide [77]. Then, the compounds were docked to the binding site using Glide, and docking was performed at Standard Precision (SP) mode. The isolated compounds, native ligands, and reference compound (obatoclax) were drawn with 2D Sketcher in Schrödinger Maestro, and the LigPrep module of the Schrödinger Software Suite [77] was utilized to generate the energy-minimized conformations and tautomers at pH 7.0 ± 2.0 using Epik. The determined chiralities from the 3D structures were minimized using the OPLS3 force field. The co-crystallized native ligands were also prepared and optimized using the LigPrep module of the Schrodinger Software Suite (Schrödinger, New York, NY, USA).

### 3.8. In Silico ADME Studies

QikProp [77] modules of Maestro of Schrödinger were used to determine some of the compounds′ pharmacokinetic and physicochemical properties. ADME descriptors were determined by considering Lipinski’s rules [89] and some critical pharmacokinetic parameters such as the molecular weight, logarithm of octanol-water partition coefficient (QPlogPo/w), and percent human oral absorption.

## 4. Conclusions

Fifteen cytotoxic sesquiterpene coumarin ethers (**1**–**15**) were isolated from the dichloromethane extract of the roots of *Ferula huber-morathii* using bioactivity-directed isolation studies. Their structures were determined by extensive spectroscopic techniques and chemical transformations. The absolute configuration of samarcandin (**14**) was unequivocally determined by the X-ray crystallographic analysis of its (*R*)-MTPA ester (**24**). The cytotoxic activities of the individual sesquiterpene coumarin ethers (**1**–**15**) were tested against the COLO 205, K-562, and MCF-7 cancer cell lines, as well as the non-cancerous HUVEC cell line. Based on the IC_50_ values, conferol (**2**) and mogoltadone (**5**) were the most potent sesquiterpene coumarins against the cancer cell lines. However, the cytotoxic selectivity of mogoltadone (**5**) against the cancer cell lines was higher than those of conferol (**2**) and induced higher caspase-3, 8, and 9 activations, specifically in the COLO 205 cell line. The Western Blot analysis showed that mogoltadone (**5**) suppressed the level of Bcl-XL and decreased the procaspase-3 level in the COLO 205 cell line. Furthermore, the effect of mogoltadone (**5**) on the β-catenin was investigated and it was found that the β-catenin levels were decreased in the COLO 205 cell line. In contrast, mogoltadone (**5**) did not significantly affect the Bcl-XL, caspase-3, or β-catenin protein levels of the non-cancerous HUVEC cell line, which may explain the cytotoxic specificity of mogoltadone (**5**) on the cancer cell lines. The in silico docking studies also showed that the interaction of mogoltadone (**5**) with the active sites of β-catenin and Bcl-XL was similar to the established ligands and reference compound (i.e., obatoclax).

These data indicate that both natural mogoltadone (**5**) isolated from *F. huber-morathii* and its semi-synthetically prepared analogs could be used as a potent anticancer compound for colorectal cancer (CRC).

## Figures and Tables

**Table 1:** Cytotoxic activities of *Ferula huber-morathii* root extracts.

Extracts	IC_50_ (µg/mL)			
	COLO 205	K-562	MCF-7	HUVEC
Dichloromethane Extract	29.52 ± 1.32	24.22 ± 1.33	63.09 ± 3.47	53.56 ± 0.74
Methanol Extract	>100	>100	>100	>100
Cisplatin *	12.21 ± 0.34	9.73 ± 0.30	35.52 ± 0.76	19.27 ± 1.57

* Positive control.

**Table 2:** ^1^ H-NMR (500 MHz) and ^13^C-NMR (125 MHz) data of compounds **1**, **2, 3,** 4, **5**, **6, 7**, **8**, **9**, **10**, **11**, **12**, **13**, **14**, and **15** (in CDCl_3_, δ in ppm, *J* in Hz).

Position	Conferone (1)		Conferol (2)		Feselol (3)		Badrakemone (4)	
	^1^H-NMR	^13^C-NMR	^1^H-NMR	^13^C-NMR	^1^H-NMR	^13^C-NMR	^1^H-NMR	^13^C-NMR
2	-	161.1	-	161.3	-	161.5	-	161.5
3	6.25; d; 9.5; 1H	113.1	6.23; d; 9.5; 1H	113.1	6.25; d; 9.4; 1H	113.2	6.25; d; 9,5; 1H	113.2
4	7.64; d; 9.5; 1H	143.4	7.63; d; 9.5; 1H	143.5	7.63; d; 9.4; 1H	143.5	7.62; d; 9.5; 1H	143.6
5	7.37; d; 8.6; 1H	128.7	7.35; d; 8.5; 1H	128.9	7.36; d; 8.5; 1H	128.9	7.36; d; 8.7; 1H	129
6	6.83; dd; 2.4; 8.6; 1H	113	6.83; dd; 2.4, 8.5; 1H	113.3	6.82; dd; 2.3; 8.5; 1H	113.3	6.82; dd; 2.4; 8.7; 1H	113.3
7	-	161.7	-	162.3	-	162.2	-	162.2
8	6.8; d; 2.4; 1H	101.2	6.81; d; 2.5; 1H	101.5	6.8; d; 2.3; 1H	101.4	6.81; d; 2.4; 1H	101.4
9	-	156	-	156.1	-	156.2	-	156.1
10	-	112.7	-	112.6	-	112.6	-	112.7
1′α	2.28; m; 1H *	38.5	1.68; m; 2H *	31.9	2.01; dt; 3.6, 9.8; 1H	37.9	2.09; ddd; 3.5; 6.3; 13.3; 1H	37.8
1′β	1.62; td; 4.2; 13.3; 1H				1.33; td; 4.3;13.2; 1H		1.81; td; 5.4; 13.3; 1H	
2′ α	2.75; td; 5.4; 14.6; 1H	34.4	1.96; m; 1H **	25.3	1.65; m; 2H	27.4	2.68; tdd; 6.2; 13.2; 15.3; 1H	34.7
2′β	2.28; m; 1H *		1.65; m; 1H *				2.41; ddd; 3.4; 5.3; 15.3; 1H	
3′	-	216.1	3.47; bt; 2.4; 1H	75.9	3.28; dd; 4.3; 11.4; 1H	79.2	-	216.2
4′	-	47.6	-	35.7	-	38.8	-	48.1
5′	1.68; dd; 4.4; 12.2; 1H	51.1	1.70; m; 1H *	43.5	1.28; dd; 5.2; 11.7; 1H	49.5	1.66; dd; 2.7; 12.5; 1H	55.2
6′α	2.13; dddd; 2.6; 4.8; 9.5; 19.4; 1H	23.9	1.96; m; 2H **	23.3	2.05; m; 2H *	23.4	1.57; dt; 6.8; 12.5; 1H	24.6
6′β	1.99; dt; 5.6; 17.3; 2H						1.68; dp; 2.5; 12.4; 1H	
7′α	5.6; dq; 1.9; 5.8; 1H	123.9	5.54; bs; 1H	124	5.56; brs; 1H	123.9	2.15; td; 3.4; 13.3; 1H	37.2
7′β	-		-		-		2.5; ddd; 2.5; 4.2; 13.3; 1H	
8′	-	132.5	-	132.6	-	132.4	-	145.8
9′	2.28; m; 1H *	53.1	2.32; bs; 1H	53.7	2.23; brs; 1H	53.9	2.3; t; 5.9; 1H	54.2
10′	-	35.9	-	37.4	-	35.9	-	38.8
11′a	4.07; dd; 5.3; 9.8; 1H	66.7	4.02; dd; 5.8, 9.4; 1H	67.2	4.01; dd; 5.9; 9.7; 1H	67.2	4.22; m; 2H	65.8
11′b	4.17; dd; 3.4; 9.8; 1H		4.17; dd; 3.3, 9.4; 1H		4.17; dd; 3.4; 9.7; 1H			
12′a	1.7; brt; 3H	21.5	1.69; bs; 3H *	21.9	1.68; d; 3.8; 3H	21.8	4.59; brs; 1H	108.9
12′b							4.98; brs; 1H	
13′	1.08, s; 3H	25.2	0.93; s; 3H	22.5	1.02; s; 3H	28.2	1.13; s; 3H	26
14′	1.12; s; 3H	22.3	0.96; s; 3H	28.2	0.9; s; 3H	15.4	1.06; s; 3H	22.1
15′	1.13; s; 3H	14.5	0.91; s; 3H	14.9	0.88; s; 3H	15	1.04; s; 3H	15
**Position**	**Mogoltadone (5)**		**Farnesiferol A (6)**		**Farnesiferol A acetate (7)**		**Gummosin (8)**	
	**^1^H-NMR**	**^13^C-NMR**	**^1^H-NMR**	**^13^C-NMR**	**^1^H-NMR**	**^13^C-NMR**	**^1^H-NMR**	**^13^C-NMR**
2	-	161.2	-	161.9	-	161.3	-	161.5
3	6.24; d; 9.5; 1H	113	6.25; d; 9.5; 1H	113.1	6.25; d; 9.4	113.1	6.22; d; 9.5; 1H	112.9
4	7.62; d; 9.5; 1H	143.4	7.63; d; 9.5; 1H	143.5	7.63; d; 9.4; 1H	143.6	7.6; d; 9.5; 1H	143.6
5	7.35; d; 8.5; 1H	128.7	7.35; d; 8.6; 1H	128.7	7.36; d; 8.3; 1H	128.9	7.32; d; 9.3; 1H	128.7
6	6.78; dd; 2.5; 8.5; 1H	113	6.81; dd; 2.4; 8.6; 1H	113.4	6.8; dd; 2.1; 8.3; 1H	113.2	6;81; dd; 2.4; 9.3; 1H	113.3
7	-	161.7	-	161.9	-	161.9	-	162.3
8	6.77; d; 2.5; 1H	101.4	6.8; d; 2.4; 1H	101.7	6.79; d; 2.1; 1H	101.9	6.75; d; 2.4; 1H	101.8
9	-	155.7	-	155.9	-	155.9	-	155.9
10	-	112.6	-	112.6	-	112.6	-	112.5
1′α	1.91; td; 4.5; 13.6; 1H	35.5	1.37; dt; 3.2; 13.0; 1H	35	1.73; 2H **	24.1	1.99; tdd; 2.2; 3.3; 13.9; 1H	25.7
1′β	1.69; ddd; 2.8; 5.9; 13.6; 1H		1.63; td; 5.6; 12.0; 1H				1.62; dq; 3.1; 13.9; 1H	
2′ α	2.76; td; 5.7; 14.7; 1H	35.1	1.7; qd; 2.5; 12.5; 2H	27.7	1.39; 1H *	34.5	1.03; dt; 3.1; 12.5; 1H	29.3
2′β	2.36; m; 1H *				1.72; 1H **		2.05; dd; 2.1; 13.2; 1H	
3′	-	216.2	3.25; dd; 5.2; 10.2; 1H	79.2	4.5; dd; 6.3; 8.5; 1H	80.9	3.45; dd; 2.1; 3.5; 1H	76.2
4′	-	47.8	-	39.2	-	38.1	-	37.7
5′	1.54; qd; 4.4; 13.03; 1H	47.6	1.31; dd; 2.8; 12.6; 1H	46.6	1.41; m; 1H *	46.7	1.77; dd; 3.1; 12.9; 1H	40.8
6′ α	1.78; dd; 3.15; 12.6; 1H	23.8	1.73; dq; 2.8; 10.5; 1H	23.1	1.7; m; 2H **	23	1.60; dq; 2.8; 13.8; 1H	23.1
6′ β	1.64; m; 1H *		1.43; qd; 4.4; 12.9; 1H				1.37; qd; 4.2; 13.0; 1H	
7′α	2.1; td; 4.8; 13.6; 1H	32	2.04; td; 5.1; 13.5; 1H	32.5	2.05; m; 1H ***	32.4	2.09; tdt; 2.3; 6.6; 14.1; 1H	32.7
7′β	2.39; dt; 3.27; 13.6; 1H		2.33; dt; 3.1; 12.4; 1H		2.35; dd; 3.9; 14.1; 1H		2.31; ddt; 1.8; 4.0; 14.1; 1H	
8′	-	146	-	146.8	-	146.6	-	147.1
9′	2.35; m; 1H *	56	2.21; t; 6.1; 1H	56.7	2.21; t; 6.0; 1H	56.7	2.18; brt; 6.2; 1H	57.1
10′	-	37.3	-	37.8	-	37.7	-	37.7
11′a	4.03; dd; 6.1; 9.9; 1H	67.9	4.02; dd; 6.3; 9.8; 1H	68.2	4.02; dd; 6.0; 9.9; 1H	68.3	4.07; dd; 6.9; 9.9; 1H	68
11′b	4.25; dd; 6.1; 9.9; 1H		4.29; dd; 5.8; 9.8; 1H		4.29; dd; 6.0; 9.9; 1H		4.39; dd; 5.4; 9.9; 1H	
12′a	4.80; t; 1.9; 1H	112.1	4.72; t; 1.9; 1H	111.5	4.73; brs; 1H	111.5	4.69; t; 2.0; 1H	111.2
12′b	4.89; t; 2.2; 1H		4.83; t; 2.1; 1H		4.82; t; 2.2; 1H		4.79; t; 2.2; 1H	
13′	1.13; s; 3H	25.8	1.04; s; 3H	28.6	0.92; s; 3H	16.9	0.84; s; 3H	22.5
14′	1.05; s; 3H	22.3	0.81; s; 3H	15.4	0.89; s; 3H	28.4	0.98; s; 3H	28.5
15′	1.2; s; 3H	21.1	0.99; s; 3H	22.2	1.01; s; 3H	22.2	0.98; s; 3H	22.1
CH_3_- (OAc)	-	-	-	-	2.06; s; 3H	21.5	-	-
C=O (OAc)	-	-	-	-	-	171	-	
**Position**	**Ferukrin (9)**		**Ferukrin acetate (10)**		**Deacetylkellerin (11)**		**Kellerin (12)**	
	**^1^H-NMR**	**^13^C-NMR**	**^1^H-NMR**	**^13^C-NMR**	**^1^H-NMR**	**^13^C-NMR**	**^1^H-NMR**	**^13^C-NMR**
2	-	161.3	-	161.4	-	161.4	-	161.3
3	6.2; d; 9.5; 1H	113.1	6.26; d; 9.5; 1H	113.3	6.23; d; 9.5; 1H	113.2	6.25; d; 9.4; 1H	113.6
4	7.6; d; 9.5; 1H	143.6	7.64; d; 9.5; 1H	143.6	7.62; d; 9.5; 1H	143.5	7.63; d; 9.4; 1H	143.5
5	7.35; d; 8.6; 1H	129.2	7.37; d; 8.5; 1H	128.7	7.34; d; 8.4; 1H	129.1	7.37; d; 8.6; 1H	128.9
6	6.78; dd; 2.4; 8.6; 1H	112.7	6.79; dd; 2.2; 8.5; 1H	112.5	6.82; dd; 2.5; 8.4; 1H	113.1	6.86; dd; 2.4; 8.6; 1H	113.1
7	-	161.8	-	161.7	-	162.1	-	162.1
8	6.75; d; 2.4; 1H	101.3	6.78; d; 2.2; 1H	101.4	6.81; d; 2.5; 1H	101.7	6.82; d; 2.4; 1H	101.3
9	-	155.8	-	155.9	-	156	-	156.2
10	-	112.7	-	112.7	-	112.8	-	112.9
1′α	1.46; td; 3.1; 12.7; 1H	35.5	1.38; dt; 3.8; 12.2; 1H	35.4	1.04; dt; 3.0; 12.7; 1H	29.9	1.09; dt; 3.9; 12.9; 1H	31
1′β	1.32; dt; 3.1; 12.7; 1H		1.59; td; 3.6; 13.2; 1H		1.93; td; 3.7; 13.3; 1H		1.76; d; 3.2; 1H	
2′α	1.69; m; 1H *	27.2	1.75; m; 1H *	23.5	1.55; m; 1H *	25.4	2; tt; 3.1; 14.2; 1H	23
2′β	1.59; dq; 5.5; 13.3; 1H		1.68; dd; 4.3; 8.7; 1H		2.04; tt; 3.1; 13.7; 1H		1.6; dq; 3.4; 14.9; 1H	
3′	3.12; dd; 4.4, 11.5; 1H	78.9	4.4; dd; 4.65; 11.6; 1H	80.8	3.4; t; 2.9; 1H	76.3	4.63; t; 2.8; 1H	78.6
4′	-	37.5	-	37.8	-	37.8	-	37.1
5′	1.38; dd; 2.1; 8.8; 1H	48.5	1.51; dd; 1.8; 7.3; 1H	48.6	1.8; m; 1H ***	42.5	1.93; dd; 2.5; 12.3; 1H	43.8
6′α	1.53; dt; 3.4; 10.5; 1H	18.3	1.73; m; 1H *	18.2	1.44; dd; 5.9; 1.7; 1H	18.3	1.69; qd; 5.1; 12.3; 1H	18.1
6′ β	1.68; m; 1H *		1.56; dd; 2.5; 6.4; 1H		1.68; m; 1H **		1.49; dq; 2.9; 12.9; 1H	
7′ α	1.68; m; 1H *	39.5	1.73; m; 1H *	39.7	1.73; td; 2.4; 7.6; 1H	39.5	1.77; d; 3.1; 2H	40
7′ β	1.71; m; 1H *		1.73; m; 1H *		1.68; m; 1H **			
8′	-	73.3	-	73.2	-	73.8	-	73.8
9′	1.5; t; 2.4; 1H	57.8	1.53; t; 3.2; 1H	57.4	1.53; m; 1H *	58.4	1.53; t; 3; 1H	58.1
10′	-	37.9	-	37.6	-	38	-	37.9
11′a	4.05; dd; 3.2; 10.3; 1H	67.7	4.07; dd; 3.3; 10.4; 1H	67.5	4.06; dd; 3.1; 10.3; 1H	68.1	4.17; t; 2.8; 2H	67.8
11′b	4.08; dd; 2.4; 10.3; 1H		4.1; dd; 2.7; 10.4; 1H		4.2; dd; 3.1; 10.3; 1H			
12′a	1.26; s; 3H	31.6	1.29; s; 3H	31.7	1.29; s; 3H	31.7	1.32; s; 3H	31.9
12’b								
13′	0.78 s 3H	15.6	0.9; s; 3H	16.7	0.87; s; 3H	22.1	0.89; s; 3H	28.4
14′	0.99; s; 3H	28.7	0.91; s; 3H	28.5	0.98; s; 3H	28.6	0.93; s; 3H	21.8
15′	1.29; s; 3H	24.3	1.36; s; 3H	24.2	1.34; s; 3H	24.3	1.36; s; 3H	24.4
CH_3_- (OAc)	-	-	2.02; s; 3H	21.3	-	-	1.78; s; 3H	21.2
C=O (OAc)	-	-	-	171	-	-	-	170.6
**Position**	**Samarcandone (13)**		**Samarcandin (14)**		**Samarcandin acetate (15)**			
	**^1^H-NMR**	**^13^C-NMR**	**^1^H-NMR**	**^13^C-NMR**	**^1^H-NMR**		**^13^C-NMR**	
2	-	161.3	-	161.5	-		161.3	
3	6.26; d; 9.5; 1H	113.5	6.22; d; 9.6; 1H	113.2	6.25; d; 9.3; 1H		113.3	
4	7.64; d; 9.5; 1H	143.5	7.61; d; 9.6; 1H	143.6	7.63; d; 9.3; 1H		143.5	
5	7.37; d; 8.6; 1H	128.9	7.33; d; 8.5; 1H	128.8	7.36; d; 8.5; 1H		128.9	
6	6.85; dd; 2.4; 8.6; 1H	113.3	6.83; dd; 2.5; 8.5; 1H	113.3	6.86; dd; 2.3; 8.5; 1H		113.4	
7	-	161.6	-	161.9	-		161.9	
8	6.91; d; 2.4; 1H	101.7	6.88; d; 2.5; 1H	101.7	6.90; d; 2.3; 1H		101.8	
9	-	156	-	156	-		156	
10	-	113.1	-	112.7	-		112.8	
1′ α	2.03; ddd; 3.9; 7.3; 13.4; 1H	38.7	1.44; dt; 13.4; 3.4; 1H	32.9	1.51; m; 2H *		33.6	
1′ β	1.73; ddd; 7.0; 10.9; 13.4; 1H		1.66; dt; 3.4; 13.4; 1H					
2′ α	2.56; ddd; 7.3; 10.9; 15.9; 1H	33.9	1.57; m; 1H **	25.2	1.66; m; 1H ***		22.7	
2′ β	2.45; dt; 3.9; 7.0; 16.1; 1H		1.92; m; 1H *		1.88; m; 1H **			
3′	-	216.5	3.42; bt; 2.6; 1H	75.7	4.66; bt; 2.6; 1H		77.8	
4′	-	47.4	-	37.5	-		36.7	
5′	1.58; m; 1H *	54.8	1.51; bd; 12.6; 1H **	48.5	1.50; m; 1H *		49.7	
6′ α	1.67; dq; 3.1; 13.4; 1H	21.4	1.34; bdq; 3.6, 12.6;1H	20.1	1.35; bdq; 3.5; 12.7; 1H		19.9	
6′ β	1.48; td; 2.9; 12.4; 1H		1.58; m; 1H **		1.63; m; 1H ***			
7′ α	1.98; dt; 3.1; 12.4; 1H	43.4	1.92; m; 1H *	44.2	1.94; m; 1H **		44.1	
7′ β	1.58; m; 1H *		1.55; m; 1H **		1.58; m; 1H ***			
8′	-	72.5	-	72.7	-		72.8	
9′	1.86; t; 5.3; 1H	58.5	1.85; bt; 5.2; 1H	59.4	1.87; bt; 5.1; 1H **		59.4	
10′	-	37.6	-	38	-		37.8	
11′a	4.21; dd; 5.6; 10.0; 1H	66.6	4.17; dd; 5.4; 9.7; 1H	66.7	4.19; dd; 5.3; 9.7; 1H		66.9	
11′b	4.42; dd; 5.1; 10.0; 1H		4.36; dd; 4.6; 9.7; 1H		4.36; dd; 5.3; 9.7; 1H			
12′a	1.29; s; 3H	24.7	1.22; s; 3H	24.7	1.25; s; 3H		24.9	
12′b								
13′	1.13; s; 3H	26.8	0.96; s; 3H	28.5	0.88; s; 3H		28.1	
14′	1.06; s; 3H	21.5	0.83; s; 3H	22.2	0.90; s; 3H		21.9	
15′	1.07; s; 3H	15.8	0.93; s; 3H	16.1	0.96; s; 3H		16	
CH_3_- (OAc)	-	-	-	-	2.08; s; 3H		21.4	
C=O (OAc)	-	-	-	-	-		170.7	

*, **, *** Overlapped or partially overlapped signals.

**Table 3:** Cytotoxic activities of sesquiterpene coumarins isolated from the roots of *Ferula huber-morathii*.

Compounds	IC_50_ (µM) ^a^			
	COLO 205	K-562	MCF-7	HUVEC
Conferone (1)	27.63 ± 0.69	55.50 ± 0.94	34.02 ± 0.68	46.12 ± 0.99
Conferol (2)	11.19 ± 0.68	35.23 ± 0.89	15.95 ± 0.46	61.03±0.29
Feselol (3)	38.41 ± 0.80	72.48 ± 0.74	35.95 ± 1.29	38.75 ± 0.83
Badrakemone (4)	>200	>200	>200	>200
Mogoltadone (5)	31.71 ± 0.15	21.11 ± 0.85	30.45 ± 0.60	>200
Farnesiferol A (6)	>200	>200	>200	74.43 ± 1.19
Farnesiferol A acetate (7)	66.03 ± 1.89	52.71 ± 0.90	27.40 ± 0.96	64.53 ± 1.56
Gummosin (8)	>200	95.53 ± 4.87	>200	174.78 ± 0.28
Ferukrin (9)	>200	>200	81.69 ± 1.96	>200
Ferukrin acetate (10)	105.72 ± 1.35	88.42 ± 0.85	>200	>200
Deacetylkellerin (11)	>200	>200	47.62 ± 0.40	>200
Kellerin (12)	51.05 ± 1.57	78.14± 3.13	18.24 ± 0.12	99.39 ±1.63
Samarcandone (13)	125.14 ± 2.13	>200	>200	190.44 ± 5.20
Samarcandin (14)	170.03 ± 3.62	143.03 ± 1.67	83.27 ± 0.39	169.16 ± 2.68
Samarcandin acetate (15)	70.29 ± 0.65	78.67 ± 1.65	>200	>200
Cisplatin ^b^	111.87 ± 3.11	8.10 ± 0.25	64.22 ± 5.25	40.68 ± 1.12
Doxorubicin ^c^	0.08 ± 0.00	0.33 ± 0.02	2.83 ± 0.25	0.20 ± 0.02

^a^ 50% inhibitory concentrations in COLO 205, K-562, MCF-7, and HUVEC cells in the test assay. Values are the means ± standard deviations obtained for at least three independent experiments. ^b,c^ Positive controls.

**Table 4:** Docking scores of conferol (**2**), mogoltadone (**5**), and compound 6 (co-crystalized ligand) and their interactions with the active site of β-catenin protein crystal structure (PDB ID: 7AFW).

Ligands	Docking Score	H-Bond	Pi-Pi Stacking
Compound 6	−5544	SER 246	-
Conferol (2)	−3881	SER 246	-
Mogoltadone (5)	−3526	SER 246	-

**Table 5:** Docking scores of conferol (**2**), mogoltadone (**5**), A-1293102, and obatoclax and their interactions with the active site of Bcl-XL protein crystal structure (PDB ID: 7LH7).

Ligands	Docking Score	H-Bond	Pi-Pi Stacking
A-1293102	−14,792	ARG139, ASN 136	PHE 105
Obatoclax	−7744	SER 106	PHE 105, PHE 146
Conferol (2)	−4935	ARG 139, ASN 136, GLU 96	
Mogoltadone (5)	−4811	ARG 139	PHE 105

**Table 6:** ADME calculation results for conferol (**2**) and mogoltadone (**5**).

Compound	MW ^a^	CNS ^b^	donorHB ^c^	accptHB ^d^	QPlogPo/w ^e^	QPlogS ^f^	QPlogBB ^g^	QPlogKhsa ^h^	PHOA ^i^	Rule of Five ^j^
Conferol (**2**)	382,499	0	1	5	4,14	−5456	−0.665	0.697	100	0
Mogoltadone (**5**)	380,483	0	0	5	3,97	−5309	−0.691	0.539	100	0

^a^ MW: Molecular Weight. ^b^ CNS: Predicted central nervous system activity on a –2 (inactive) to +2 (active) scale. ^c^ Donor HB: Estimated number of hydrogen bonds that would be donated by the solute to water molecules in an aqueous solution. ^d^ Accpt HB: Estimated number of hydrogen bonds that would be accepted by the solute from water molecules in an aqueous solution. ^e^ QplogPo/w: Predicted octanol/water partition coefficient. ^f^ QplogS: Predicted aqueous solubility, log S. S in mol dm^−3^ is the concentration of the solute in a saturated solution that is in equilibrium with the crystalline solid. ^g^ QplogBB: Predicted brain/blood partition coefficient. ^h^ QplogKhsa: Prediction of binding to human serum albumin. ^i^ PHOA: Predicted human oral absorption on 0 to 100% scale. ^j^ Rule of Five Number of violations of Lipinski’s rule of five. The rules are: mol_MW < 500, QPlogPo/w < 5, donorHB ≤ 5, accptHB ≤ 10.

## Data Availability

Data is contained within the article and Supplementary Materials.

## Supplementary Materials

The following supporting information can be downloaded at https://www.mdpi.com/article/10.3390/ph16060792/s1, Figure S1; Bioactivity-guided isolation scheme for the cytotoxic sesquiterpene coumarins of the dichloromethane root extract of *Ferula huber-morathii*; Figures S2–S91, S93, S97–S98, S100–S109; NMR spectra of the sesquiterpene coumarins **1**–**16**, reference compounds **17** and **18**, and spectra of (*R*)-MTPA ester of samarcandin (**24**); Figure S92; Chemical transformations of the cytotoxic sesquiterpene coumarins; Figure S94; Previously proposed structures of sesquiterpene compounds isolated from the chloroform extract of *Ferula huber-morathii* [38]; Figure S95; TLC comparison of the dichloromethane extracts of *Ferula huber-morathii*, reference compounds **17** and **18**, and isolated cytotoxic sesquiterpene coumarins **1**–**15**; Figure S96; HPLC comparison of the sesquiterpene coumarins of the dichloromethane extract of *Ferula huber-morathii* and reference compounds **17** and **18**; Figure S99; Preparation of the (*R*)-MTPA ester of samarcandin (**24**); Figure S110; Structures of Bcl-XL and β-catenin ligands; Figure S111; Validation of the docking models. Superposition of docked pose and experimental binding conformation (green) of native ligands in the binding pocket of 7AFW (left), 7LH7 (right); Table S1; Physical appearance and comparison of optical rotation values of isolated sesquiterpene coumarins (**1**–**15**) with literature data [65,90]; Table S2; Crystal data and details of the structure of (*R*)-MTPA ester of samarcandin (**24**).
