# Supplementary material for: Sesquiterpene Coumarin Ethers with Selective Cytotoxic Activities from the Roots of Ferula huber-morathii Peşmen (Apiaceae) and Unequivocal Determination of the Absolute Stereochemistry of Samarcandin
Source: Pharmaceuticals (Basel). 2023 May 26;16(6):792. doi: 10.3390/ph16060792 (PMC10305617; doi:10.3390/ph16060792)
Supplement: Supplementary file 1 [file pharmaceuticals-16-00792-s001.zip › pharmaceuticals-2396985-supplementary.pdf]

Supplementary Materials

Sesquiterpene Coumarin Ethers with Selective Cytotoxic Activities from the Roots of *Ferula huber-morathii* Peşmen (Apiaceae) and Unequivocal Determination of the Absolute Stereochemistry of Samarcandin

Fatma Memnune Eruçar [1], Fadıl Kaan Kuran [1], Gülsüm Altıparmak Ülbegi [2], Süheyla Özbey [3], Şule Nur Karavuş [4], Gülşah Gamze Arcan [2], Seçil Yazıcı Tütüniş [1], Nur Tan [1], Pınar Aksoy Sağırlı [2] and Mahmut Miski [1,*]

[1] Department of Pharmacognosy, Faculty of Pharmacy, İstanbul University, 34116 İstanbul, Türkiye; memnune.erucar@istanbul.edu.tr (F.M.E.); kaankuran@istanbul.edu.tr (F.K.K.); secilyaz@istanbul.edu.tr (S.Y.T.); nurtan@istanbul.edu.tr (N.T.)
[2] Department of Biochemistry, Faculty of Pharmacy, İstanbul University, 34116 İstanbul, Türkiye; gulsum.altiparmakulbegi@istanbul.edu.tr (G.A.Ü.); gamze.arcan@istanbul.edu.tr (G.G.A.); aksoyp@istanbul.edu.tr (P.A.S.)
[3] Department of Engineering Physics, Faculty of Engineering, Hacettepe University, 06800 Ankara, Türkiye; sozbey@hacettepe.edu.tr
[4] Department of Pharmacognosy, School of Pharmacy, İstanbul Medipol University, 34810 İstanbul, Türkiye; sule.karavus@medipol.edu.tr
* Correspondence: mahmut.miski@gmail.com; Tel.: +90-545-550-4455

Table of Contents

Figure S1. Bioactivity-guided isolation scheme for the cytotoxic sesquiterpene coumarins of the dichloromethane root extract of *Ferula huber-morathii*........................ 6
Figure S2. ^{1}H-NMR spectrum (500 MHz, $CDCl_3$) of conferone (**1**)................................. 8
Figure S3. ^{13}C-NMR spectrum (125 MHz, $CDCl_3$) of conferone (**1**)................................. 9
Figure S4. ^{1}H-^{1}H COSY spectrum ($CDCl_3$) of conferone (**1**)... 10
Figure S5. HSQC spectrum ($CDCl_3$) of conferone (**1**)... 11
Figure S6. HMBC spectrum ($CDCl_3$) of conferone (**1**)... 12
Figure S7. NOESY spectrum ($CDCl_3$) of conferone (**1**)... 13
Figure S8. ^{1}H-NMR spectrum (500 MHz, $CDCl_3$) of conferol (**2**)................................. 14
Figure S9. ^{13}C-NMR (APT) spectrum (125 MHz, $CDCl_3$) of conferol (**2**)....................... 15
Figure S10. ^{1}H-^{1}H COSY spectrum ($CDCl_3$) of conferol (**2**)... 16
Figure S11. HSQC spectrum ($CDCl_3$) of conferol (**2**)... 17
Figure S12. HMBC spectrum ($CDCl_3$) of conferol (**2**)... 18
Figure S13. NOESY spectrum ($CDCl_3$) of conferol (**2**)... 19
Figure S14. ^{1}H-NMR spectrum (500 MHz, $CDCl_3$) of feselol (**3**)................................... 20
Figure S15. ^{13}C-NMR spectrum (125 MHz, $CDCl_3$) of feselol (**3**)................................... 21
Figure S16. ^{1}H-^{1}H COSY spectrum ($CDCl_3$) of feselol (**3**).. 22
Figure S17. HSQC spectrum ($CDCl_3$) of feselol (**3**).. 23
Figure S18. HMBC spectrum ($CDCl_3$) of feselol (**3**).. 24
Figure S19. NOESY spectrum ($CDCl_3$) of feselol (**3**).. 25
Figure S20. ^{1}H-NMR spectrum (500 MHz, $CDCl_3$) of badrakemone (**4**)....................... 26
Figure S21. ^{13}C-NMR spectrum (125 MHz, $CDCl_3$) of badrakemone (**4**)...................... 27
Figure S22. ^{1}H-^{1}H COSY spectrum ($CDCl_3$) of badrakemone (**4**).................................. 28
Figure S23. HSQC spectrum ($CDCl_3$) of badrakemone (**4**)... 29
Figure S24. HMBC spectrum ($CDCl_3$) of badrakemone (**4**)... 30
Figure S25. NOESY spectrum ($CDCl_3$) of badrakemone (**4**)... 31
Figure S26. ^{1}H-NMR spectrum (500 MHz, $CDCl_3$) of mogoltadone (**5**)....................... 32
Figure S27. ^{13}C-NMR (APT) spectrum (125 MHz, $CDCl_3$) of mogoltadone (**5**)........... 33
Figure S28. ^{1}H-^{1}H COSY spectrum ($CDCl_3$) of mogoltadone (**5**).................................. 34
Figure S29. HSQC spectrum ($CDCl_3$) of mogoltadone (**5**)... 35
Figure S30. HMBC spectrum ($CDCl_3$) of mogoltadone (**5**)... 36
Figure S31. NOESY spectrum ($CDCl_3$) of mogoltadone (**5**)... 37
Figure S32. ^{1}H-NMR spectrum (500 MHz, $CDCl_3$) of farnesiferol A (**6**)...................... 38
Figure S33. ^{13}C-NMR spectrum (125 MHz, $CDCl_3$) of farnesiferol A (**6**)...................... 39
Figure S34. ^{1}H-^{1}H COSY spectrum ($CDCl_3$) of farnesiferol A (**6**).................................. 40
Figure S35. HSQC spectrum ($CDCl_3$) of farnesiferol A (**6**).. 41
Figure S36. HMBC spectrum ($CDCl_3$) of farnesiferol A (**6**).. 42

Figure S37. NOESY spectrum ($CDCl_3$) of farnesiferol A (**6**) 43
Figure S38. ^{1}H-NMR spectrum (500 MHz, $CDCl_3$) of farnesiferol A acetate (**7**).......... 44
Figure S39. ^{13}C-NMR spectrum (125 MHz, $CDCl_3$) of farnesiferol A acetate (**7**) 45
Figure S40. ^{1}H-^{1}H COSY spectrum ($CDCl_3$) of farnesiferol A acetate (**7**)..................... 46
Figure S41. HSQC spectrum ($CDCl_3$) of farnesiferol A acetate (**7**) 47
Figure S42. HMBC spectrum ($CDCl_3$) of farnesiferol A acetate (**7**) 48
Figure S43. NOESY spectrum ($CDCl_3$) of farnesiferol A acetate (**7**)............................. 49
Figure S44. ^{1}H-NMR spectrum (500 MHz, $CDCl_3$) of gummosin (**8**) 50
Figure S45. ^{13}C-NMR (APT) spectrum (125 MHz, $CDCl_3$) of gummosin (**8**)................ 51
Figure S46. ^{1}H-^{1}H COSY spectrum ($CDCl_3$) of gummosin (**8**)....................................... 52
Figure S47. HSQC spectrum ($CDCl_3$) of gummosin (**8**).. 53
Figure S48. HMBC spectrum ($CDCl_3$) of gummosin (**8**)... 54
Figure S49. NOESY spectrum ($CDCl_3$) of gummosin (**8**) 55
Figure S50. ^{1}H-NMR spectrum (500 MHz, CDCl3) of ferukrin (**9**) 56
Figure S51. ^{13}C-NMR spectrum (125 MHz, $CDCl_3$) of ferukrin (**9**) 57
Figure S52. ^{1}H-^{1}H COSY spectrum ($CDCl_3$) of ferukrin (**9**).. 58
Figure S53. HSQC spectrum ($CDCl_3$) of ferukrin (**9**) 59
Figure S54. HMBC spectrum ($CDCl_3$) of ferukrin (**9**) 60
Figure S55. NOESY spectrum ($CDCl_3$) of ferukrin (**9**).. 61
Figure S56. ^{1}H-NMR spectrum (500 MHz, $CDCl_3$) of ferukrin acetate (**10**).................. 62
Figure S57. ^{13}C-NMR spectrum (125 MHz, $CDCl_3$) of ferukrin acetate (**10**)................ 63
Figure S58. ^{1}H-^{1}H COSY spectrum ($CDCl_3$) of ferukrin acetate (**10**) 64
Figure S59. HSQC spectrum ($CDCl_3$) of ferukrin acetate (**10**)....................................... 65
Figure S60. HMBC spectrum ($CDCl_3$) of ferukrin acetate (**10**)...................................... 66
Figure S61. NOESY spectrum ($CDCl_3$) of ferukrin acetate (**10**)..................................... 67
Figure S62. ^{1}H-NMR spectrum (500 MHz, $CDCl_3$) of deacetylkellerin (**11**) 68
Figure S63. ^{13}C-NMR (APT) spectrum (125 MHz, $CDCl_3$) of deacetylkellerin (**11**)..... 69
Figure S64. ^{1}H-^{1}H COSY spectrum ($CDCl_3$) of deacetylkellerin (**11**)............................ 70
Figure S65. HSQC spectrum ($CDCl_3$) of deacetylkellerin (**11**)....................................... 71
Figure S66. HMBC spectrum ($CDCl_3$) of deacetylkellerin (**11**)...................................... 72
Figure S67. NOESY spectrum ($CDCl_3$) of deacetylkellerin (**11**) 73
Figure S68. ^{1}H-NMR spectrum (500 MHz, $CDCl_3$) of kellerin (**12**) 74
Figure S69. ^{13}C-NMR (APT) spectrum (125 MHz, $CDCl_3$) of kellerin (**12**)................... 75
Figure S70. ^{1}H-^{1}H COSY spectrum ($CDCl_3$) of kellerin (**12**)... 76
Figure S71. HSQC spectrum ($CDCl_3$) of kellerin (**12**) 77
Figure S72. HMBC spectrum ($CDCl_3$) of kellerin (**12**) 78
Figure S73. NOESY spectrum ($CDCl_3$) of kellerin (**12**).. 79
Figure S74. ^{1}H-NMR spectrum (500 MHz, $CDCl_3$) of samarcandone (**13**).................... 80
Figure S75. ^{13}C-NMR (APT) spectrum (125 MHz, $CDCl_3$) of samarcandone (**13**) 81

Figure S76. ^{1}H-^{1}H COSY spectrum ($CDCl_3$) of samarcandone (**13**) 82
Figure S77. HSQC spectrum ($CDCl_3$) of samarcandone (**13**) 83
Figure S78. HMBC spectrum ($CDCl_3$) of samarcandone (**13**) 84
Figure S79. NOESY spectrum ($CDCl_3$) of samarcandone (**13**) 85
Figure S80. ^{1}H-NMR spectrum (500 MHz, $CDCl_3$) of samarcandin (**14**) 86
Figure S81. ^{13}C-NMR spectrum (125 MHz, $CDCl_3$) of samarcandin (**14**) 87
Figure S82. ^{1}H-^{1}H COSY spectrum ($CDCl_3$) of samarcandin (**14**) 88
Figure S83. HSQC spectrum ($CDCl_3$) of samarcandin (**14**) 89
Figure S84. HMBC spectrum ($CDCl_3$) of samarcandin (**14**) 90
Figure S85. NOESY spectrum ($CDCl_3$) of samarcandin (**14**) 91
Figure S86. ^{1}H-NMR spectrum (500 MHz, $CDCl_3$) of samarcandin acetate (**15**) 92
Figure S87. ^{13}C-NMR (APT) spectrum (125 MHz, $CDCl_3$) of samarcandin acetate (**15**) 93
Figure S88. ^{1}H-^{1}H COSY spectrum ($CDCl_3$) of samarcandin acetate (**15**) 94
Figure S89. HSQC spectrum ($CDCl_3$) of samarcandin acetate (**15**) 95
Figure S90. HMBC spectrum ($CDCl_3$) of samarcandin acetate (**15**) 96
Figure S91. NOESY spectrum ($CDCl_3$) of samarcandin acetate (**15**) 97
Figure S92. Chemical transformations of the cytotoxic sesquiterpene coumarins of *Ferula huber-morathii* 98
Figure S93. Comparison of ^{1}H-NMR spectra of isosamarcandin (**16**) and ferukrin (**9**) 99
Figure S94. Previously proposed structures of sesquiterpene compounds isolated from the chloroform extract of *Ferula huber-morathii* [3] 100
Figure S95. TLC comparison of the dichloromethane extracts of *Ferula huber-morathii* with reference compounds; elaeochytrin A (**18**) and ferutinin (**17**), and isolated sesquiterpene coumarins (**1-15**), mobile phase; Hxn:EtOAc (6:4), detection; **A**: 254 nm; **B**: 366 nm; **C**: Anisaldehyde reagent 101
Figure S96. A. HPLC chromatogram of **t**he dichloromethane root extract of *Ferula huber-morathii* spiked with reference compounds ferutinin (**17**) and elaeochytrin A (**18**), **B.** HPLC chromatogram of the dichloromethane root extract of *F. huber-morathii* and **C.** HPLC chromatogram of the mixture of reference compounds ferutinin (**17**) and elaeochytrin A (**18**). Detection: UV detector at 259 nm 102
Figure S97. ^{1}H-NMR spectrum (500 MHz, $CDCl_3$) of reference compound ferutinin (**17**) 105
Figure S98. ^{1}H-NMR spectrum (500 MHz, $CDCl_3$) of reference compound elaeochtyrin A (**18**) 107
Figure S99. Preparation of the (*R*)-MTPA ester of samarcandin (**24**) and structure of *ent*-samarcandin (**23**) 108

Figure S100. ^{1}H-NMR spectrum (500 MHz, $CDCl_3$) of (*R*)-MTPA ester of samarcandin (**24**) ... 110
Figure S101. ^{13}C-NMR spectrum (125 MHz, $CDCl_3$) of (*R*)-MTPA ester of samarcandin (**24**) ... 112
Figure S102. ^{1}H-^{1}H COSY spectrum ($CDCl_3$) of (*R*)-MTPA ester of samarcandin (**24**) ... 113
Figure S103. HSQC spectrum of (*R*)-MTPA ester of samarcandin (**24**) ... 114
Figure S104. HMBC spectrum of (*R*)-MTPA ester of samarcandin (**24**) ... 115
Figure S105. NOESY spectrum of (*R*)-MTPA ester of samarcandin (**24**) ... 116
Figure S106. (+)-HRESIMS spectrum of (*R*)-MTPA ester of samarcandin (**24**) ... 117
Figure S107. IR spectrum of (*R*)-MTPA ester of samarcandin (**24**) ... 118
Figure S108. UV spectrum of (*R*)-MTPA ester of samarcandin (**24**) ... 119
Figure S109. Packings of the (*R*)-MTPA ester of samarcandin (**24**) in unit cell ... 120
Figure S110. Structures of Bcl-XL and β-Catenin ligands ... 122
Figure S111. Validation of the docking models. Superposition of docked pose and experimental binding conformation (green) of Native Ligans in the binding pocket of 7AFW (left), 7LH7 (right) ... 123

Table S1. Physical appearance and comparison of optical rotation values of isolated terpenoid coumarins with literature data ... 7
Table S2. Crystal data and details of the structure determination of (*R*)-MTPA ester of samarcandin (**24**) ... 121

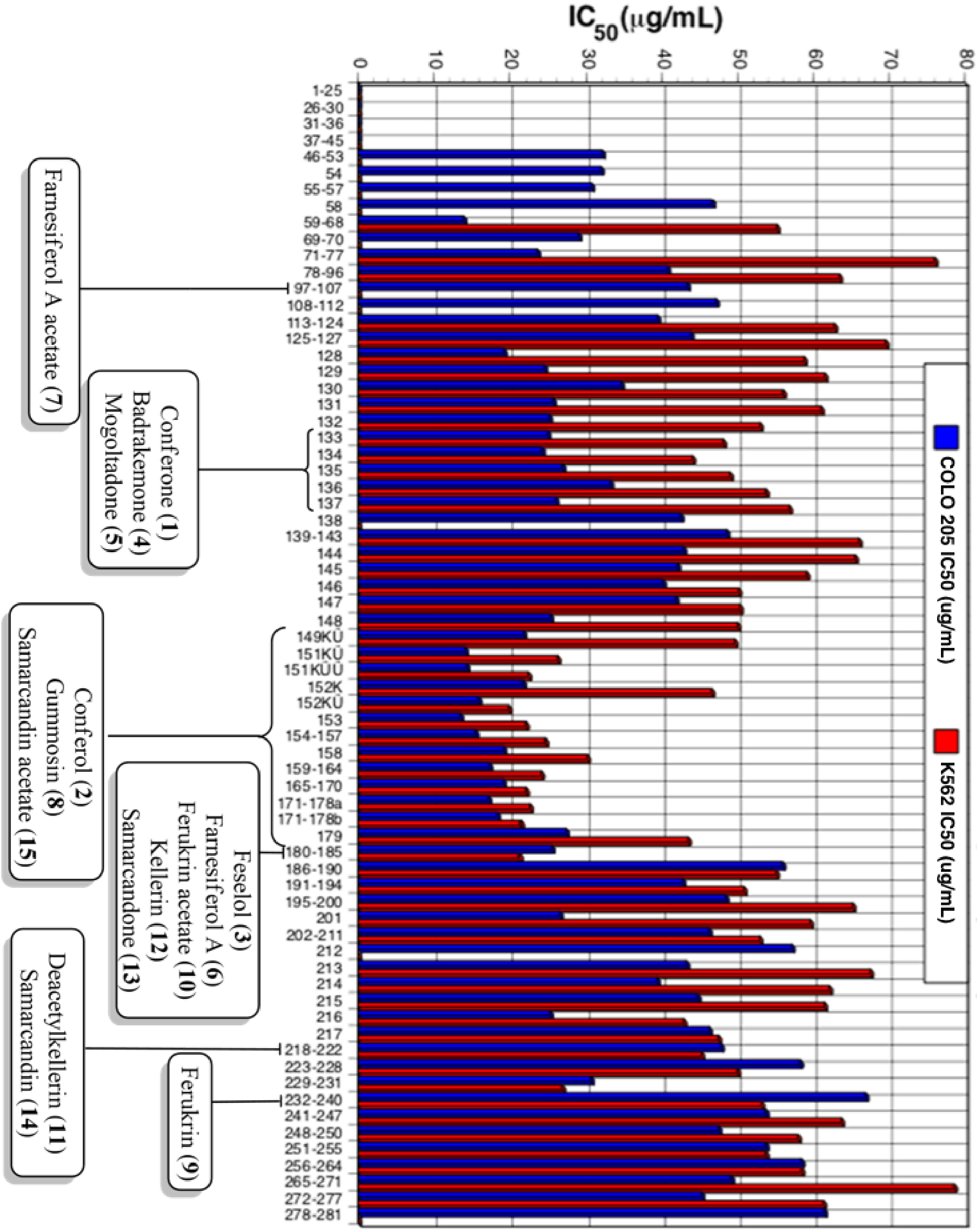

Figure S1. Bioactivity-guided isolation scheme for the cytotoxic sesquiterpene coumarins of the dichloromethane root extract of *Ferula huber-morathii*

Table S1. Physical appearance and comparison of optical rotation values of isolated terpenoid coumarins with literature data

Compound	Physical appearance	Isolated Comp.	Lit. Reference	
		$[a]_D^{temp.}$ (Solv.)	$[a]_D^{temp.}$ (Solv.)	Ref.
Conferone (**1**)	White powder	-55.0°[24] (CH_2Cl_2)	-51.0°[20] (EtOH)	[1]
Conferol (**2**)	White powder	-55.1°[24] (CH_2Cl_2)	-84.2°[20] ($CHCl_3$)	[1]
Feselol (**3**)	Gum	-60.9°[24] (CH_2Cl_2)	-98.5°[20] (EtOH)	[1]
Badrekemone (**4**)	White powder	-39.7°[24] (CH_2Cl_2)	-42.0°[22] ($CHCl_3$)	[1]
Mogoltadone (**5**)	White powder	-19.4°[25] (CH_2Cl_2)	-41.7°[21] ($CHCl_3$)	[1]
Farnesiferol A (**6**)	Gum	-50.1°[24] (CH_2Cl_2)	-55.0°[25] ($CHCl_3$)	[1]
Farnesiferol A acetate (**7**)	Gum	-27.3°[24] (CH_2Cl_2)	-55.0°[22] ($CHCl_3$)	[2]
Gummosin (**8**)	Gum	-37.0°[24] (CH_2Cl_2)	-54.0°[25] ($CHCl_3$)	[1]
Ferukrin (**9**)	White powder	+25.3°[24] (CH_2Cl_2)	+30.0°[22] (EtOH)	[1]
Ferukrin acetate (**10**)	White powder	+7.6°[24] (CH_2Cl_2)	+20.0°[22] (EtOH)	[1]
Deacetylkellerin (**11**)	White powder	+25.3°[24] (CH_2Cl_2)	+52.0°[22] (EtOH)	[1]
Kellerin (**12**)	White powder	+63.6°[24] (CH_2Cl_2)	+66.4°[20] (EtOH)	[1]
Samarcandone (**13**)	White powder	+10.0°[24] (CH_2Cl_2)	+25.0°[25] (EtOH)	[1]
Samarcandin (**14**)	Gum	+6.8°[24] (CH_2Cl_2)	+30.0°[25] (EtOH)	[1]
Samarcandin acetate (**15**)	Gum	-4.3°[24] (CH_2Cl_2)<br>+23.9°[23] (EtOH)	+29.4°[25] (EtOH)	[1]

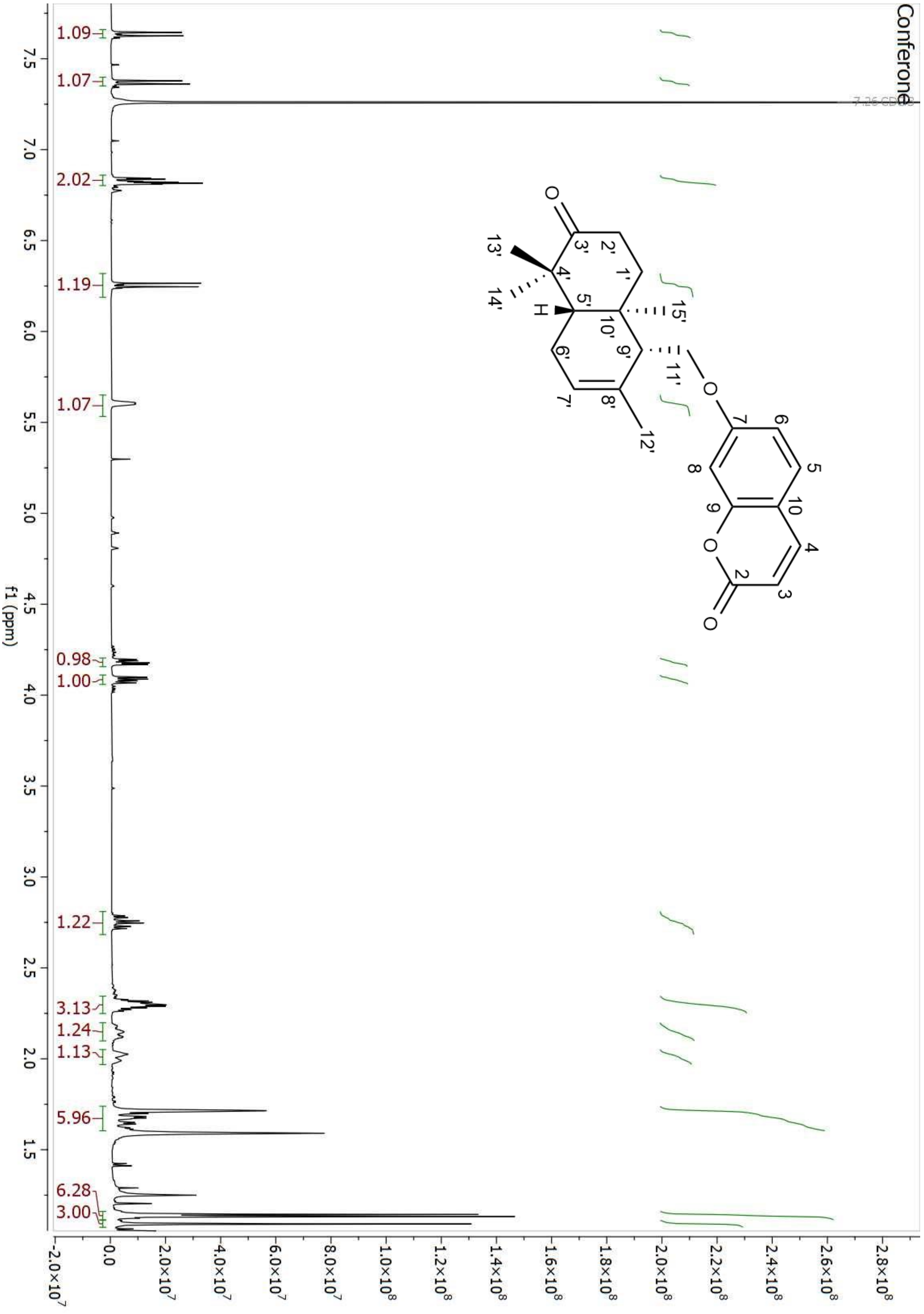

Figure S2. ^{1}H-NMR spectrum (500 MHz, $CDCl_3$) of conferone (**1**)

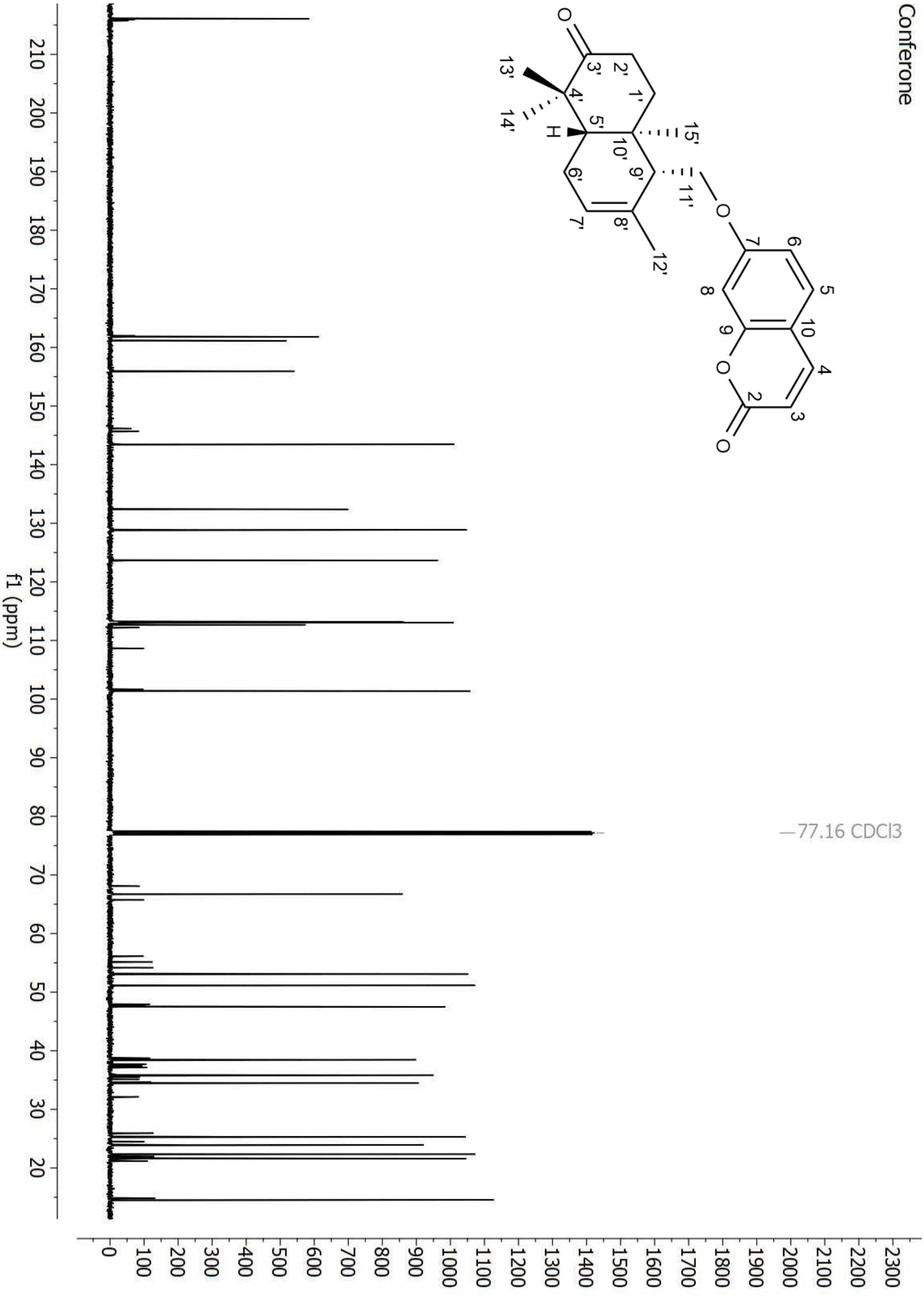

Figure S3. ^{13}C-NMR spectrum (125 MHz, $CDCl_3$) of conferone (**1**)

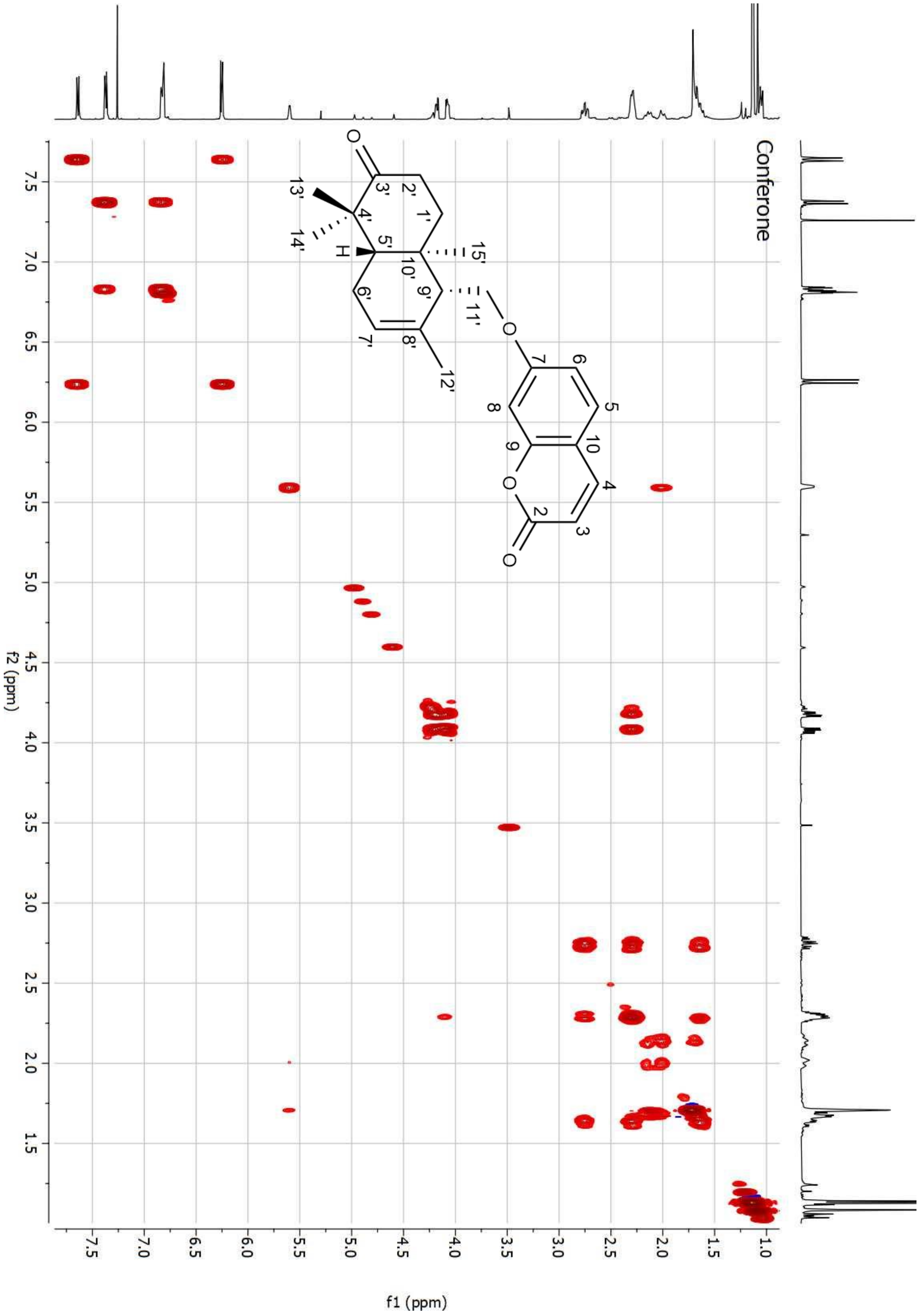

Figure S4. ^{1}H-^{1}H COSY spectrum ($CDCl_3$) of conferone (**1**)

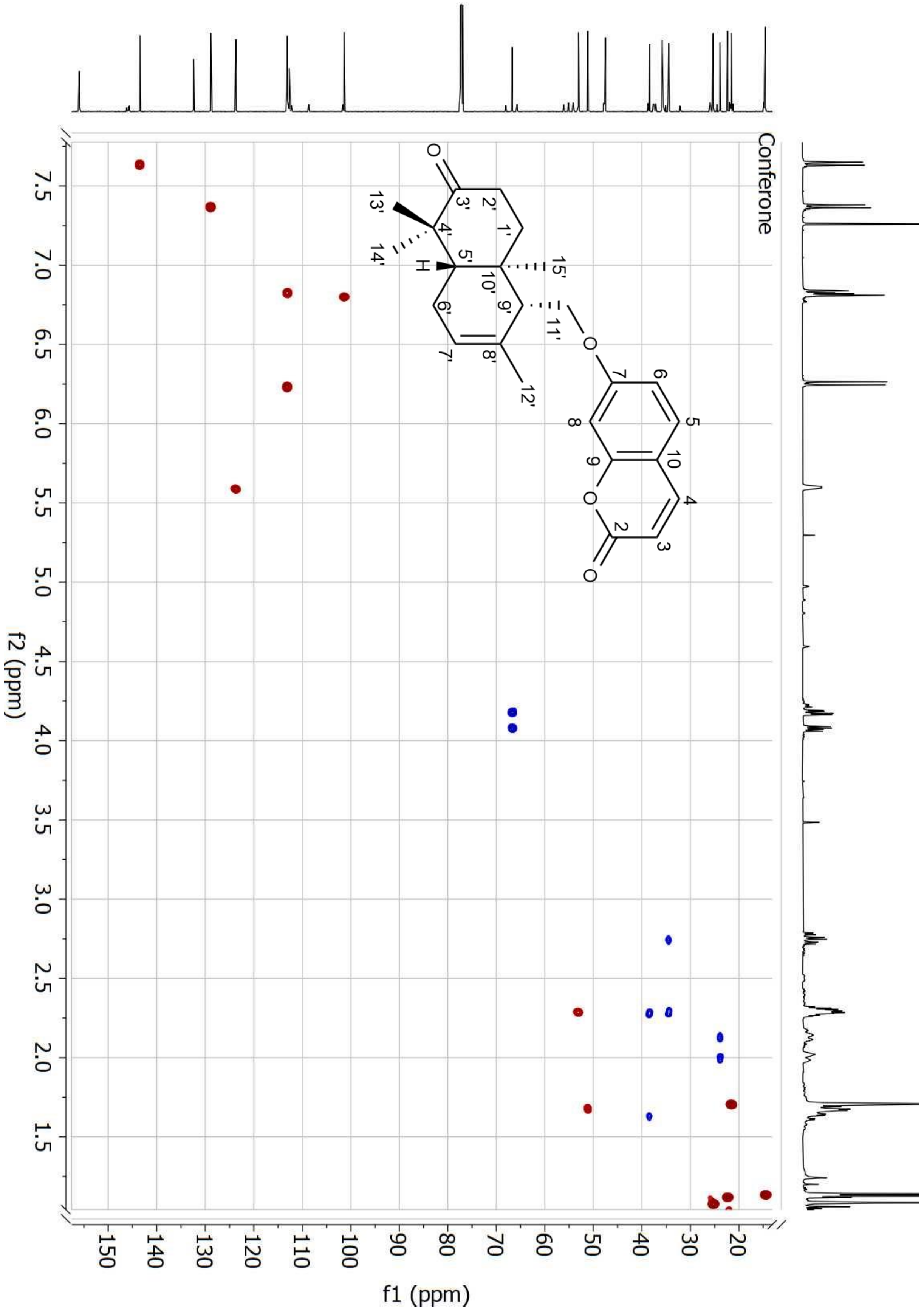

Figure S5. HSQC spectrum ($CDCl_3$) of conferone (**1**)

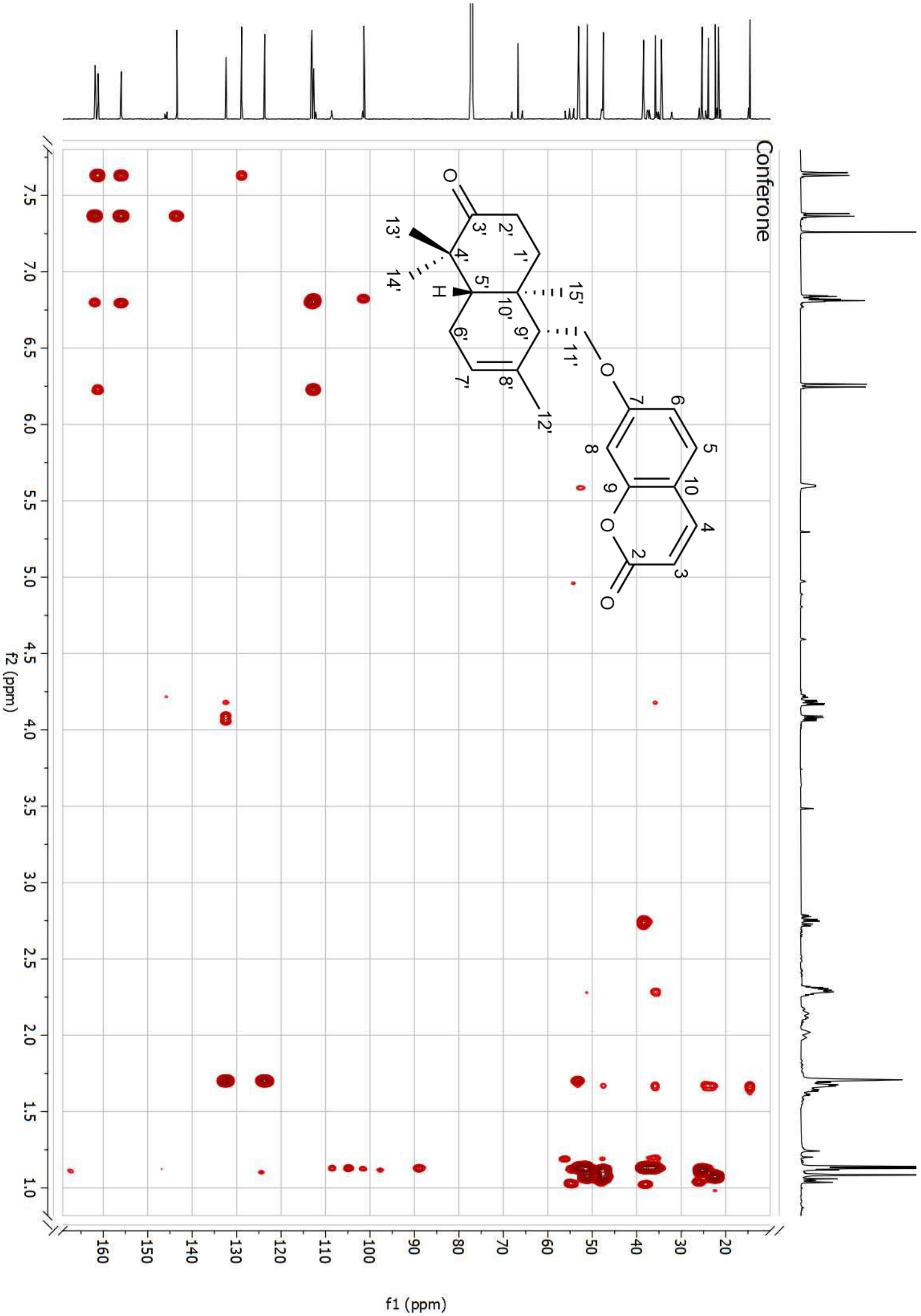

Figure S6. HMBC spectrum (CDCl3) of conferone (**1**)

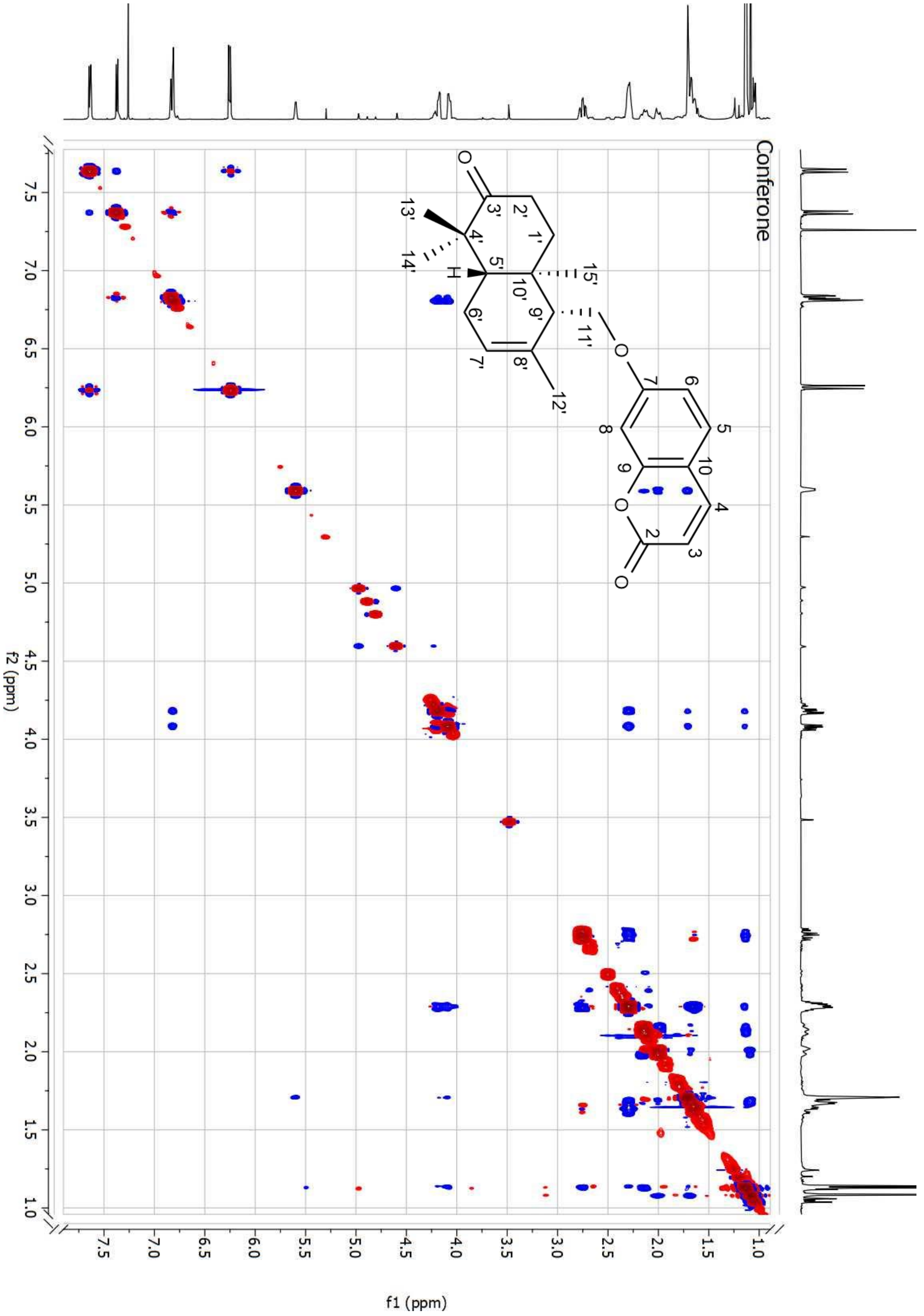

Figure S7. NOESY spectrum ($CDCl_3$) of conferone (**1**)

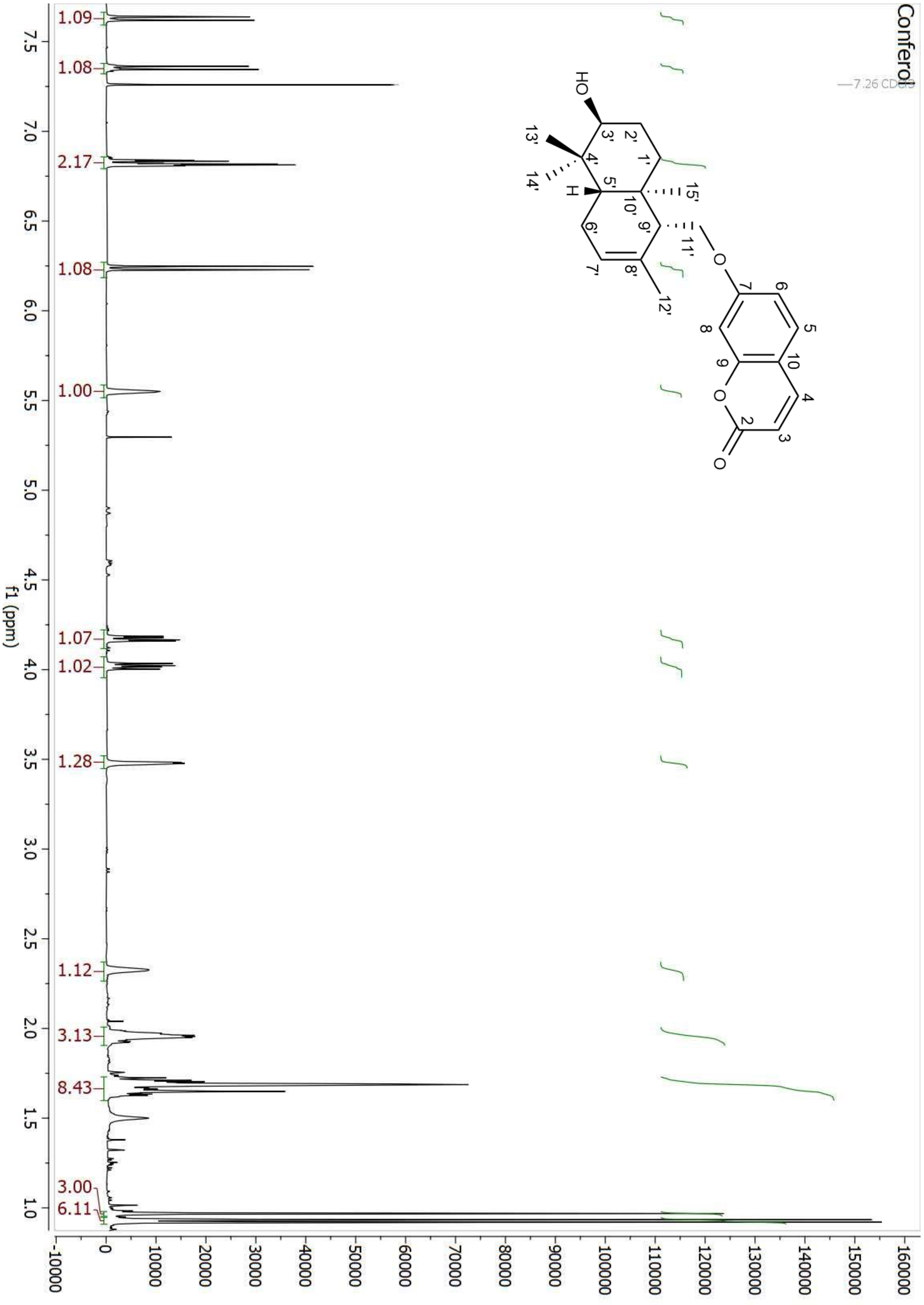

Figure S8. ^{1}H-NMR spectrum (500 MHz, $CDCl_3$) of conferol (**2**)

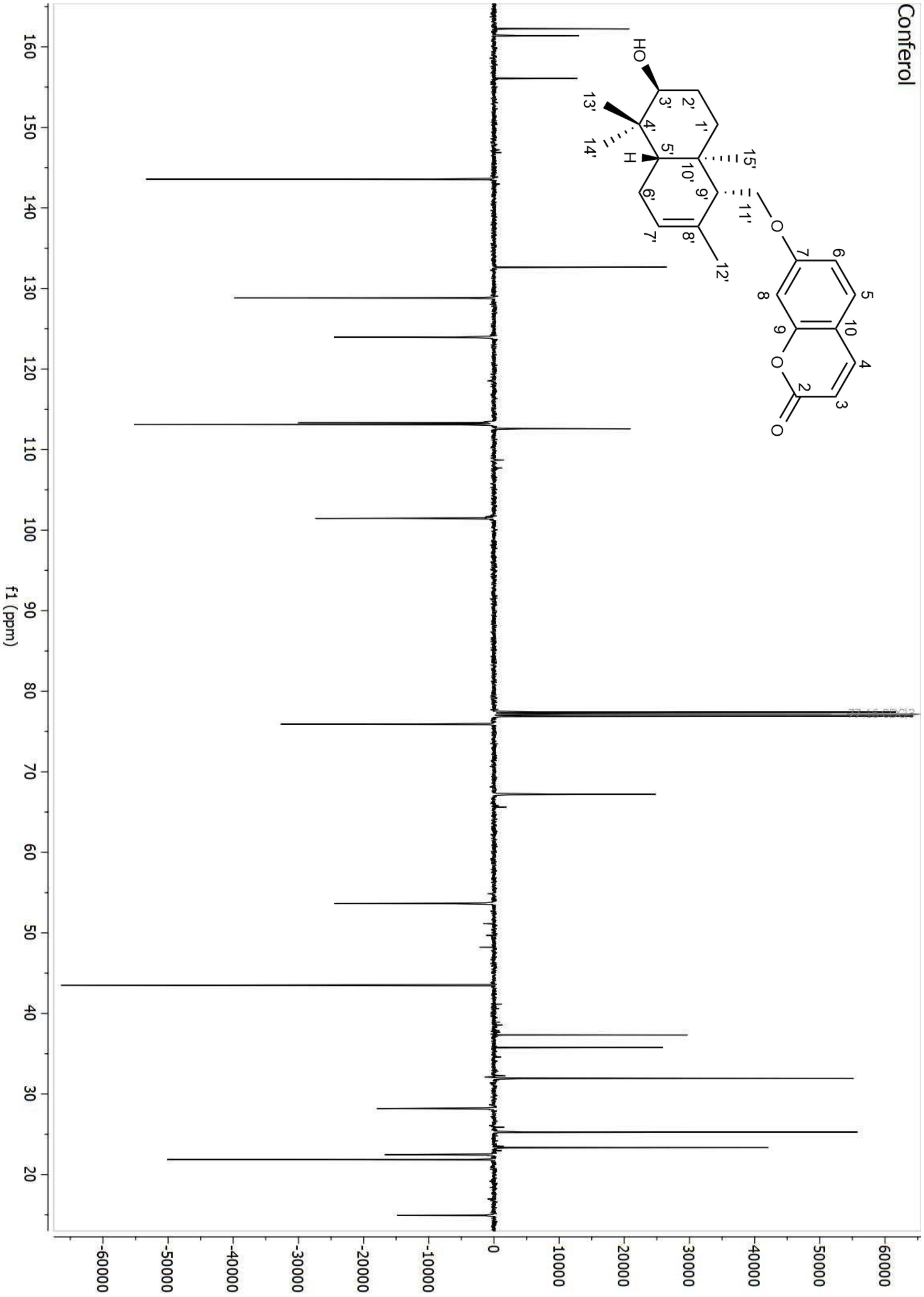

Figure S9. ^{13}C-NMR (APT) spectrum (125 MHz, $CDCl_3$) of conferol (**2**)

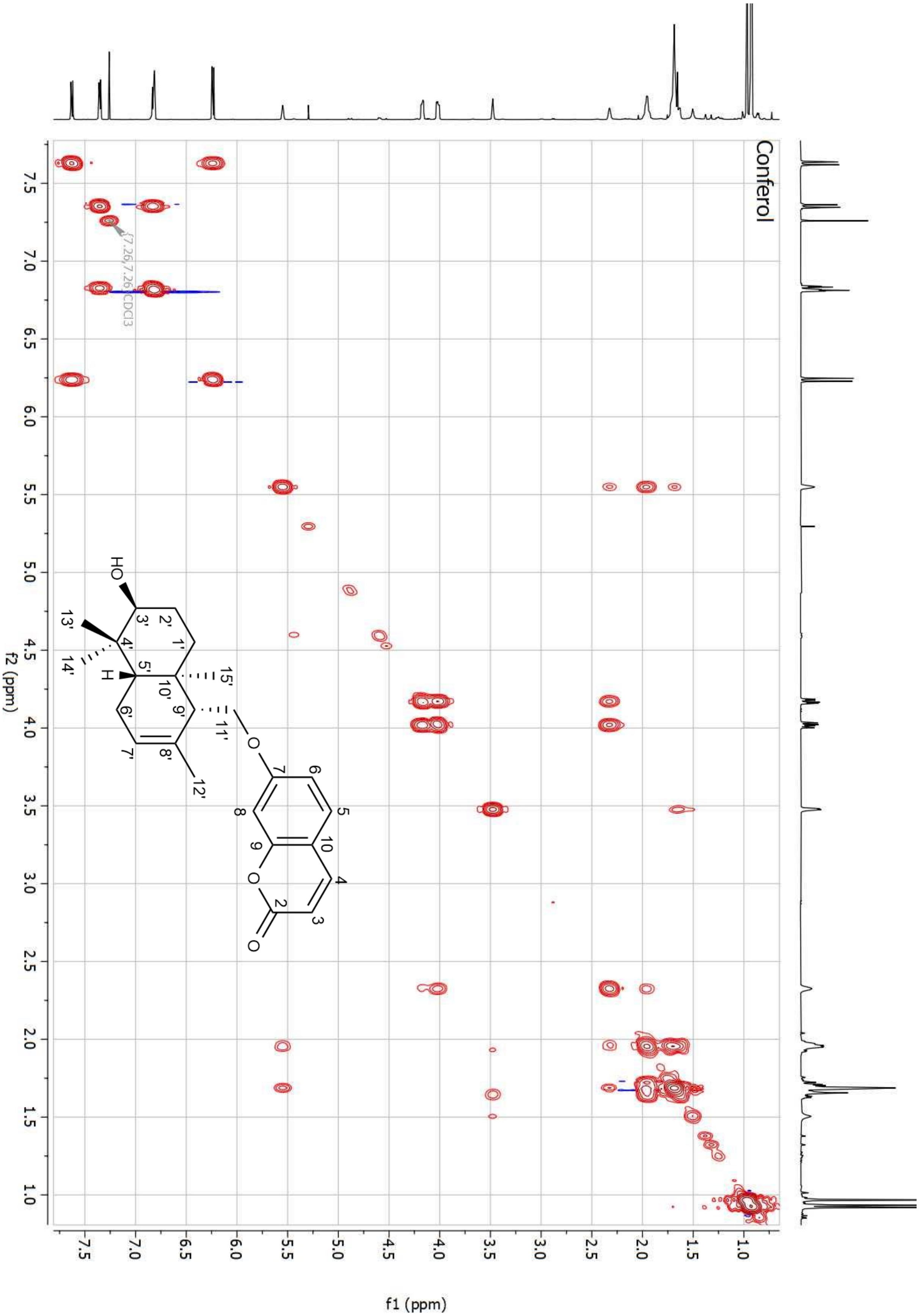

Figure S10. ^1H-^1H COSY spectrum ($CDCl_3$) of conferol (**2**)

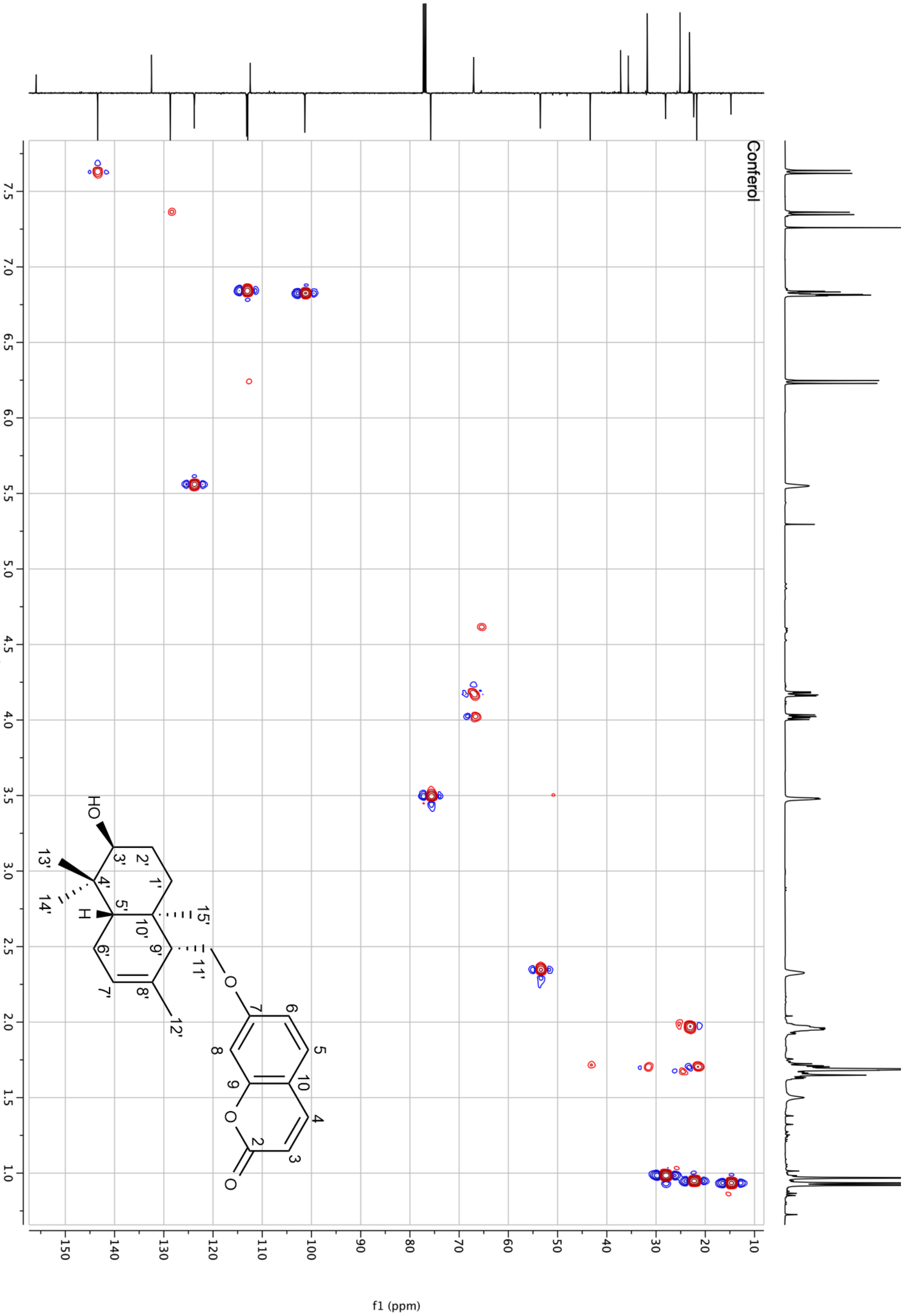

Figure S11. HSQC spectrum ($CDCl_3$) of conferol (**2**)

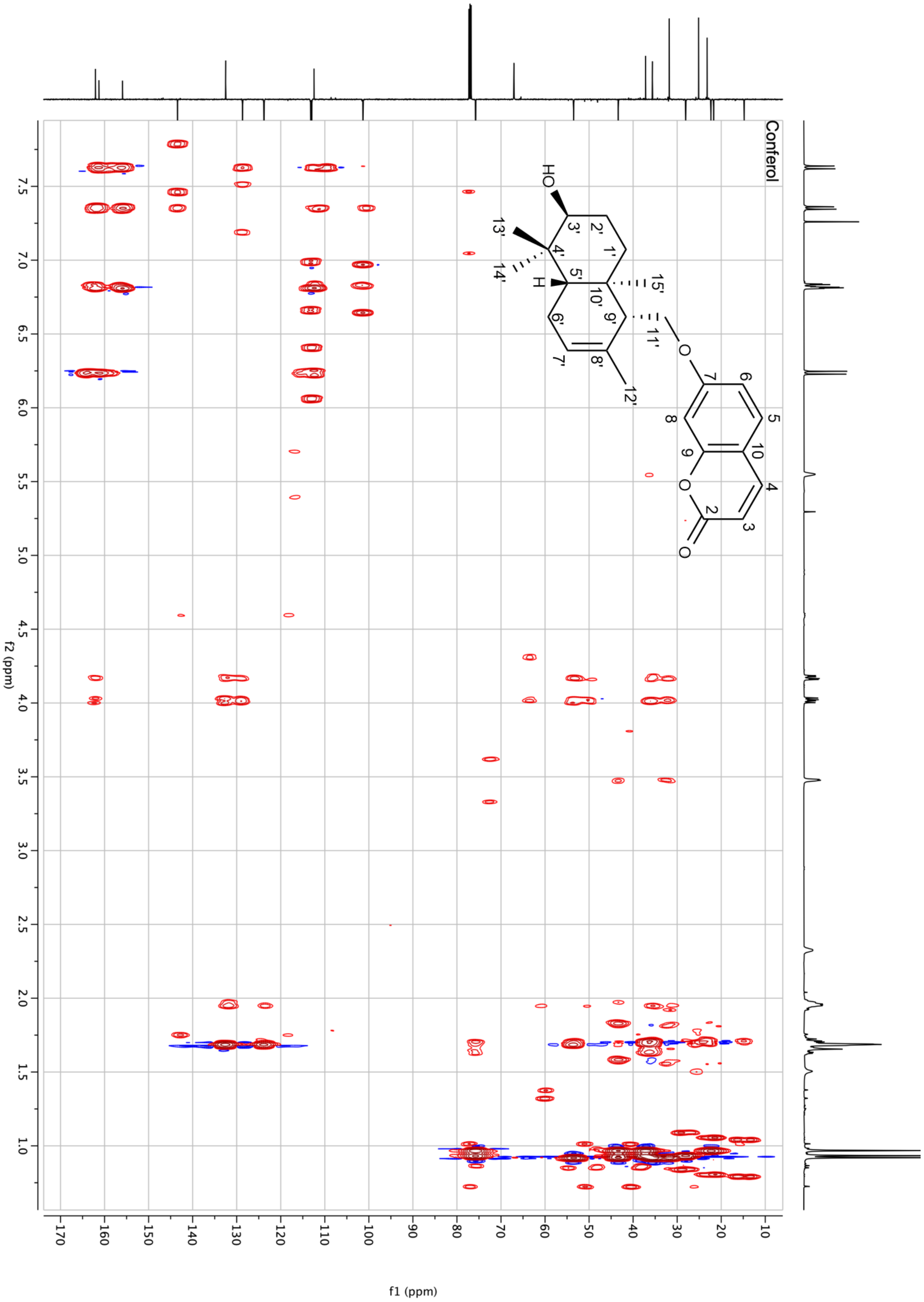

Figure S12. HMBC spectrum ($CDCl_3$) of conferol (**2**)

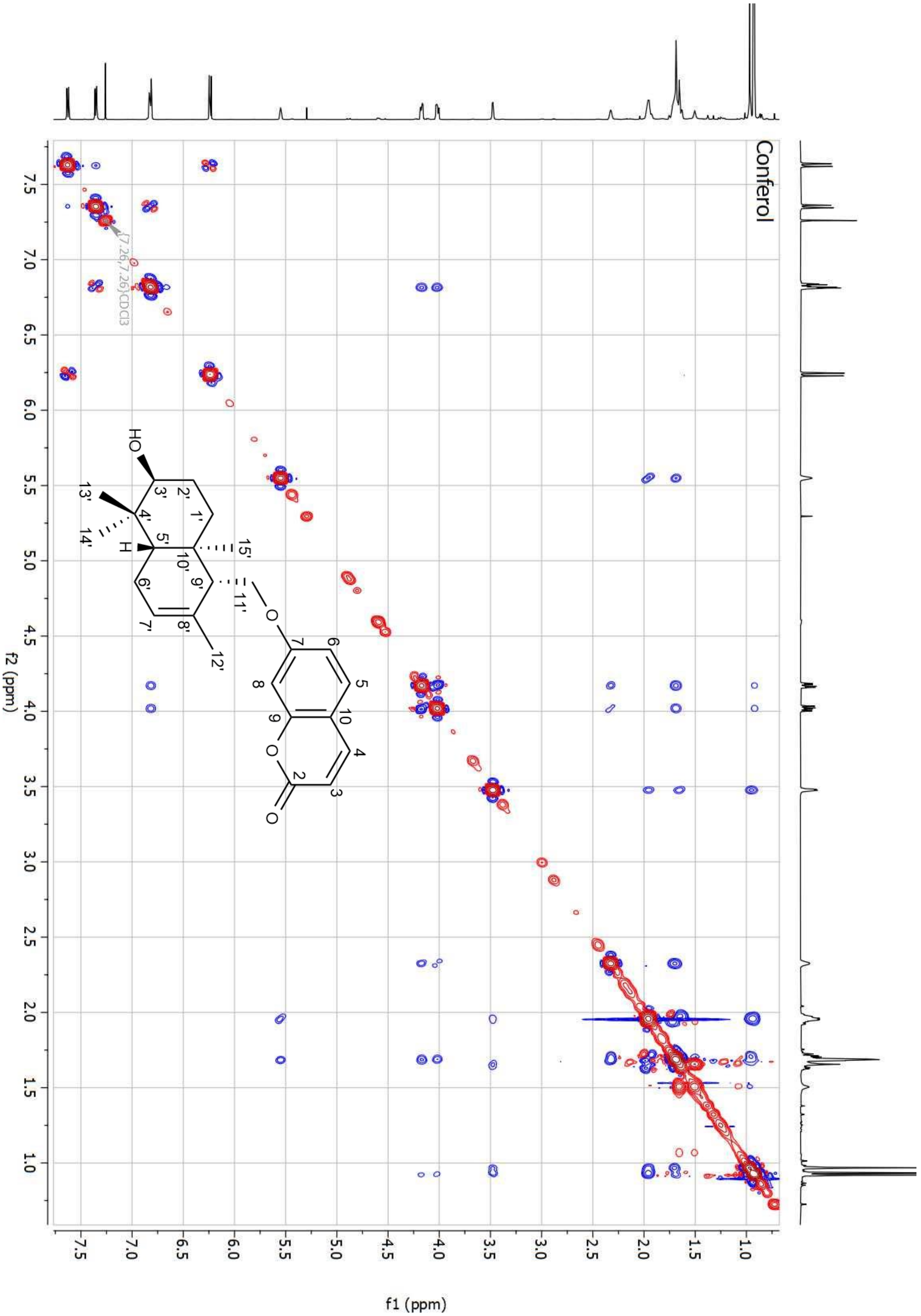

Figure S13. NOESY spectrum ($CDCl_3$) of conferol (**2**)

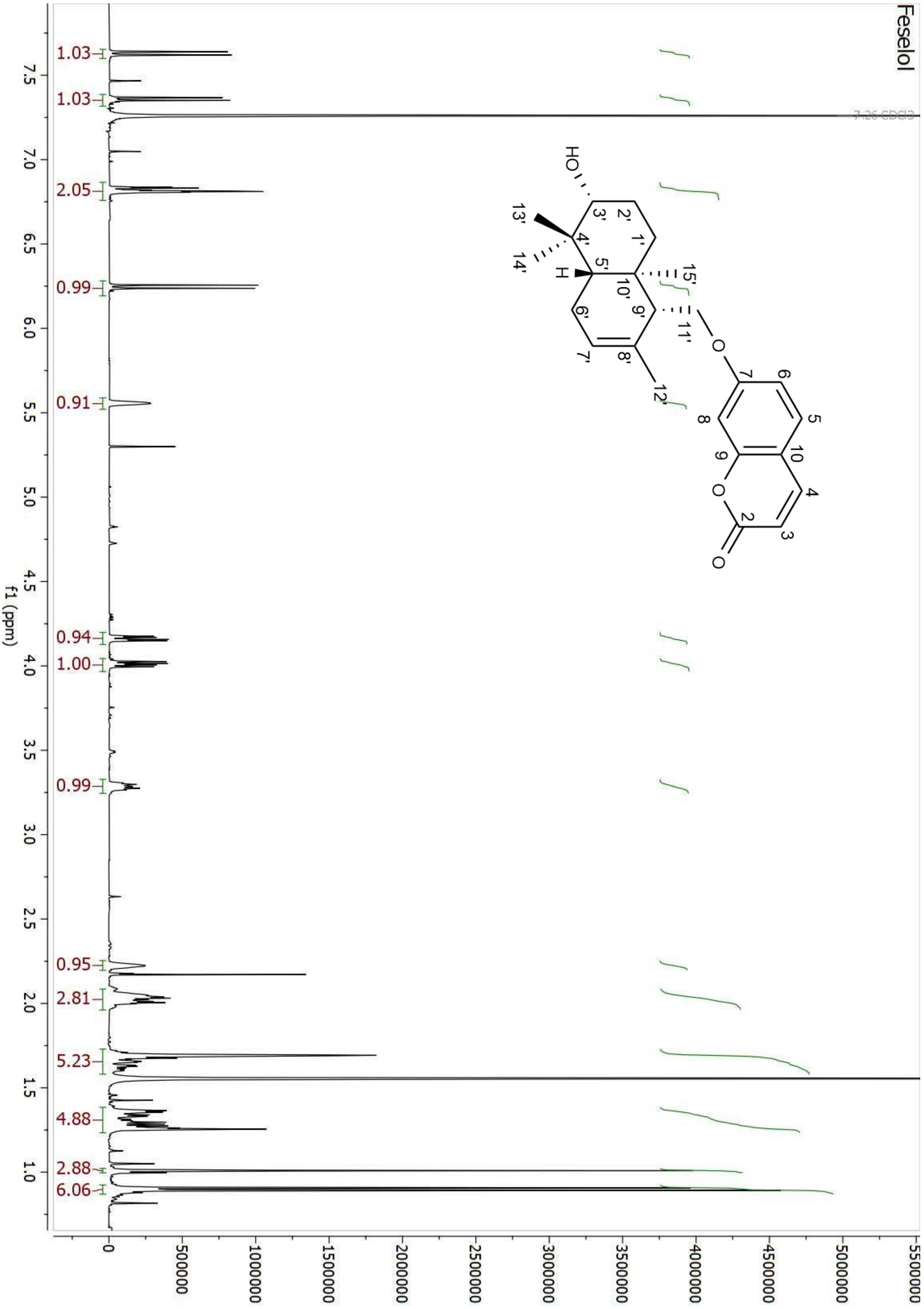

Figure S14. ^{1}H-NMR spectrum (500 MHz, $CDCl_3$) of feselol (**3**)

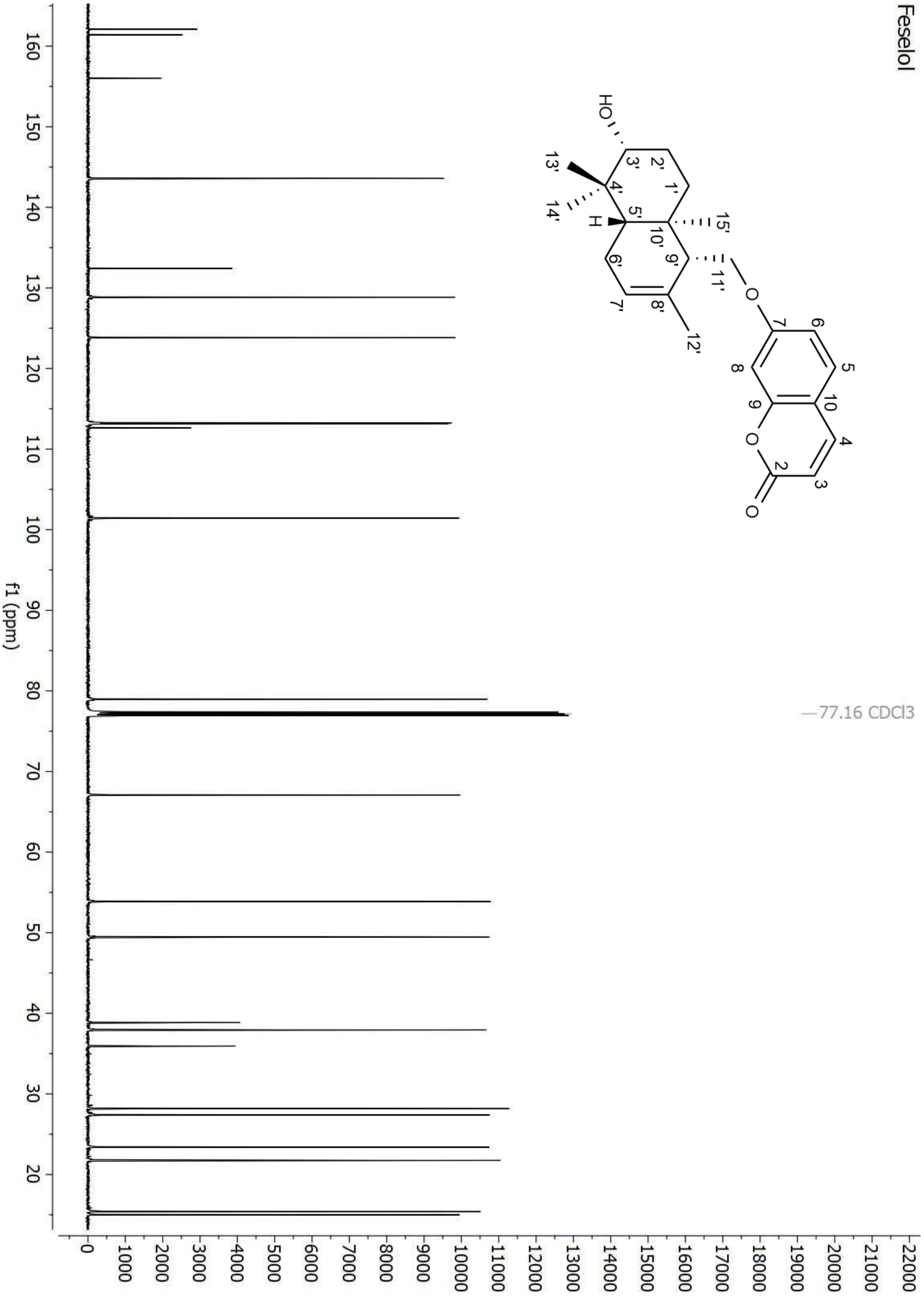

Figure S15. ^{13}C-NMR spectrum (125 MHz, $CDCl_3$) of feselol (**3**)

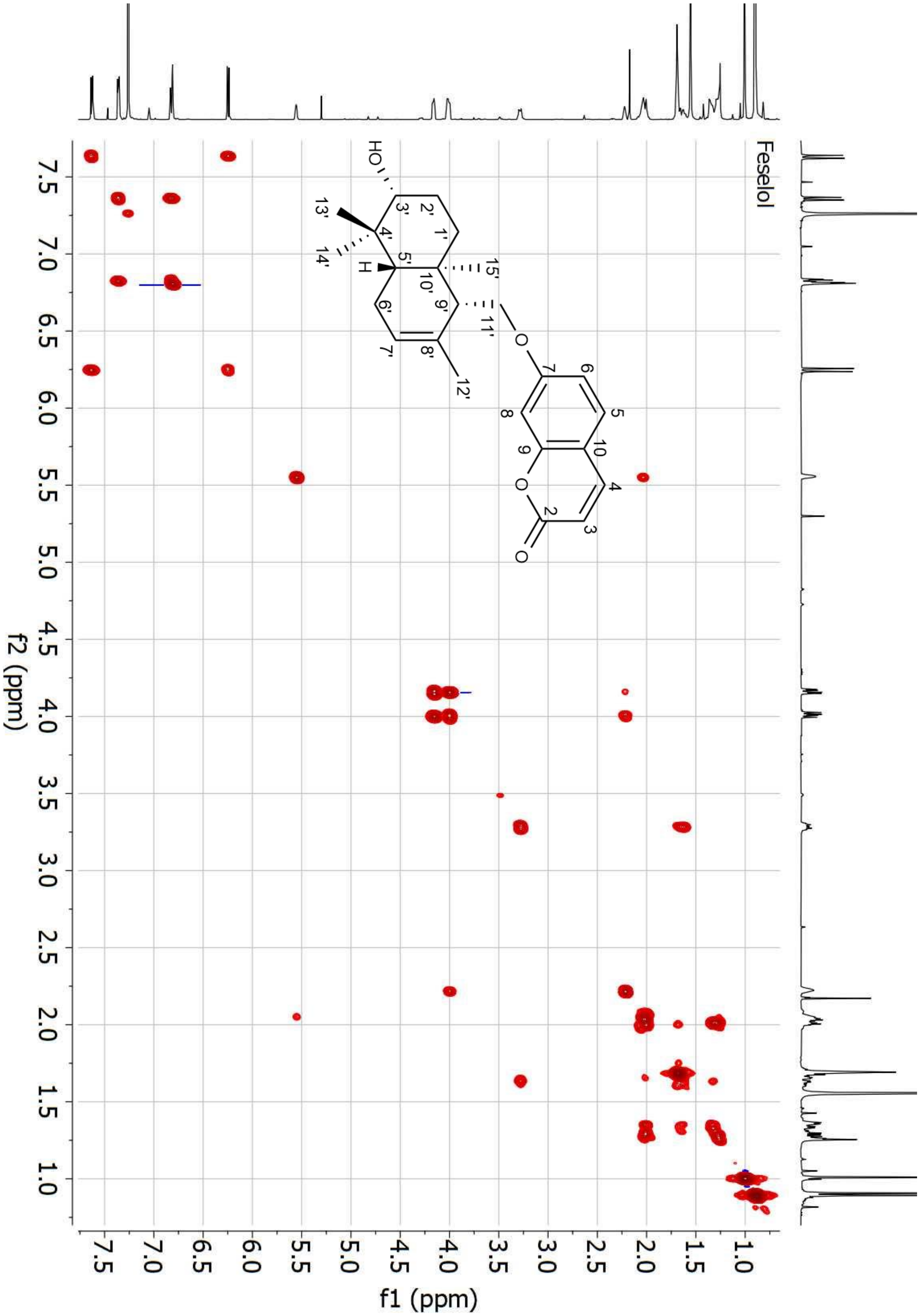

Figure S16. ^{1}H-^{1}H COSY spectrum ($CDCl_3$) of feselol (**3**)

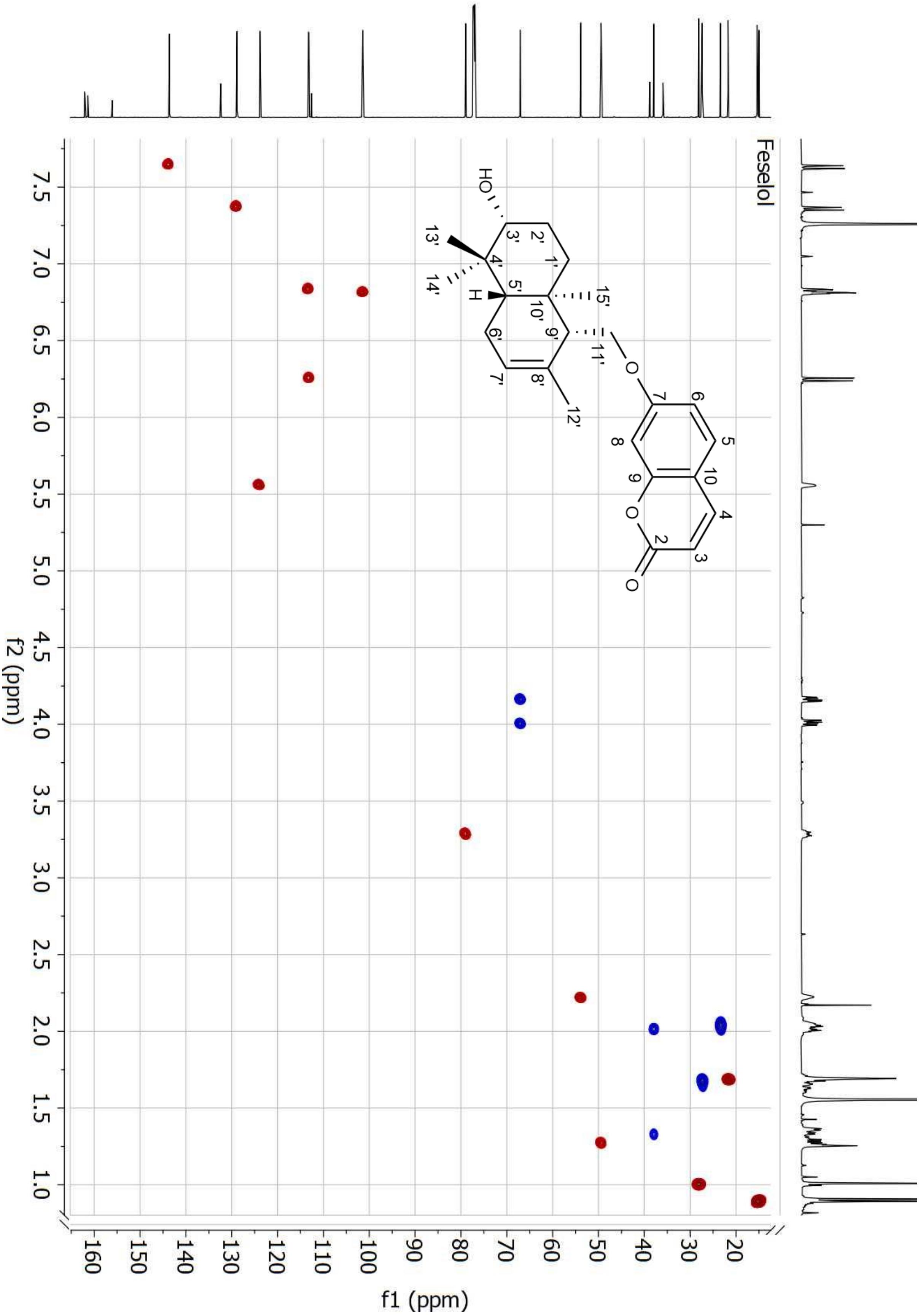

Figure S17. HSQC spectrum ($CDCl_3$) of feselol (**3**)

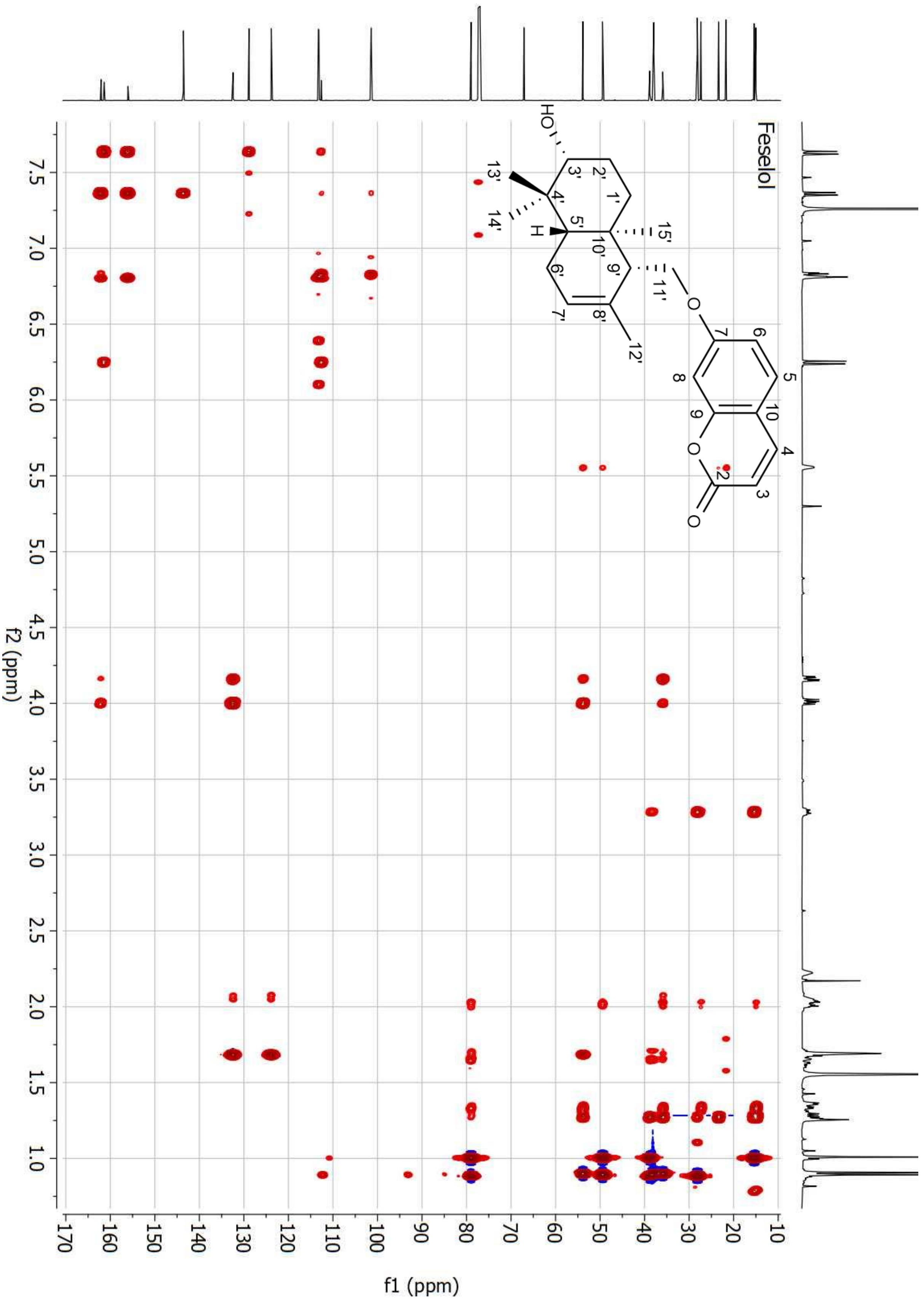

Figure S18. HMBC spectrum ($CDCl_3$) of feselol (**3**)

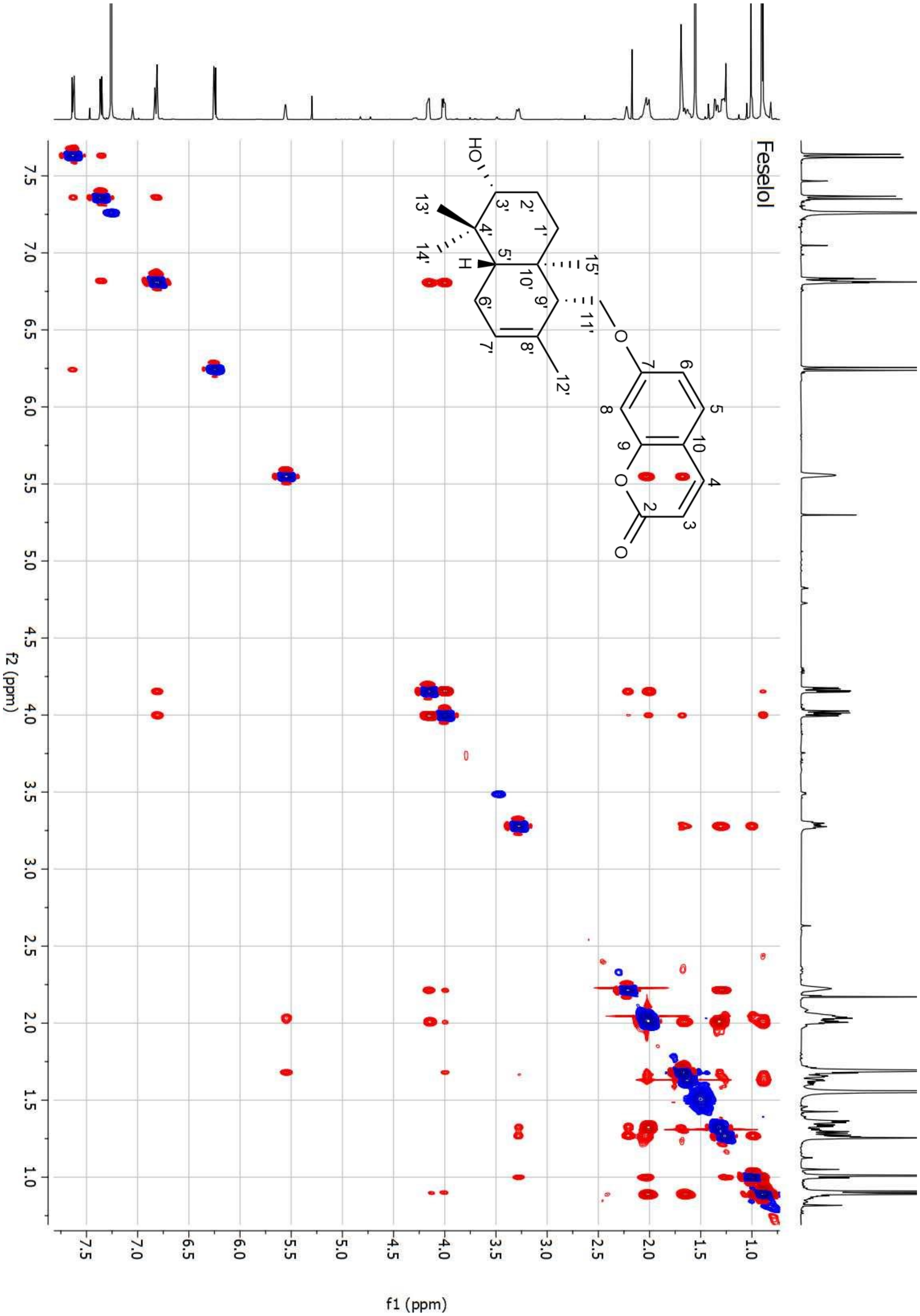

Figure S19. NOESY spectrum ($CDCl_3$) of feselol (**3**)

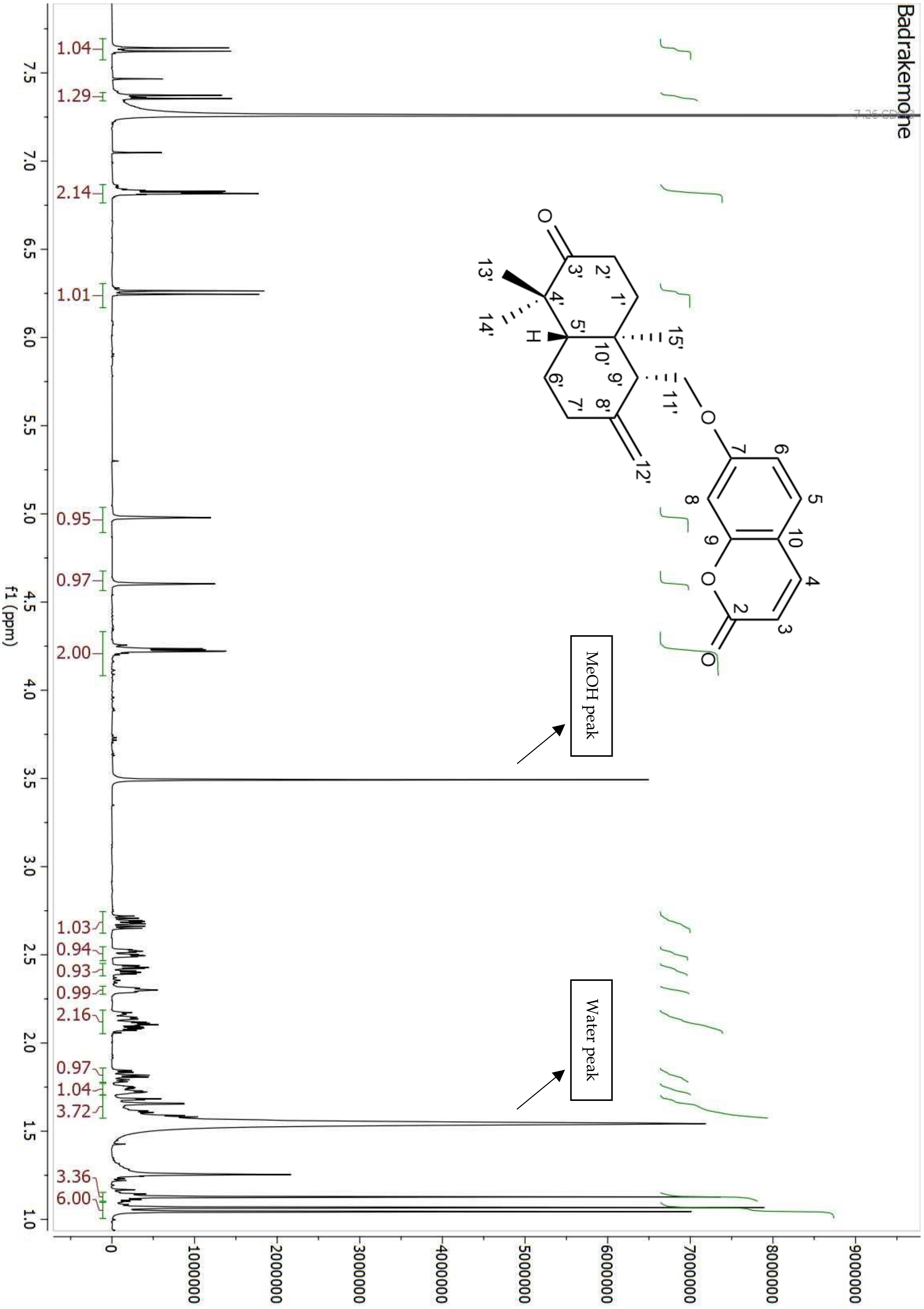

Figure S20. ^{1}H-NMR spectrum (500 MHz, $CDCl_3$) of badrakemone (**4**)

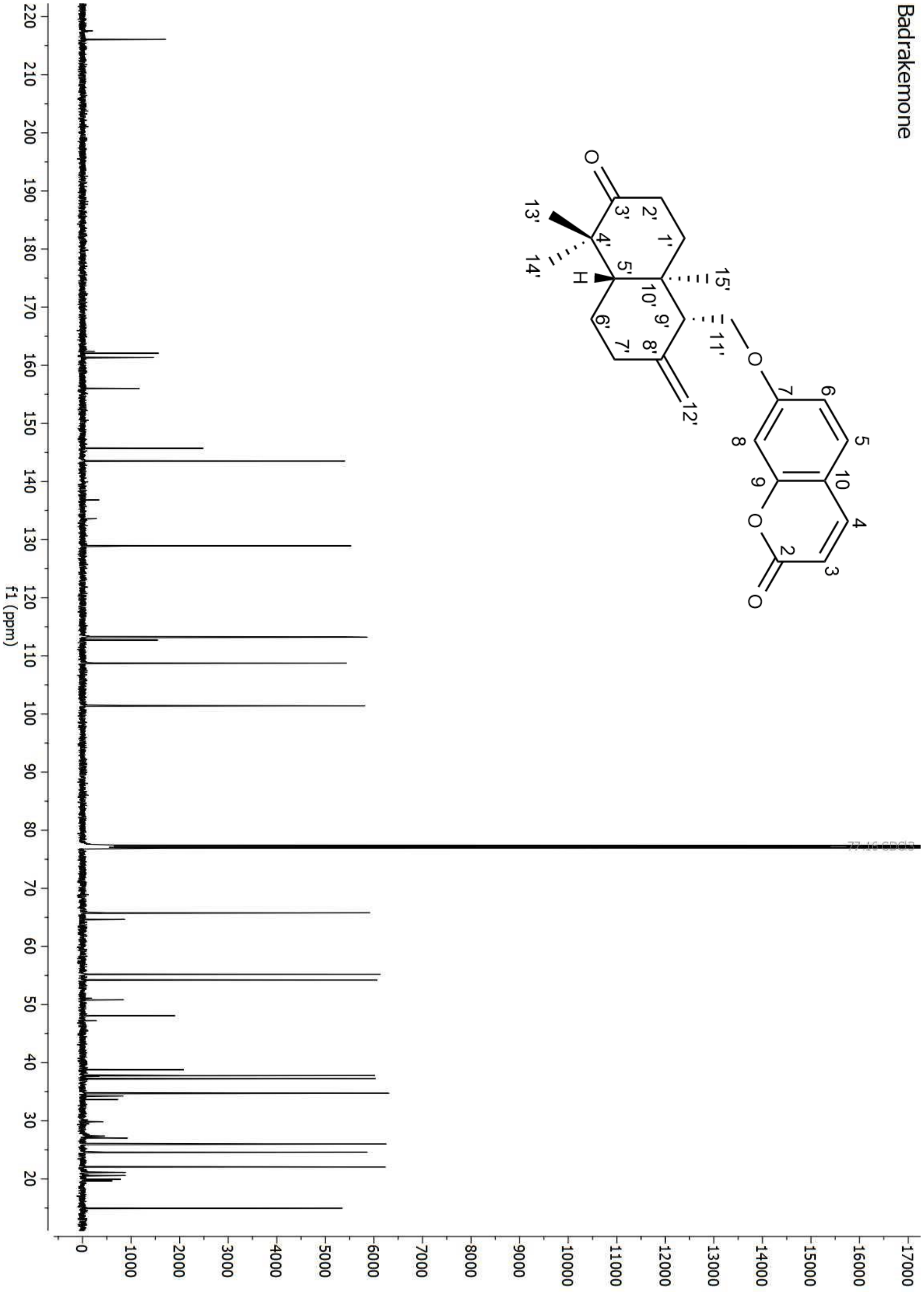

Figure S21. ^{13}C-NMR spectrum (125 MHz, $CDCl_3$) of badrakemone (**4**)

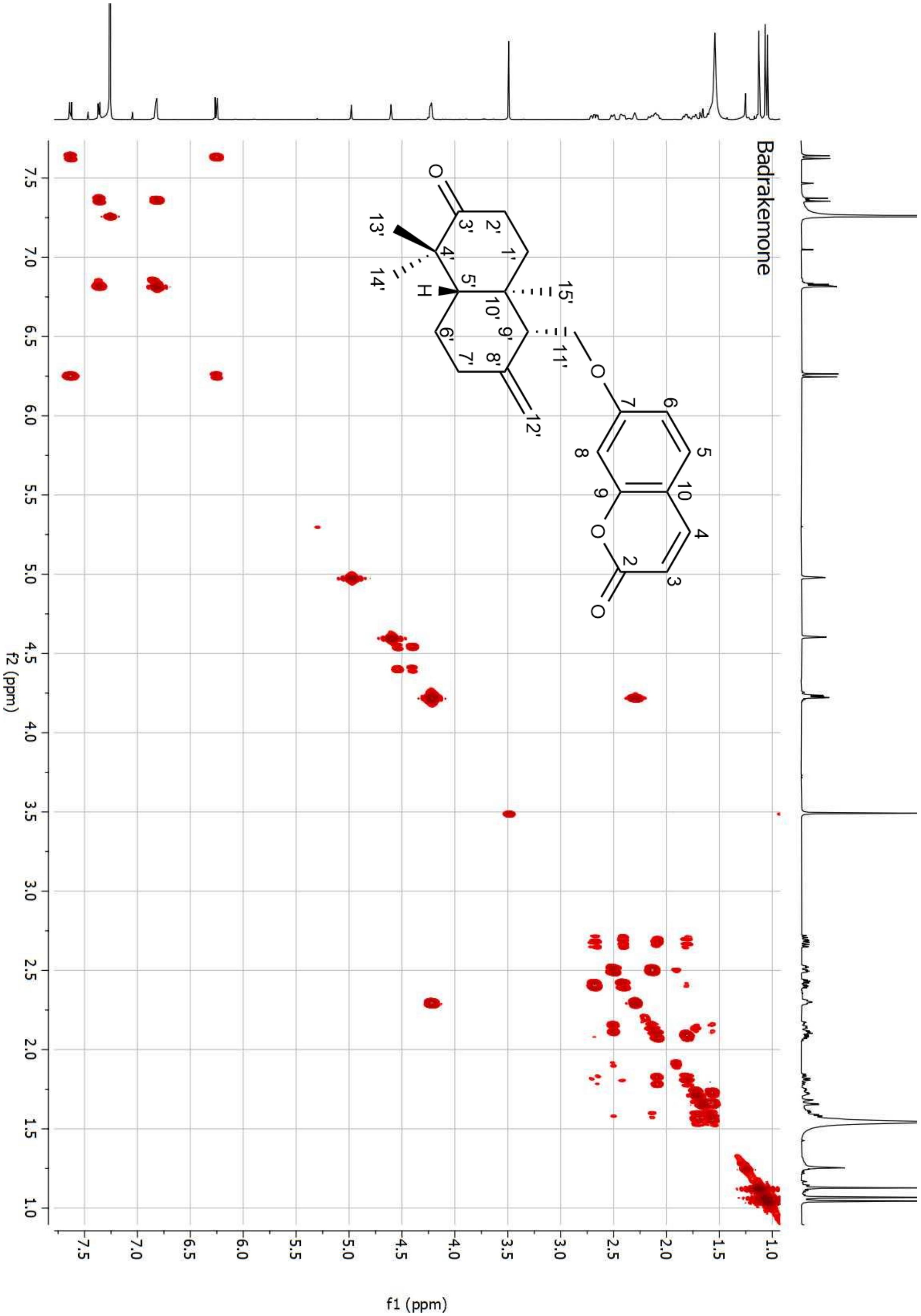

Figure S22. ^{1}H-^{1}H COSY spectrum ($CDCl_3$) of badrakemone (**4**)

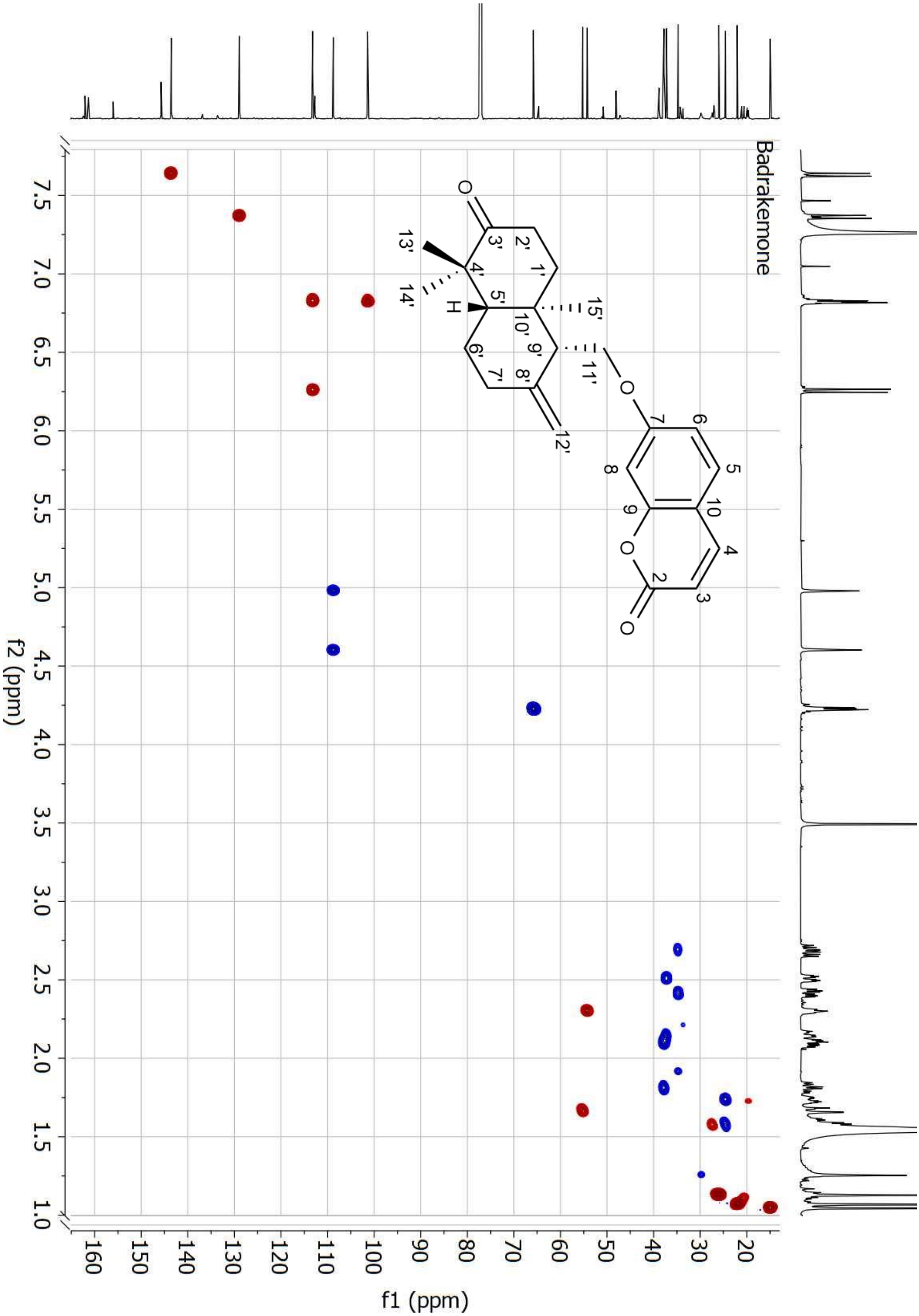

Figure S23. HSQC spectrum ($CDCl_3$) of badrakemone (**4**)

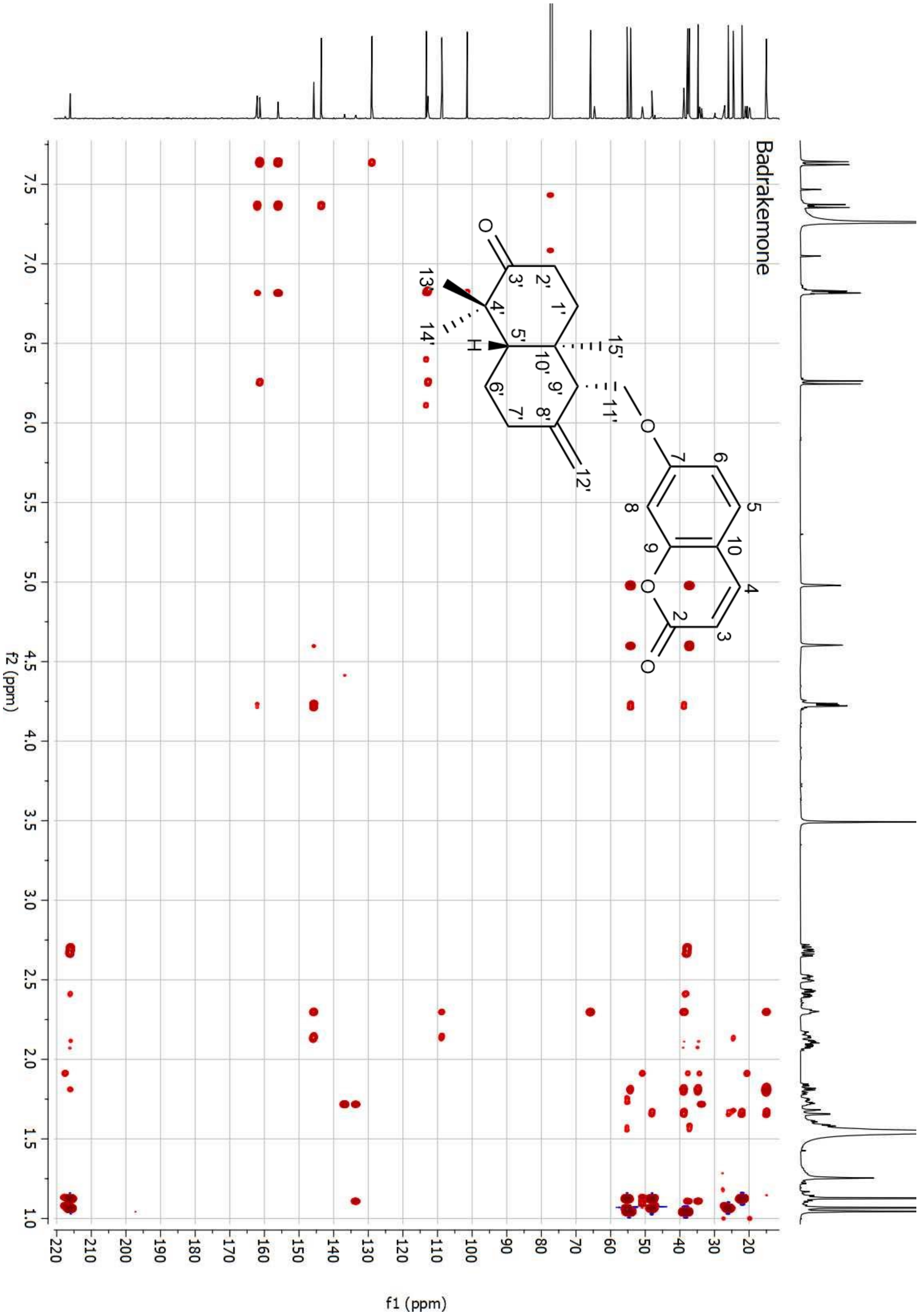

Figure S24. HMBC spectrum ($CDCl_3$) of badrakemone (**4**)

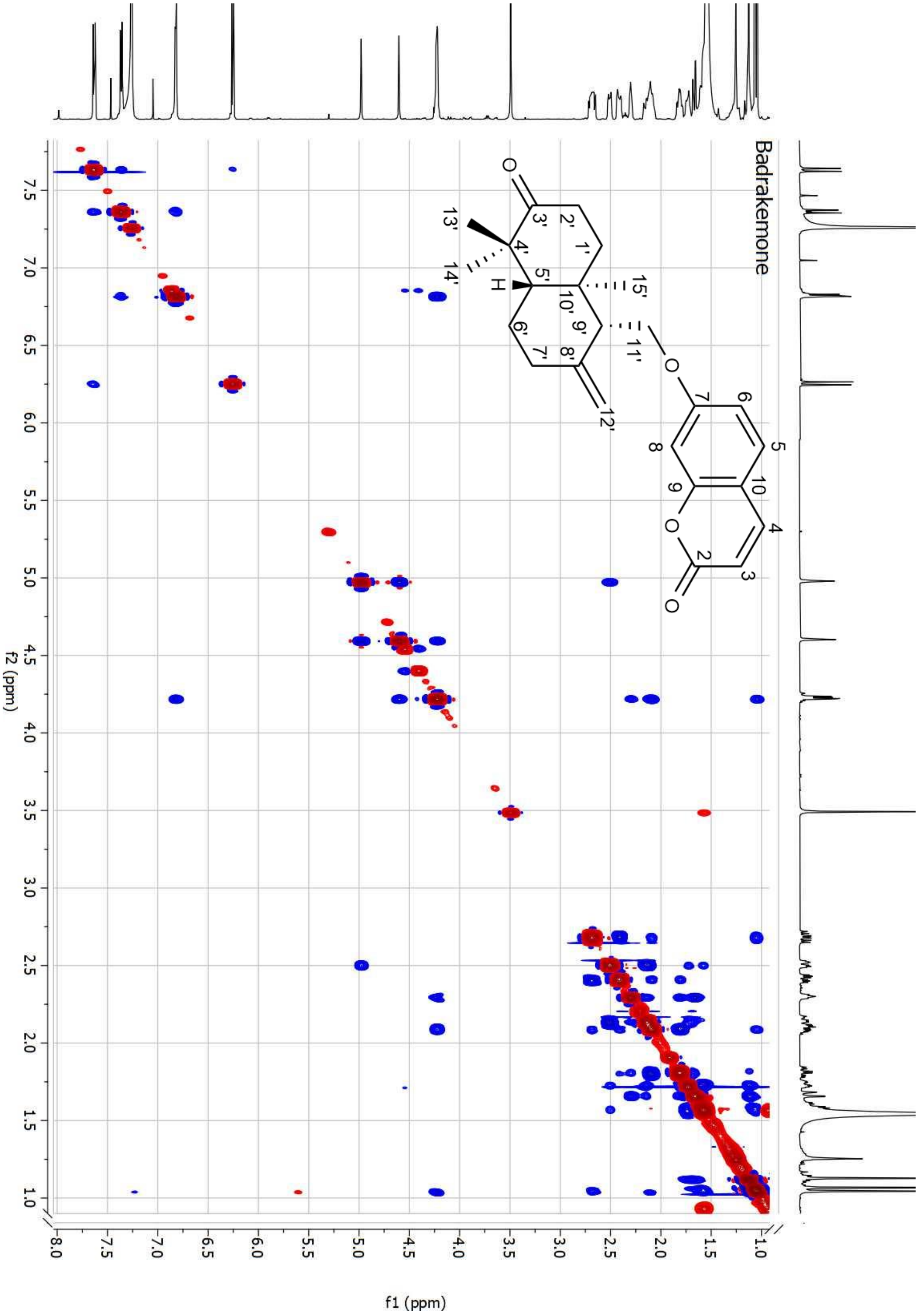

Figure S25. NOESY spectrum ($CDCl_3$) of badrakemone (**4**)

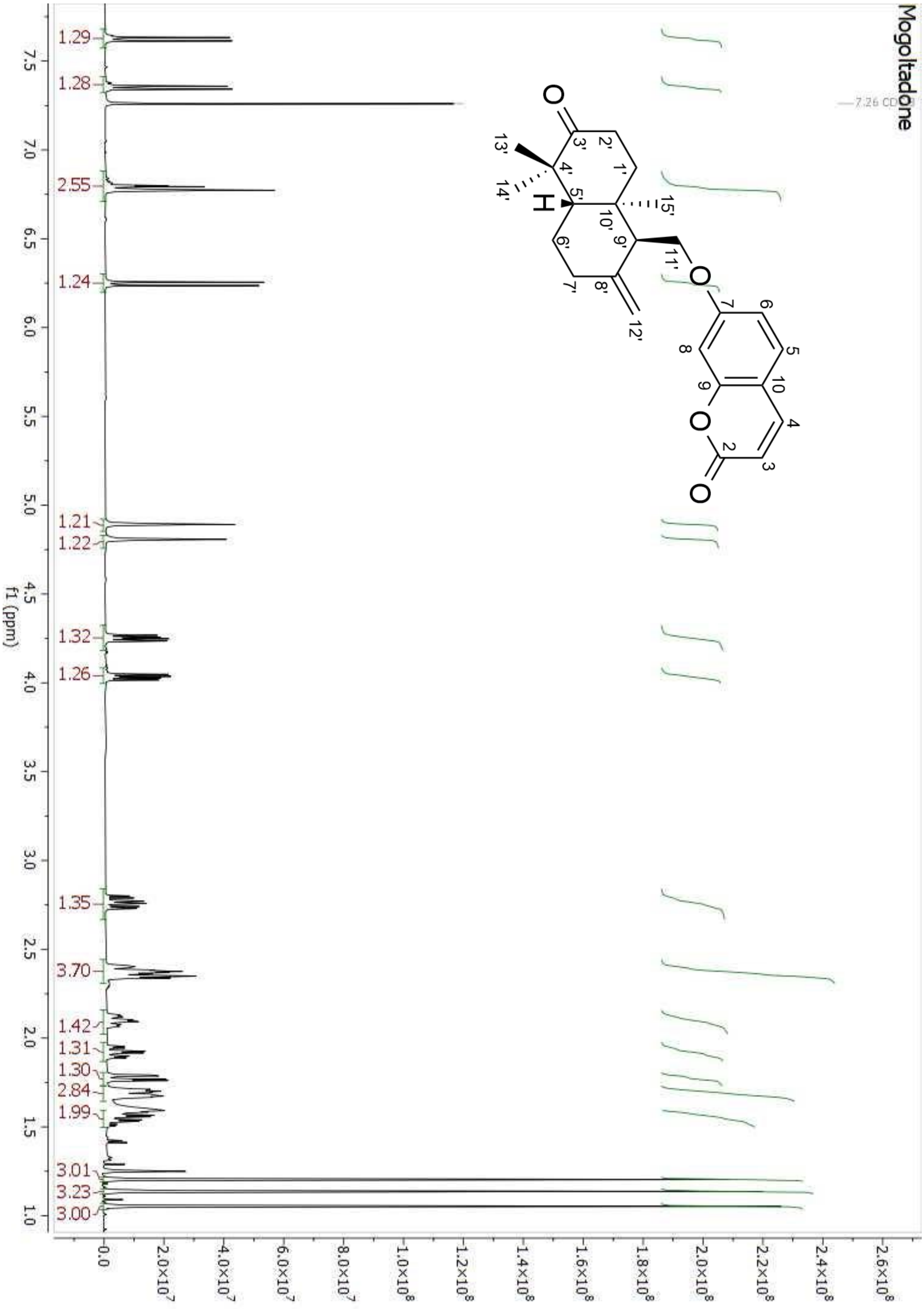

Figure S26. ^{1}H-NMR spectrum (500 MHz, $CDCl_3$) of mogoltadone (**5**)

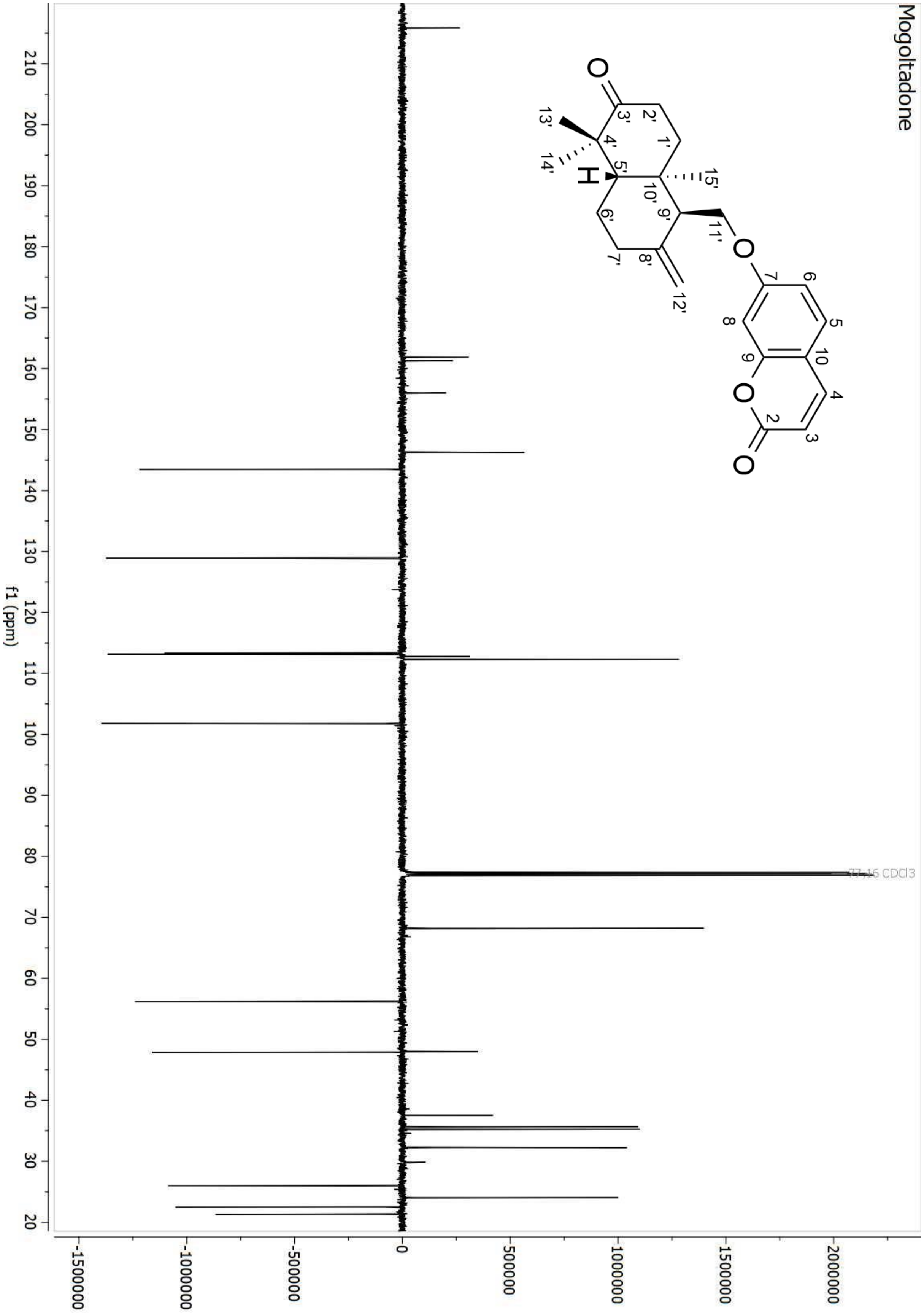

Figure S27. ^{13}C-NMR (APT) spectrum (125 MHz, $CDCl_3$) of mogoltadone (**5**)

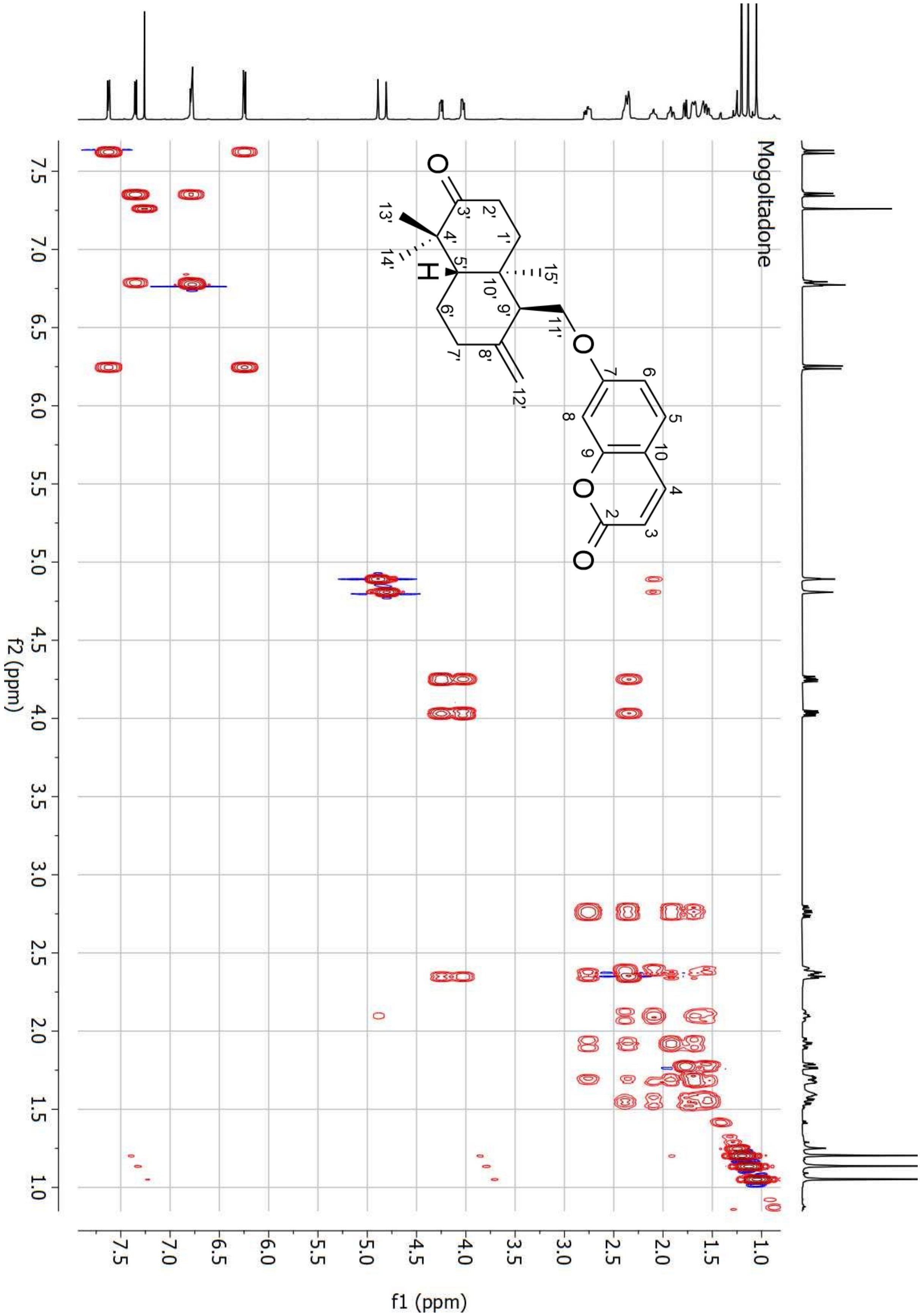

Figure S28. ^{1}H-^{1}H COSY spectrum ($CDCl_3$) of mogoltadone (**5**)

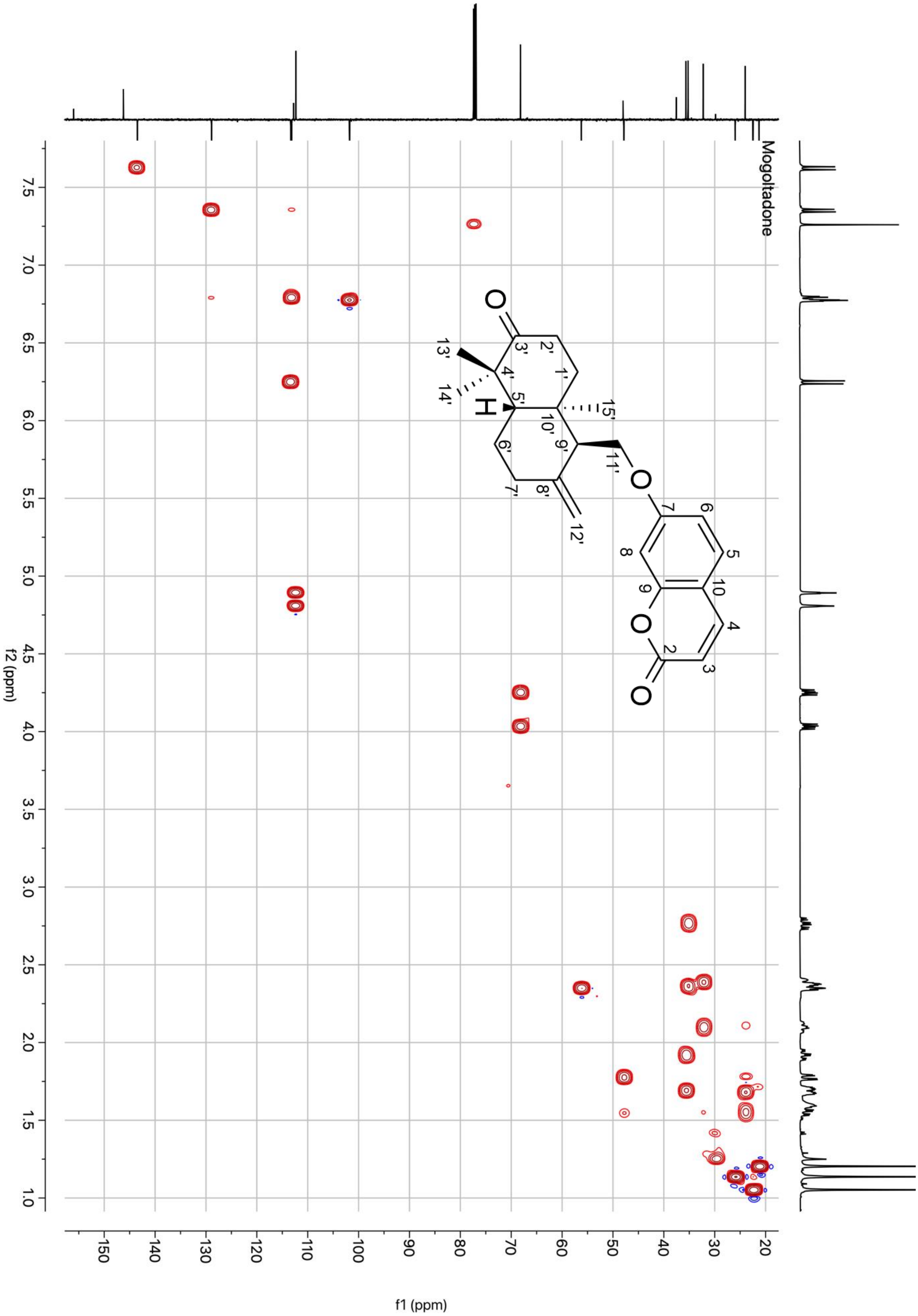

Figure S29. HSQC spectrum ($CDCl_3$) of mogoltadone (**5**)

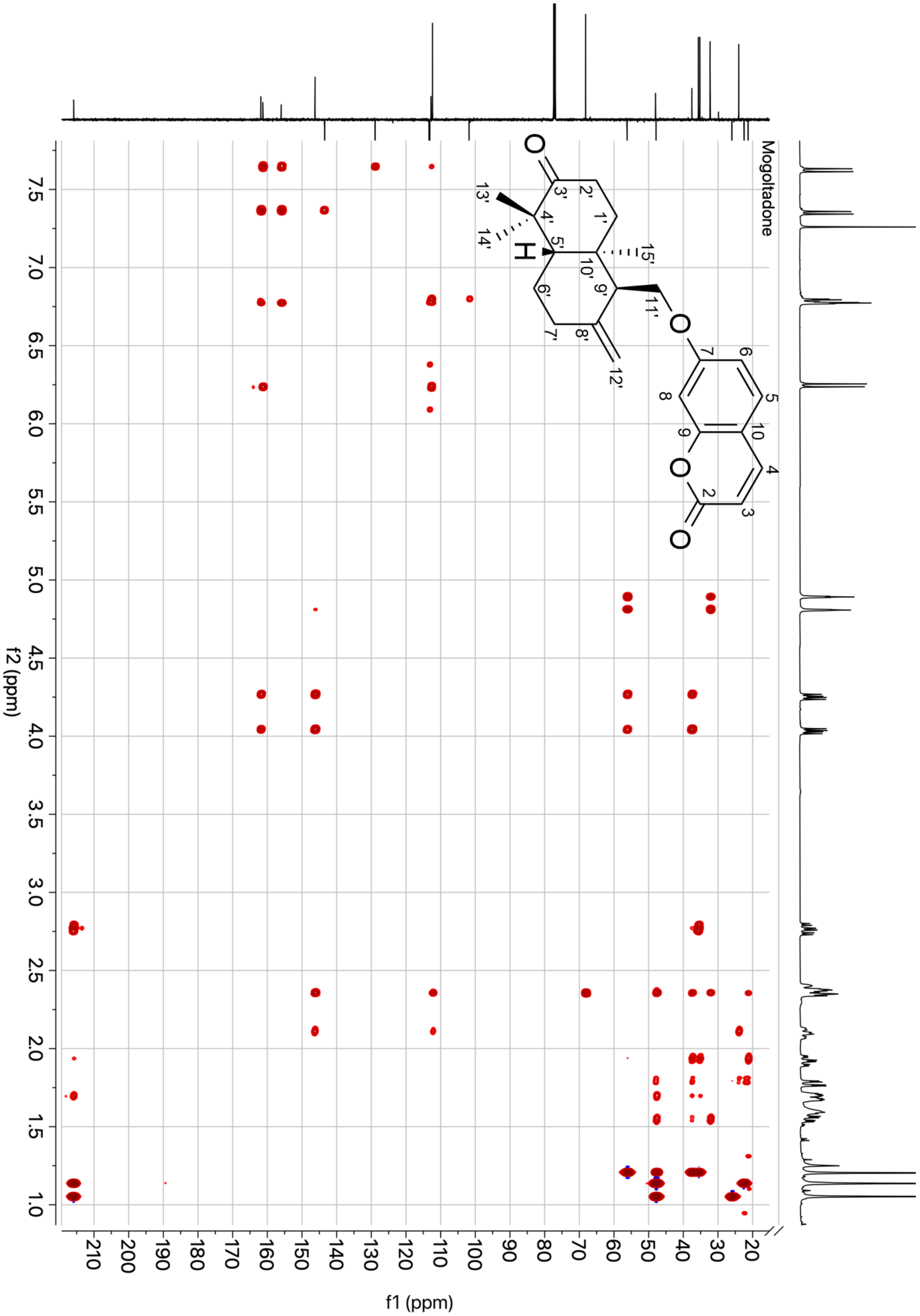

Figure S30. HMBC spectrum ($CDCl_3$) of mogoltadone (**5**)

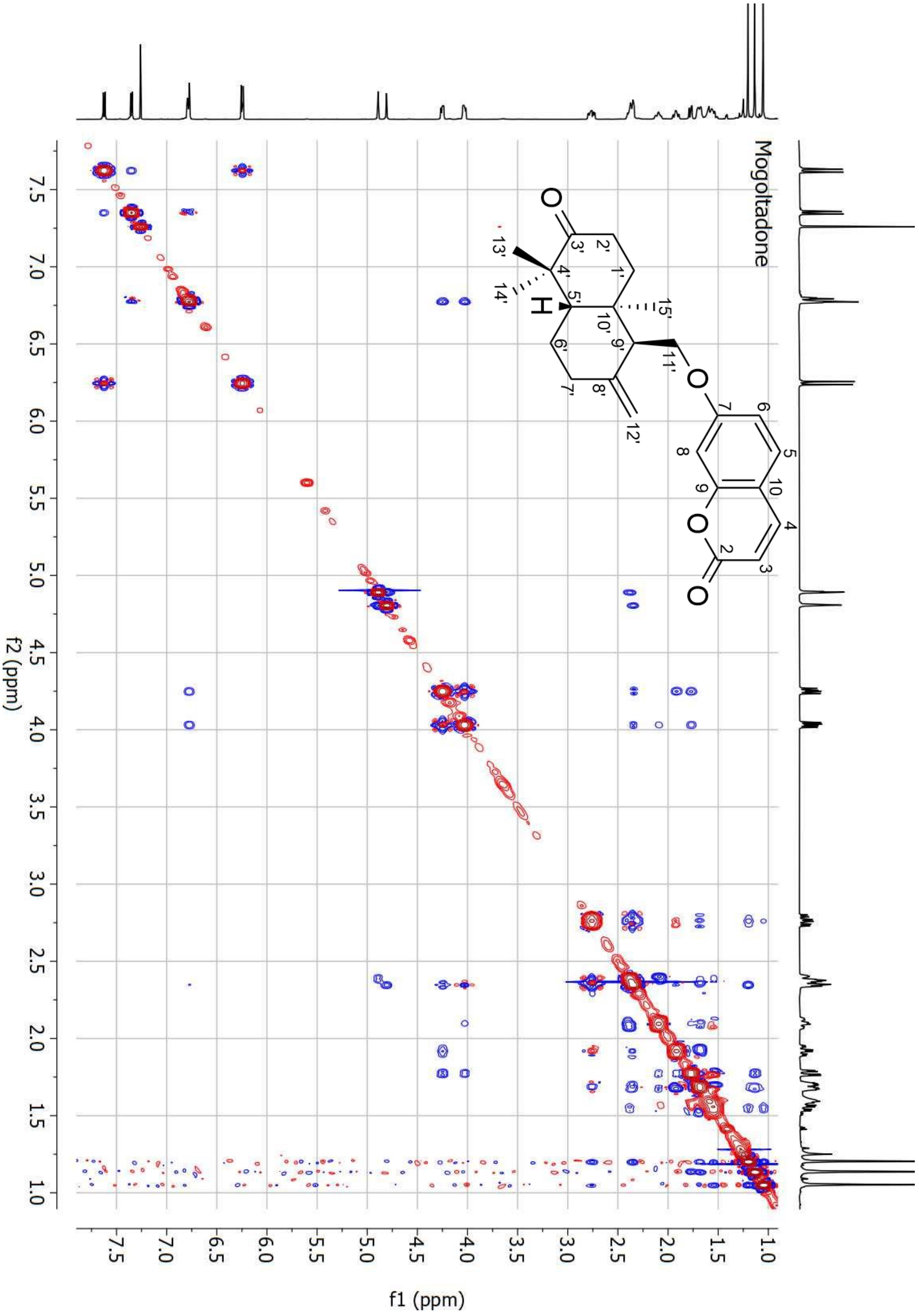

Figure S31. NOESY spectrum ($CDCl_3$) of mogoltadone (**5**)

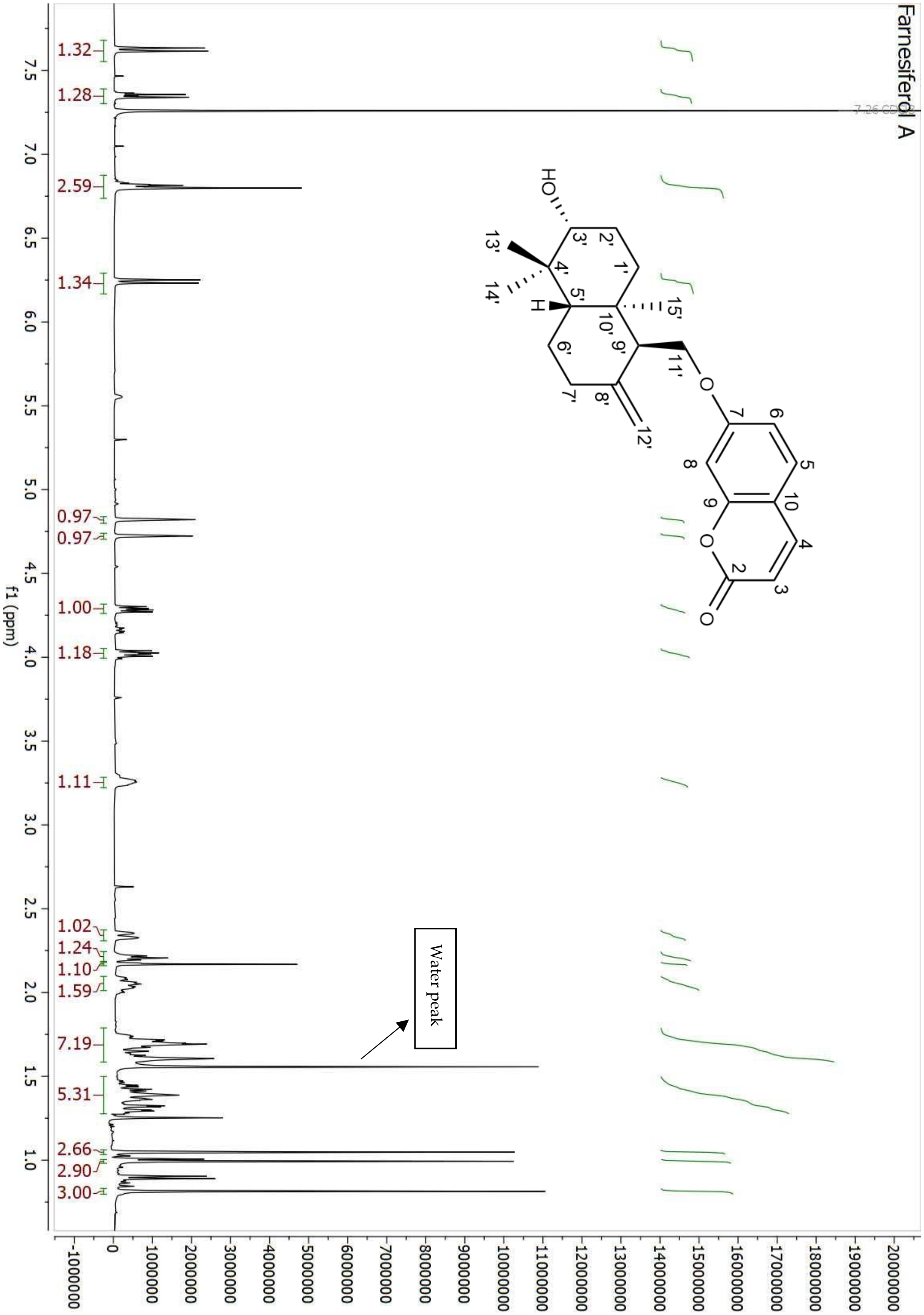

Figure S32. ^{1}H-NMR spectrum (500 MHz, $CDCl_3$) of farnesiferol A (**6**)

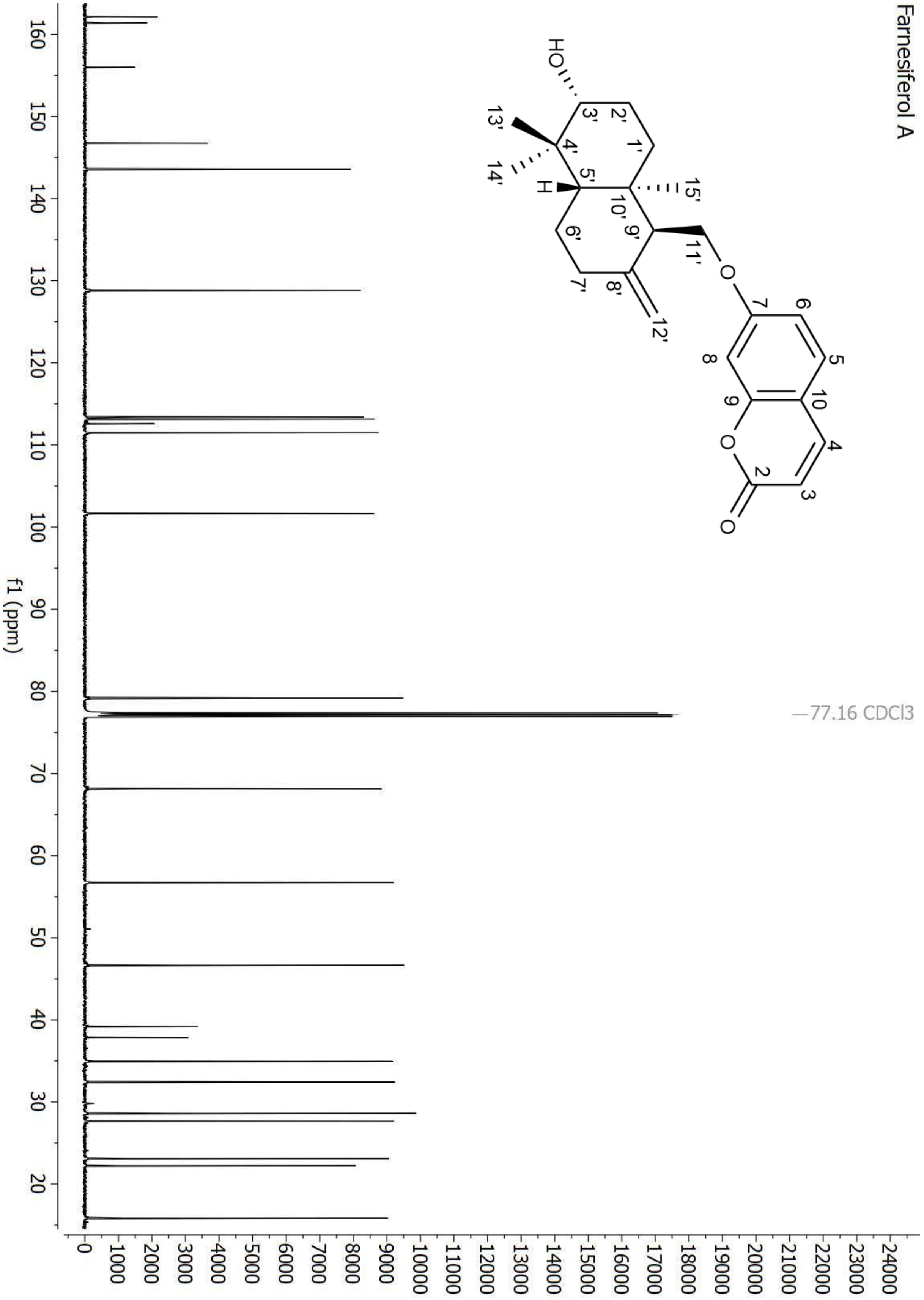

Figure S33. ^{13}C-NMR spectrum (125 MHz, $CDCl_3$) of farnesiferol A (**6**)

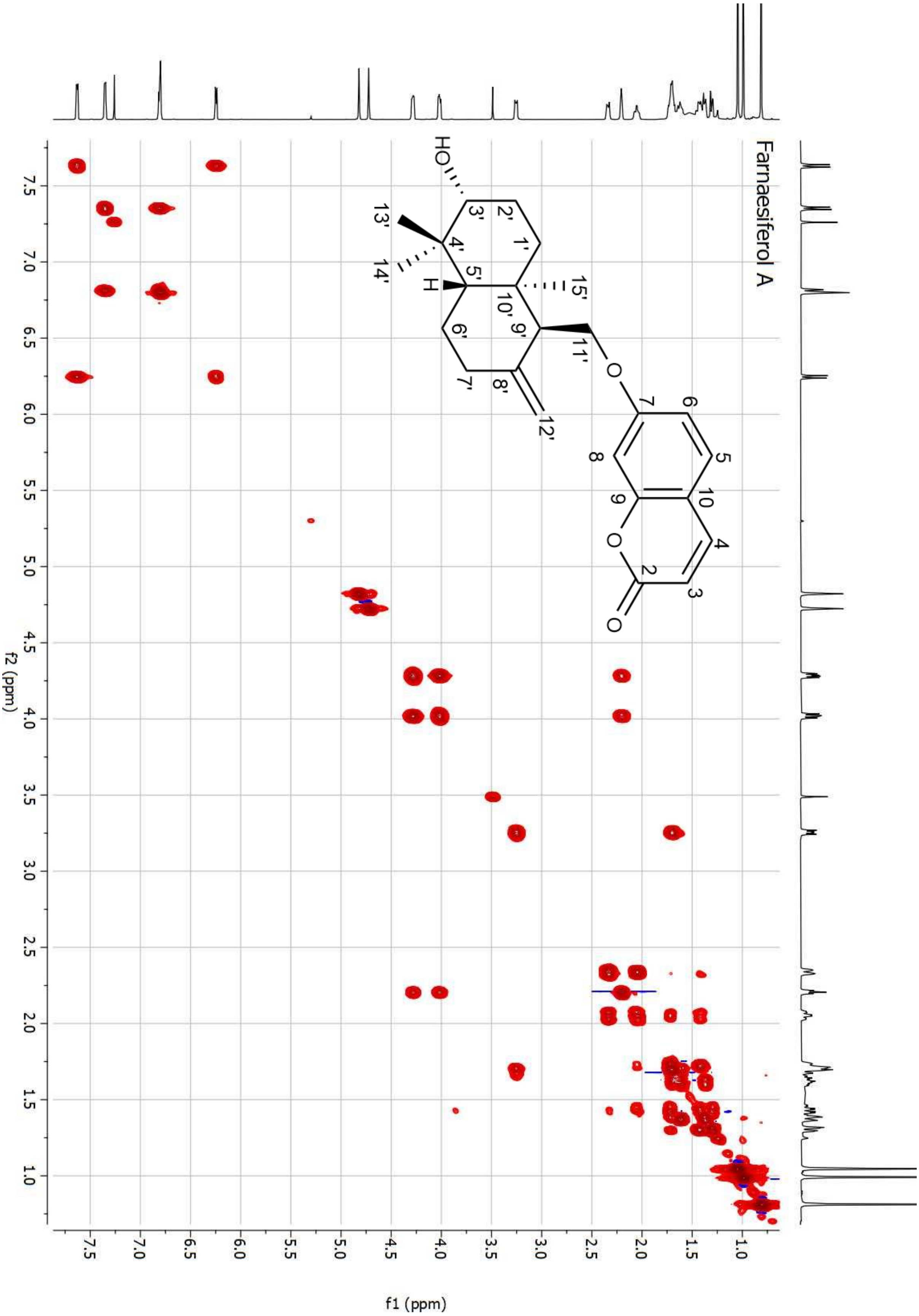

Figure S34. ^{1}H-^{1}H COSY spectrum ($CDCl_3$) of farnesiferol A (**6**)

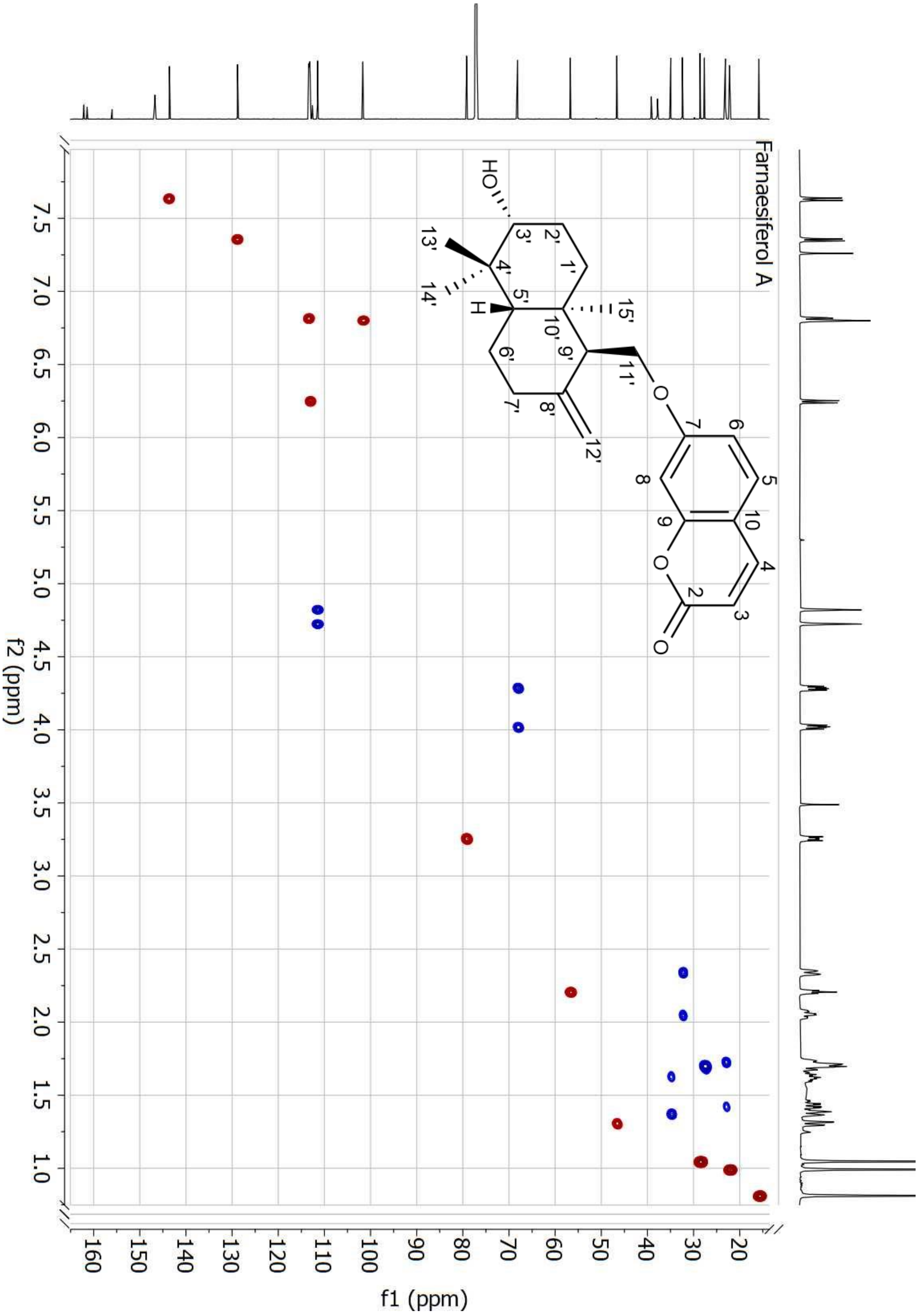

Figure S35. HSQC spectrum ($CDCl_3$) of farnesiferol A (**6**)

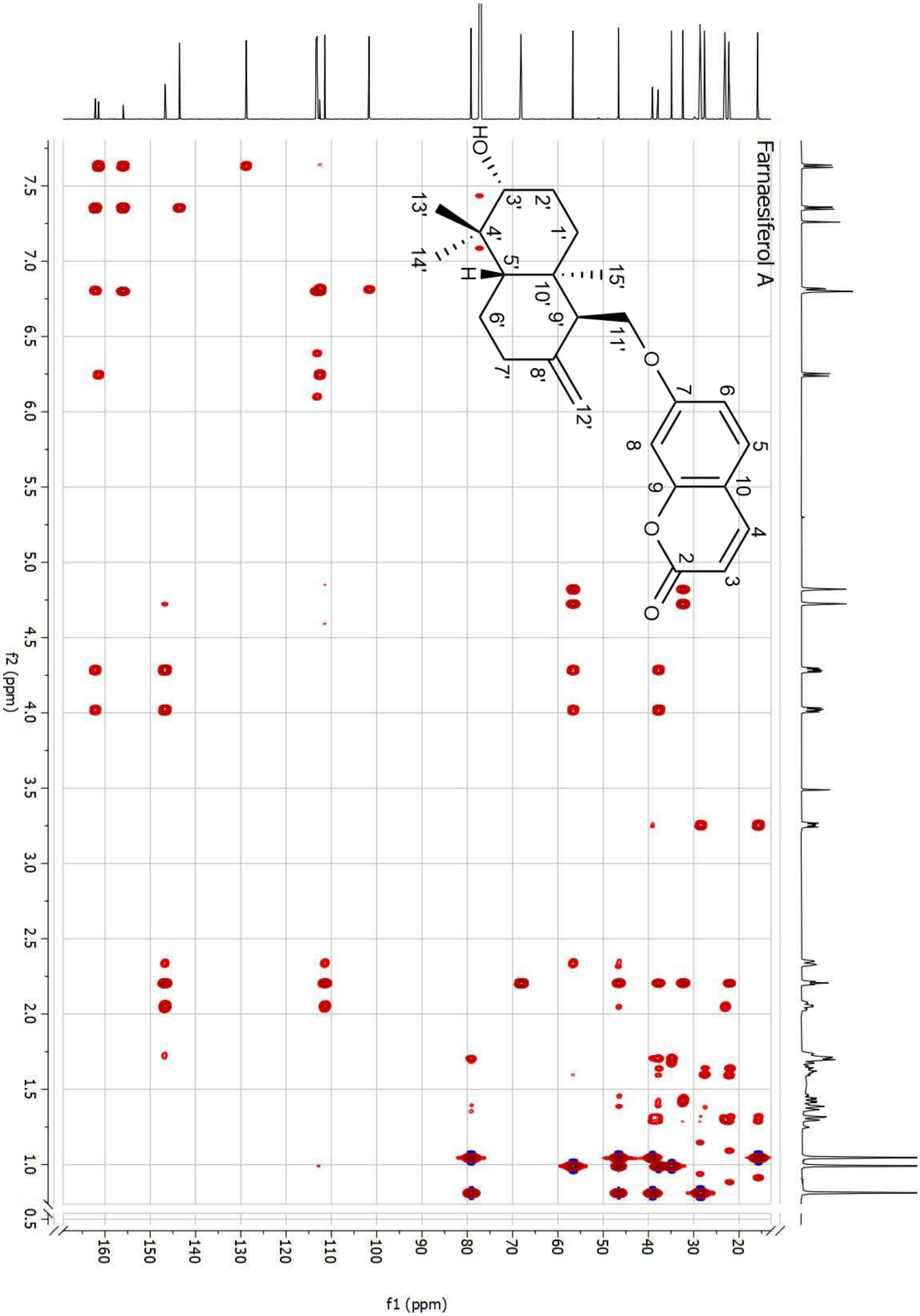

Figure S36. HMBC spectrum ($CDCl_3$) of farnesiferol A (**6**)

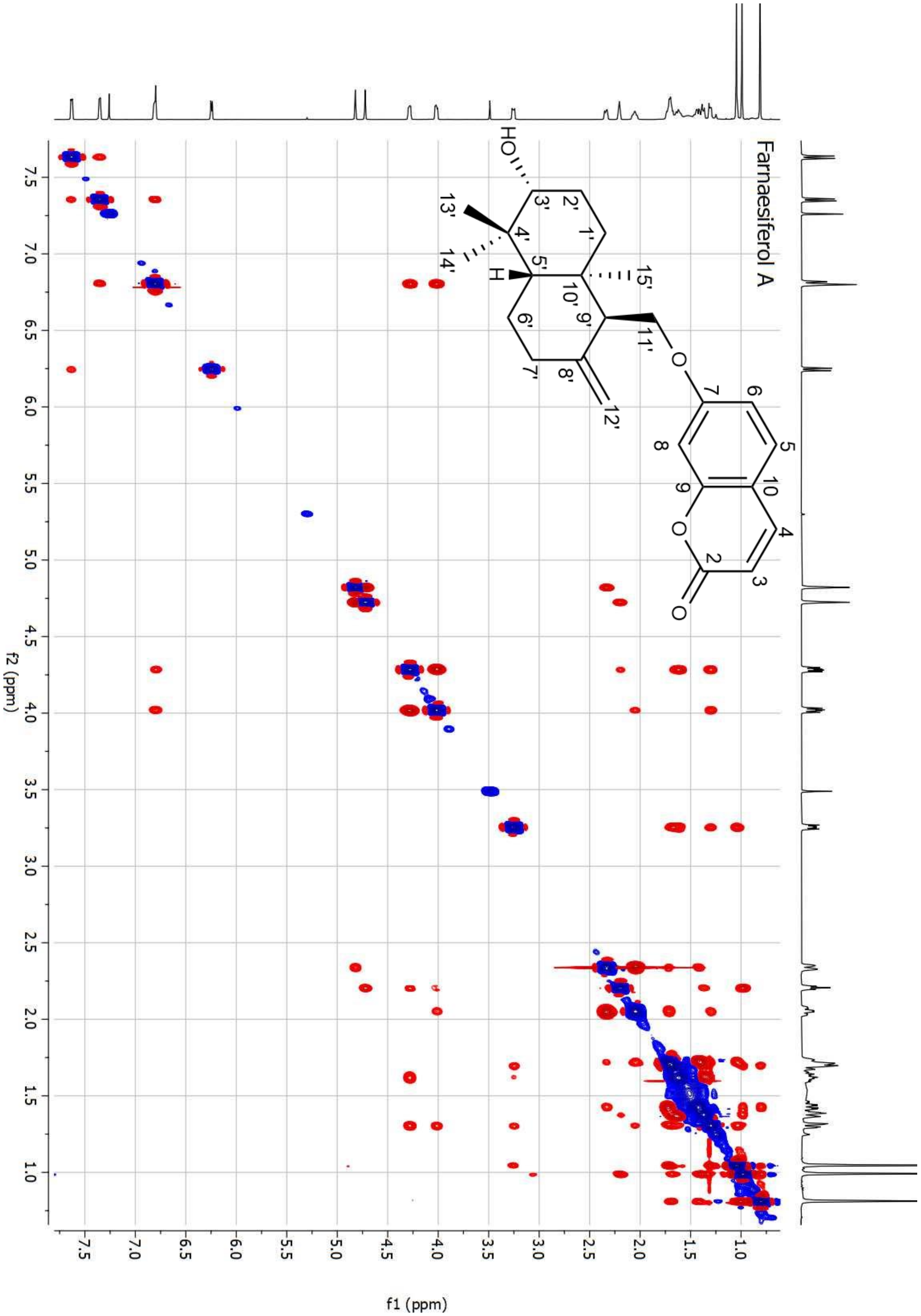

Figure S37. NOESY spectrum ($CDCl_3$) of farnesiferol A (**6**)

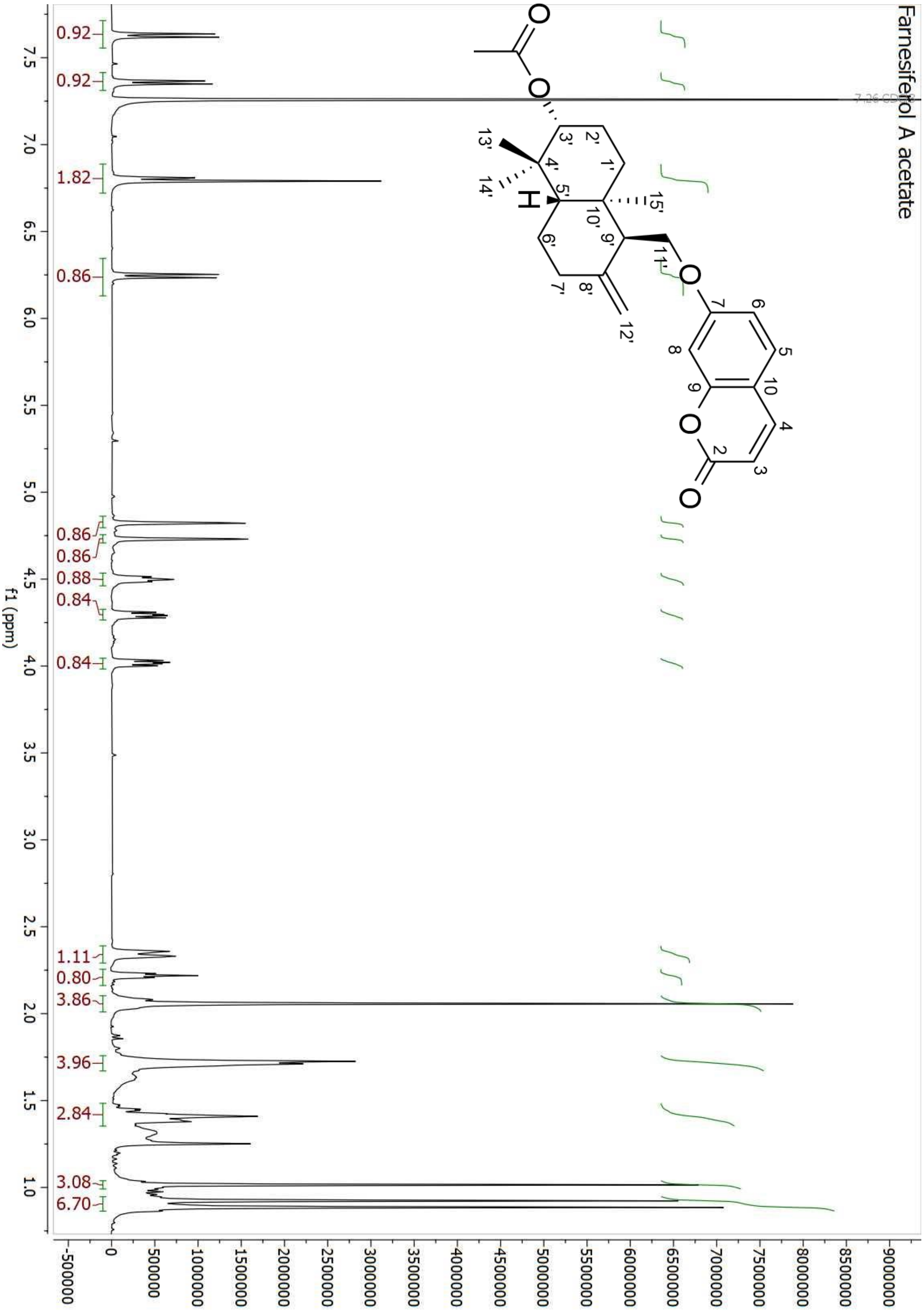

Figure S38. ^{1}H-NMR spectrum (500 MHz, $CDCl_3$) of farnesiferol A acetate (**7**)

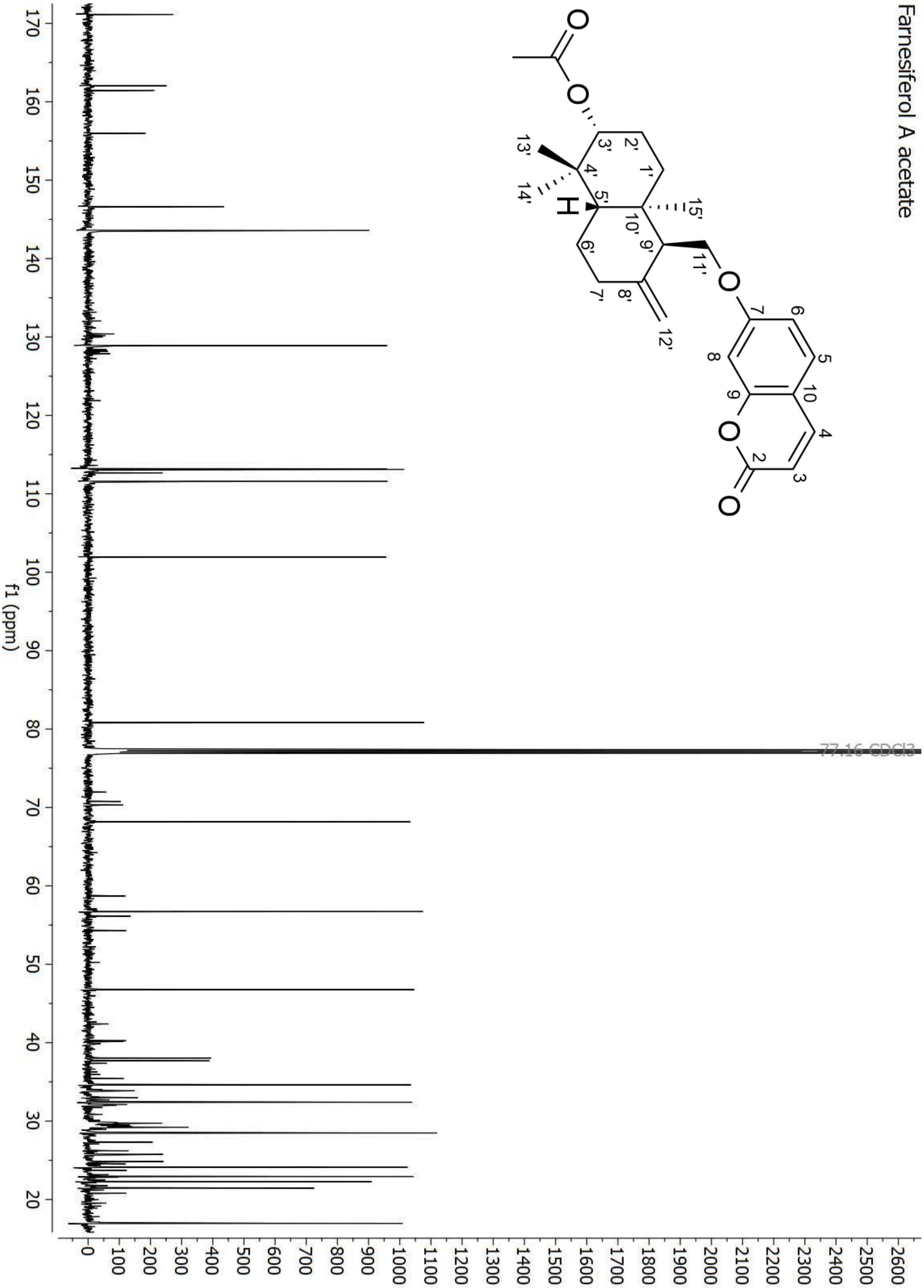

Figure S39. ^{13}C-NMR spectrum (125 MHz, $CDCl_3$) of farnesiferol A acetate (**7**)

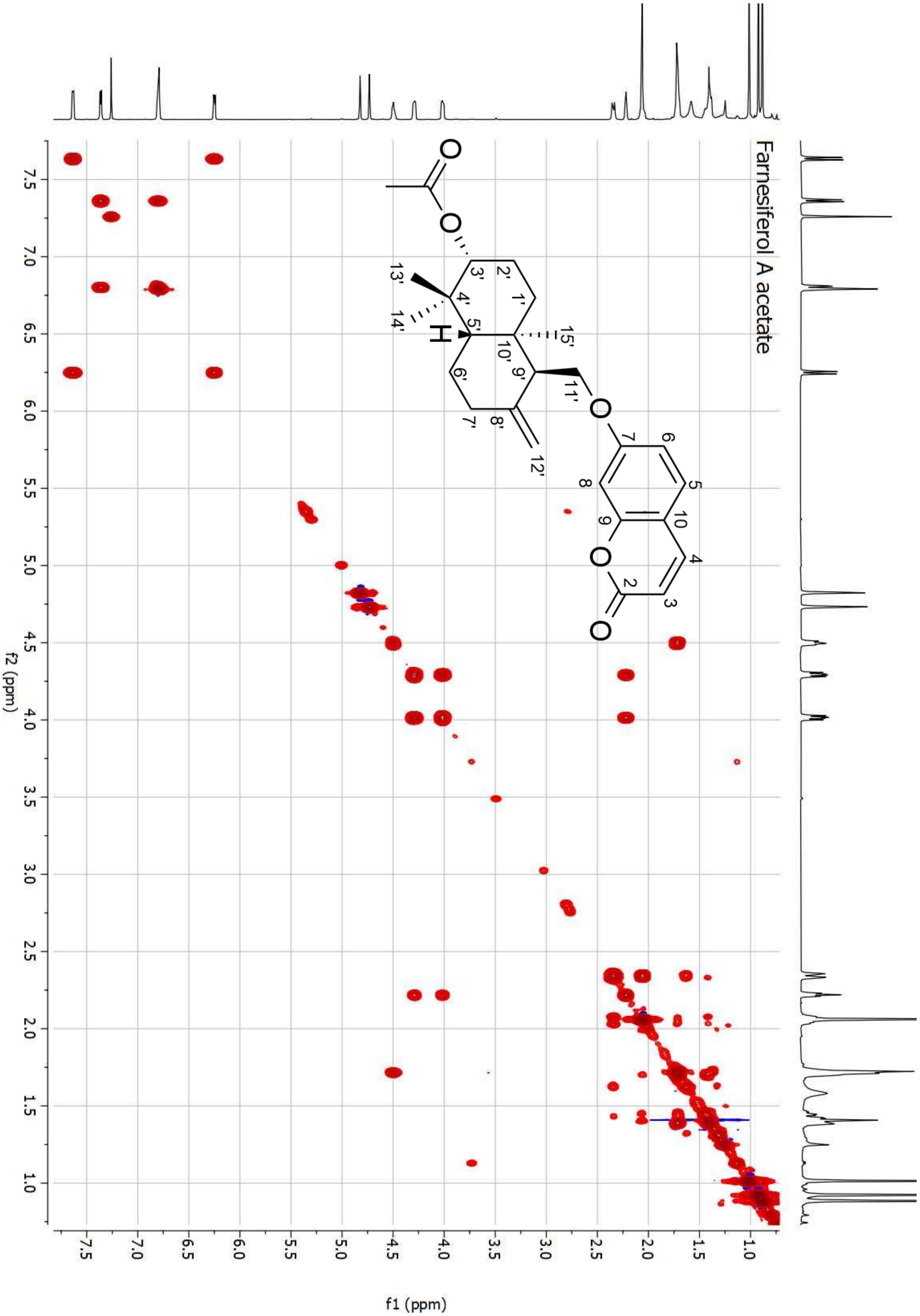

Figure S40. ^{1}H-^{1}H COSY spectrum ($CDCl_3$) of farnesiferol A acetate (**7**)

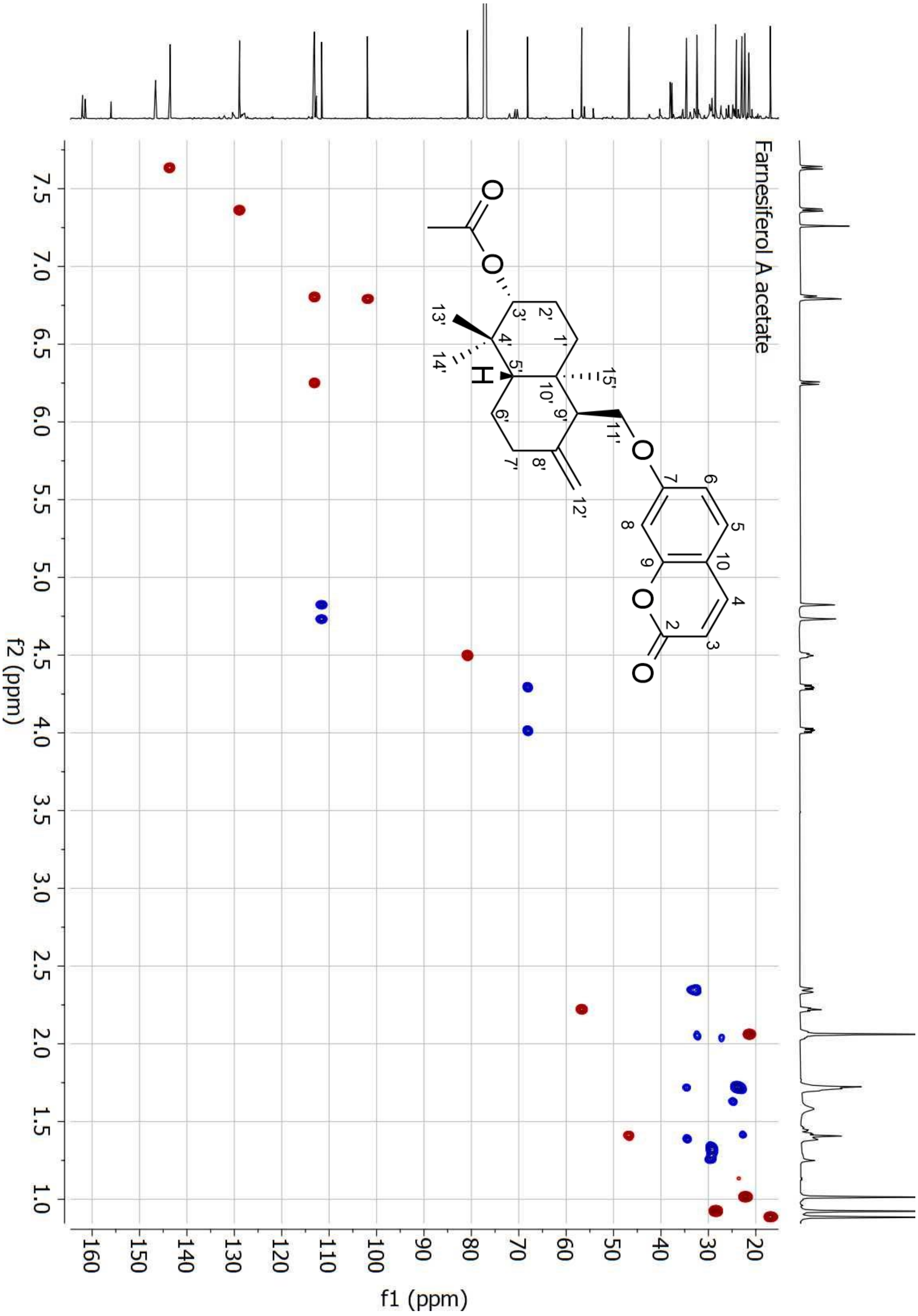

Figure S41. HSQC spectrum ($CDCl_3$) of farnesiferol A acetate (**7**)

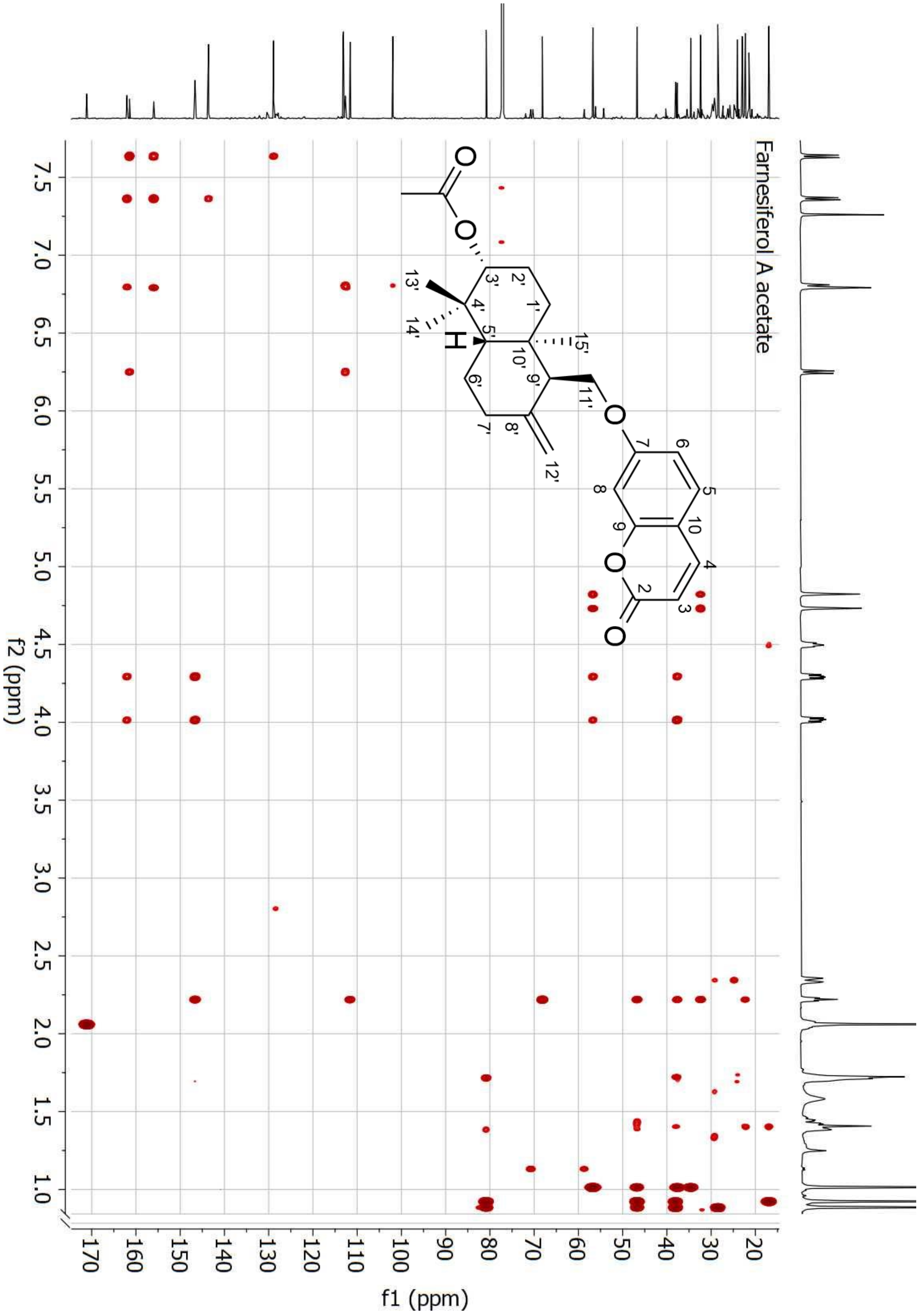

Figure S42. HMBC spectrum ($CDCl_3$) of farnesiferol A acetate (**7**)

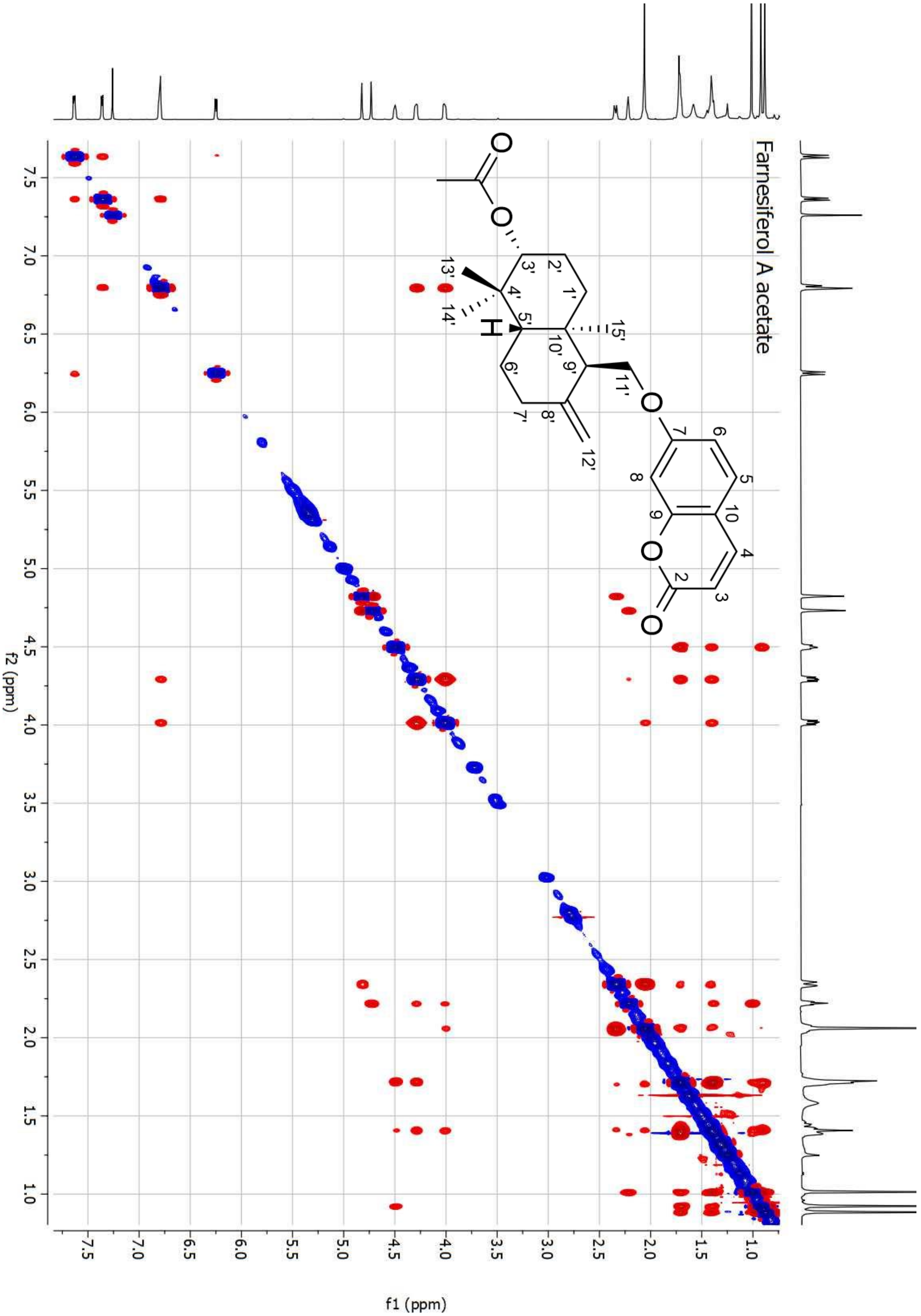

Figure S43. NOESY spectrum (CDCl3) of farnesiferol A acetate (**7**)

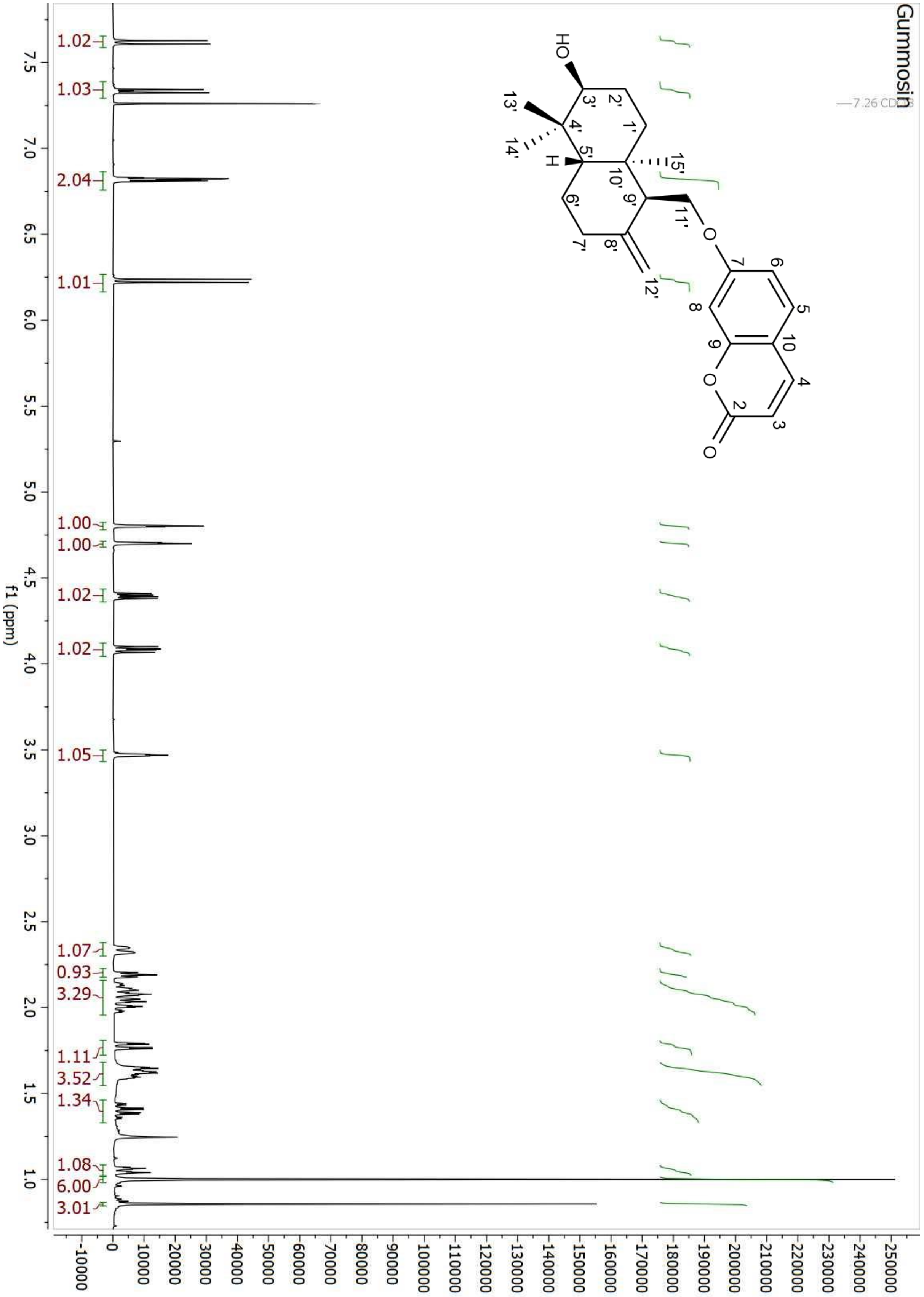

Figure S44. ^{1}H-NMR spectrum (500 MHz, $CDCl_3$) of gummosin (**8**)

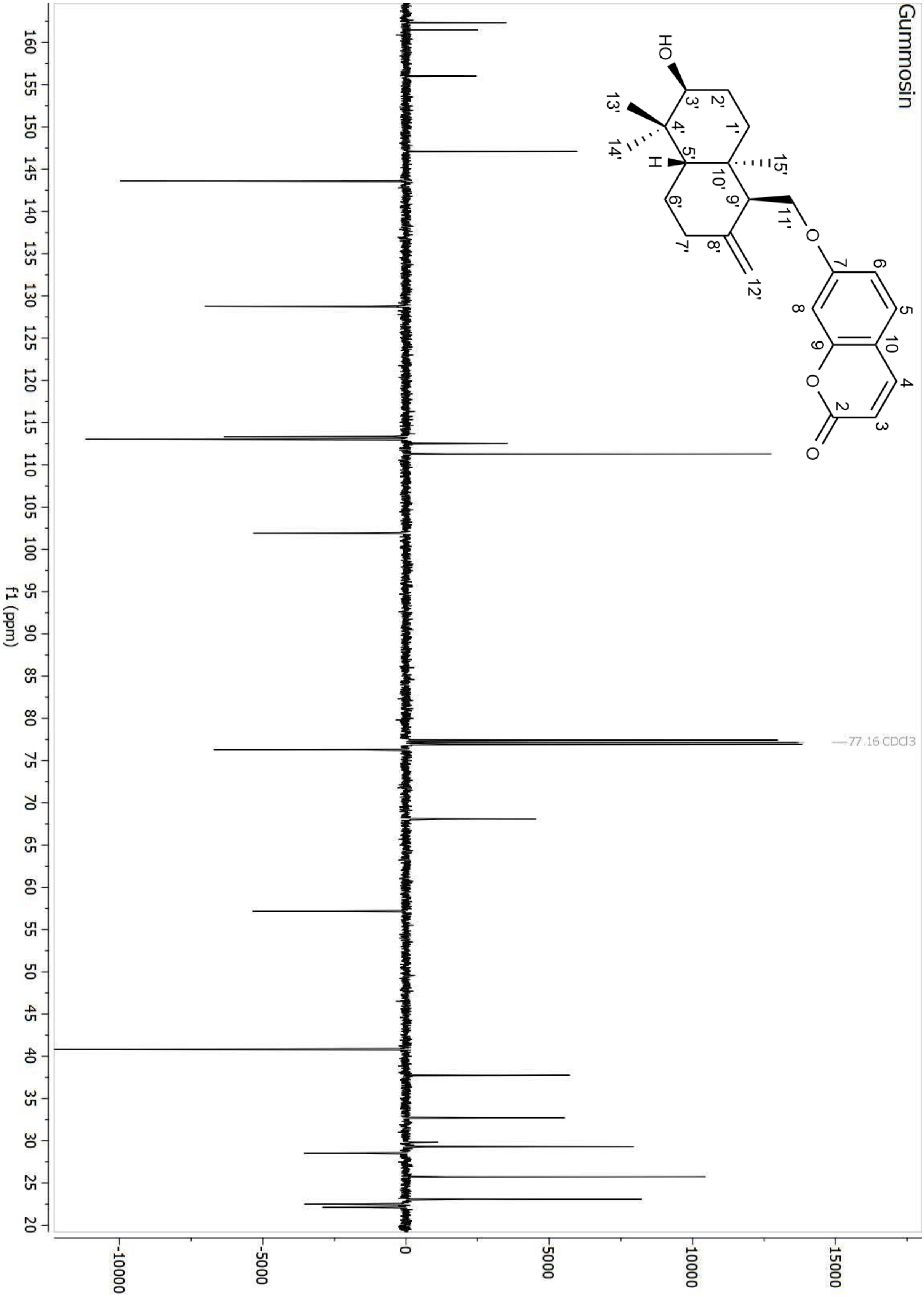

Figure S45. ^{13}C-NMR (APT) spectrum (125 MHz, $CDCl_3$) of gummosin (**8**)

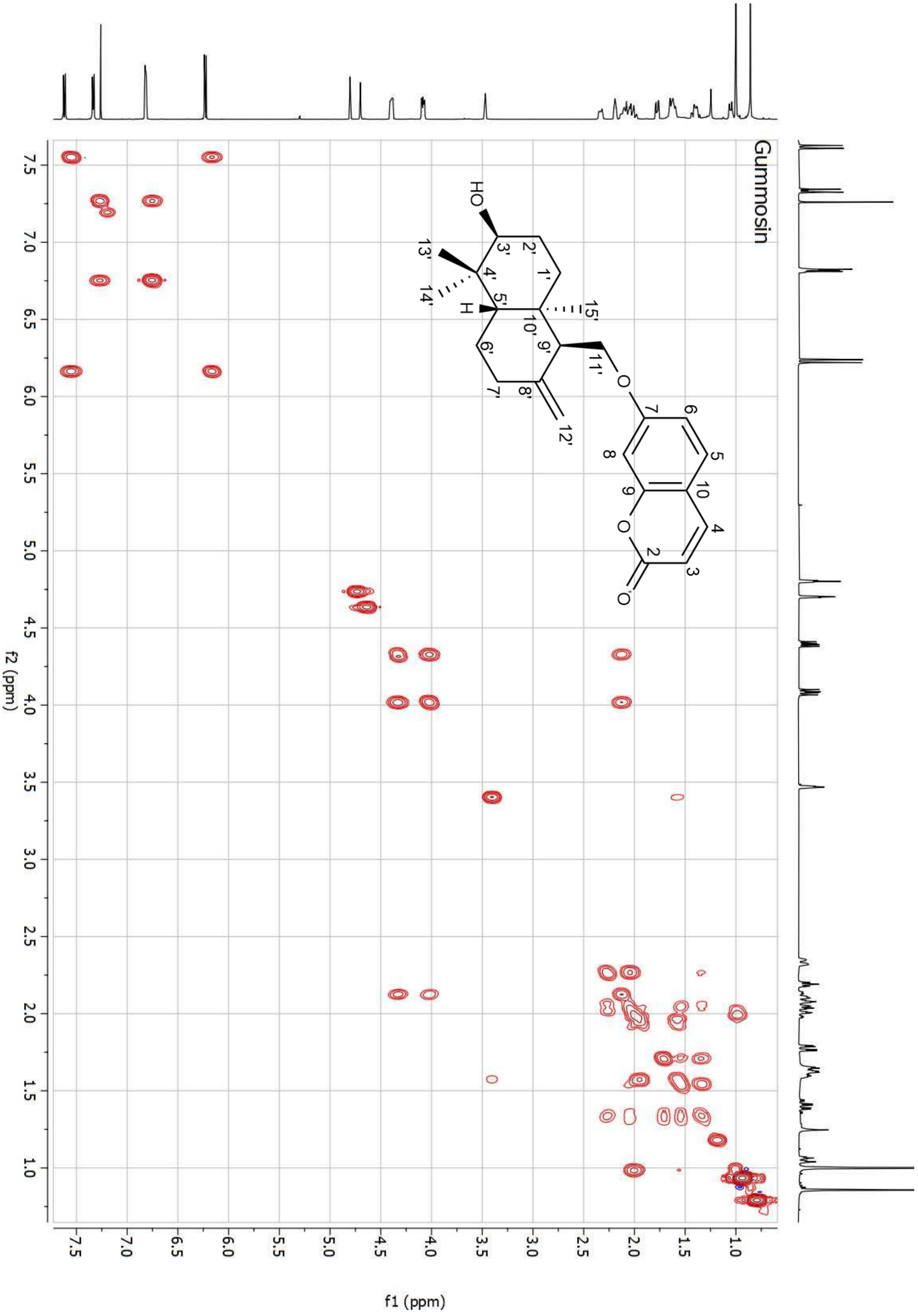

Figure S46. ^{1}H-^{1}H COSY spectrum ($CDCl_3$) of gummosin (**8**)

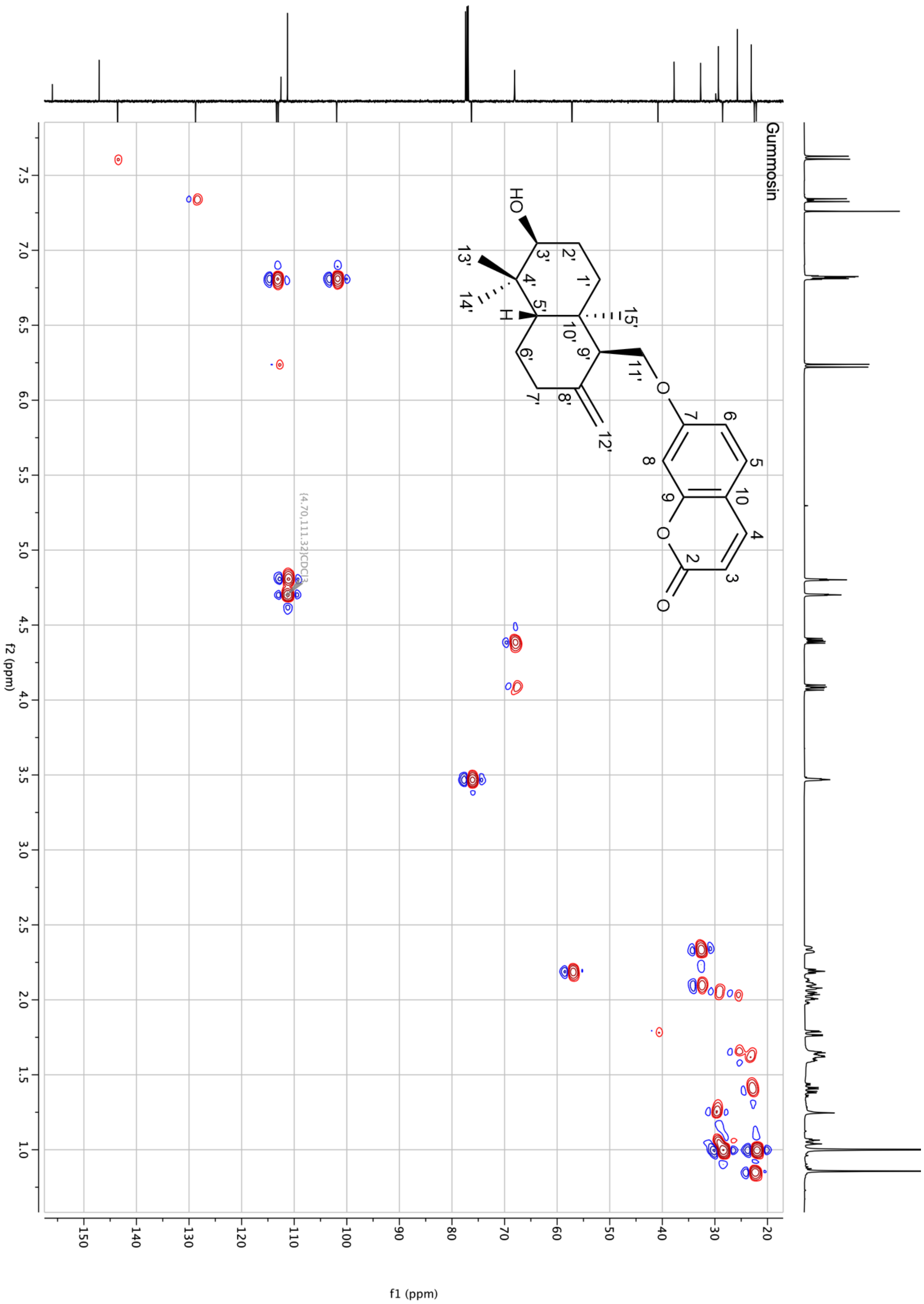

Figure S47. HSQC spectrum ($CDCl_3$) of gummosin (**8**)

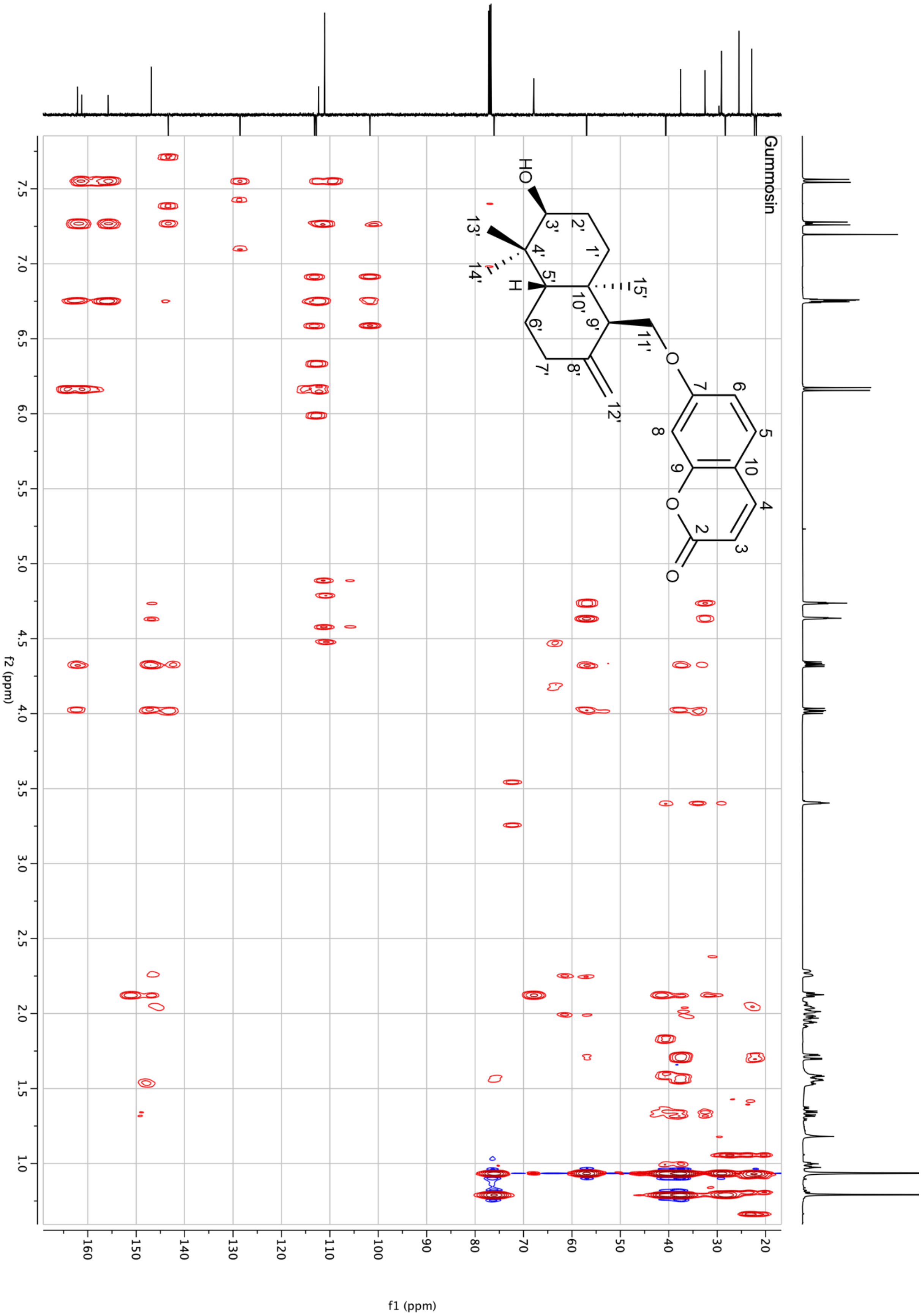

Figure S48. HMBC spectrum ($CDCl_3$) of gummosin (**8**)

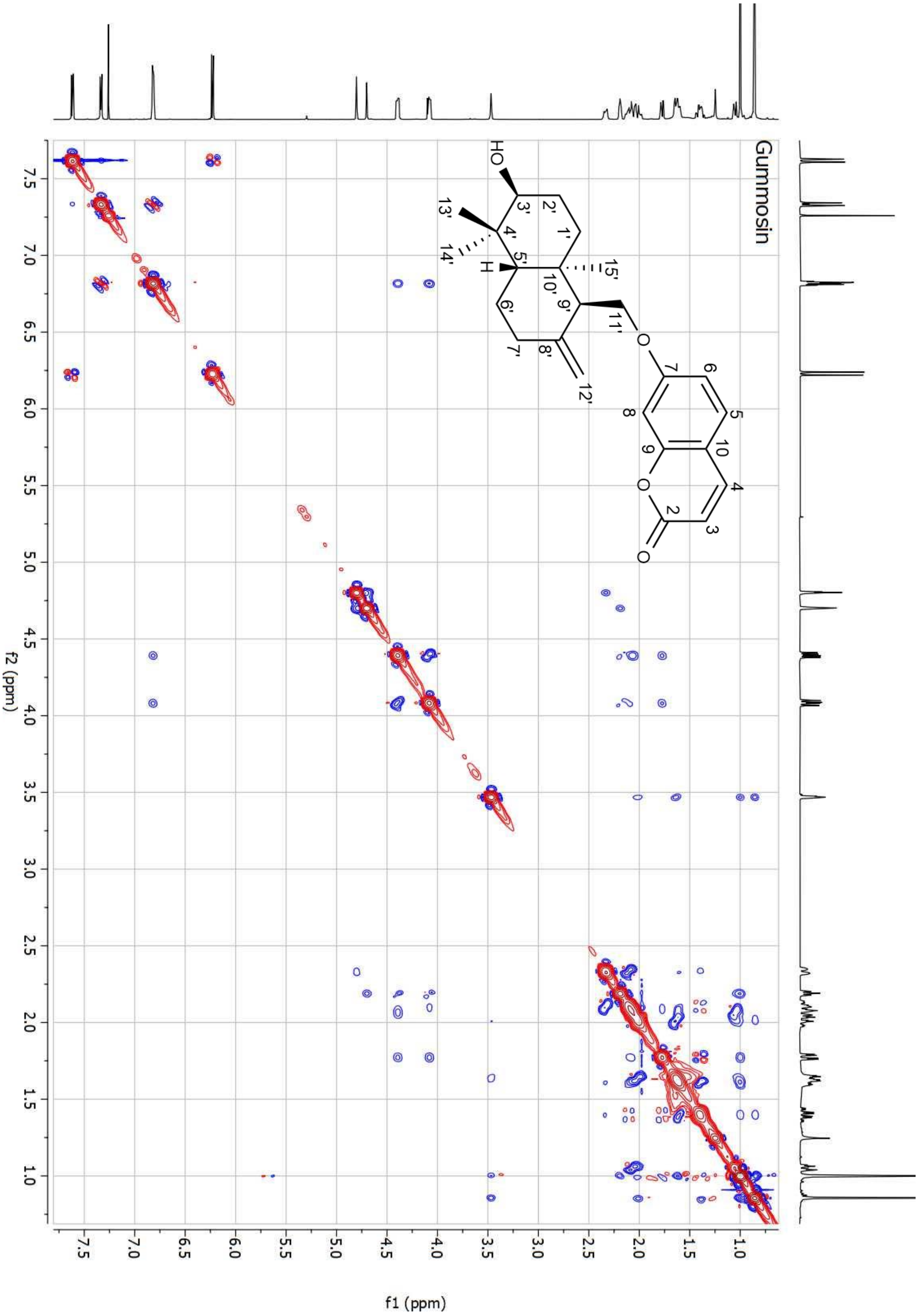

Figure S49. NOESY spectrum ($CDCl_3$) of gummosin (**8**)

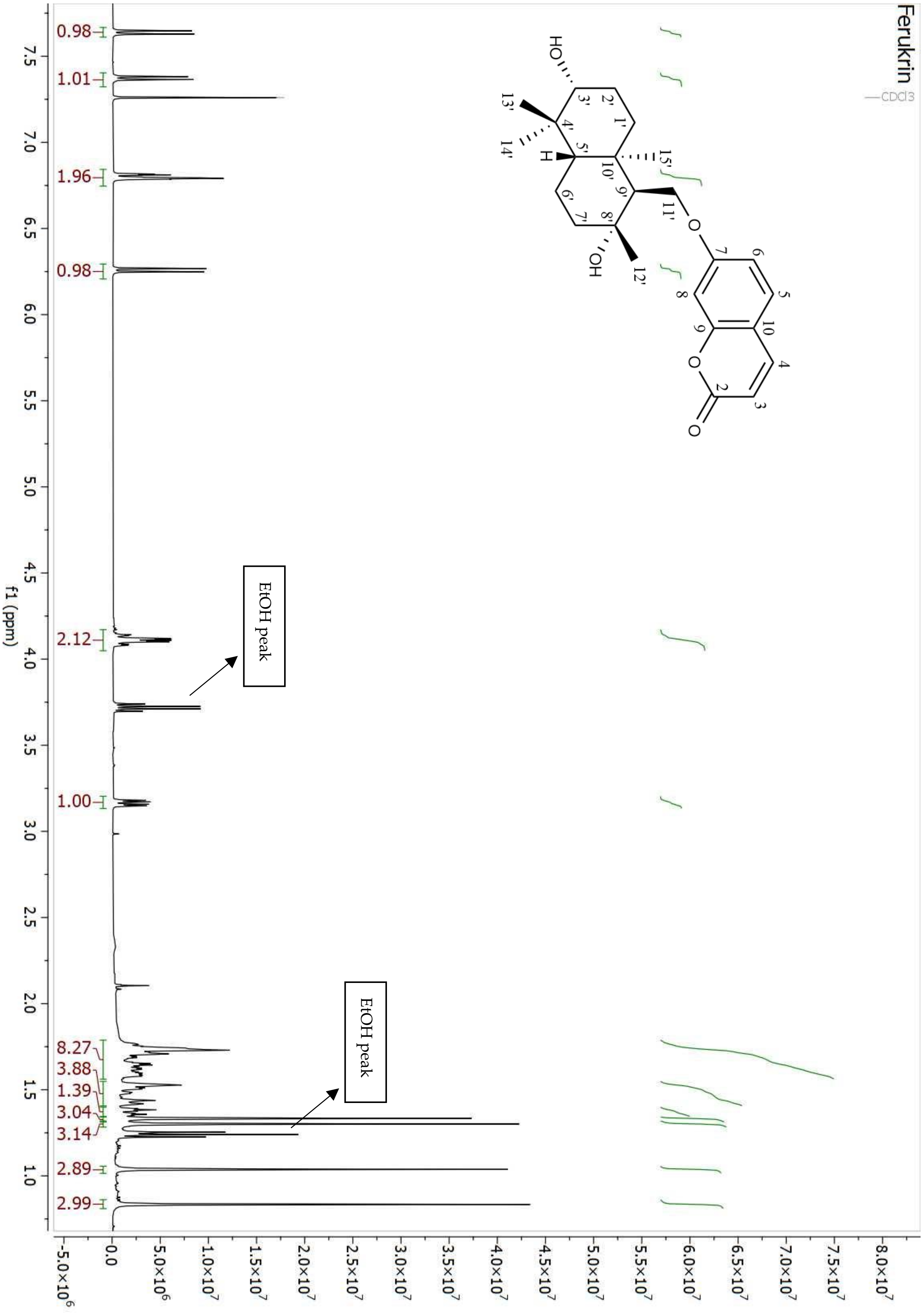

Figure S50. ^{1}H-NMR spectrum (500 MHz, CDCl3) of ferukrin (**9**)

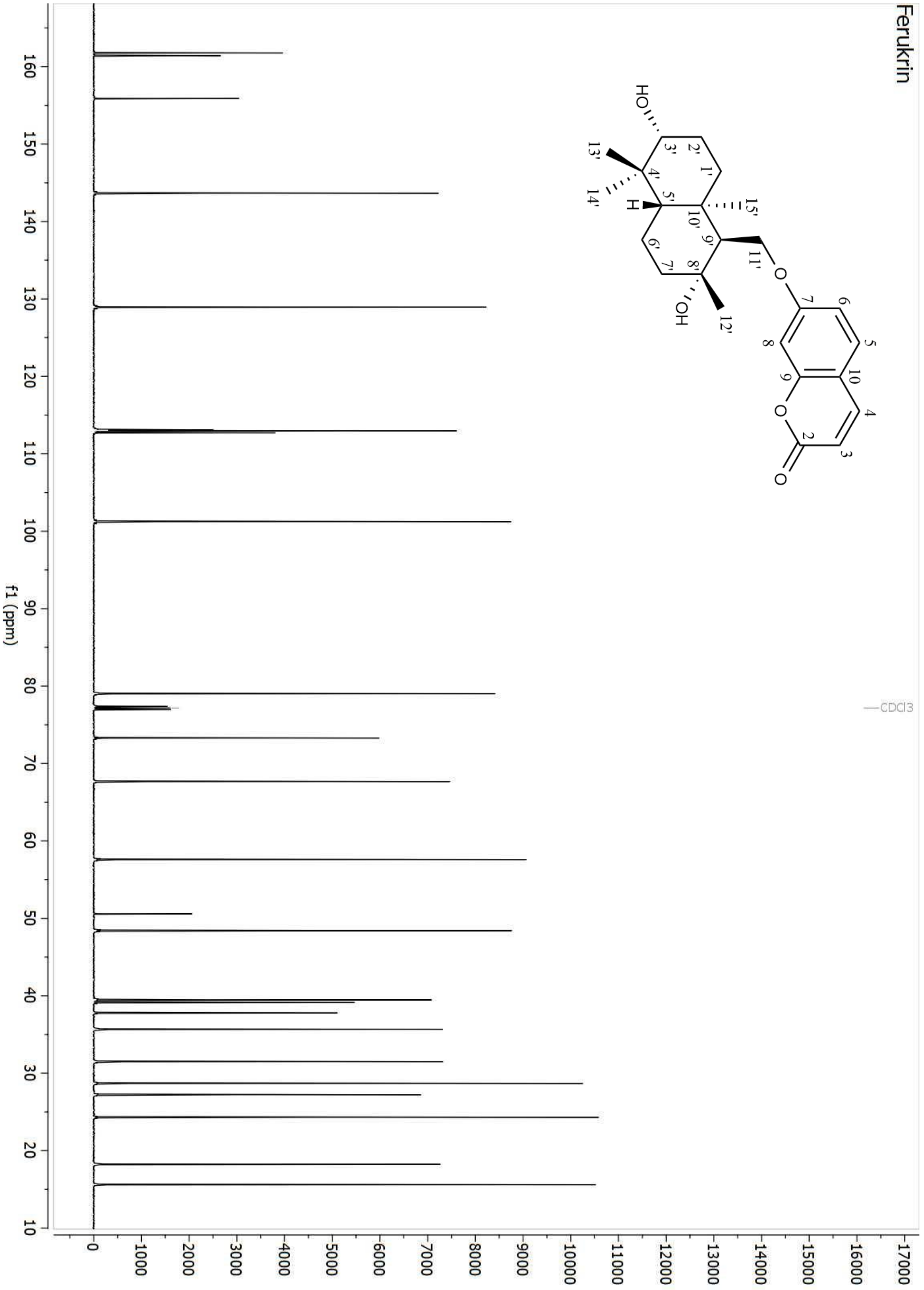

Figure S51. ^{13}C-NMR spectrum (125 MHz, $CDCl_3$) of ferukrin (**9**)

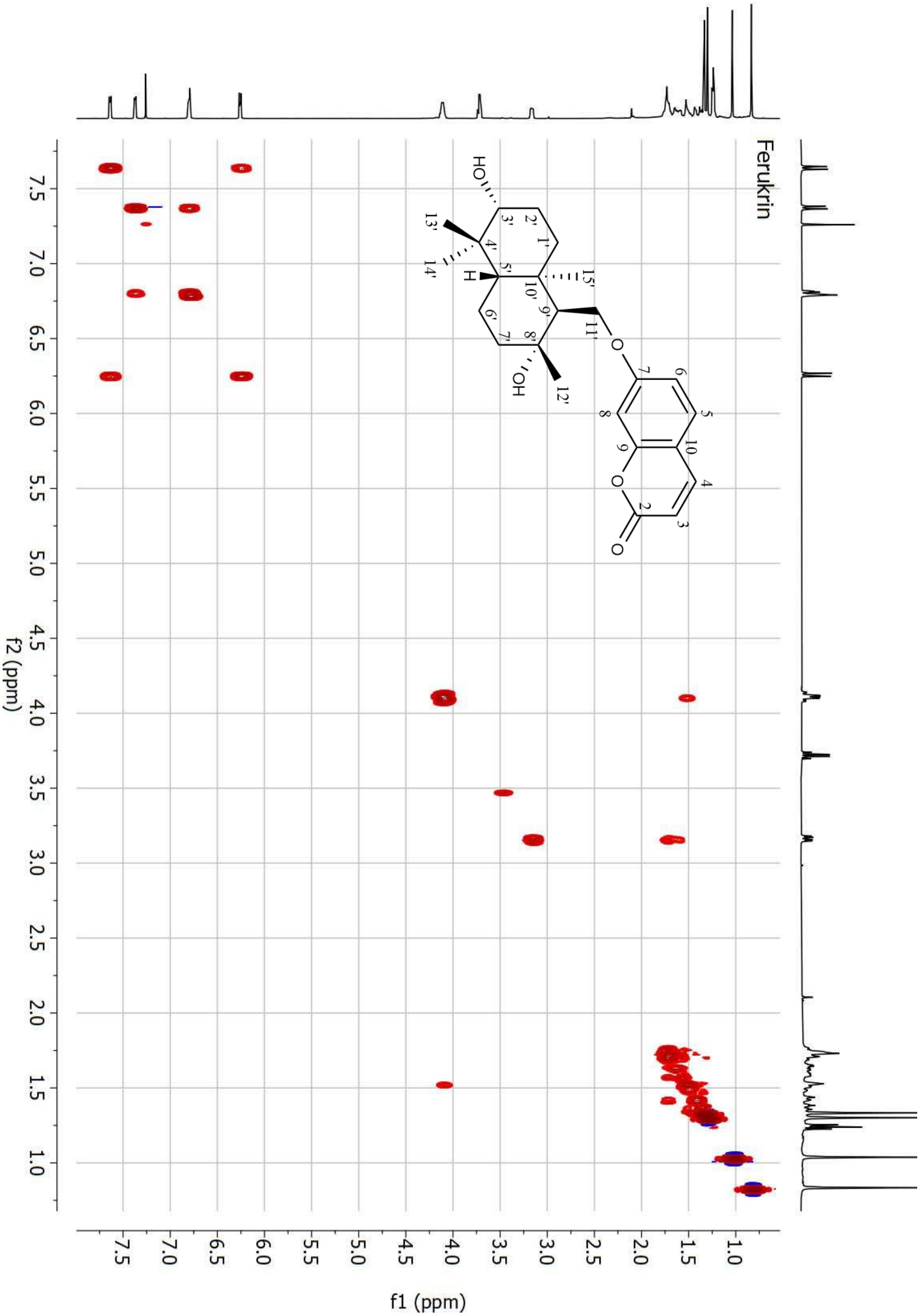

Figure S52. ^{1}H-^{1}H COSY spectrum ($CDCl_3$) of ferukrin (**9**)

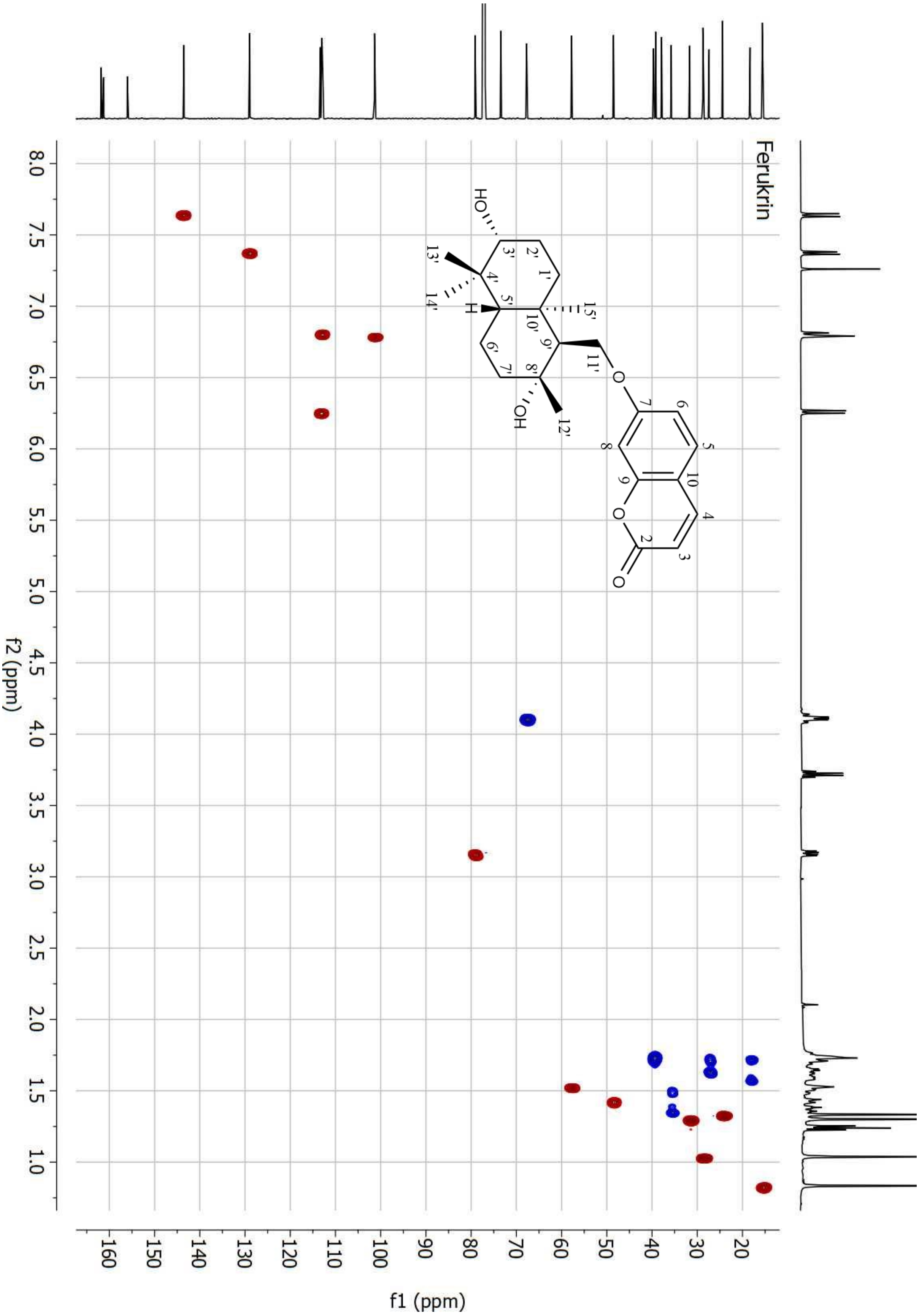

Figure S53. HSQC spectrum ($CDCl_3$) of ferukrin (**9**)

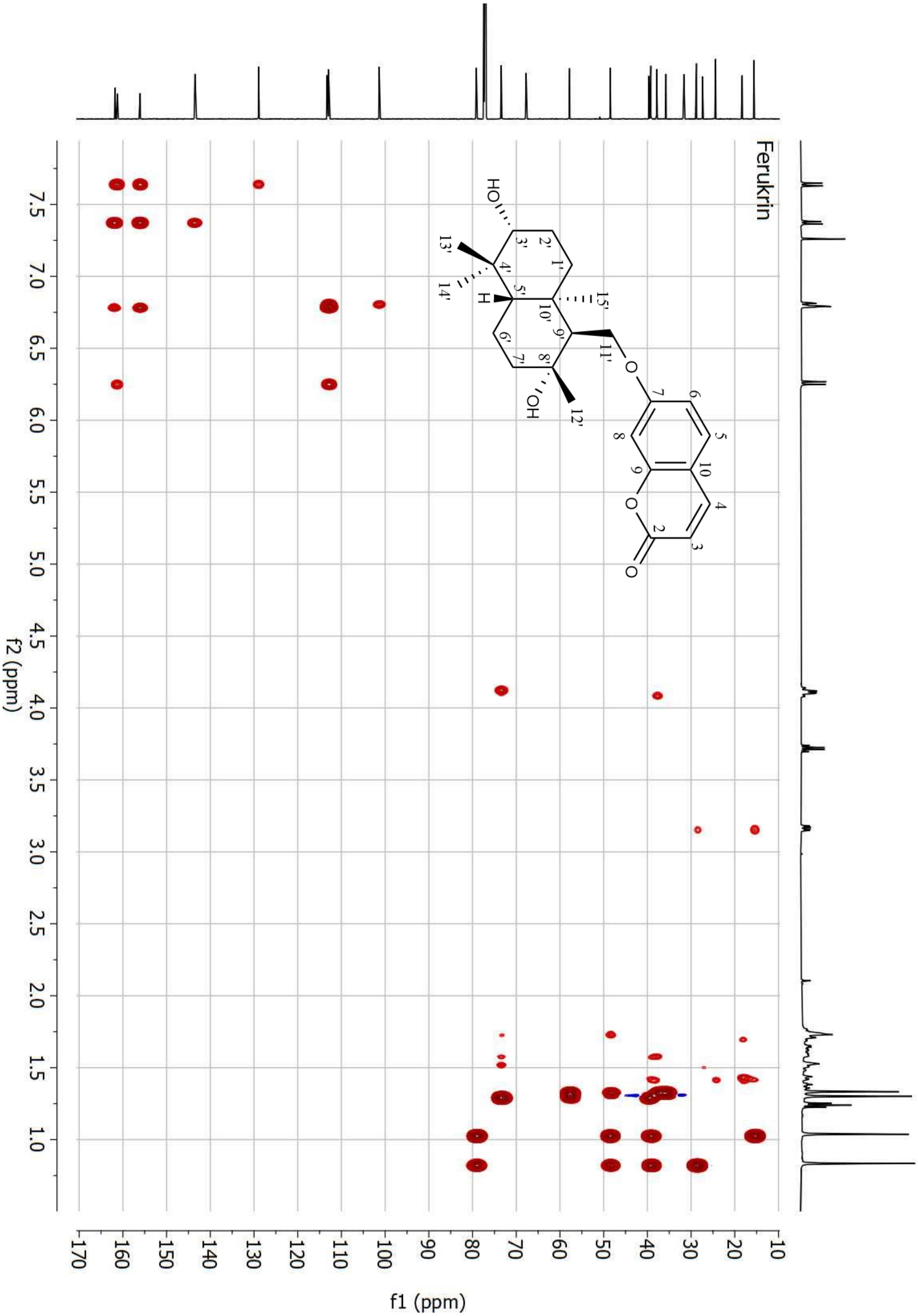

Figure S54. HMBC spectrum ($CDCl_3$) of ferukrin (**9**)

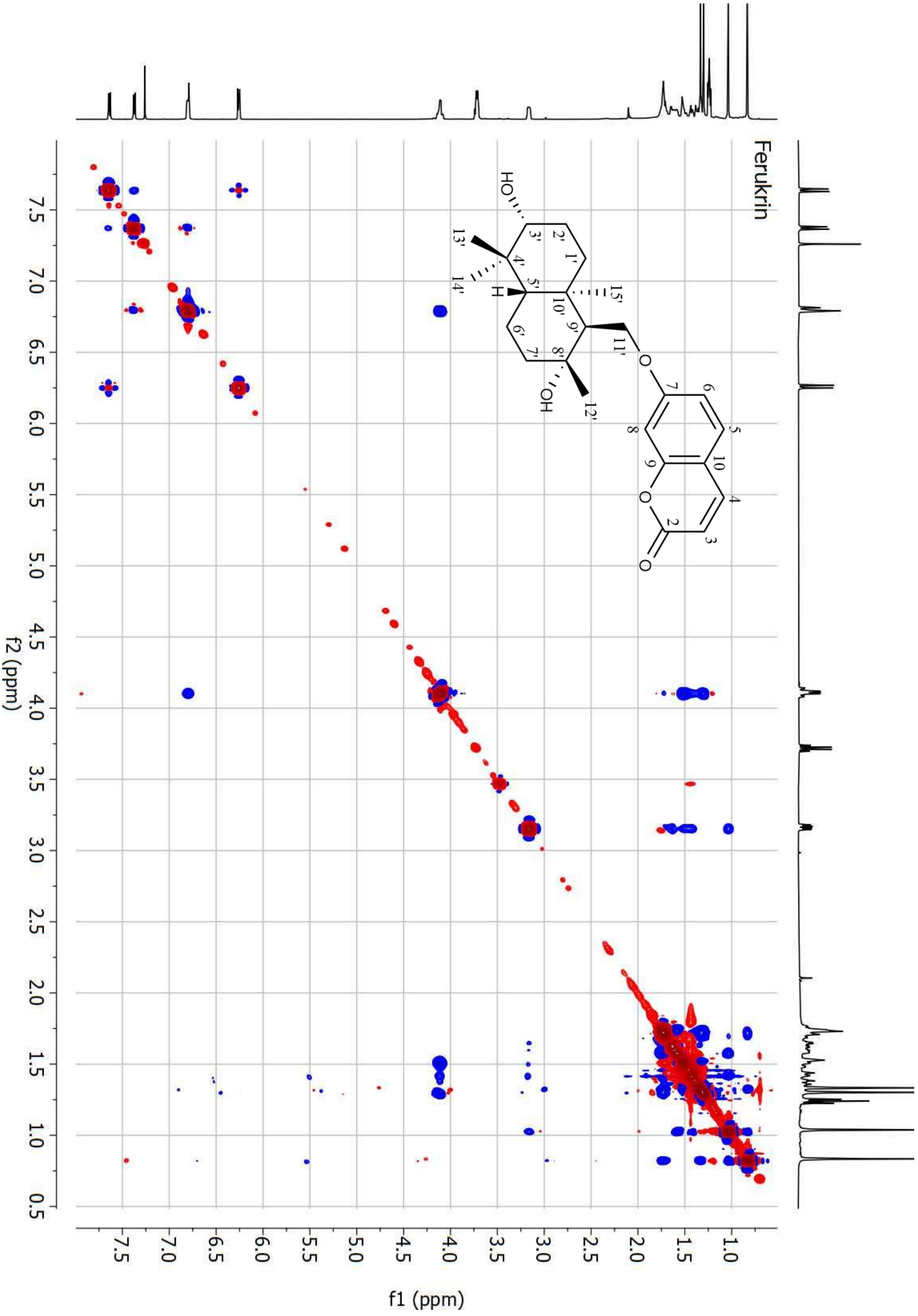

Figure S55. NOESY spectrum ($CDCl_3$) of ferukrin (**9**)

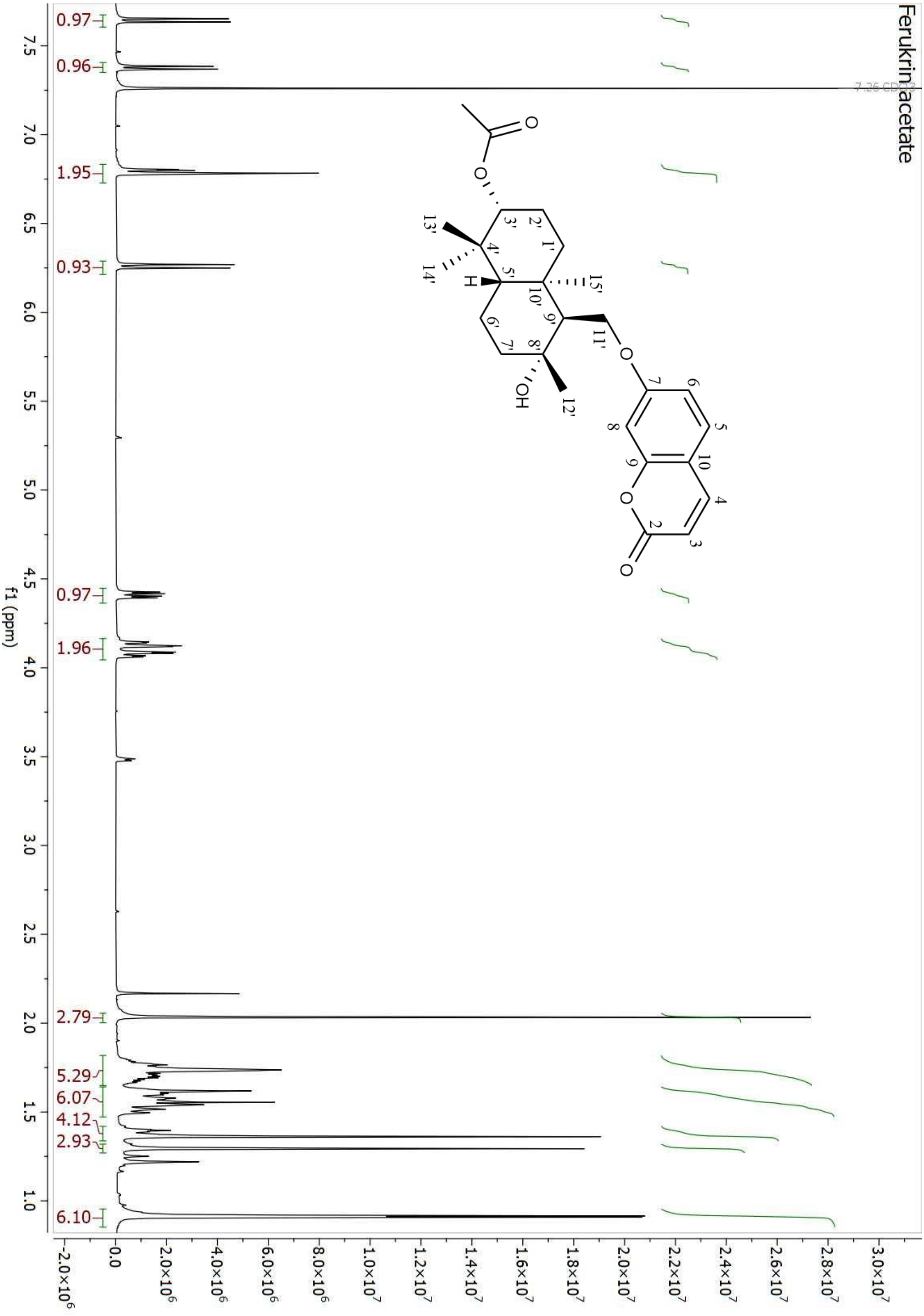

Figure S56. ^{1}H-NMR spectrum (500 MHz, $CDCl_3$) of ferukrin acetate (**10**)

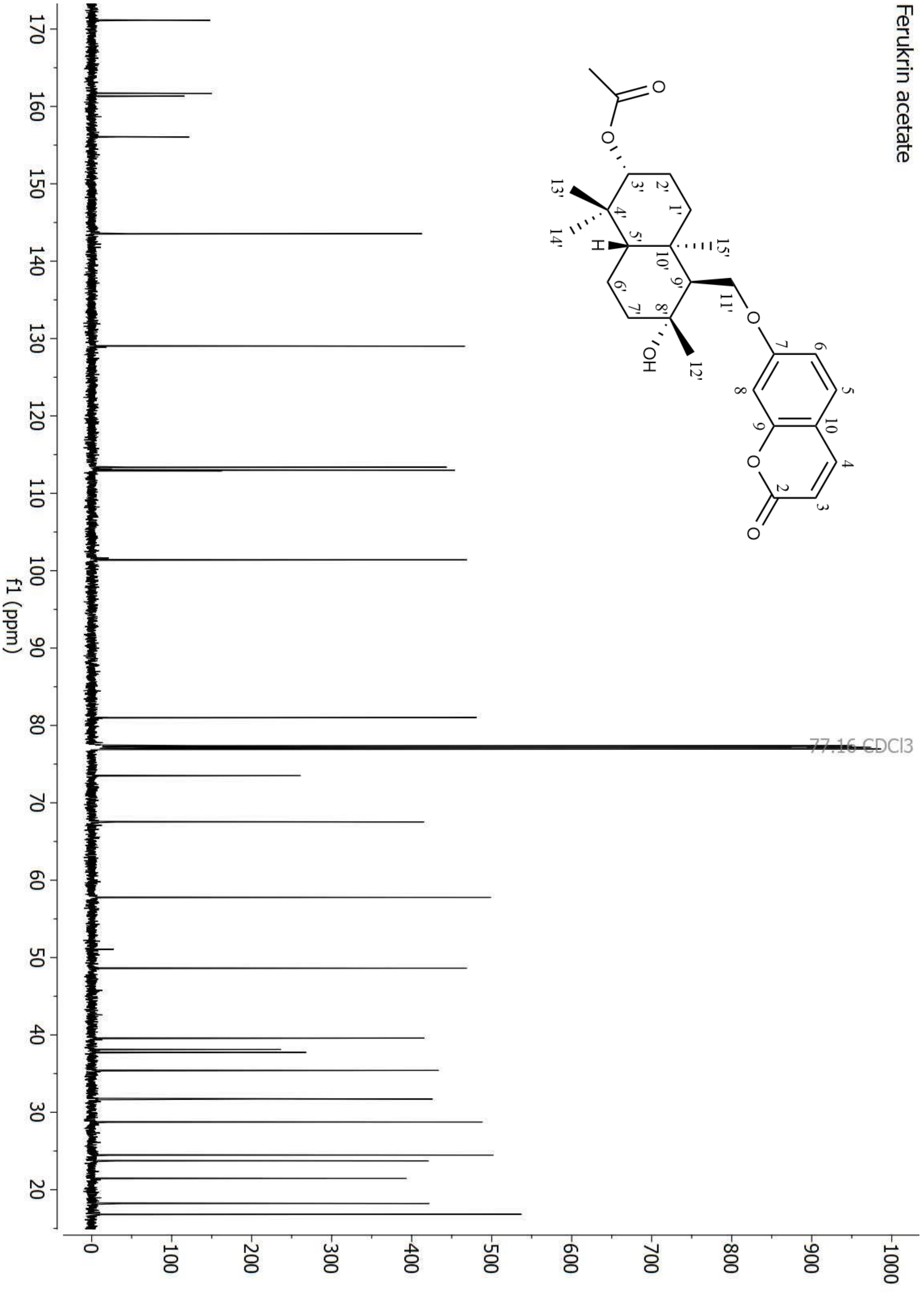

Figure S57. ^{13}C-NMR spectrum (125 MHz, $CDCl_3$) of ferukrin acetate (**10**)

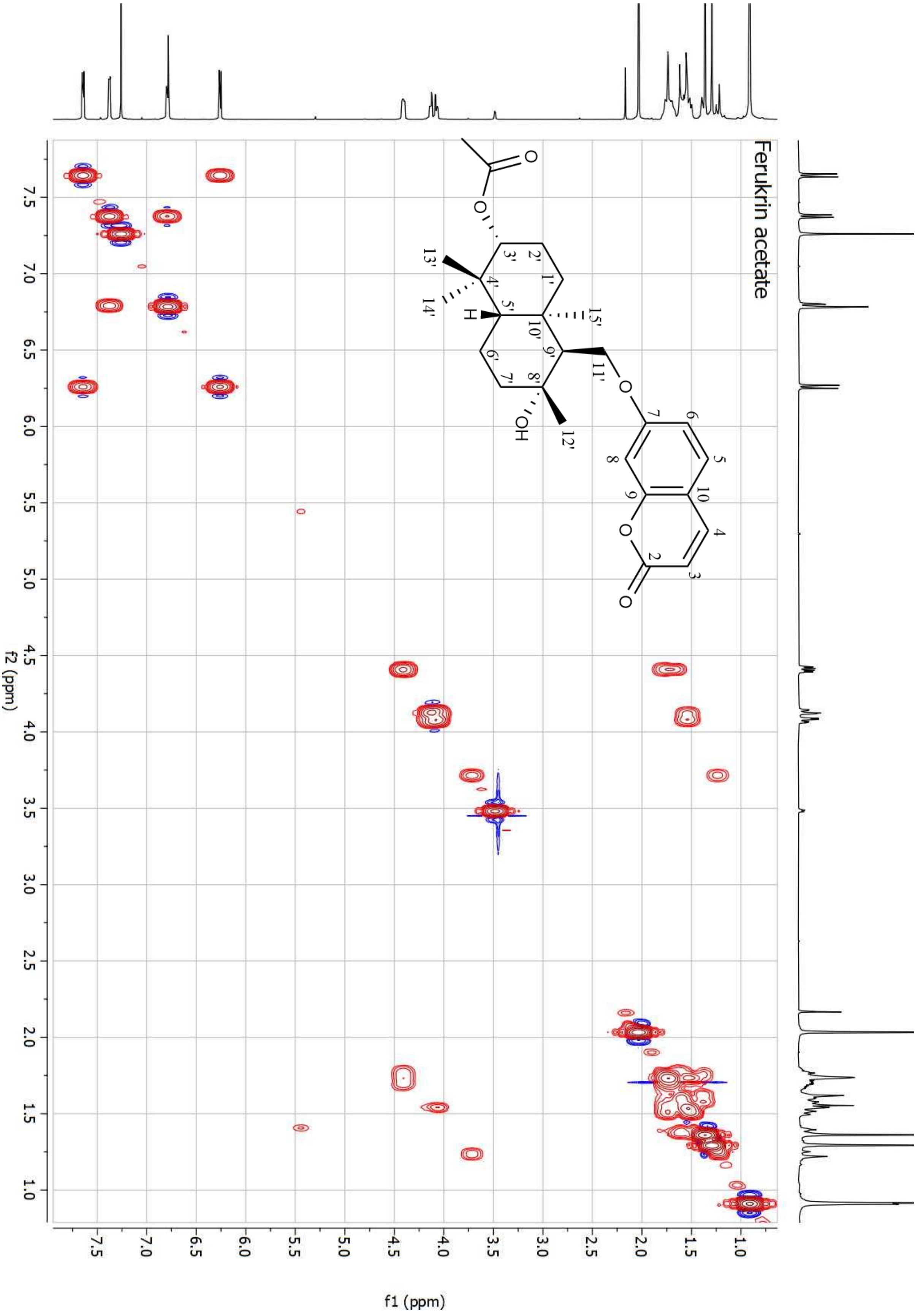

Figure S58. ^{1}H-^{1}H COSY spectrum ($CDCl_3$) of ferukrin acetate (**10**)

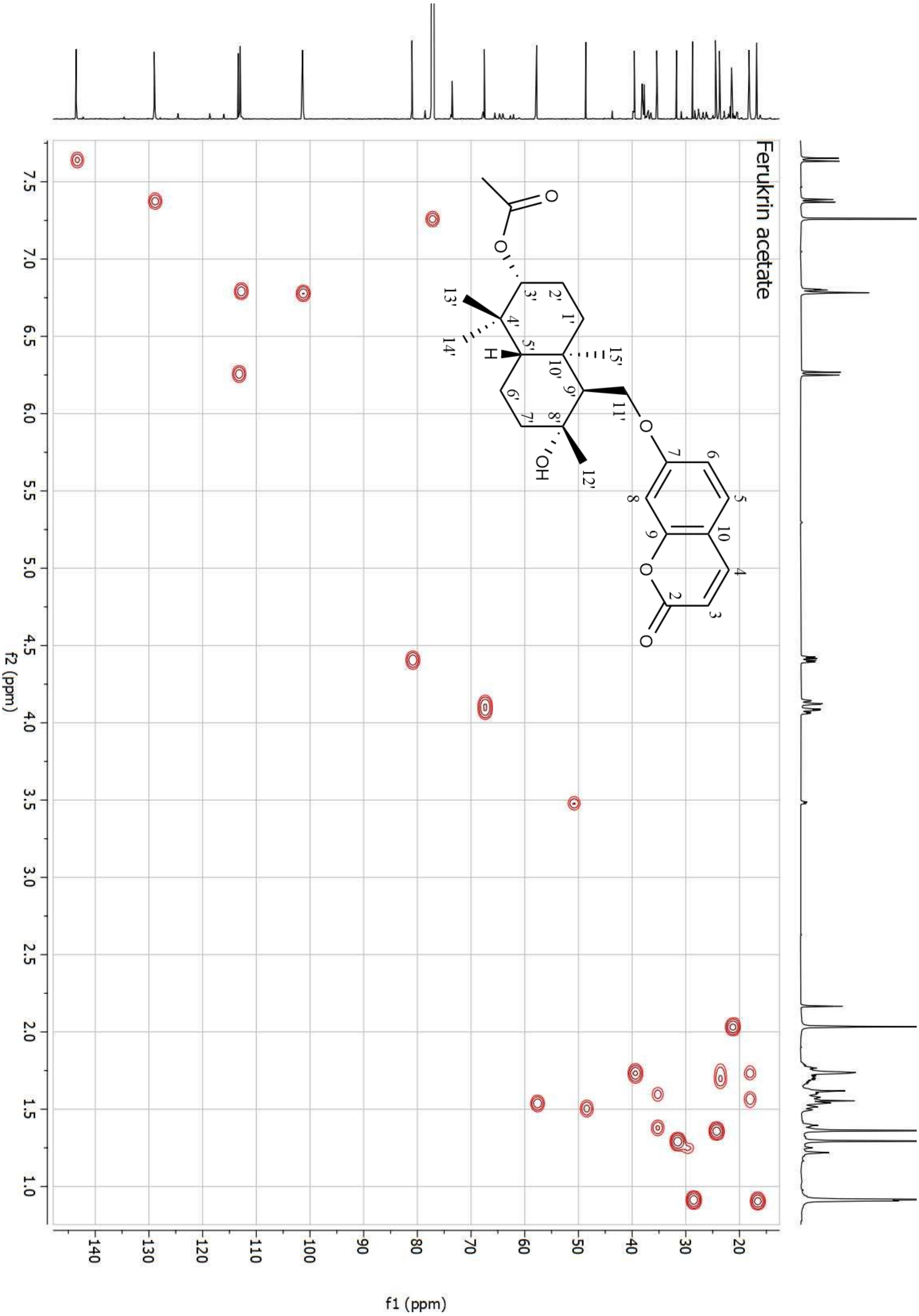

Figure S59. HSQC spectrum ($CDCl_3$) of ferukrin acetate (**10**)

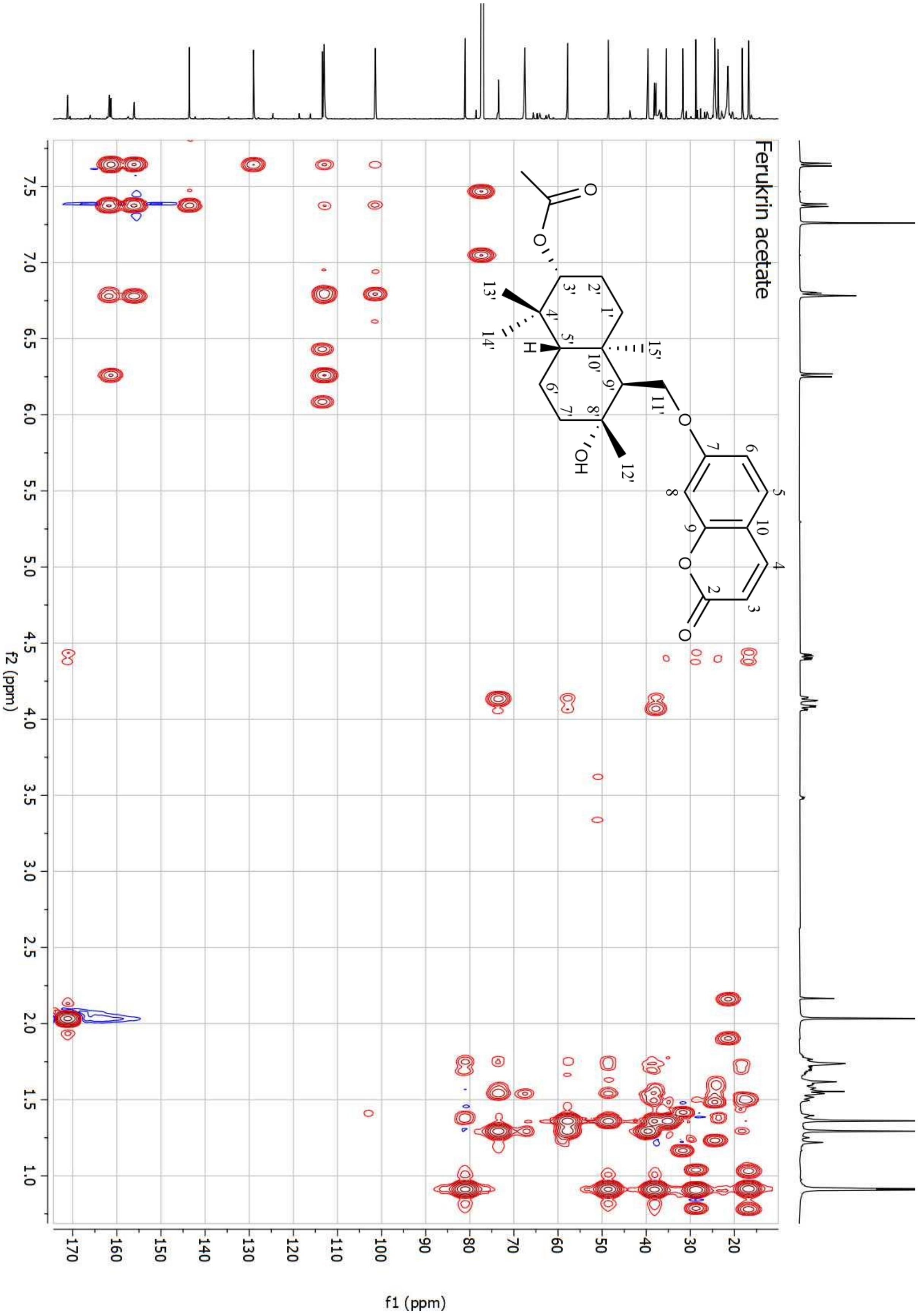

Figure S60. HMBC spectrum ($CDCl_3$) of ferukrin acetate (**10**)

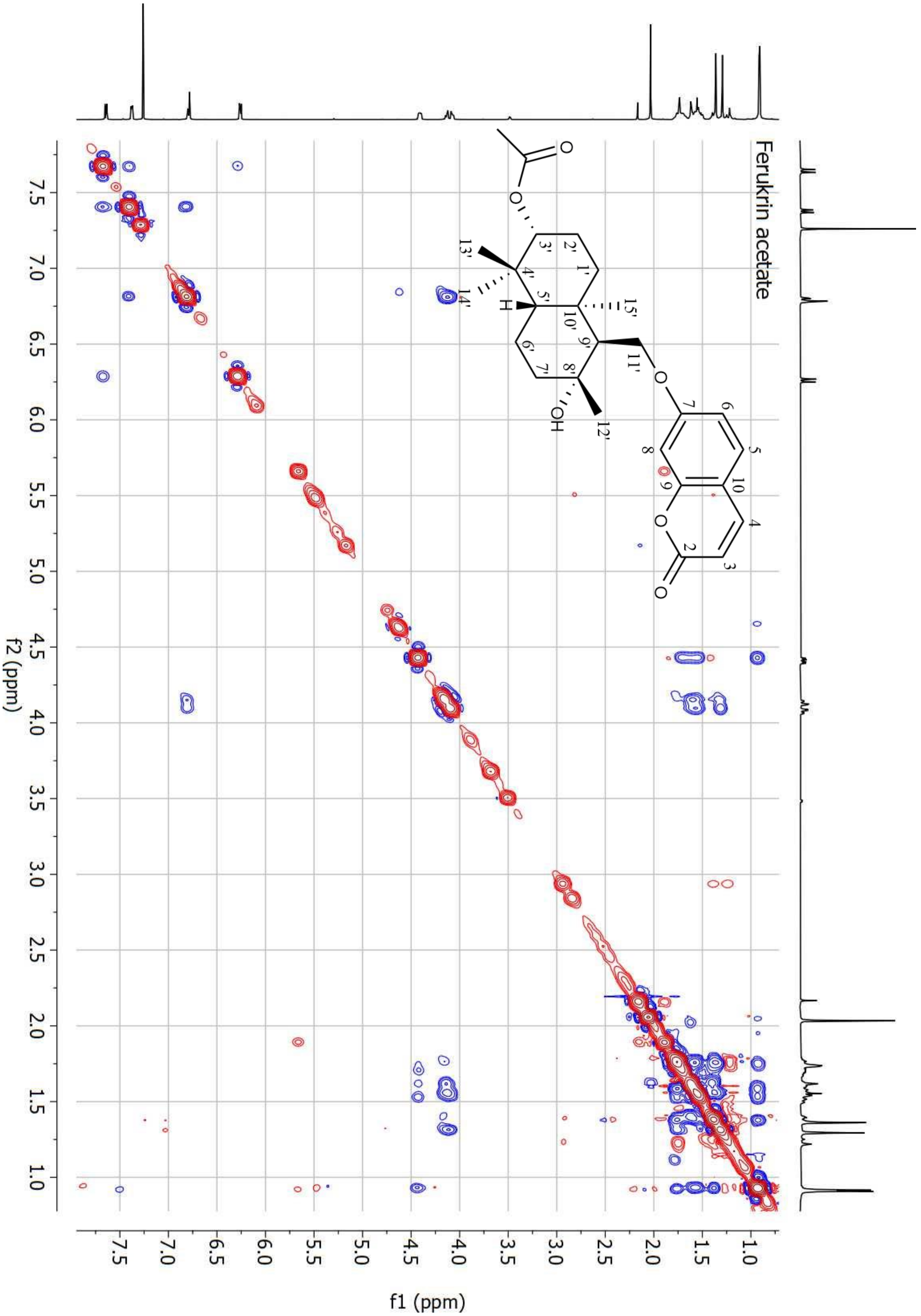

Figure S61. NOESY spectrum ($CDCl_3$) of ferukrin acetate (**10**)

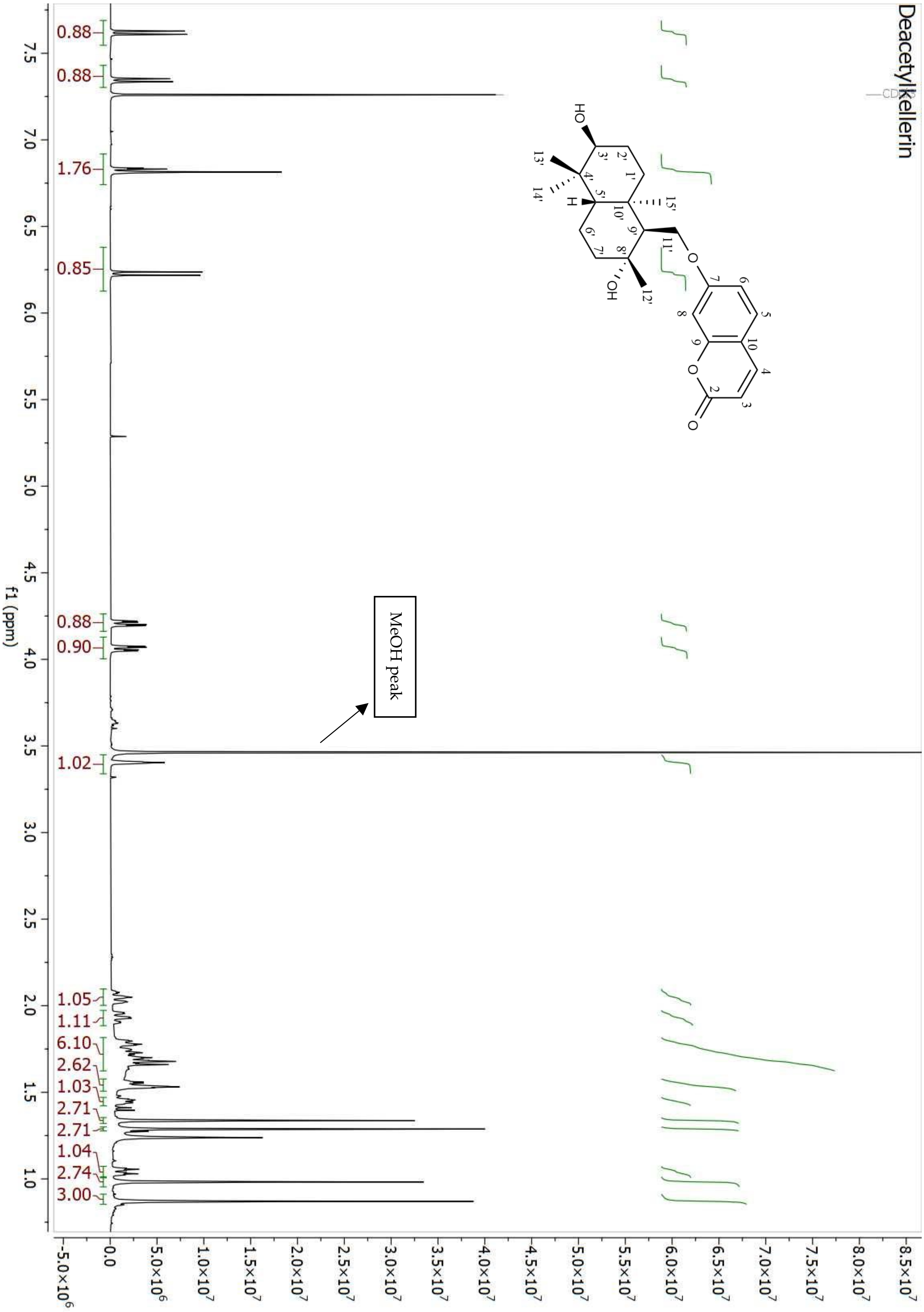

Figure S62. ^{1}H-NMR spectrum (500 MHz, $CDCl_3$) of deacetylkellerin (**11**)

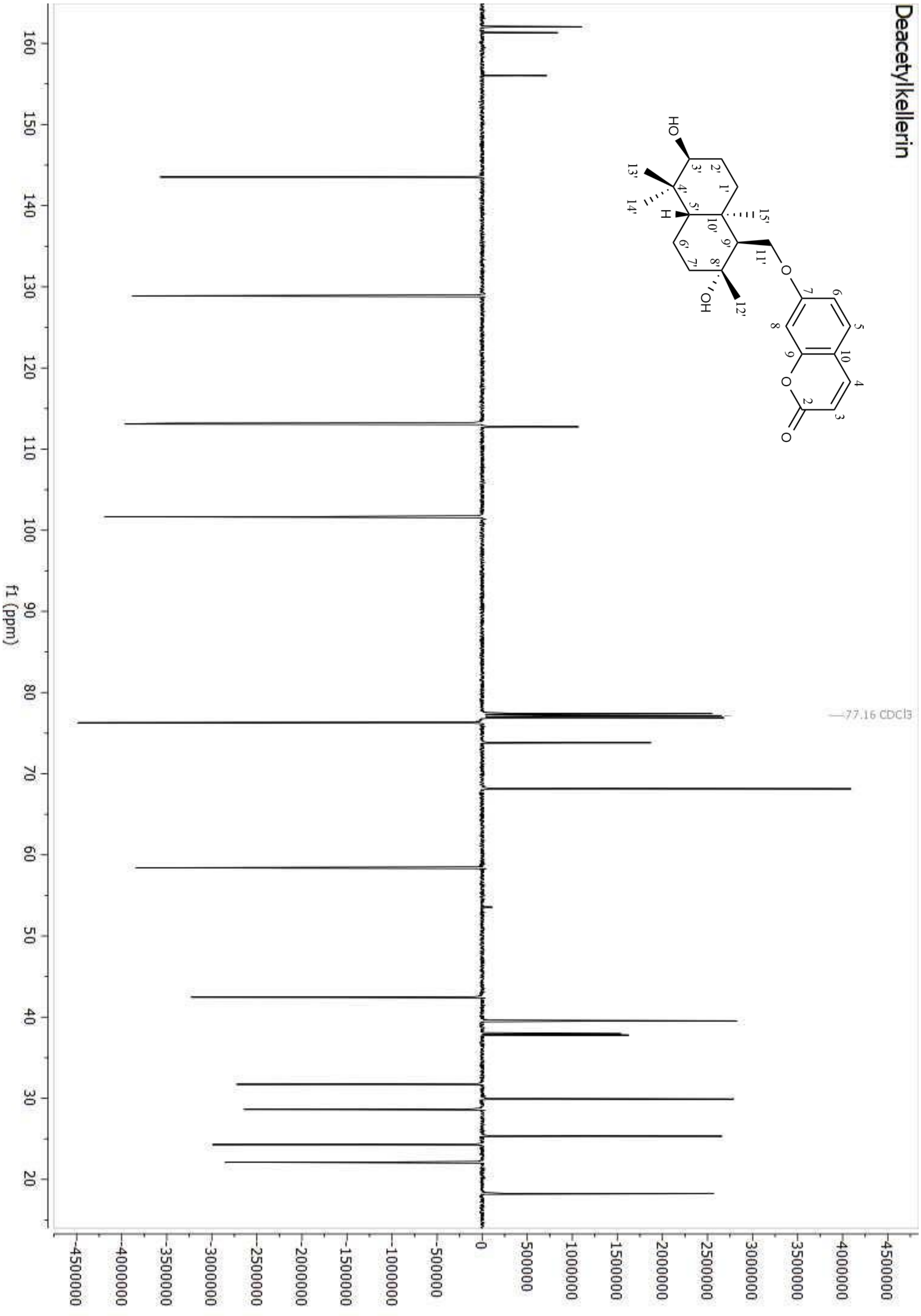

Figure S63. ^{13}C-NMR (APT) spectrum (125 MHz, $CDCl_3$) of deacetylkellerin (**11**)

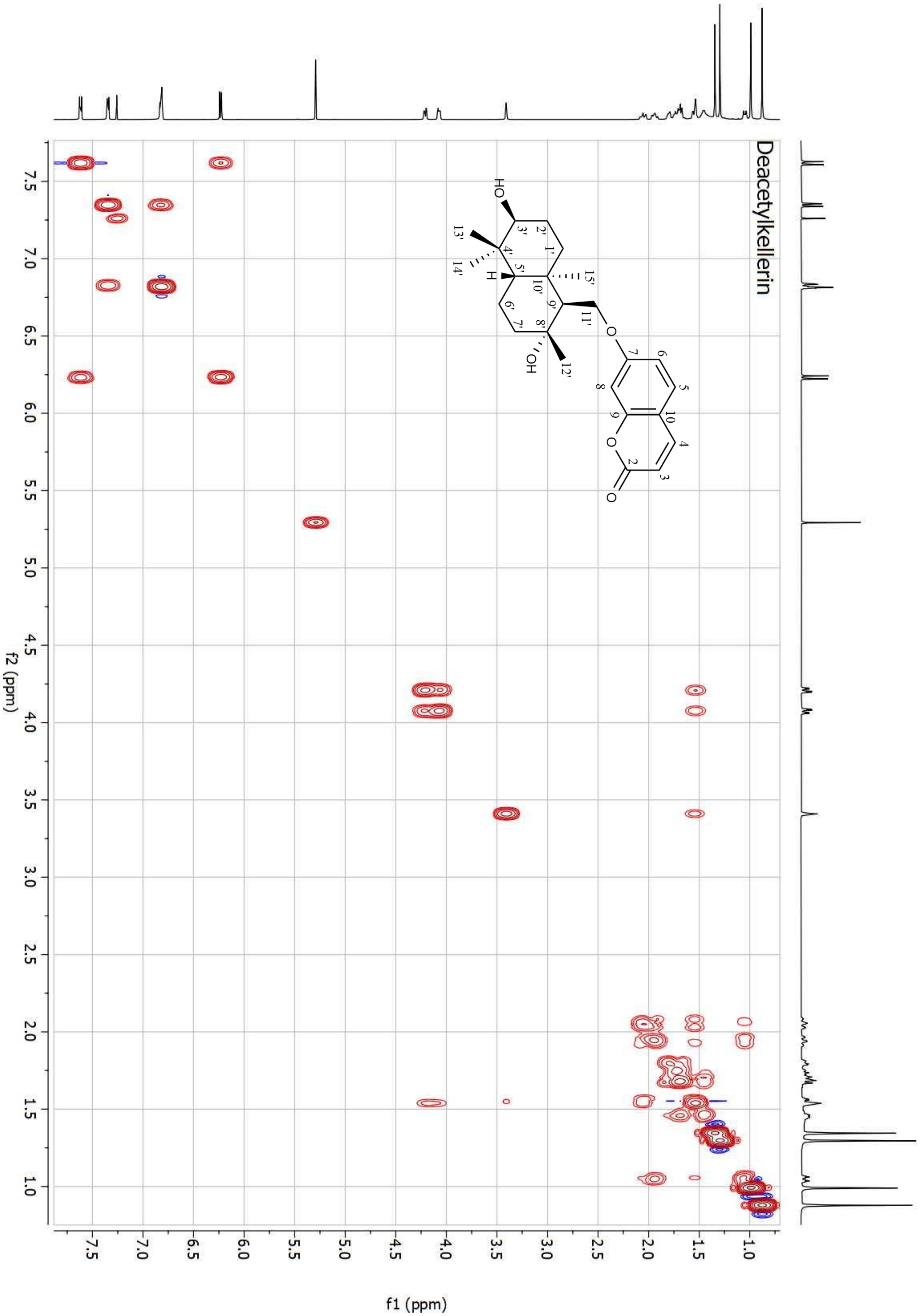

Figure S64. ^{1}H-^{1}H COSY spectrum ($CDCl_3$) of deacetylkellerin (**11**)

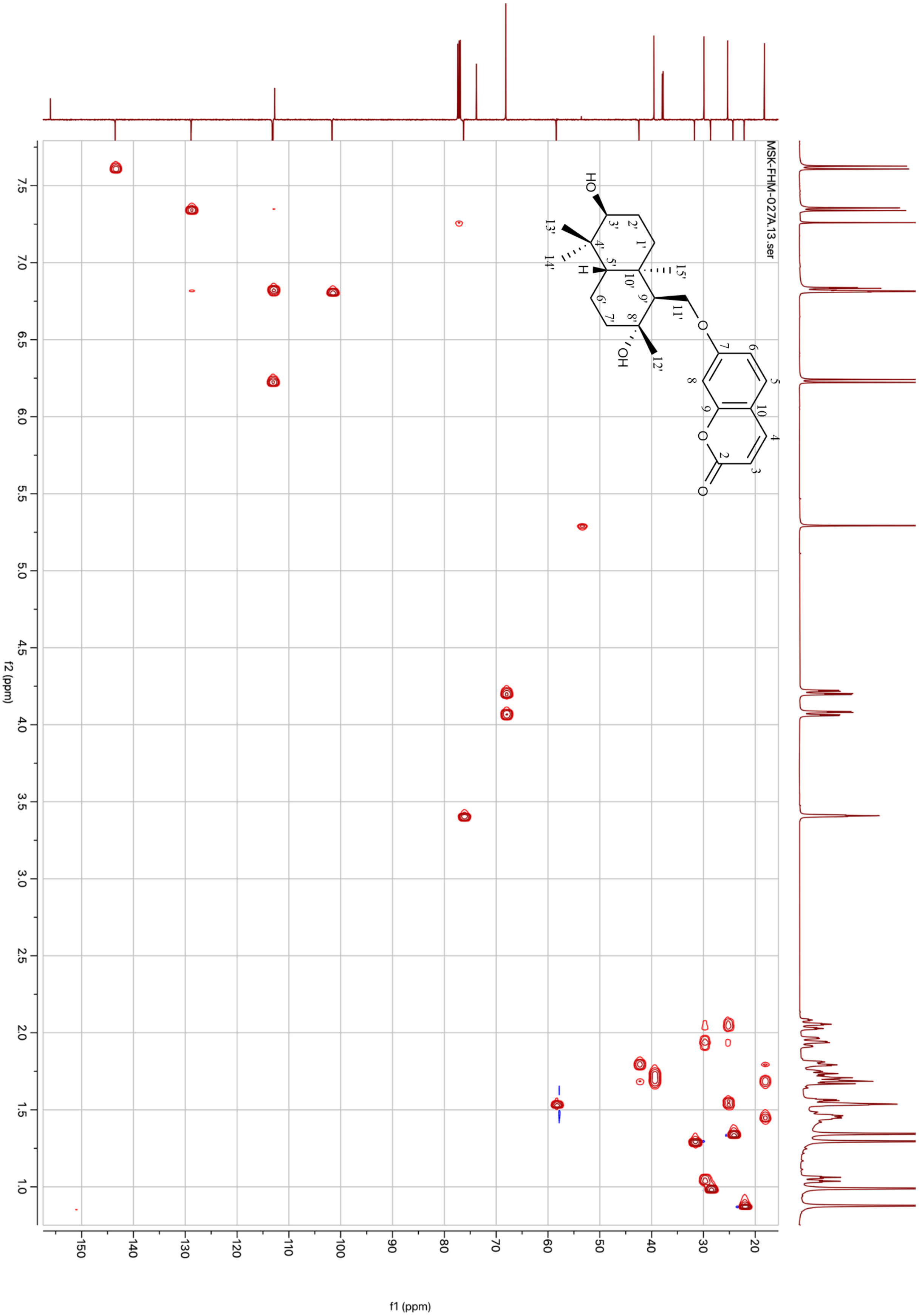

Figure S65. HSQC spectrum ($CDCl_3$) of deacetylkellerin (**11**)

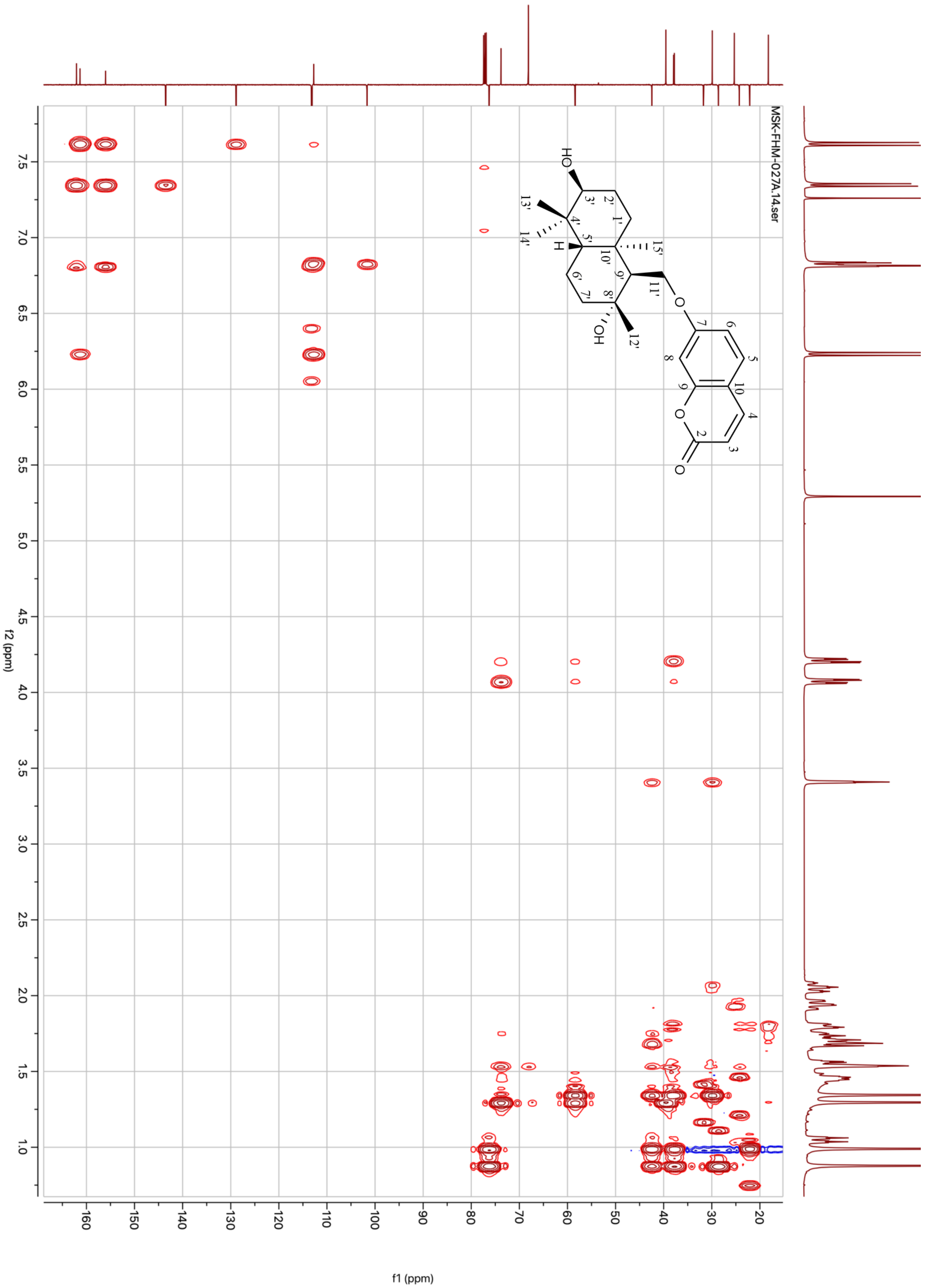

Figure S66. HMBC spectrum ($CDCl_3$) of deacetylkellerin (**11**)

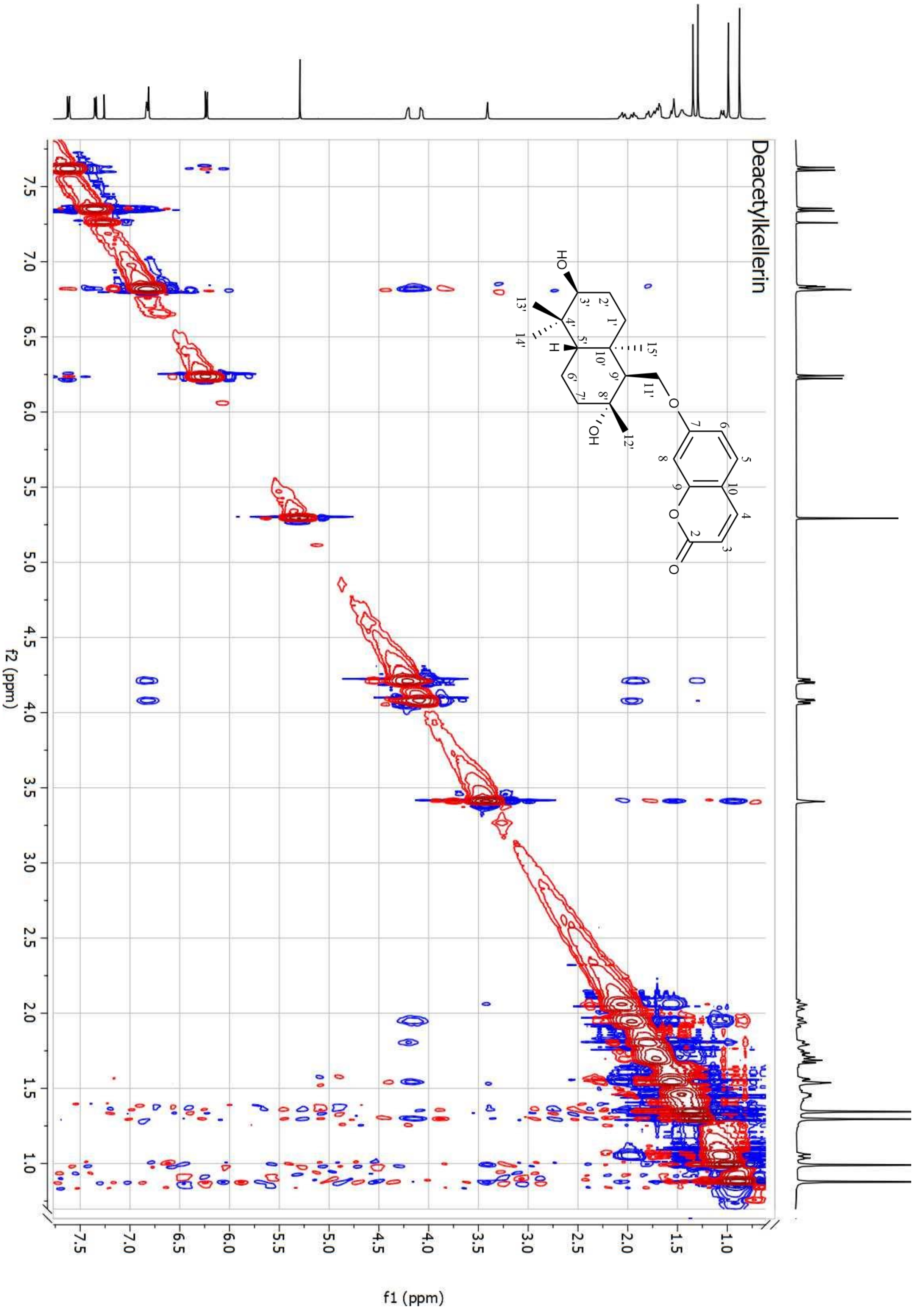

Figure S67. NOESY spectrum ($CDCl_3$) of deacetylkellerin (**11**)

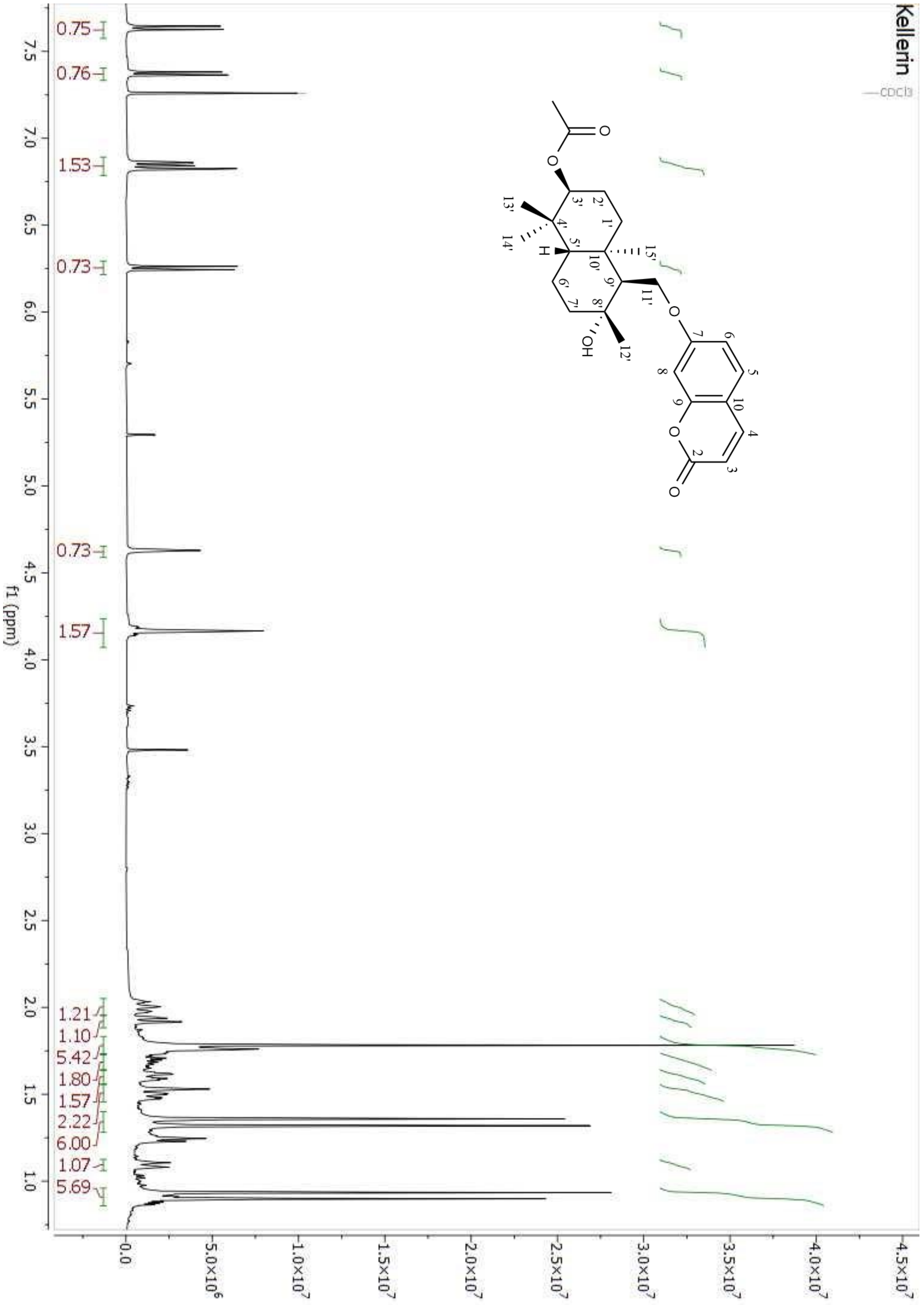

Figure S68. ^{1}H-NMR spectrum (500 MHz, $CDCl_3$) of kellerin (**12**)

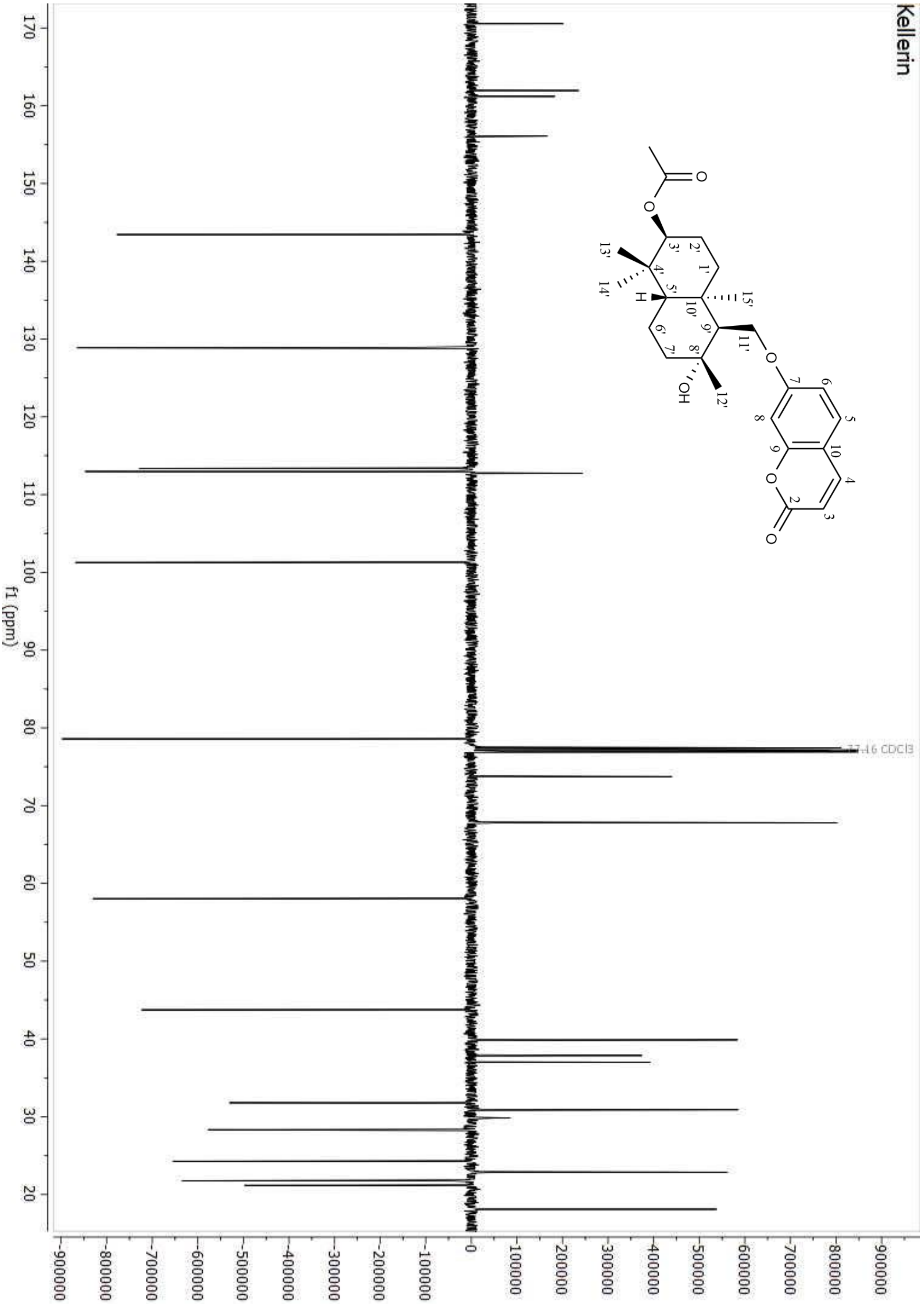

Figure S69. ^{13}C-NMR (APT) spectrum (125 MHz, $CDCl_3$) of kellerin (**12**)

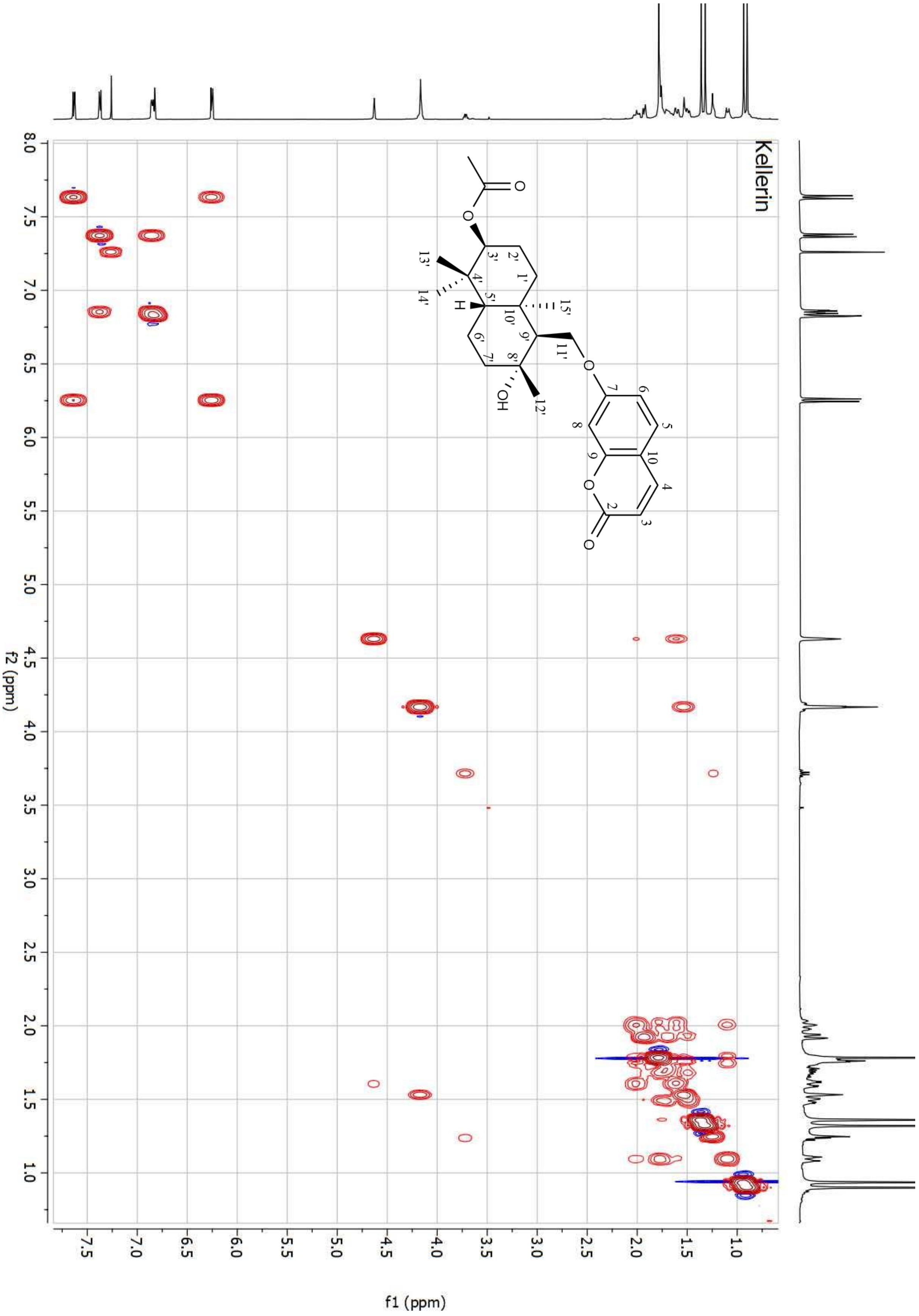

Figure S70. ^{1}H-^{1}H COSY spectrum ($CDCl_3$) of kellerin (**12**)

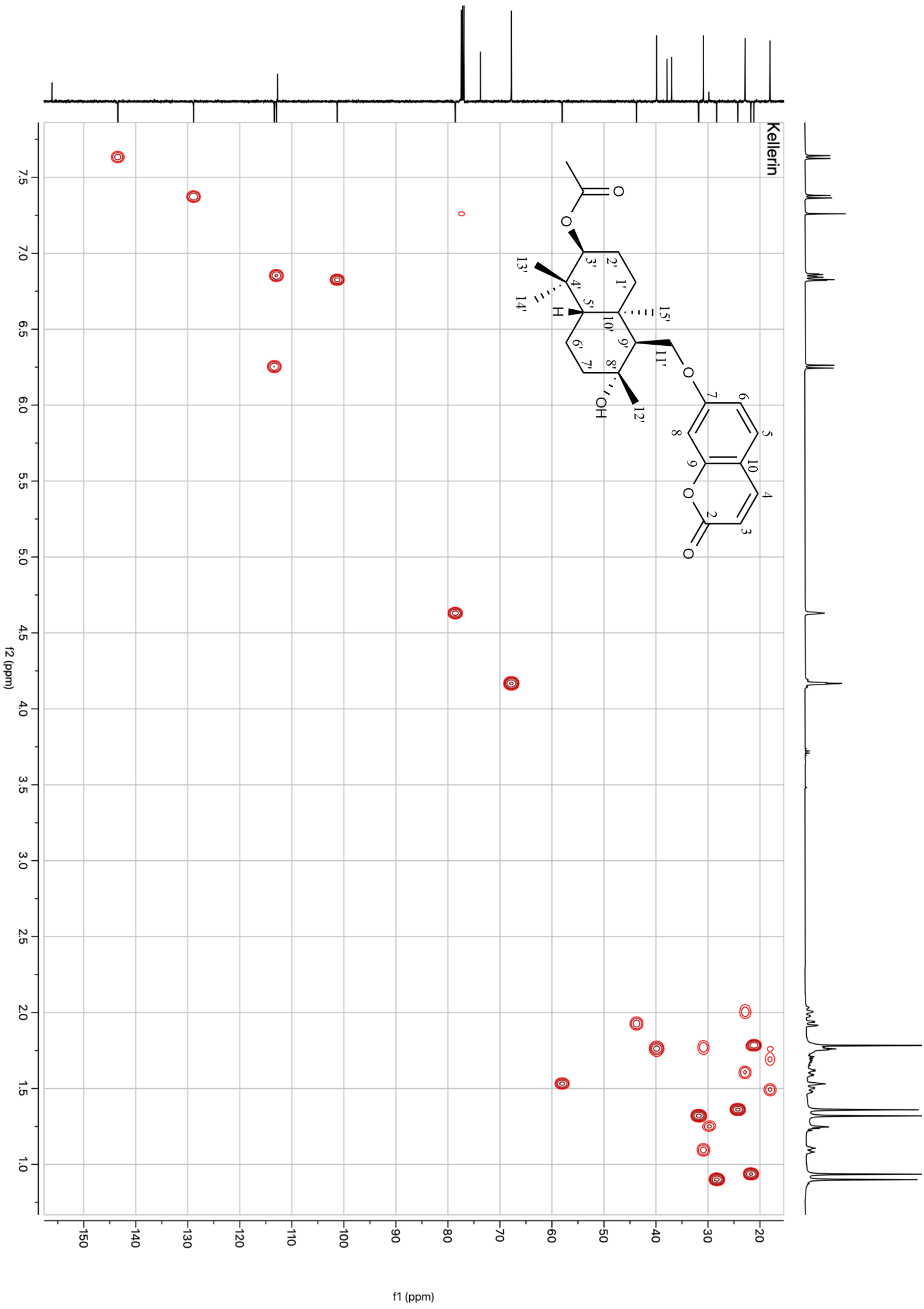

Figure S71. HSQC spectrum ($CDCl_3$) of kellerin (**12**)

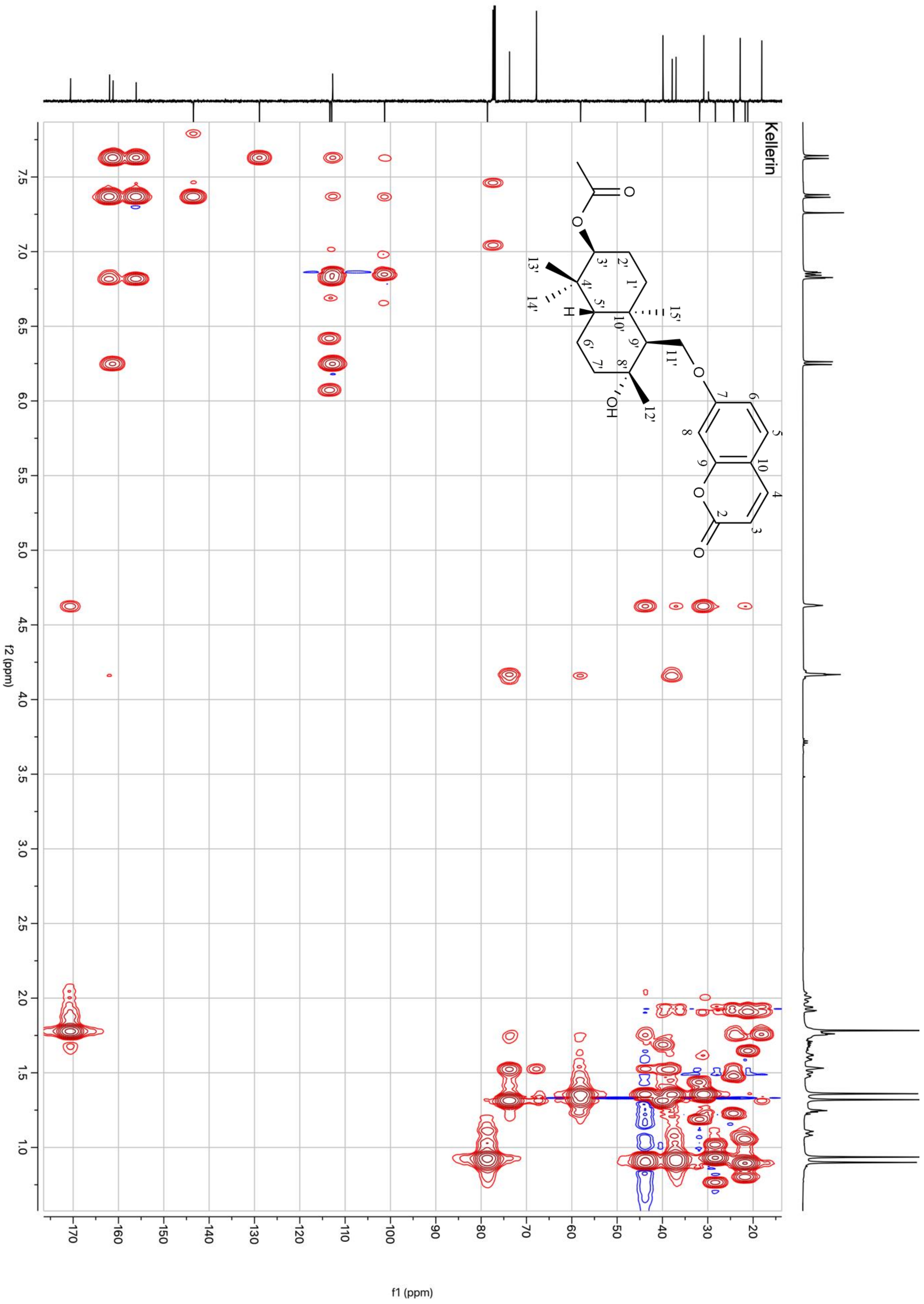

Figure S72. HMBC spectrum ($CDCl_3$) of kellerin (**12**)

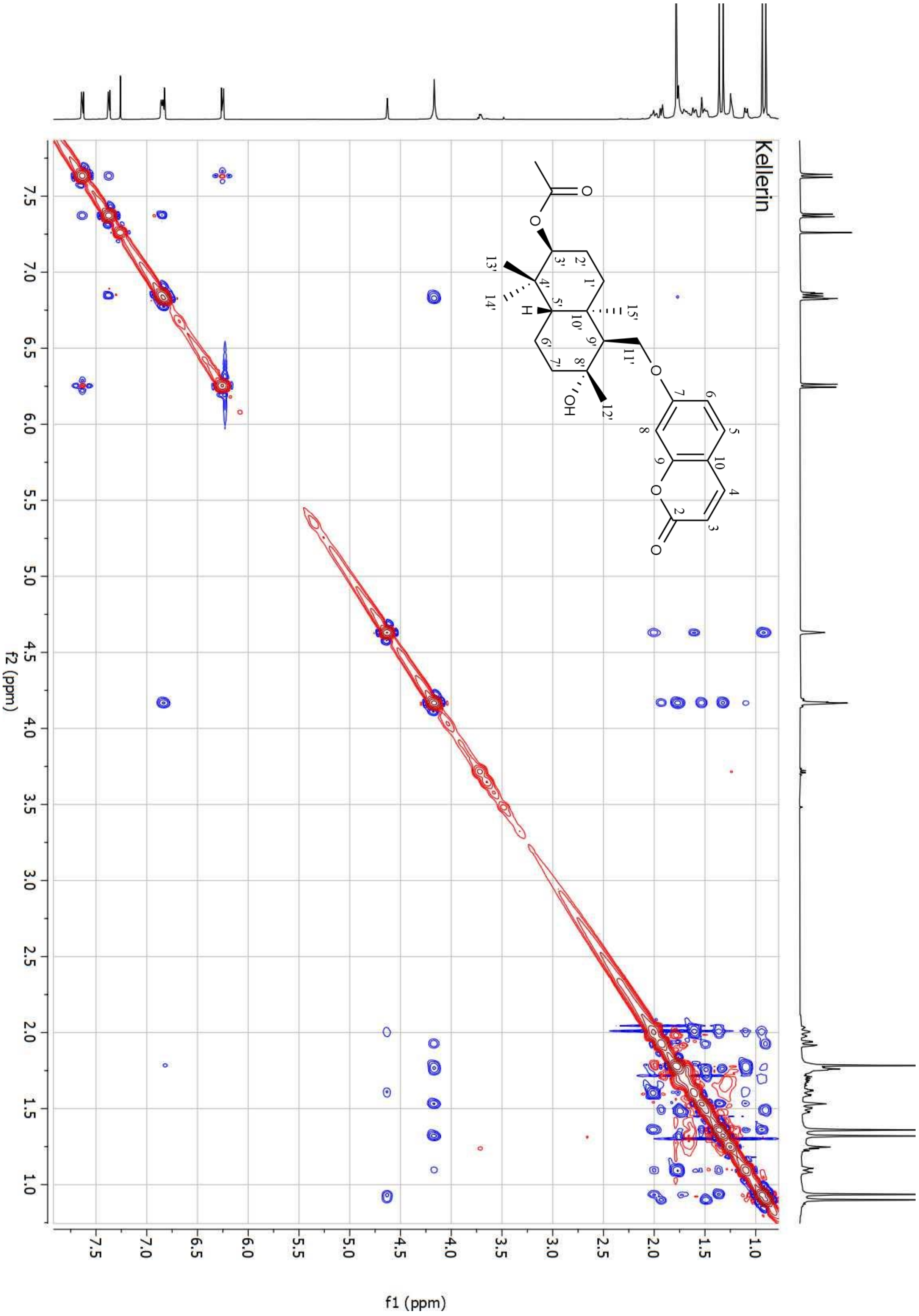

Figure S73. NOESY spectrum ($CDCl_3$) of kellerin (**12**)

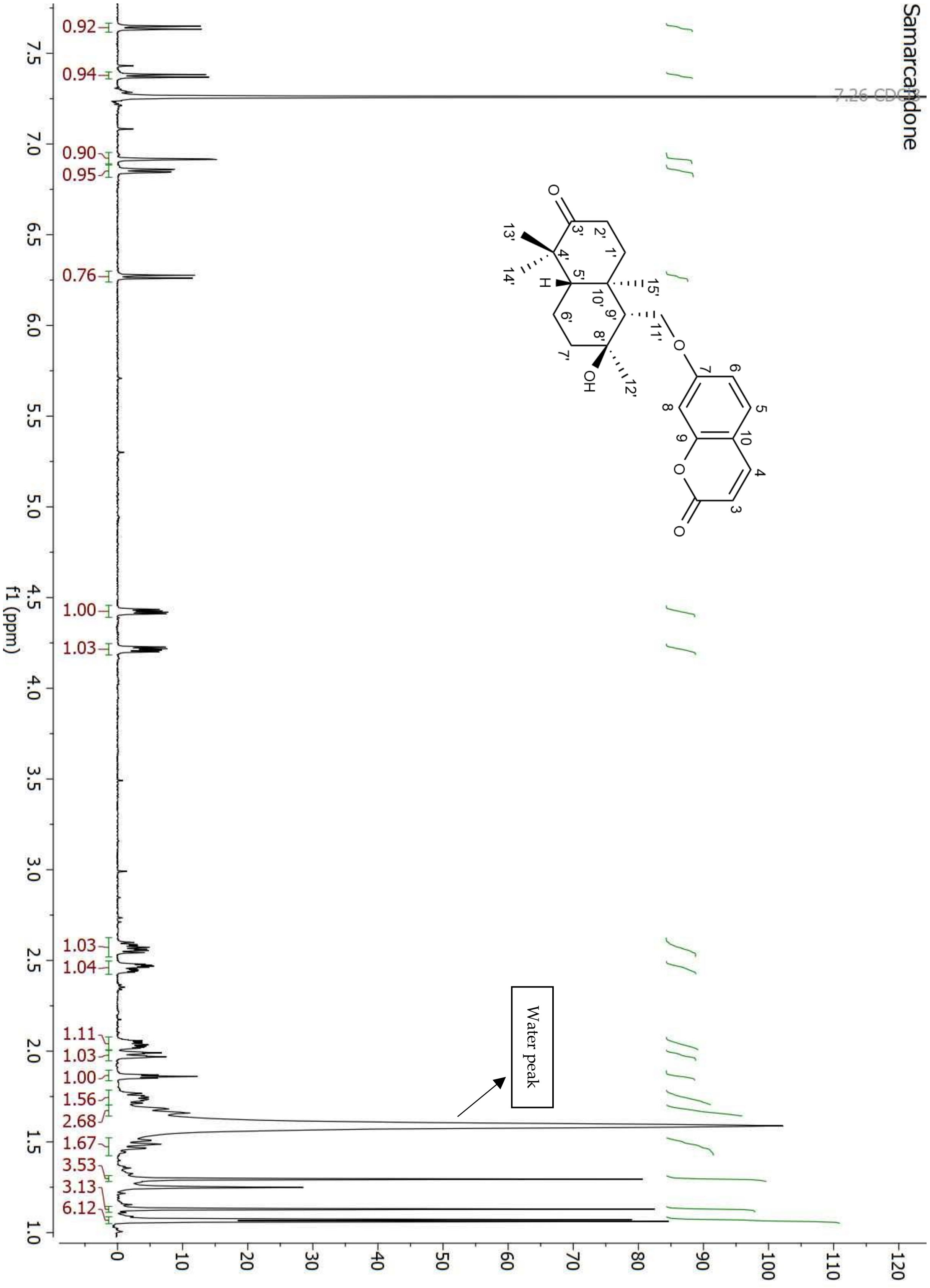

Figure S74. ^{1}H-NMR spectrum (500 MHz, $CDCl_3$) of samarcandone (**13**)

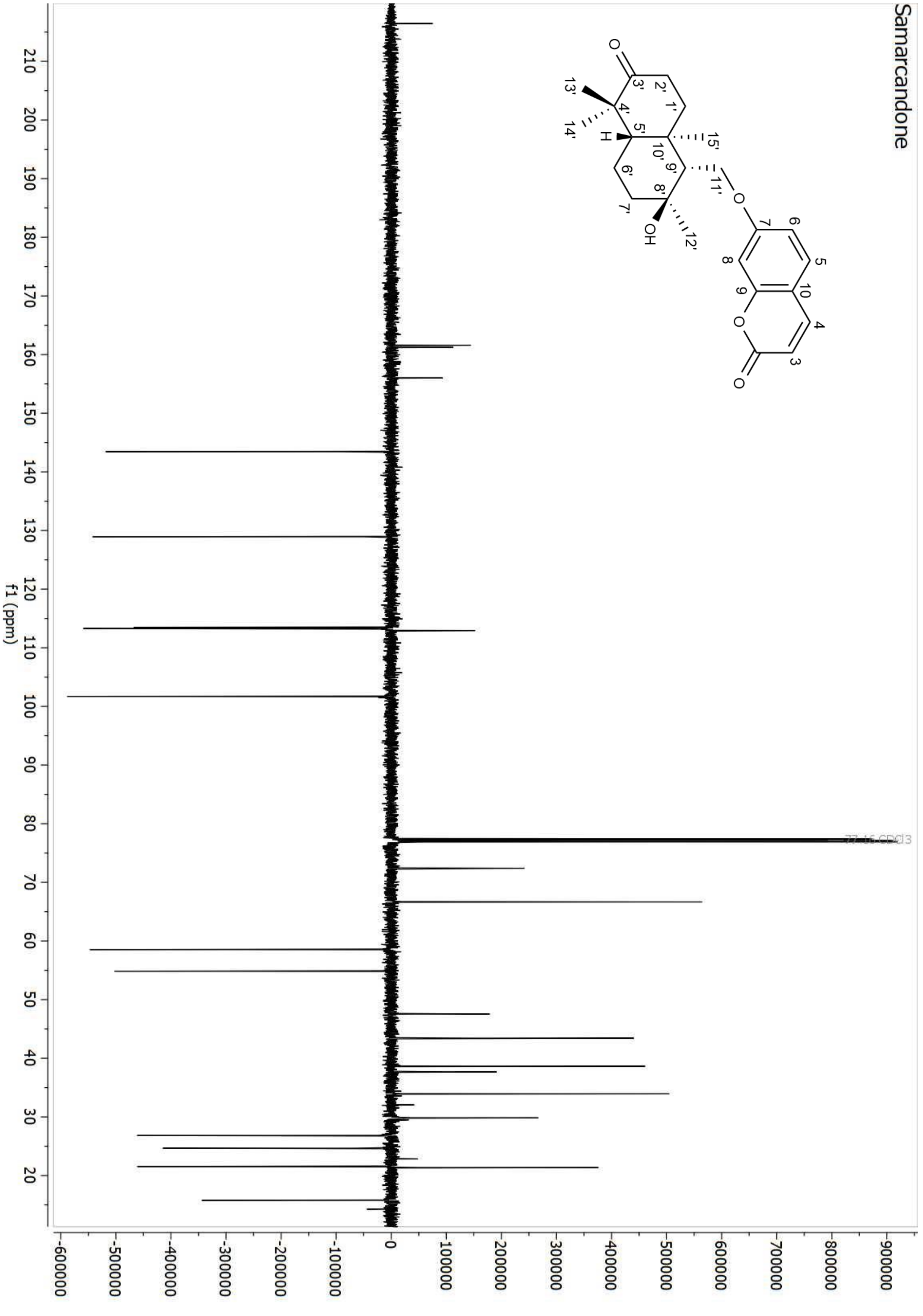

Figure S75. ^{13}C-NMR (APT) spectrum (125 MHz, $CDCl_3$) of samarcandone (**13**)

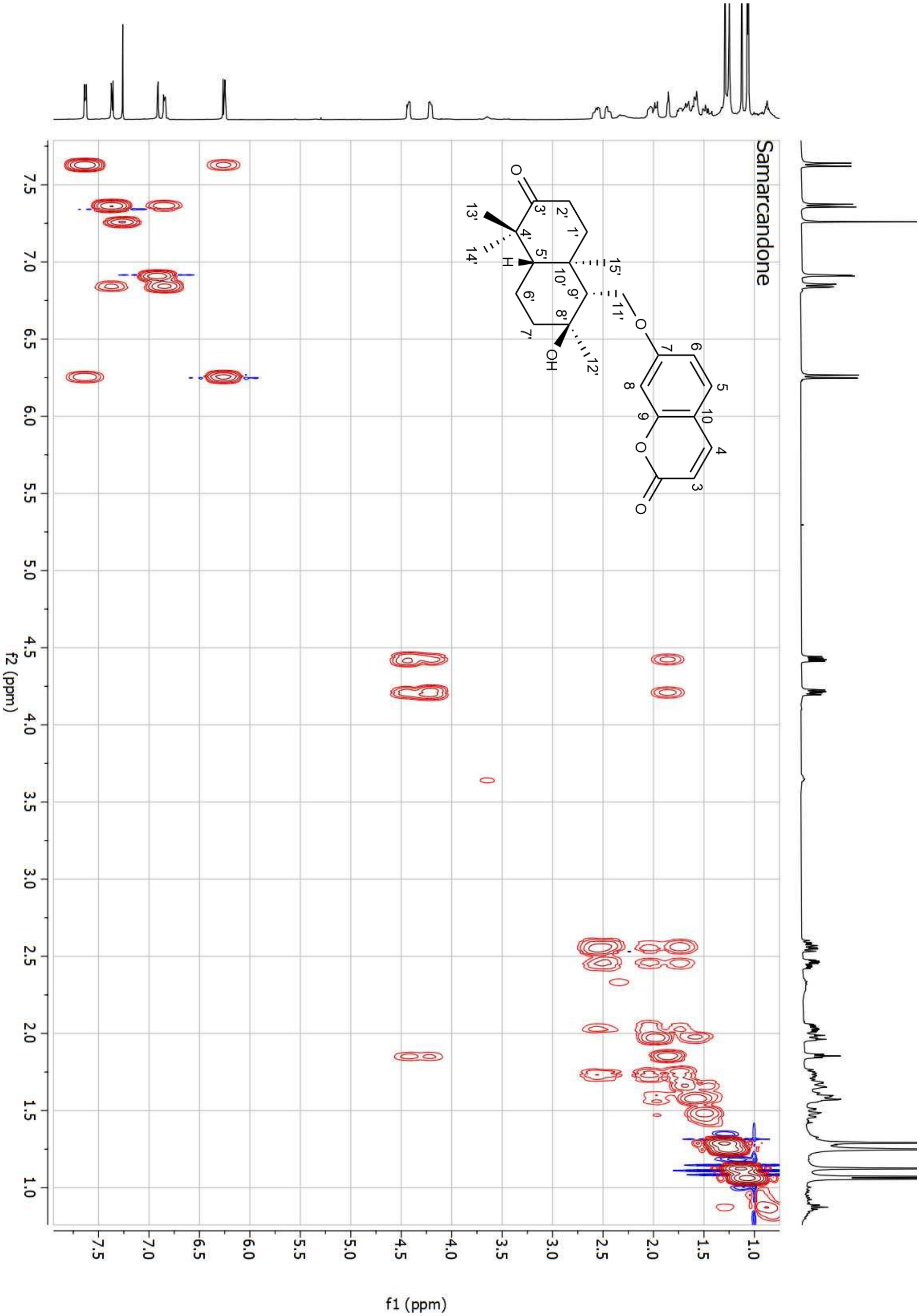

Figure S76. ^{1}H-^{1}H COSY spectrum ($CDCl_3$) of samarcandone (**13**)

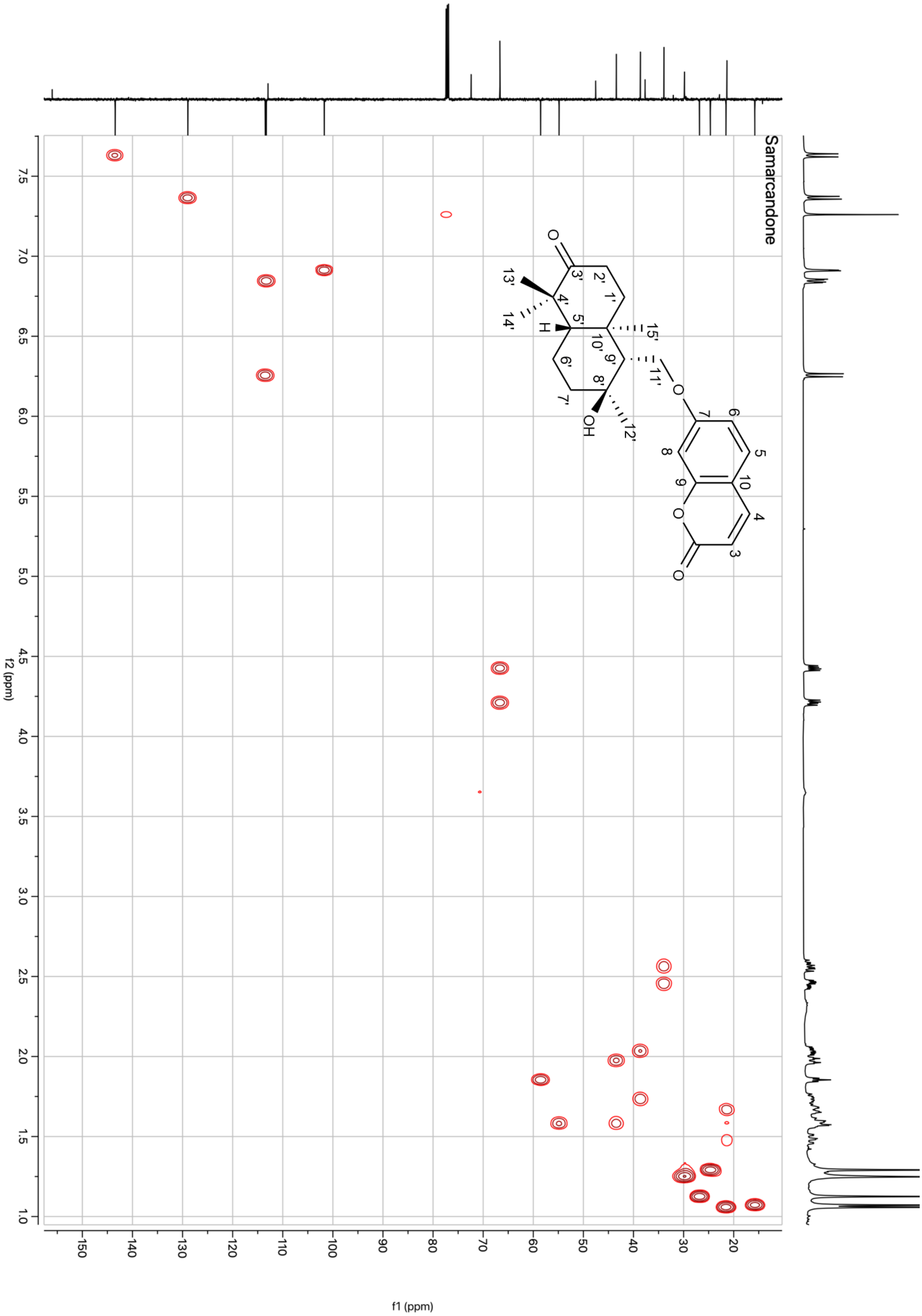

Figure S77. HSQC spectrum ($CDCl_3$) of samarcandone (**13**)

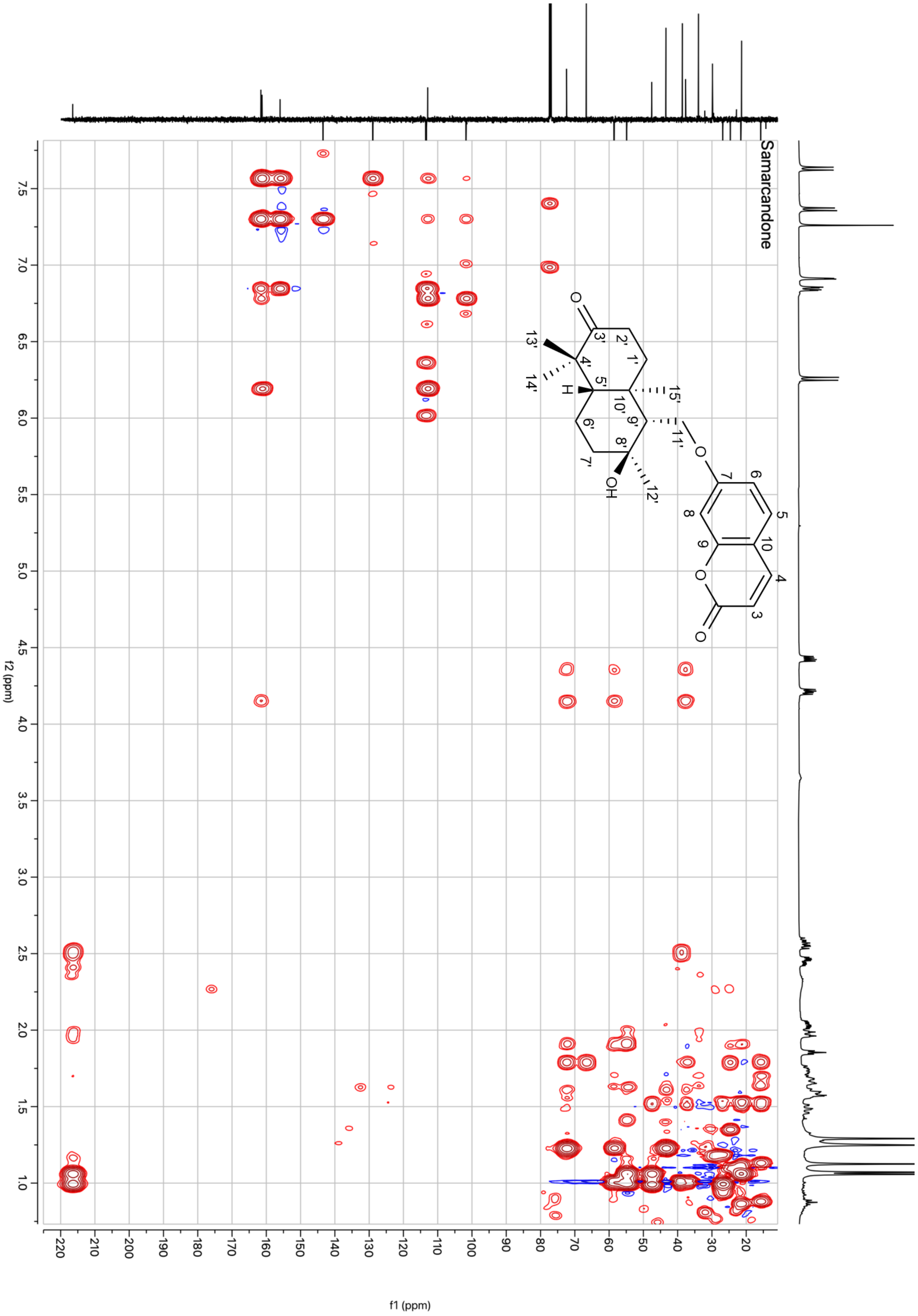

Figure S78. HMBC spectrum ($CDCl_3$) of samarcandone (**13**)

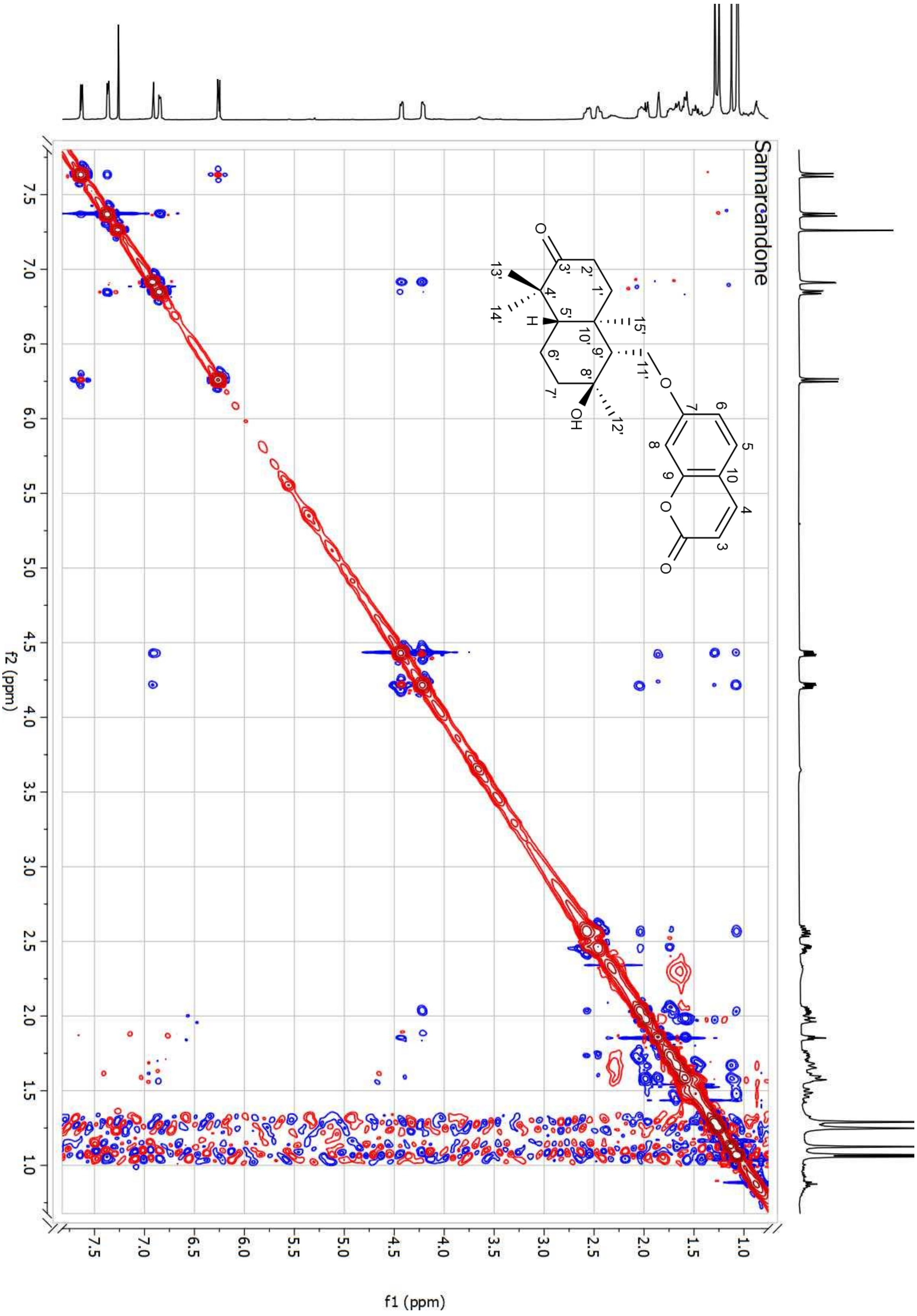

Figure S79. NOESY spectrum ($CDCl_3$) of samarcandone (**13**)

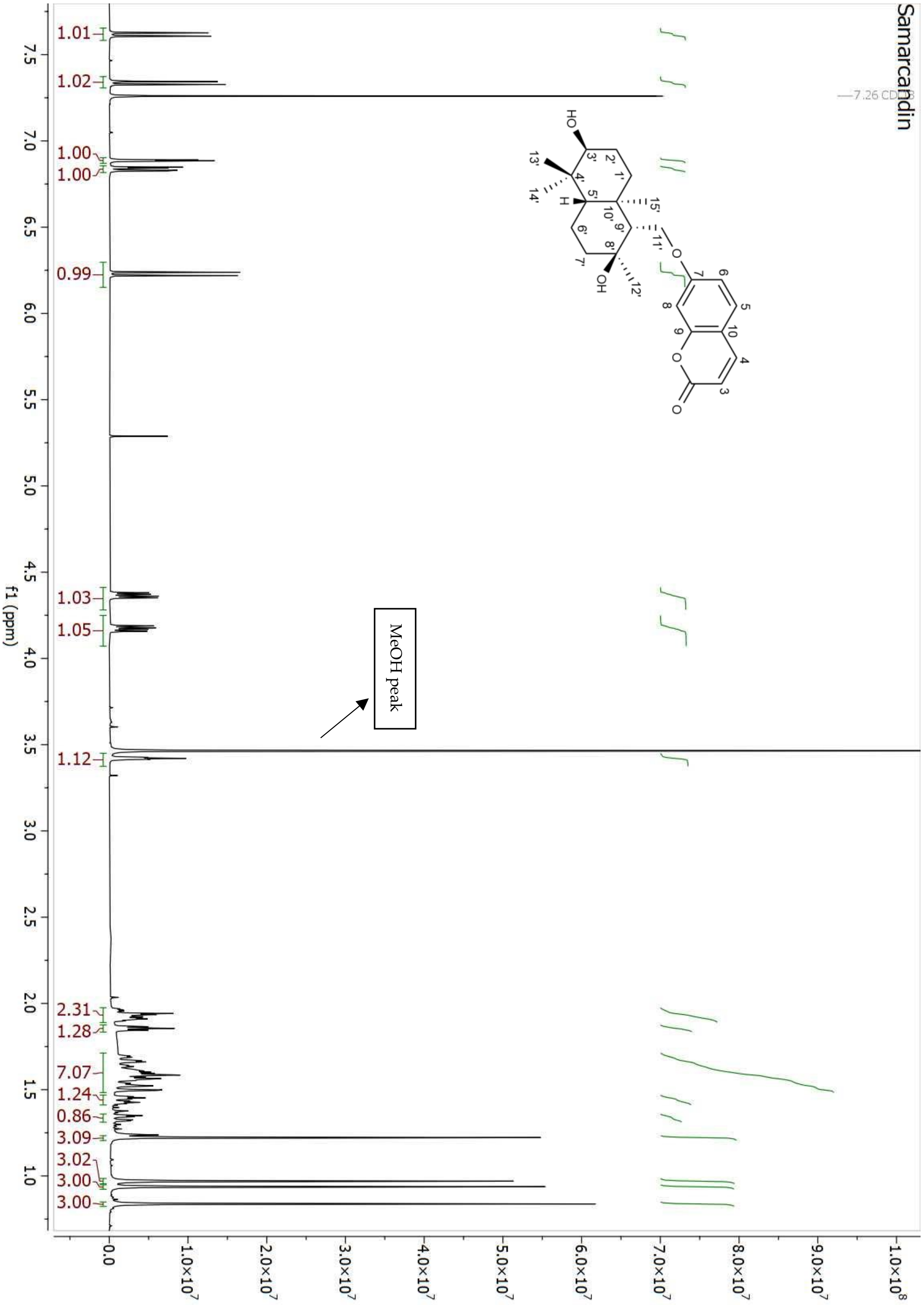

Figure S80. ¹H-NMR spectrum (500 MHz, $CDCl_3$) of samarcandin (**14**)

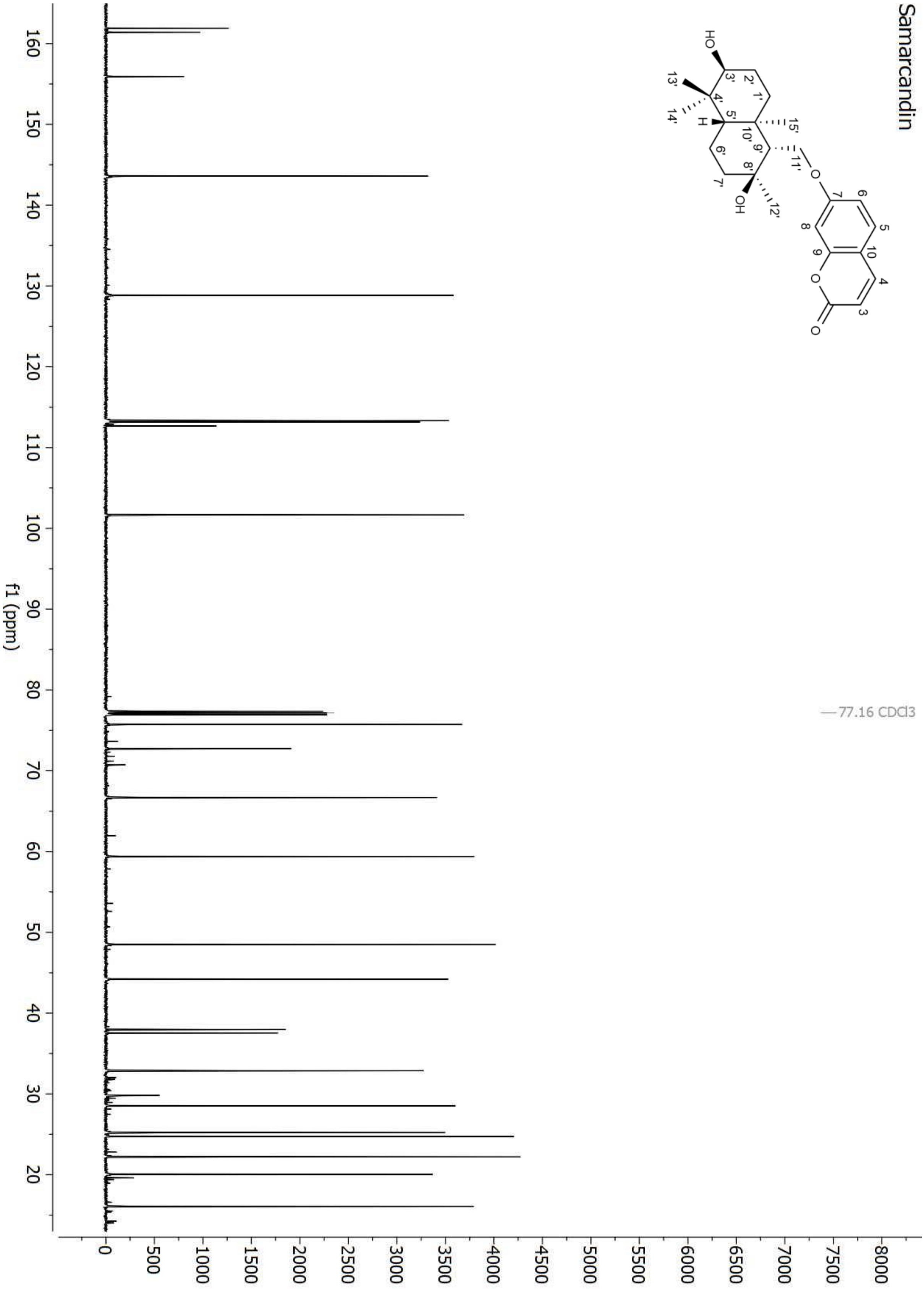

Figure S81. ^{13}C-NMR spectrum (125 MHz, $CDCl_3$) of samarcandin (**14**)

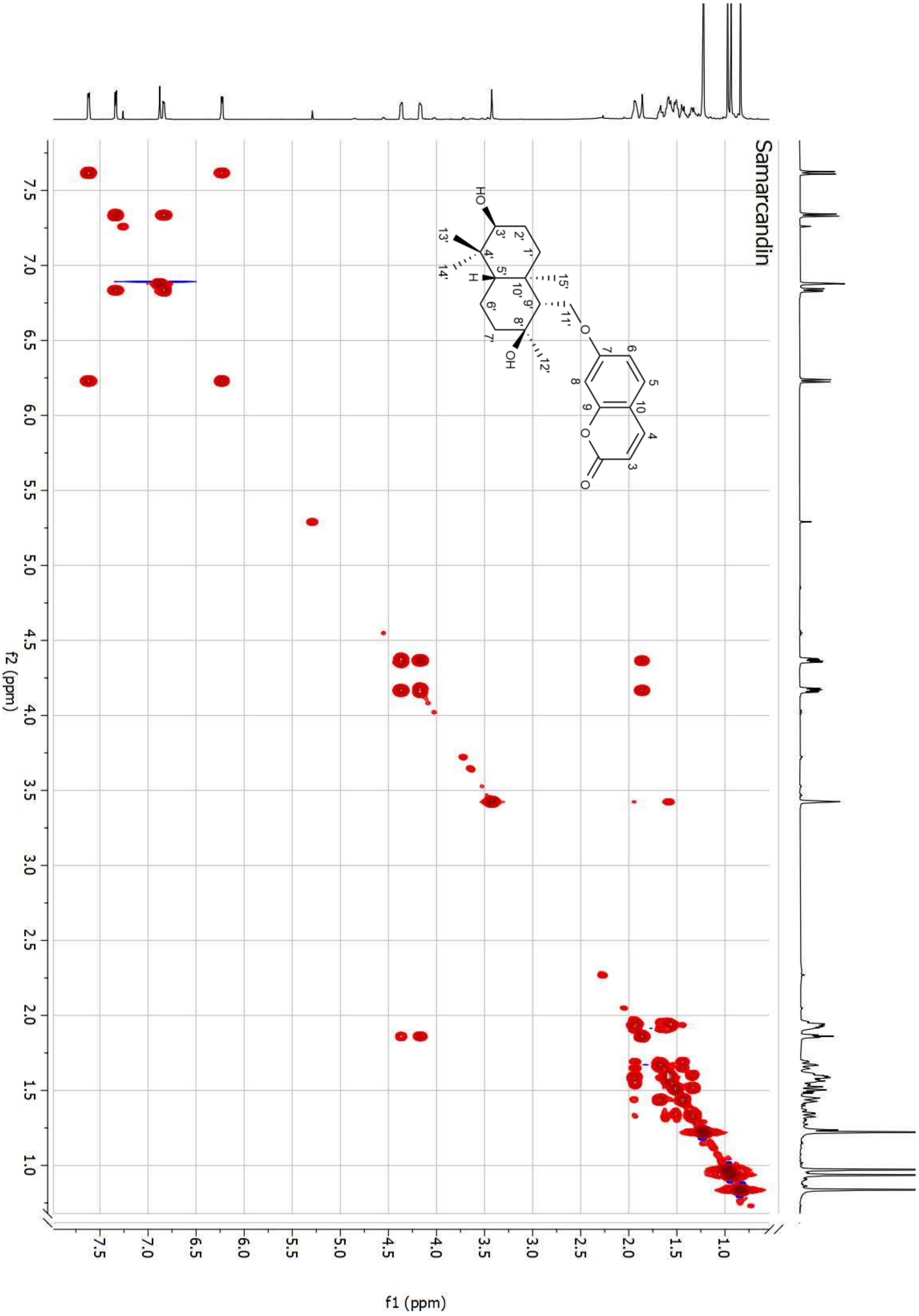

Figure S82. ^{1}H-^{1}H COSY spectrum ($CDCl_3$) of samarcandin (**14**)

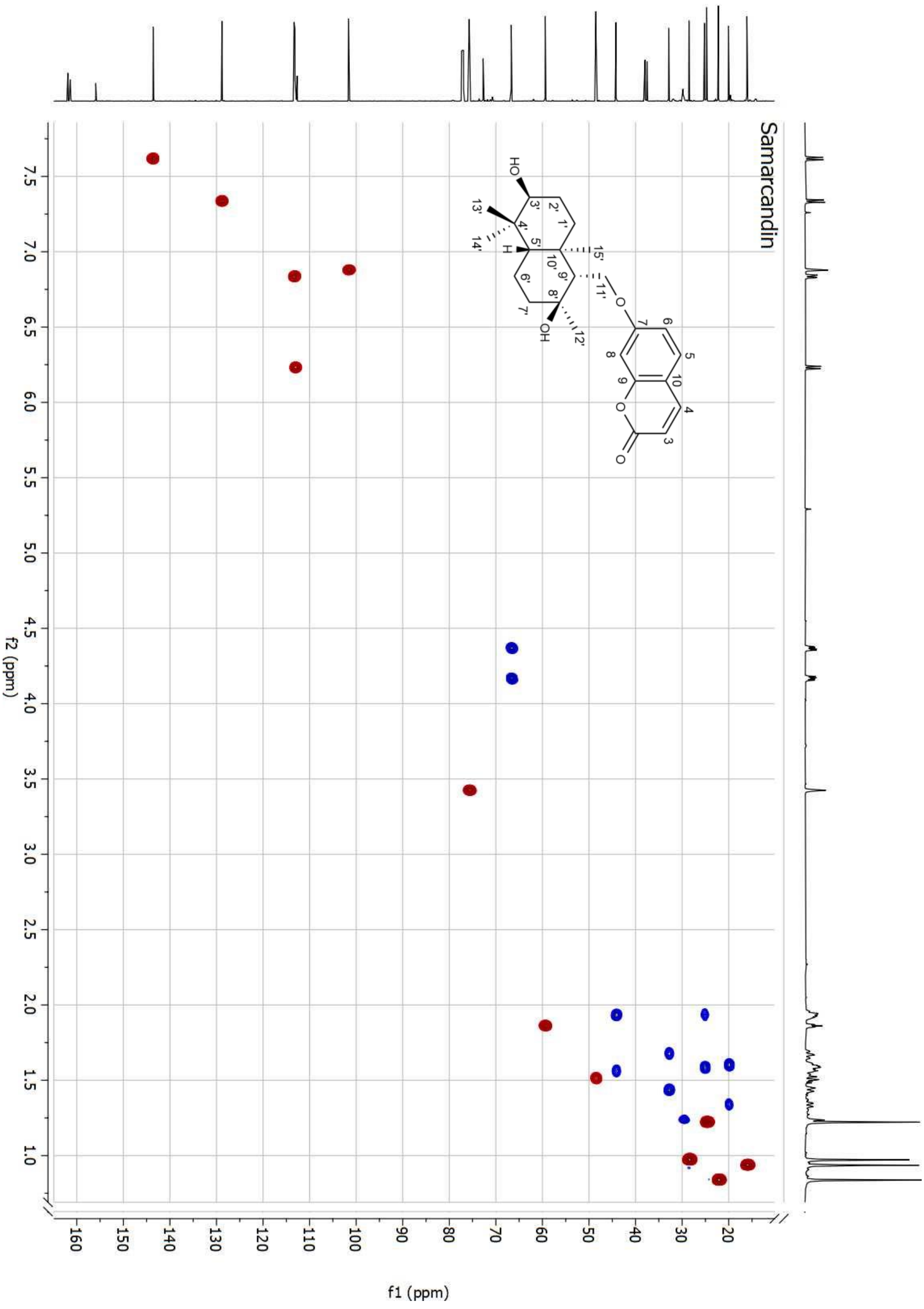

Figure S83. HSQC spectrum ($CDCl_3$) of samarcandin (**14**)

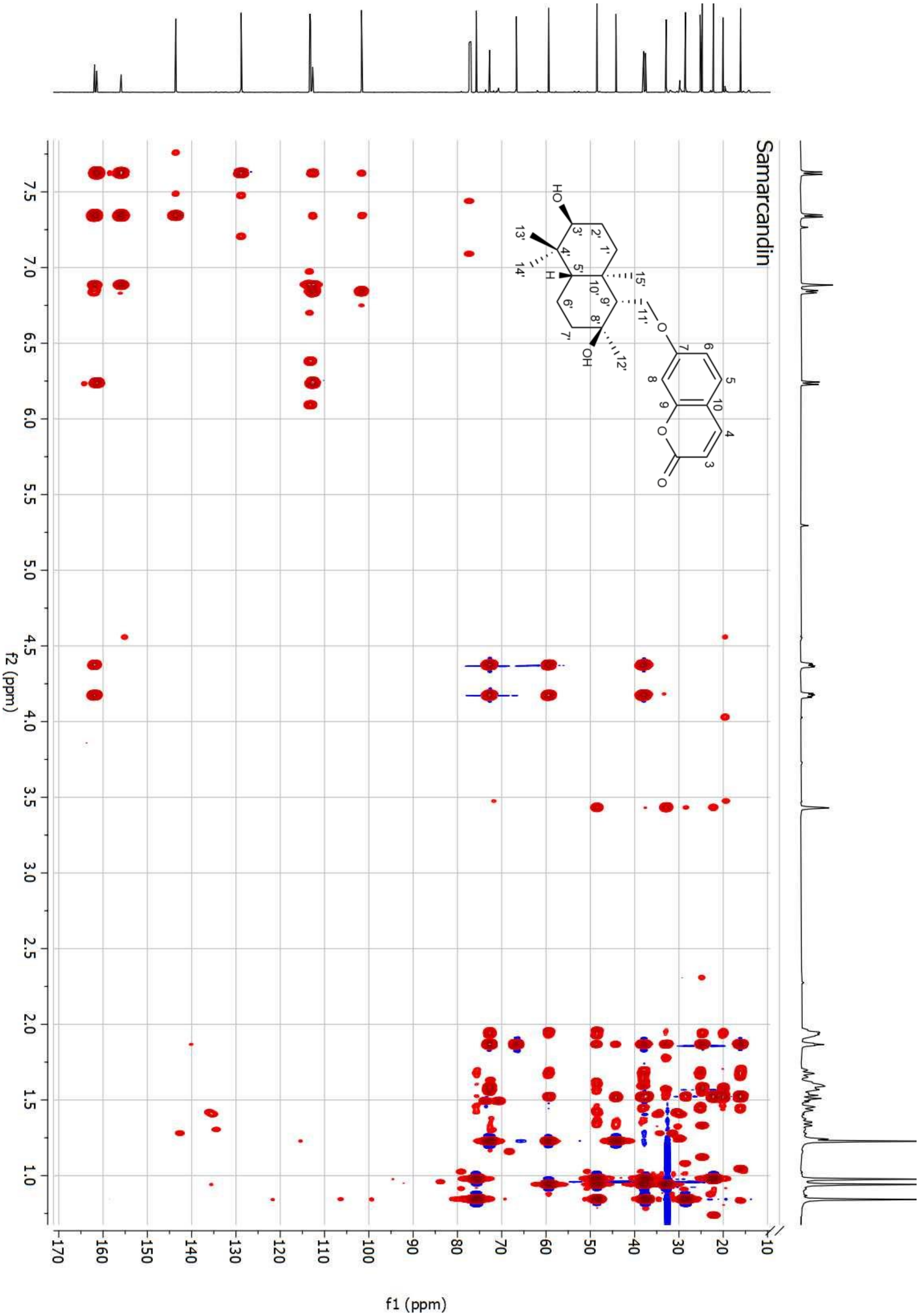

Figure S84. HMBC spectrum (CDCl3) of samarcandin (**14**)

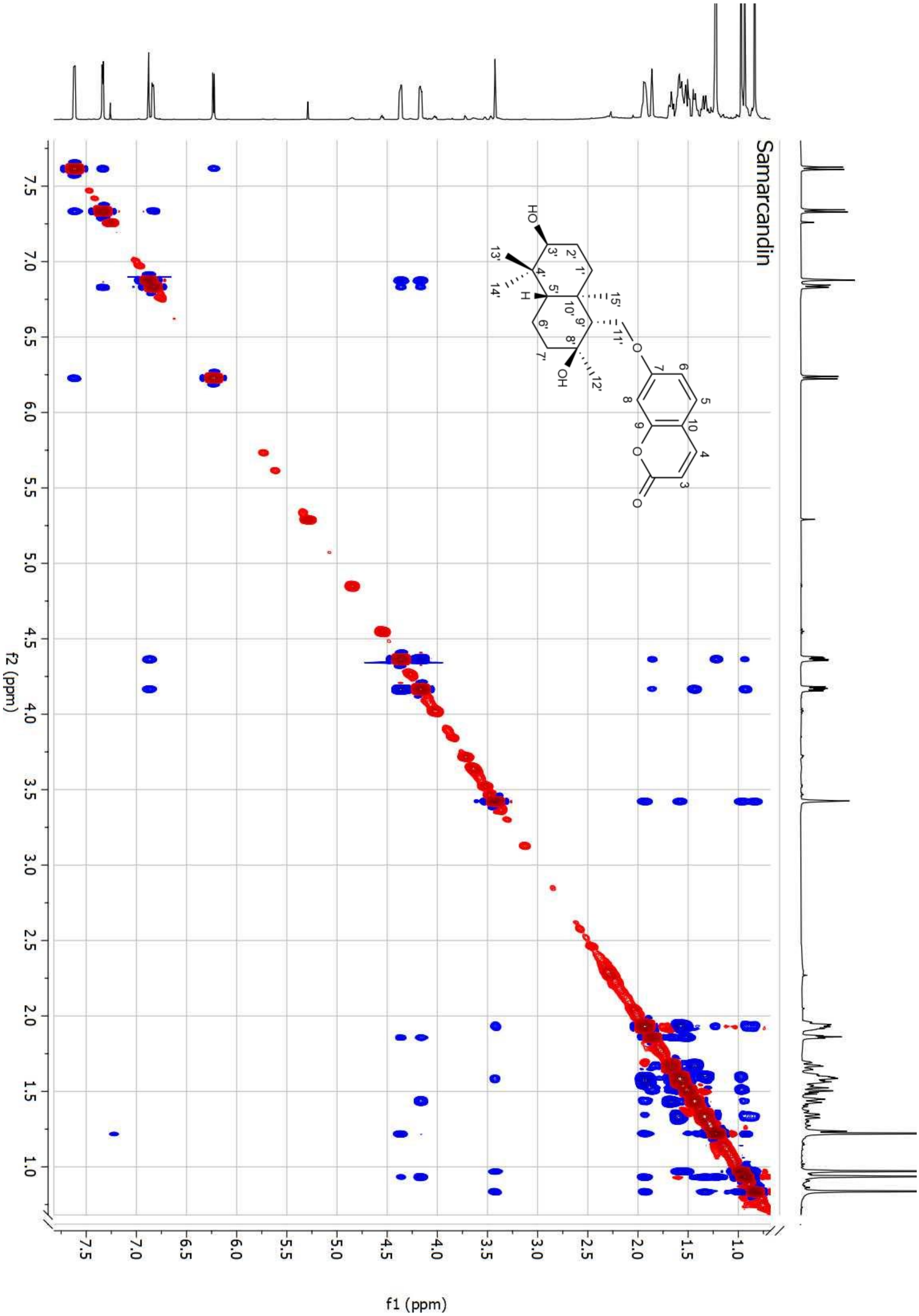

Figure S85. NOESY spectrum ($CDCl_3$) of samarcandin (**14**)

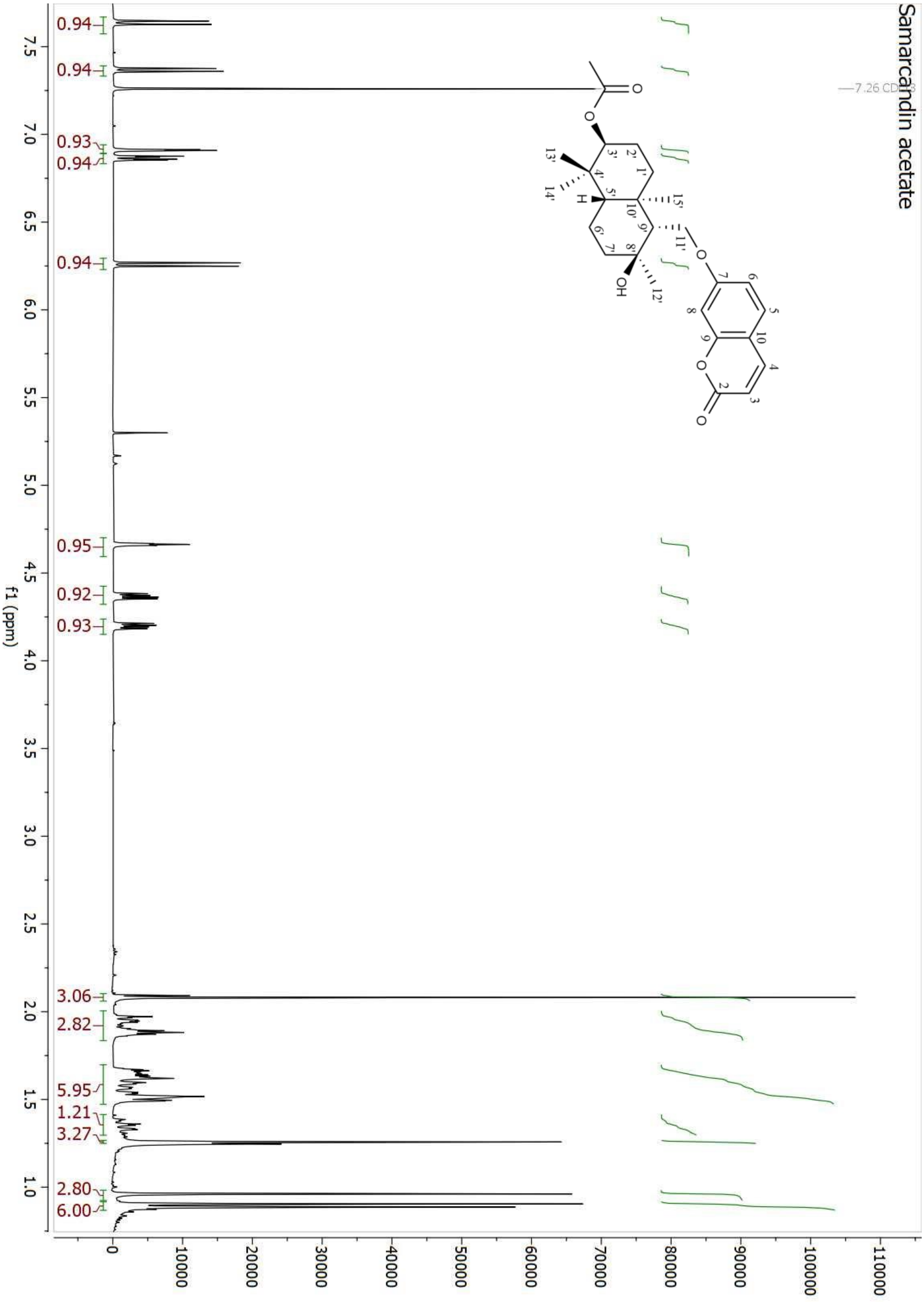

Figure S86. ^{1}H-NMR spectrum (500 MHz, $CDCl_3$) of samarcandin acetate (**15**)

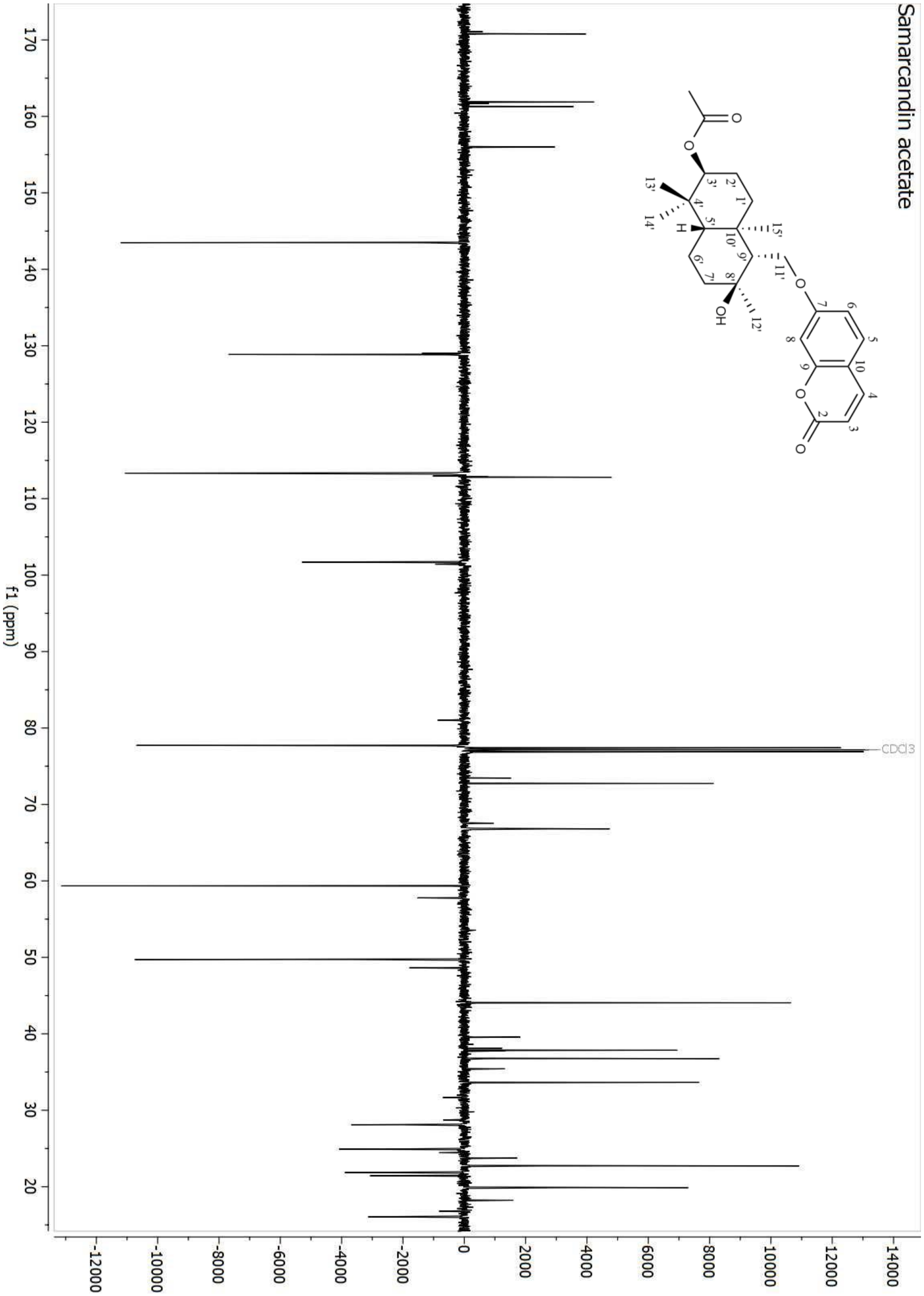

Figure S87. ^{13}C-NMR (APT) spectrum (125 MHz, $CDCl_3$) of samarcandin acetate (**15**)

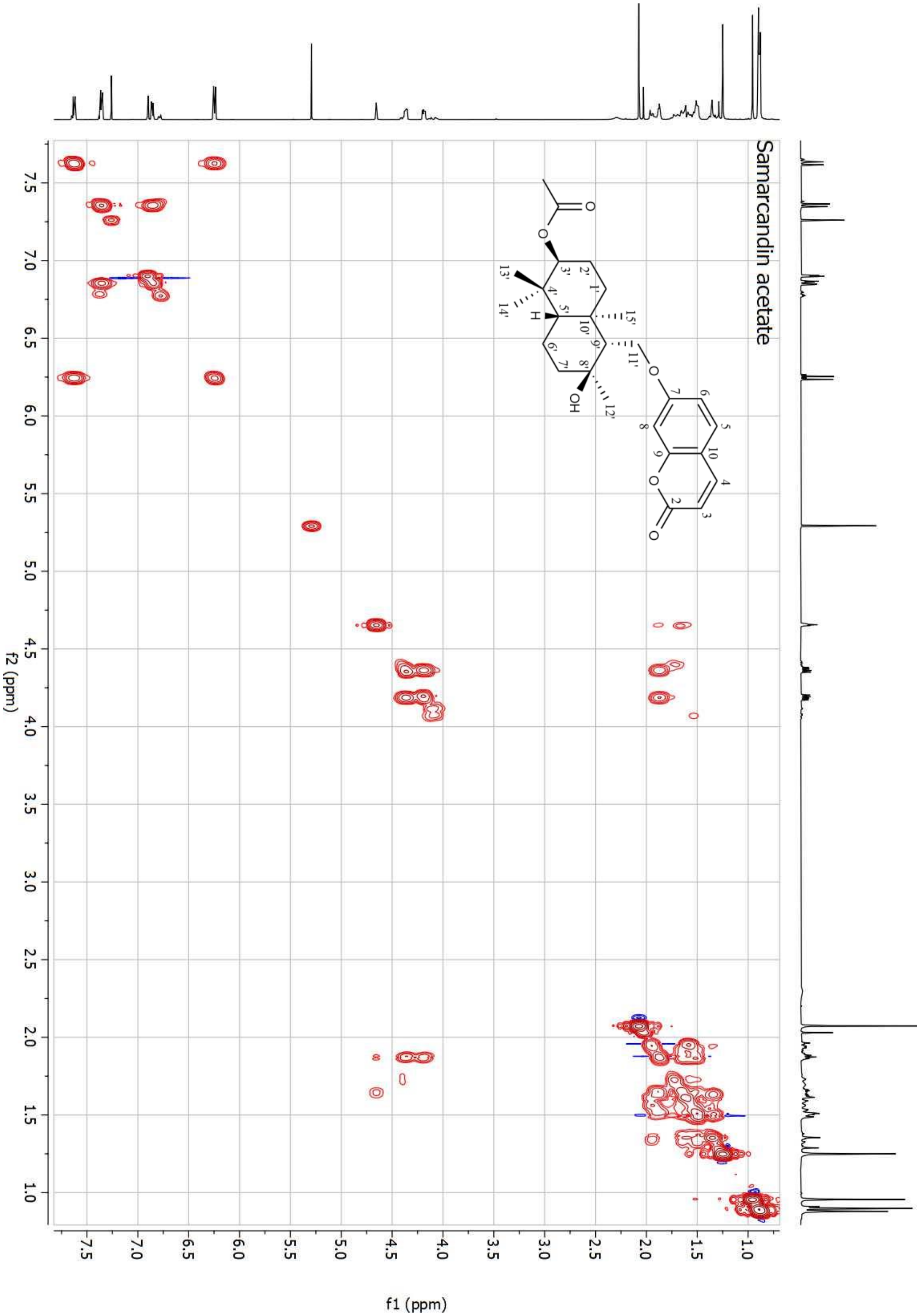

Figure S88. ^{1}H-^{1}H COSY spectrum ($CDCl_3$) of samarcandin acetate (**15**)

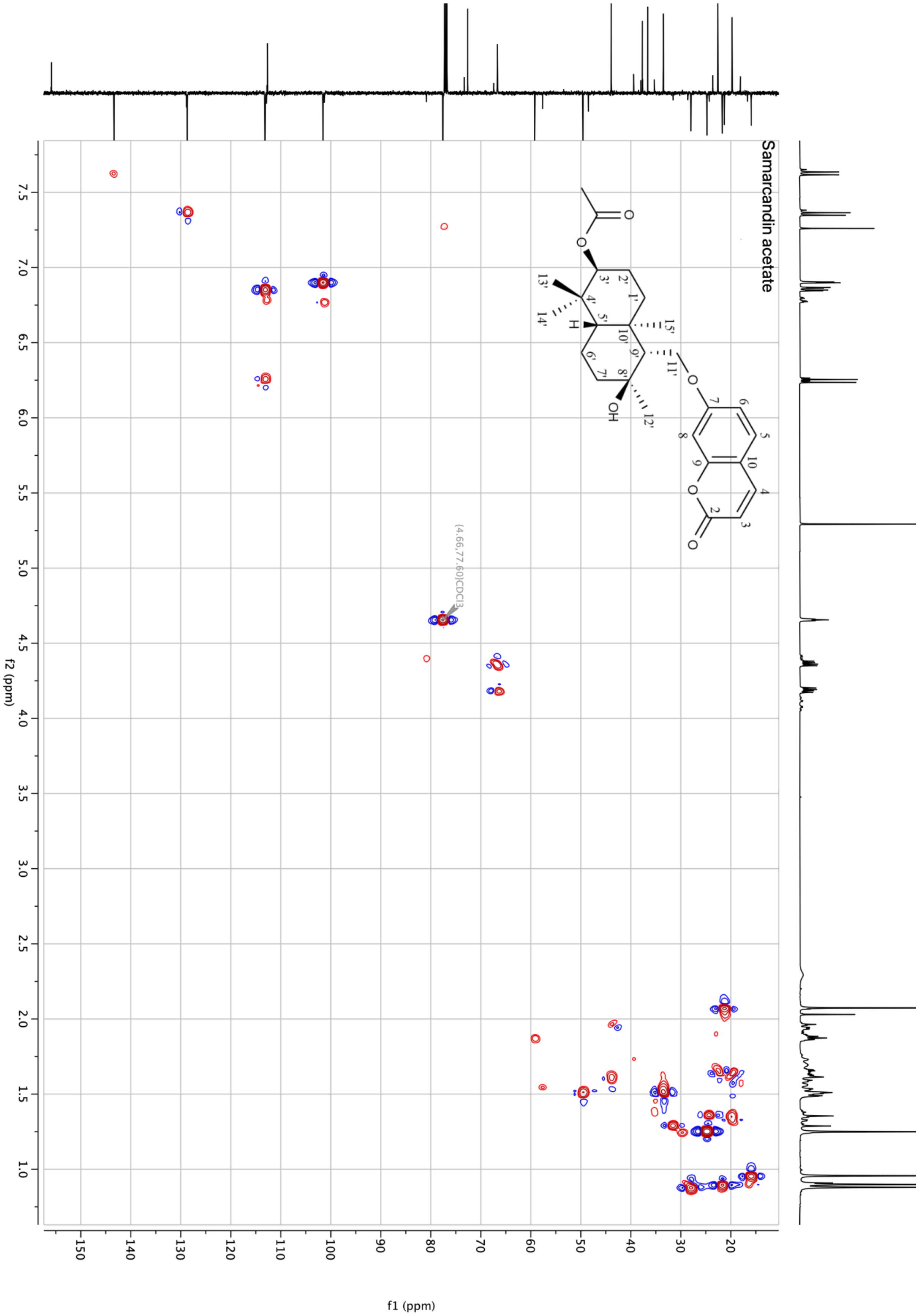

Figure S89. HSQC spectrum ($CDCl_3$) of samarcandin acetate (**15**)

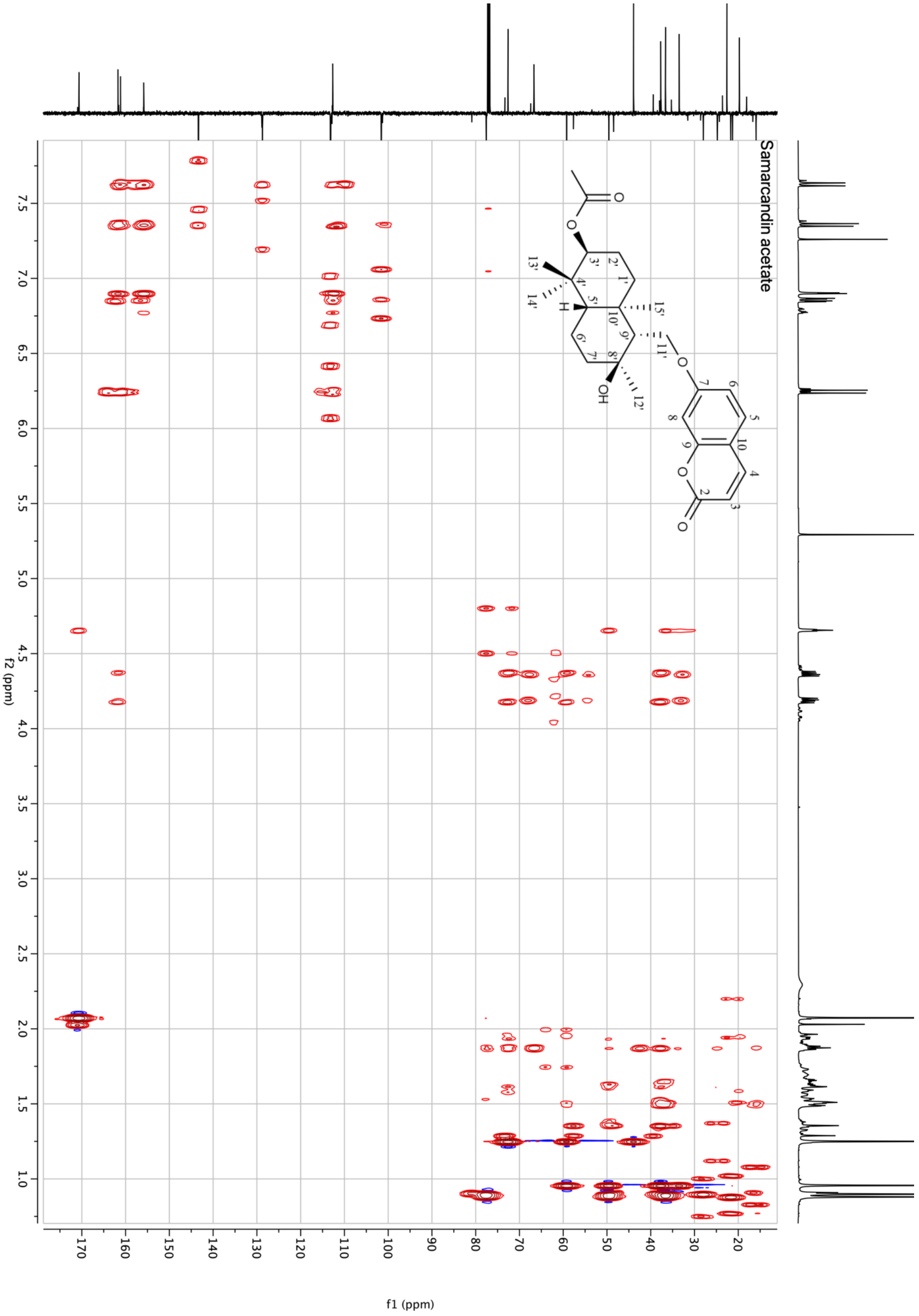

Figure S90. HMBC spectrum ($CDCl_3$) of samarcandin acetate (**15**)

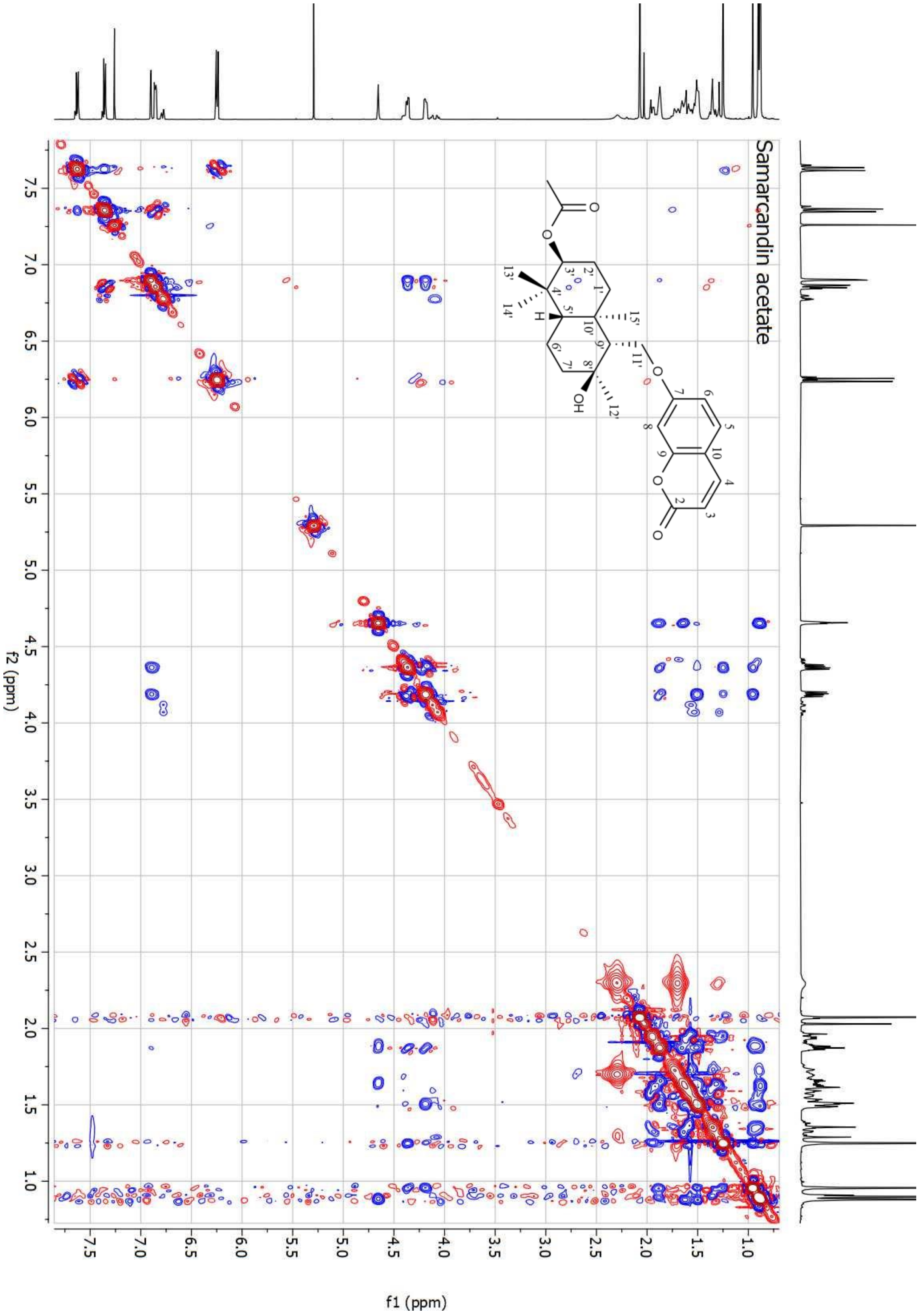

Figure S91. NOESY spectrum ($CDCl_3$) of samarcandin acetate (**15**)

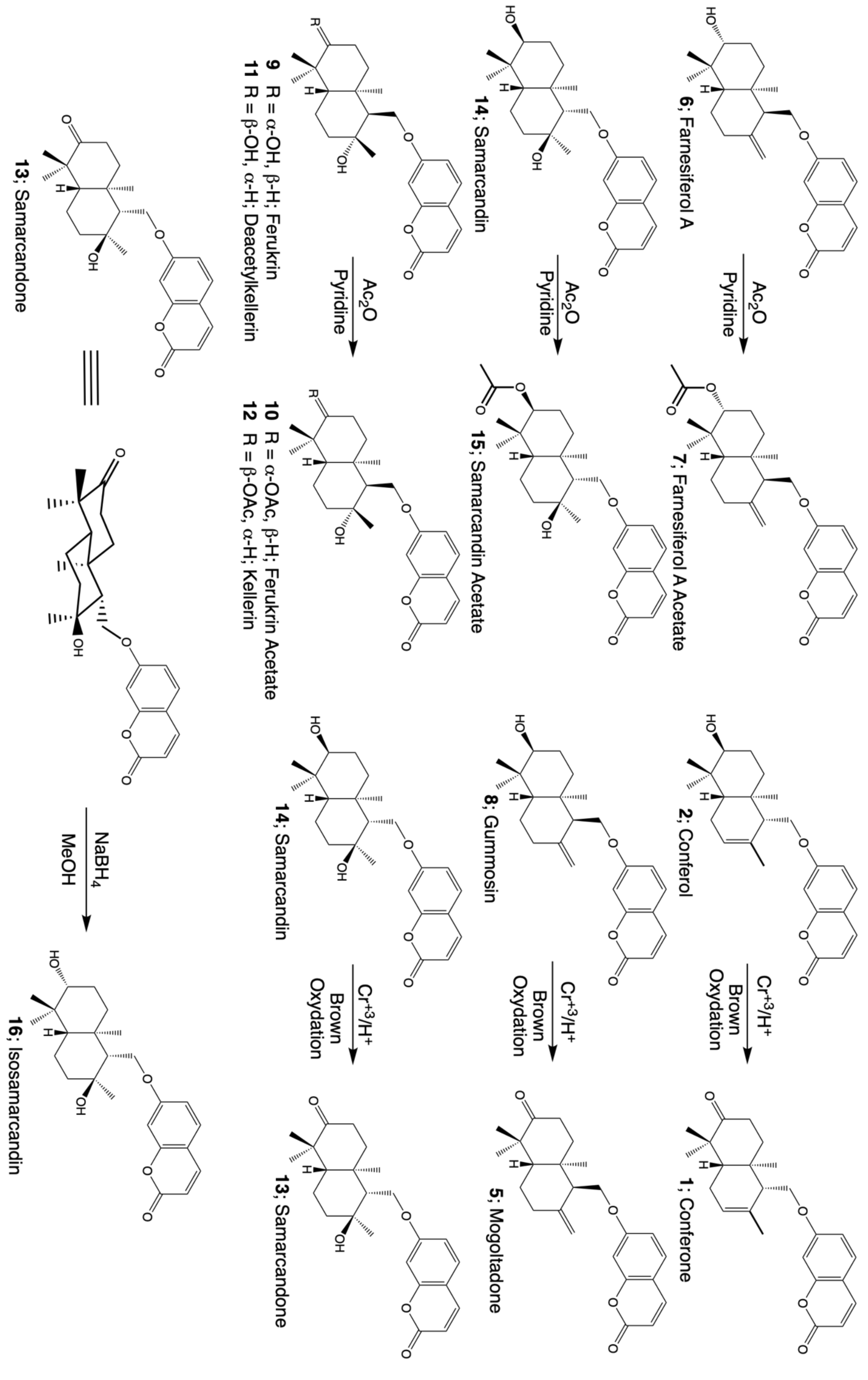

Figure S92. Chemical transformations of the cytotoxic sesquiterpene coumarins of *Ferula huber-morathii*

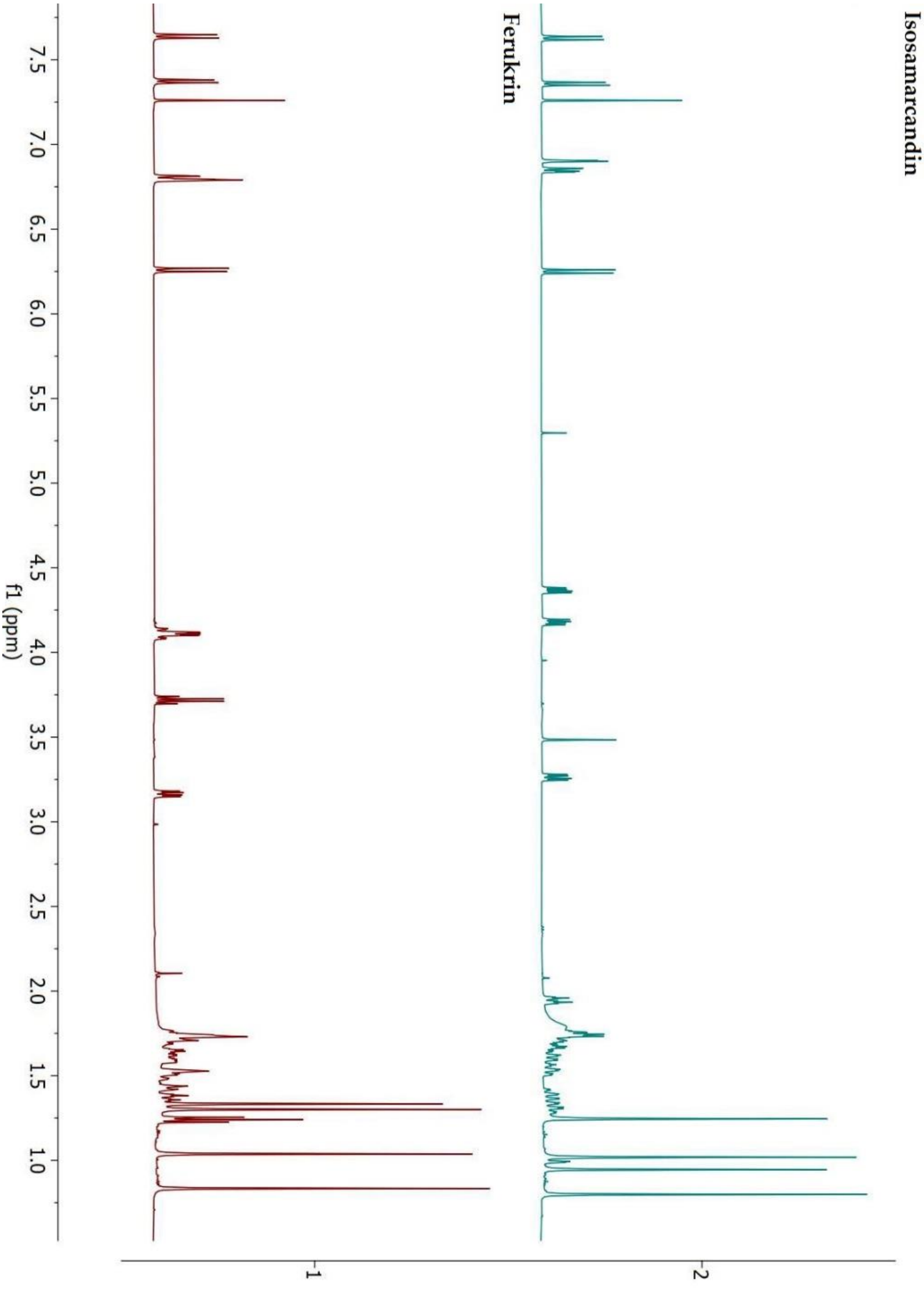

Figure S93. Comparison of ¹H-NMR spectra of isosamarcandin (**16**) and ferukrin (**9**)

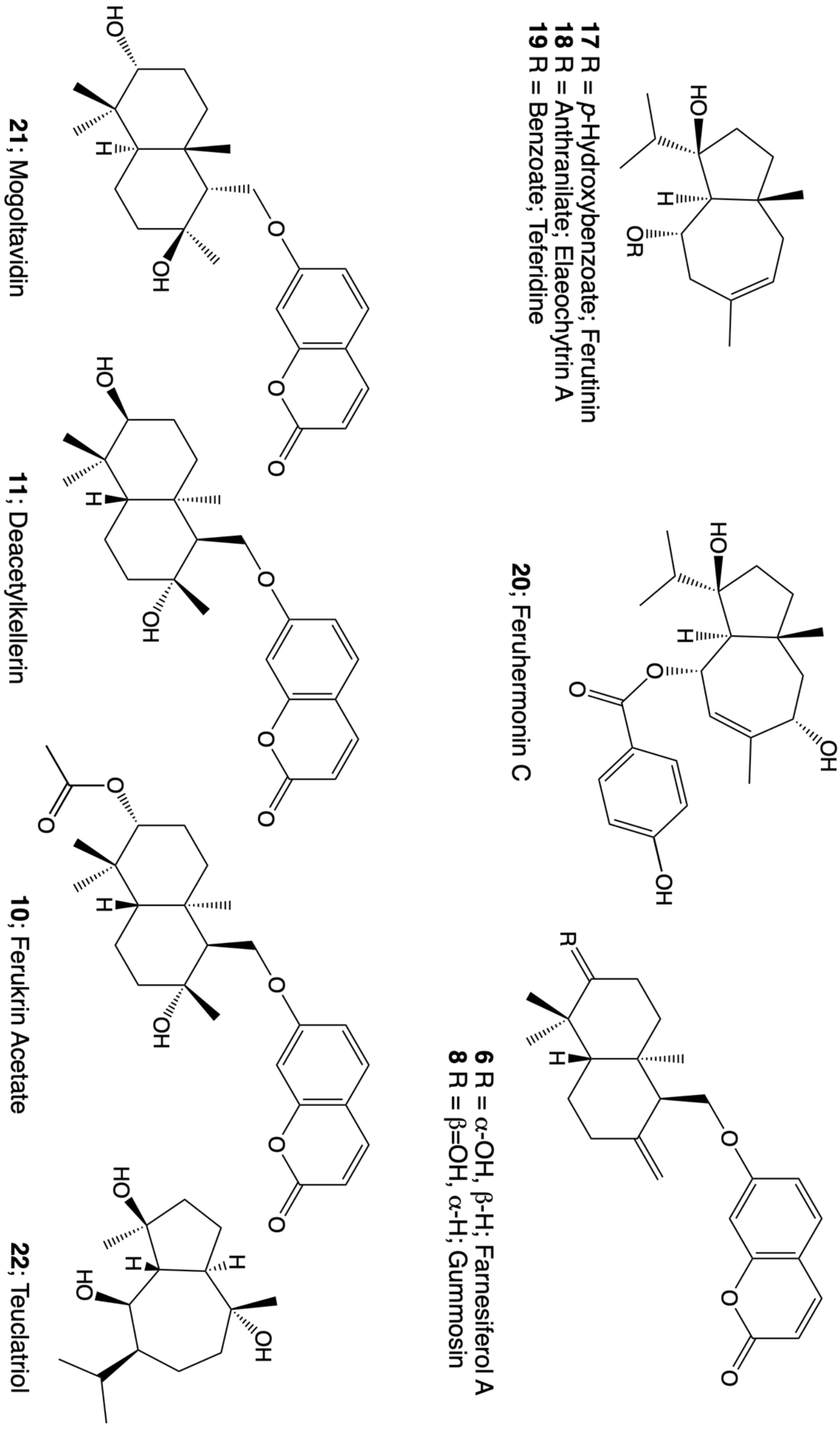

Figure S94. Previously proposed structures of sesquiterpene compounds isolated from the chloroform extract of *Ferula huber-morathii* [3]

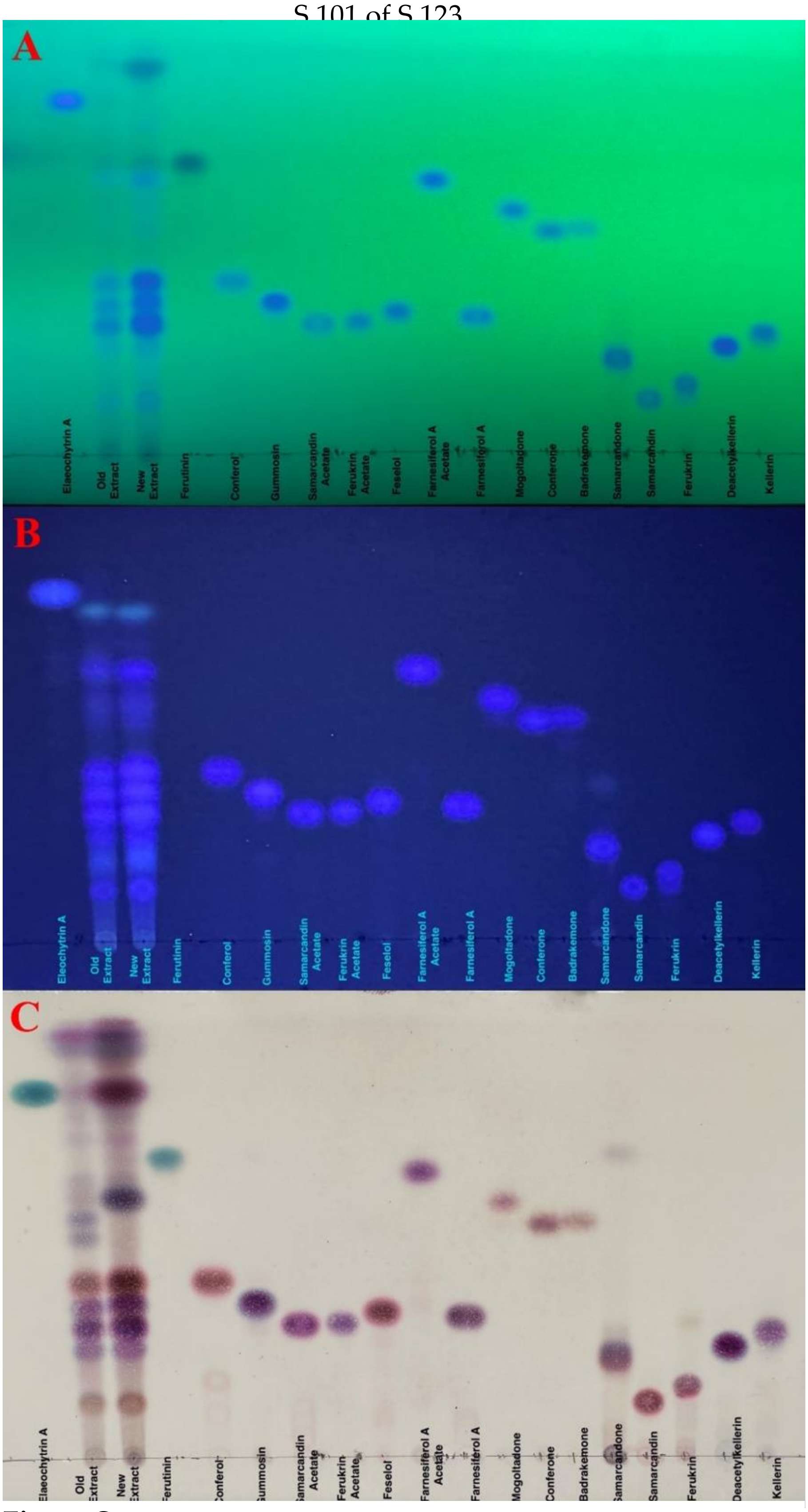

Figure S95. TLC comparison of the dichloromethane extracts of *Ferula huber-morathii* with reference compounds; elaeochytrin A (**18**) and ferutinin (**17**), and isolated sesquiterpene coumarins (**1-15**), mobile phase; Hxn:EtOAc (6:4), detection; **A**: 254 nm; **B**: 366 nm; **C**: Anisaldehyde reagent

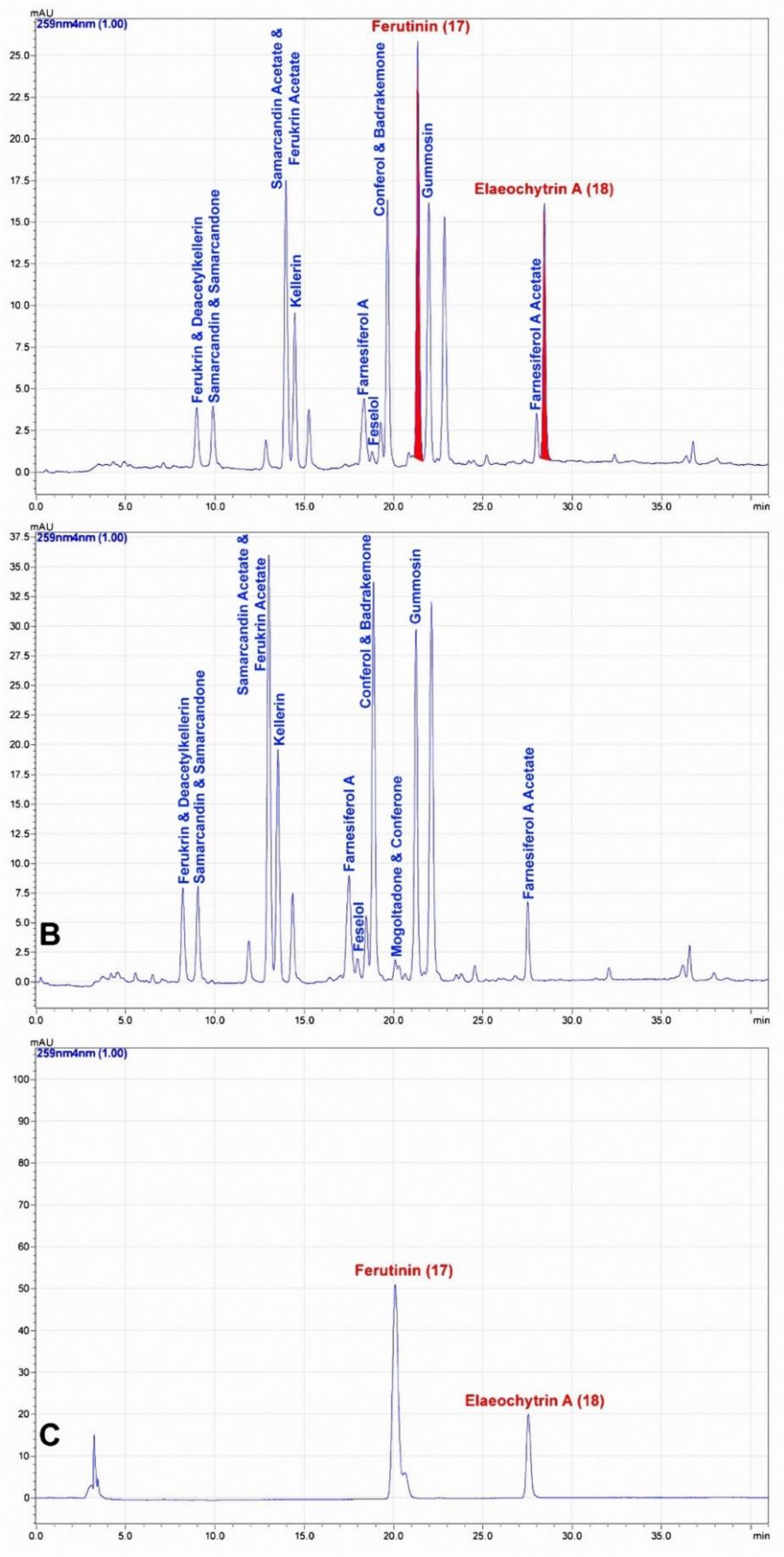

Figure S96. A. HPLC chromatogram of **t**he dichloromethane root extract of *Ferula huber-morathii* spiked with reference compounds ferutinin (**17**) and elaeochytrin A (**18**), **B.** HPLC chromatogram of the dichloromethane root extract of *F. huber-morathii*

and **C.** HPLC chromatogram of the mixture of reference compounds ferutinin (**17**) and elaeochytrin A (**18**). Detection: UV detector at 259 nm

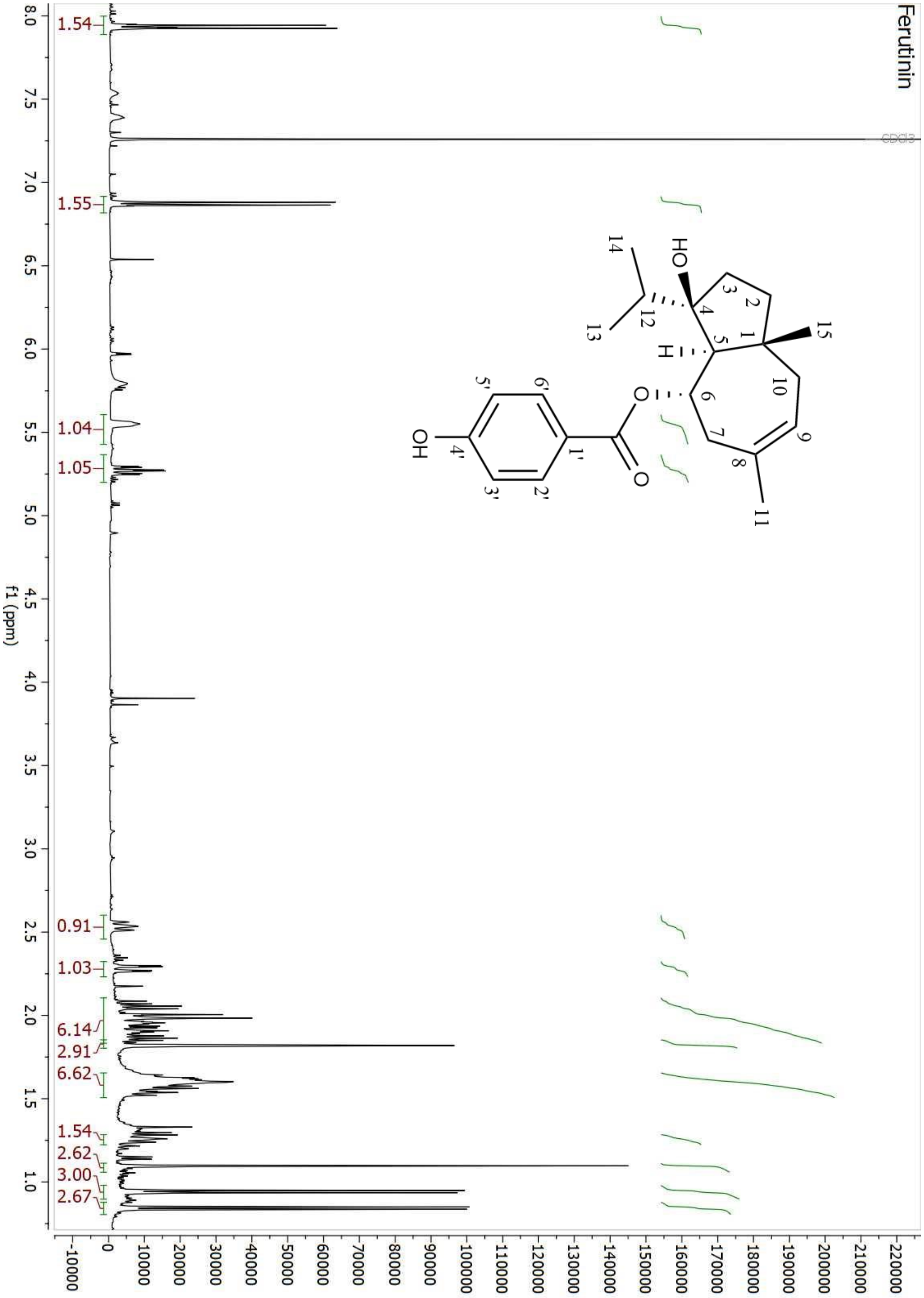
Ferutinin
CDCl3
1.54
1.55
1.04
1.05
0.91
1.03
6.14
2.91
6.62
1.54
2.62
3.00
2.67
f1 (ppm)
HO
OH
O
H

Figure S97. ^{1}H-NMR spectrum (500 MHz, $CDCl_3$) of reference compound ferutinin (**17**)

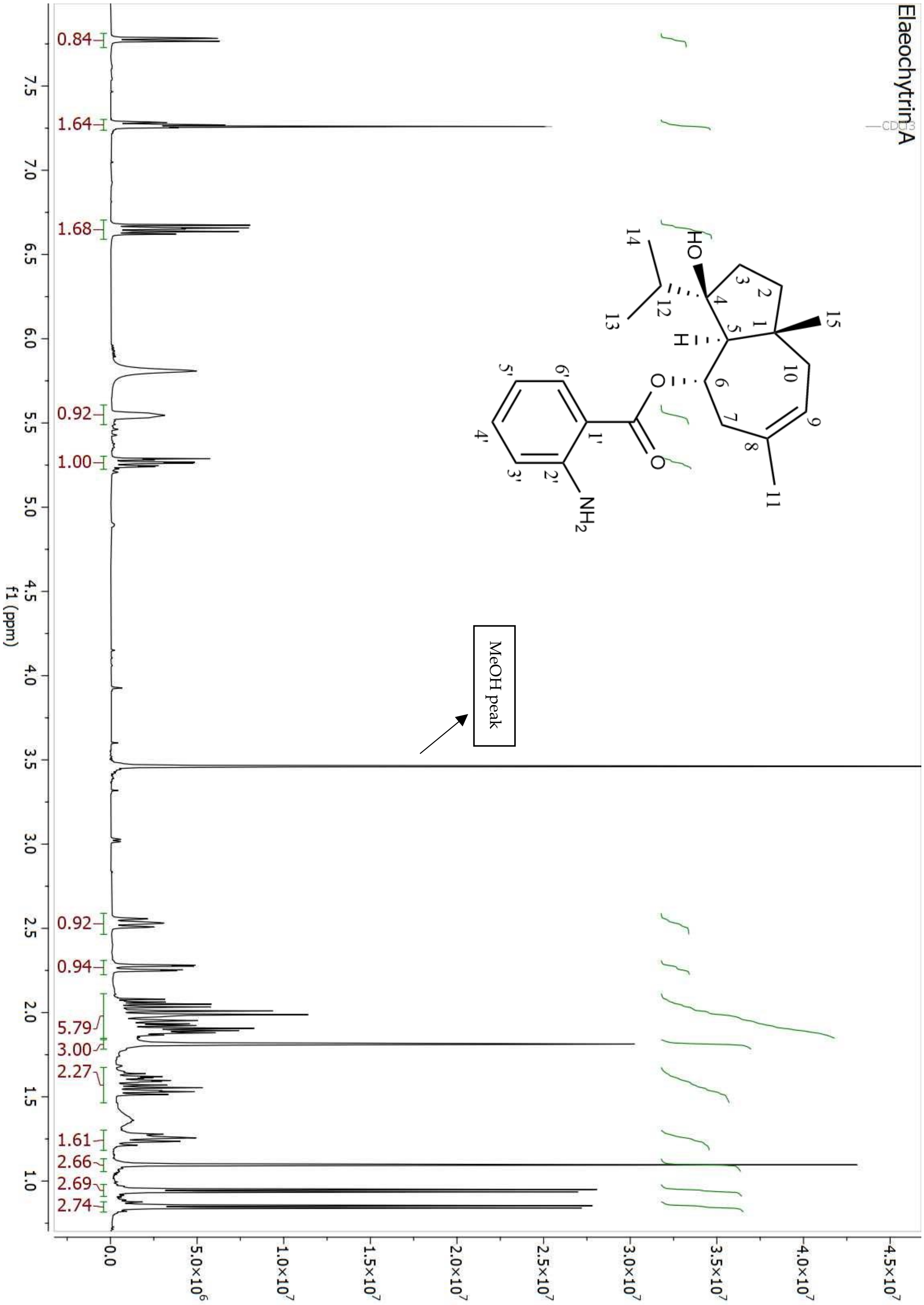
Elaeochytrin A
CDCl3
MeOH peak
f1 (ppm)
HO
H
O
O
NH2
1
2
3
4
5
6
7
8
9
10
11
12
13
14
15
1'
2'
3'
4'
5'
6'
0.84
1.64
1.68
0.92
1.00
0.92
0.94
5.79
3.00
2.27
1.61
2.66
2.69
2.74
7.5
7.0
6.5
6.0
5.5
5.0
4.5
4.0
3.5
3.0
2.5
2.0
1.5
1.0
0.0
5.0×10^6
1.0×10^7
1.5×10^7
2.0×10^7
2.5×10^7
3.0×10^7
3.5×10^7
4.0×10^7
4.5×10^7

Figure S98. ^{1}H-NMR spectrum (500 MHz, $CDCl_3$) of reference compound elaeochtyrin A (**18**)

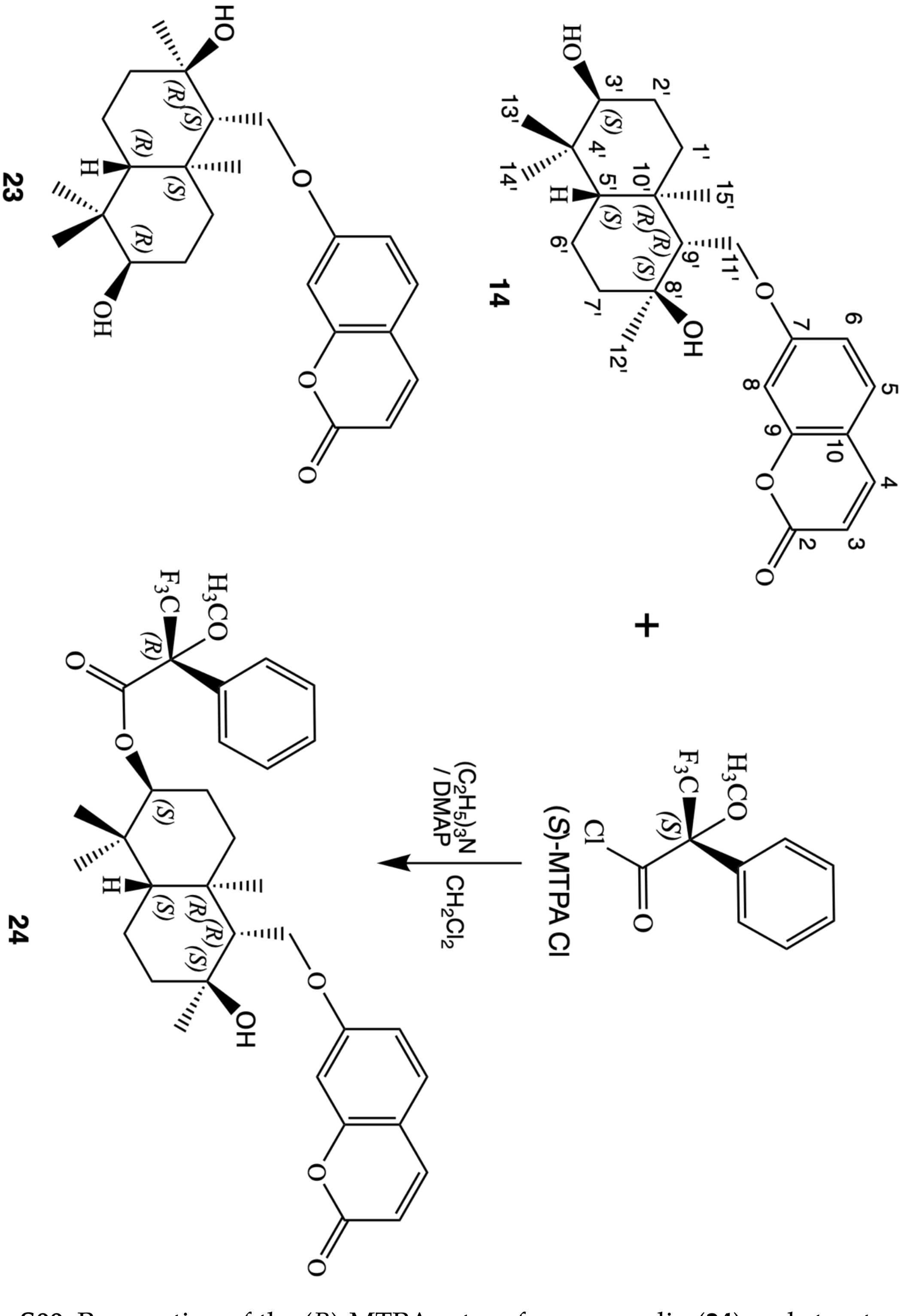

Figure S99. Preparation of the (*R*)-MTPA ester of samarcandin (**24**) and structure of *ent*-samarcandin (**23**)

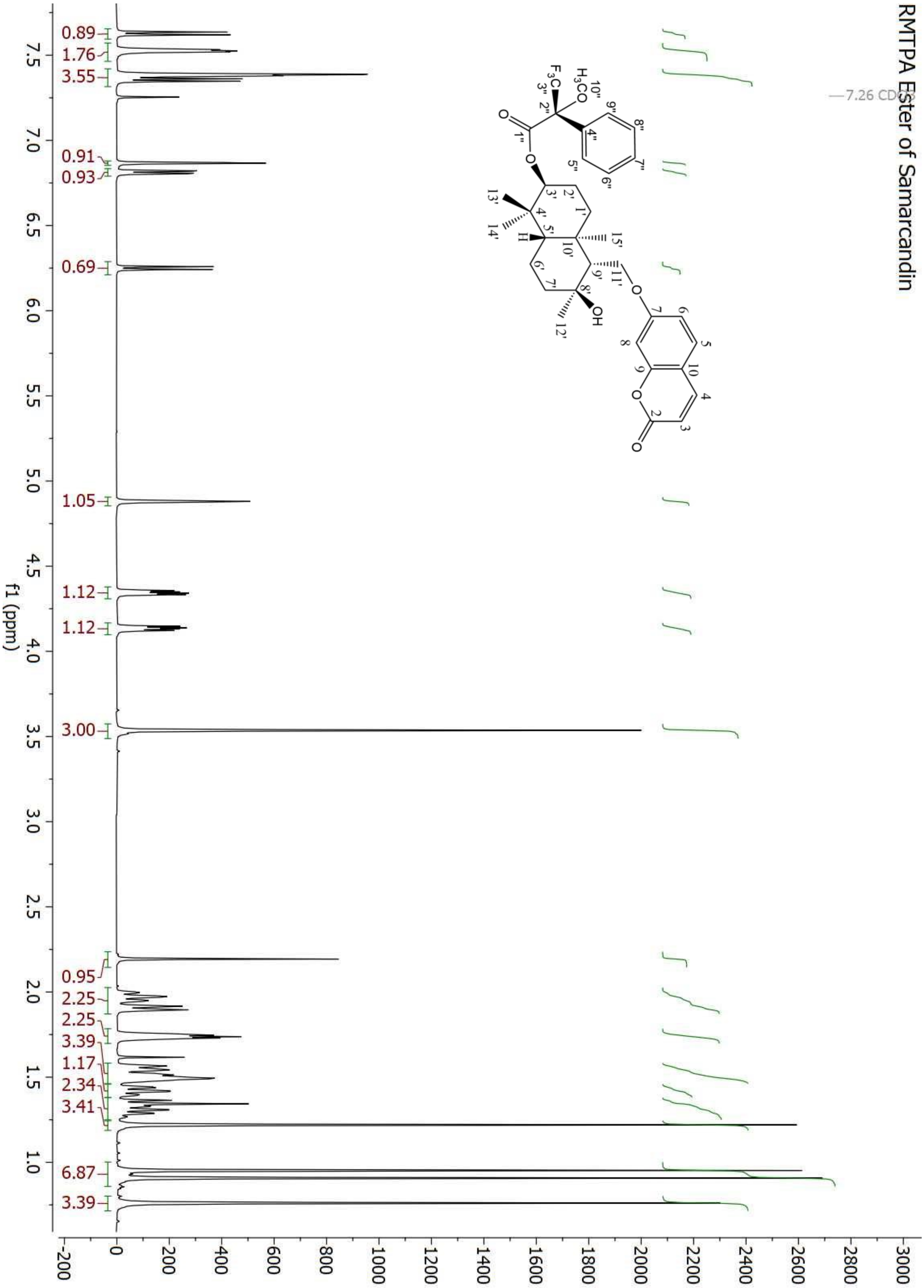
RMTPA Ester of Samarcandin
f1 (ppm)

Figure S100. ^{1}H-NMR spectrum (500 MHz, $CDCl_3$) of (*R*)-MTPA ester of samarcandin (**24**)

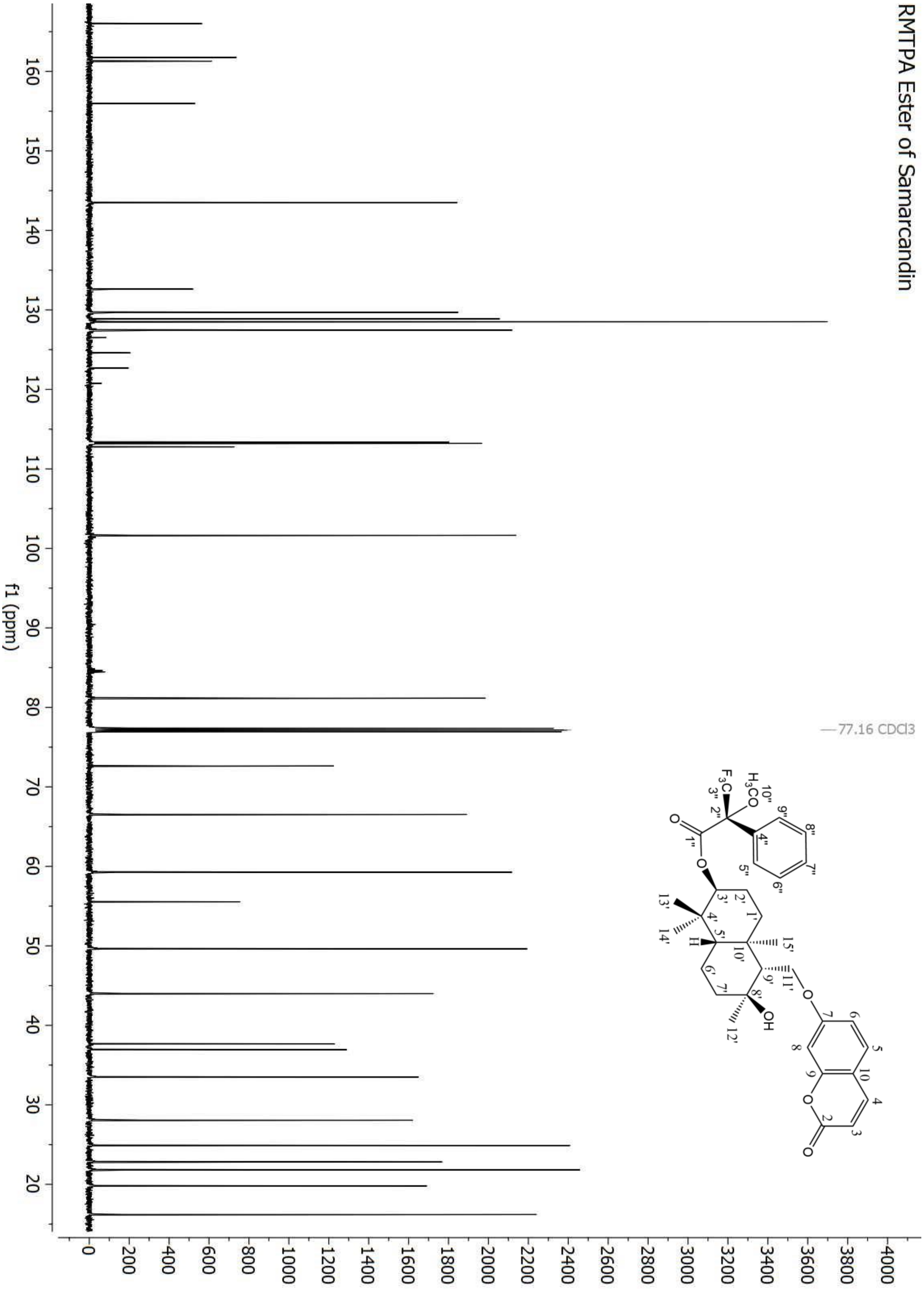
RMTPA Ester of Samarcandin
—77.16 CDCl3
f1 (ppm)

Figure S101. ^{13}C-NMR spectrum (125 MHz, $CDCl_3$) of (*R*)-MTPA ester of samarcandin (**24**)

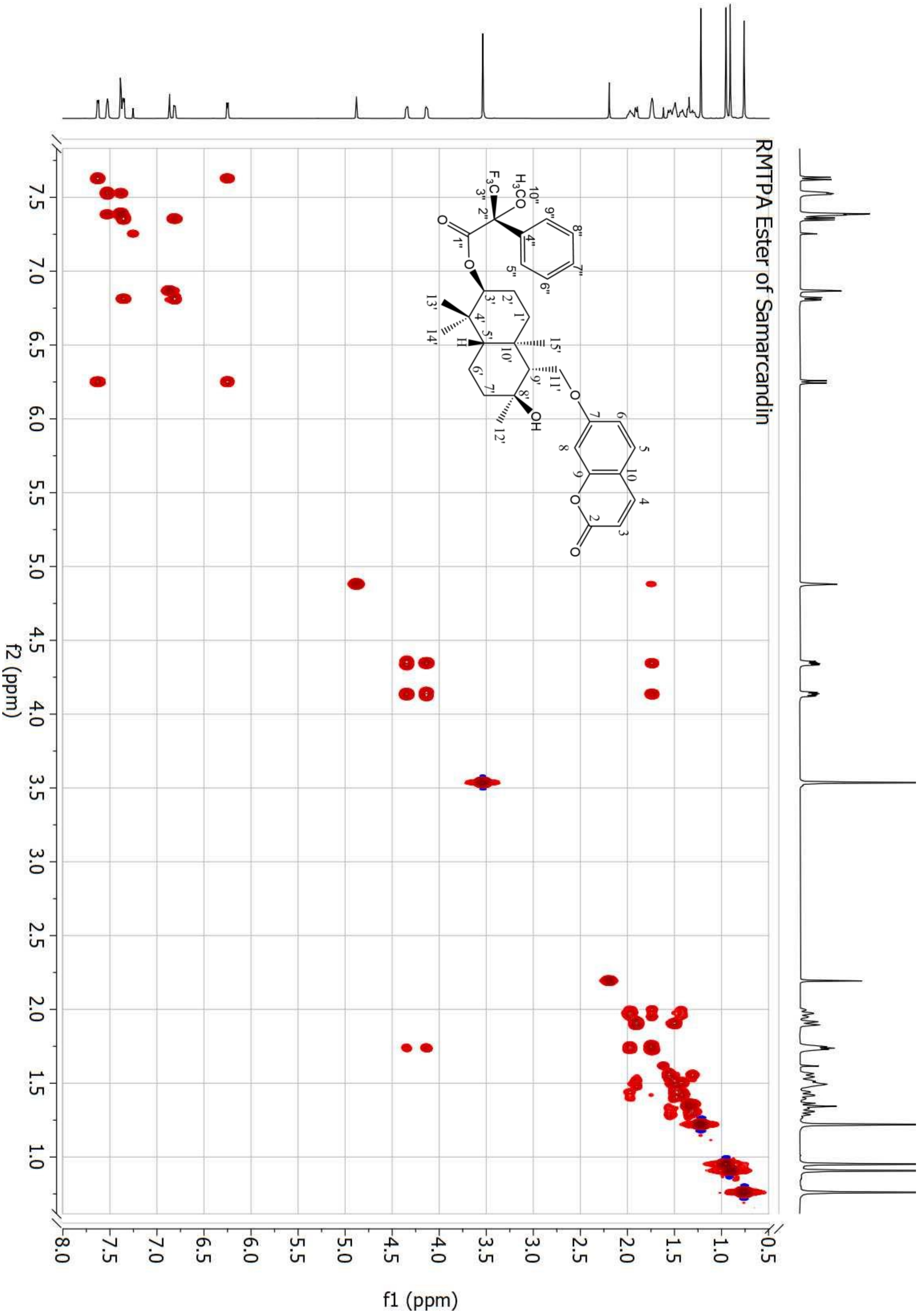

Figure S102. ^{1}H-^{1}H COSY spectrum ($CDCl_3$) of (*R*)-MTPA ester of samarcandin (**24**)

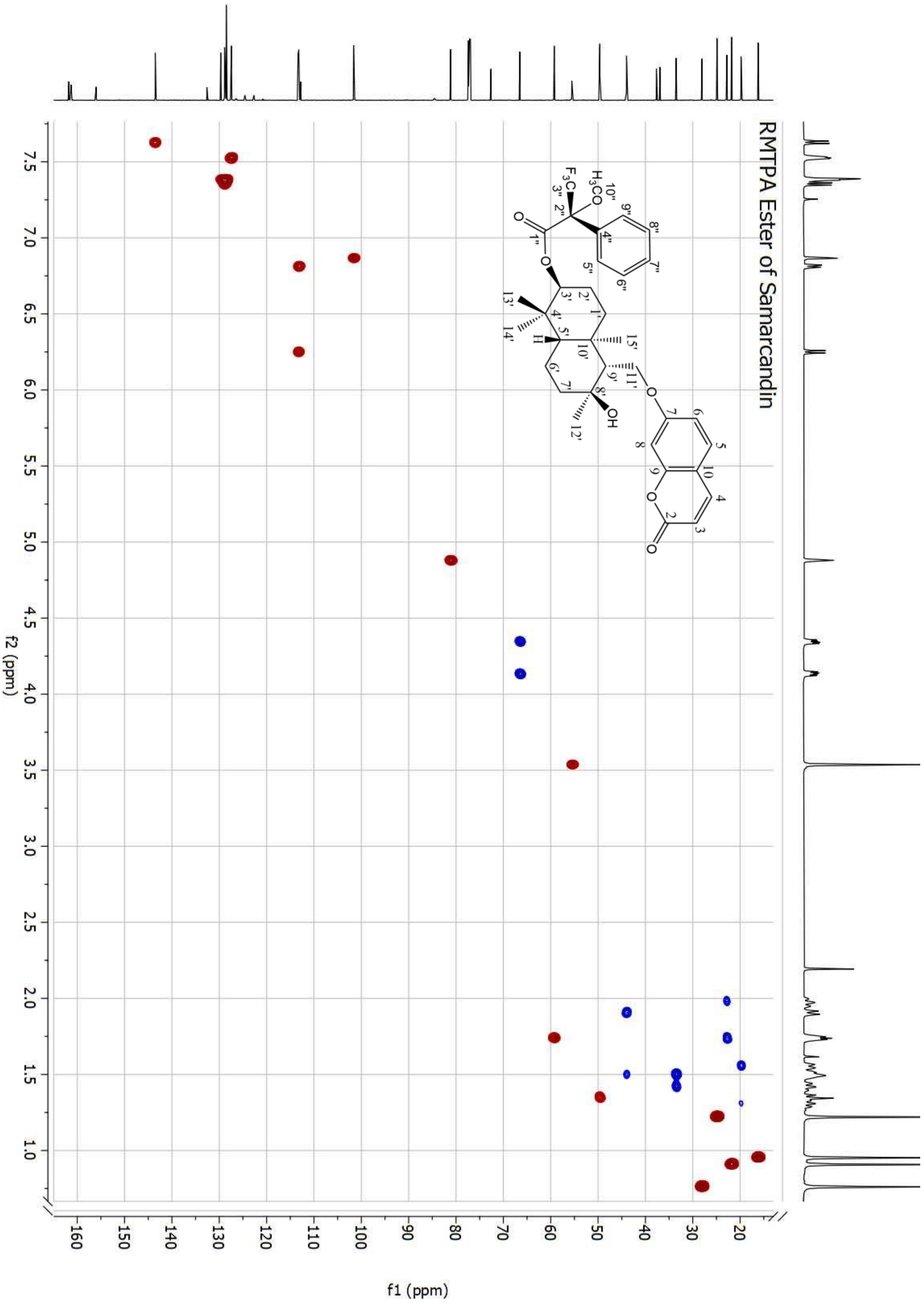

Figure S103. HSQC spectrum of (*R*)-MTPA ester of samarcandin (**24**)

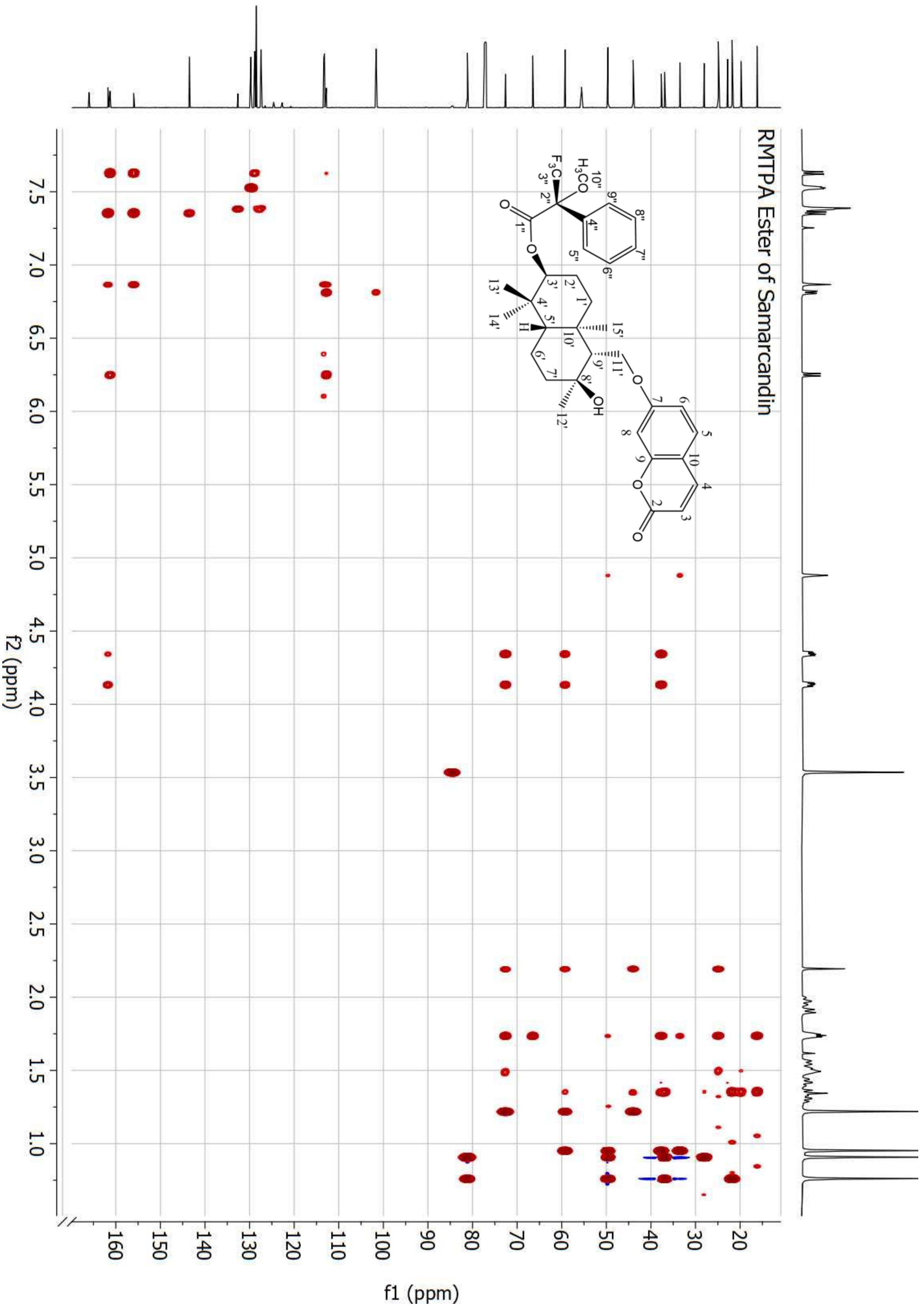

Figure S104. HMBC spectrum of (*R*)-MTPA ester of samarcandin (**24**)

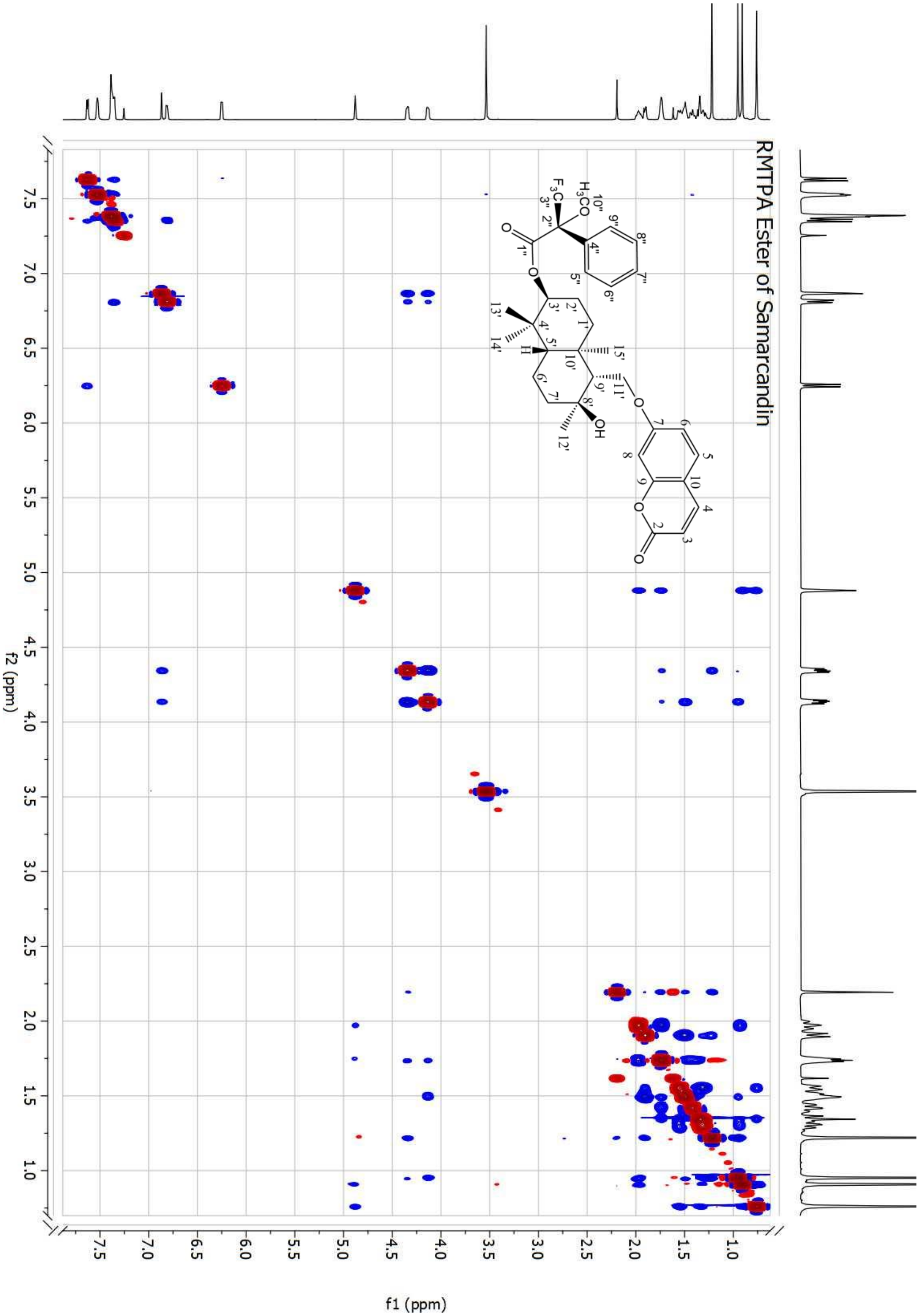

Figure S105. NOESY spectrum of (*R*)-MTPA ester of samarcandin (**24**)

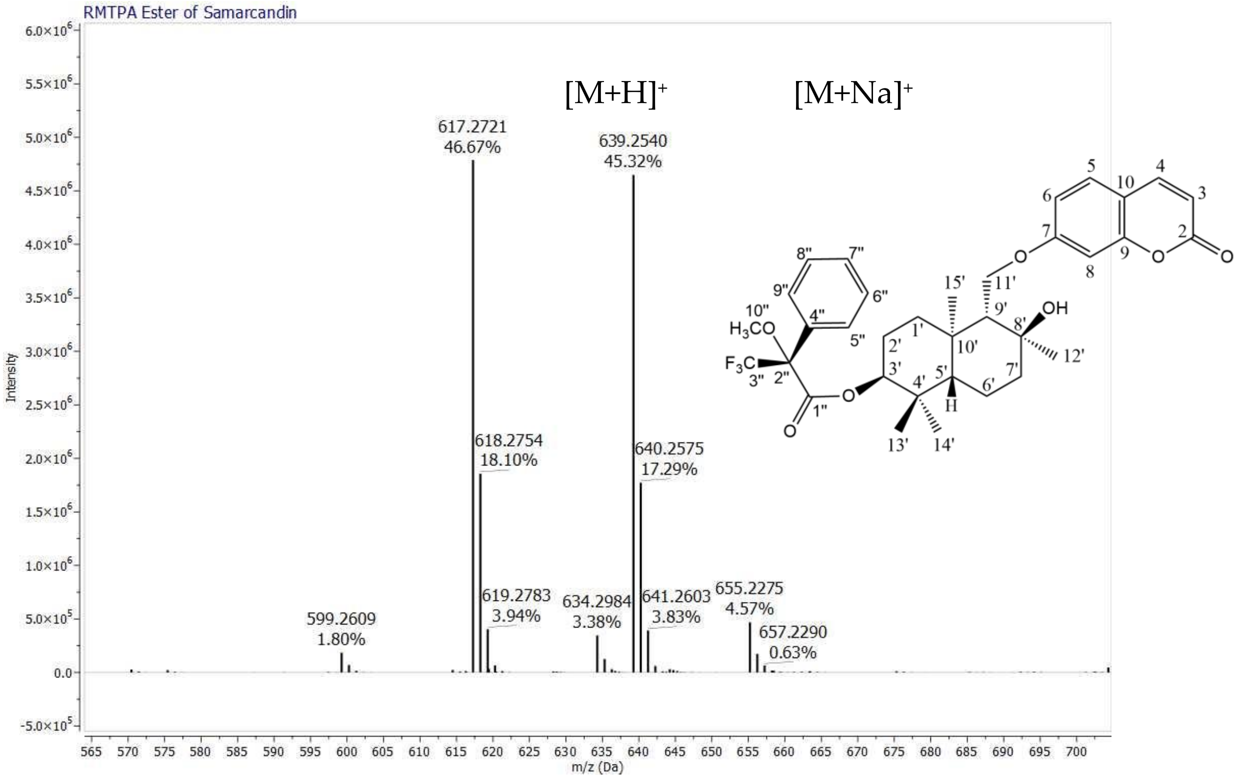

Figure S106. (+)-HRESIMS spectrum of (*R*)-MTPA ester of samarcandin (**24**)

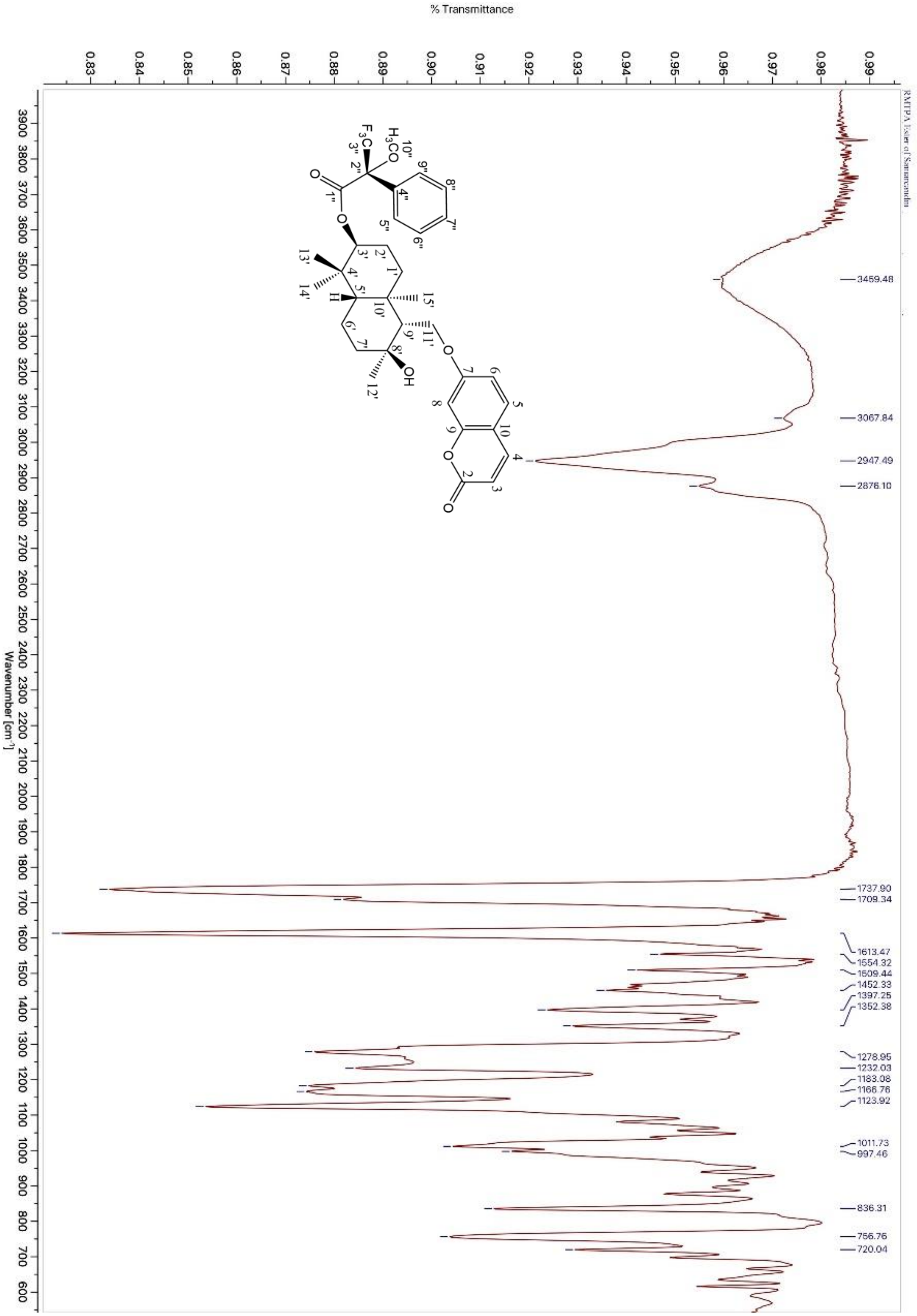

Figure S107. IR spectrum of (*R*)-MTPA ester of samarcandin (**24**)

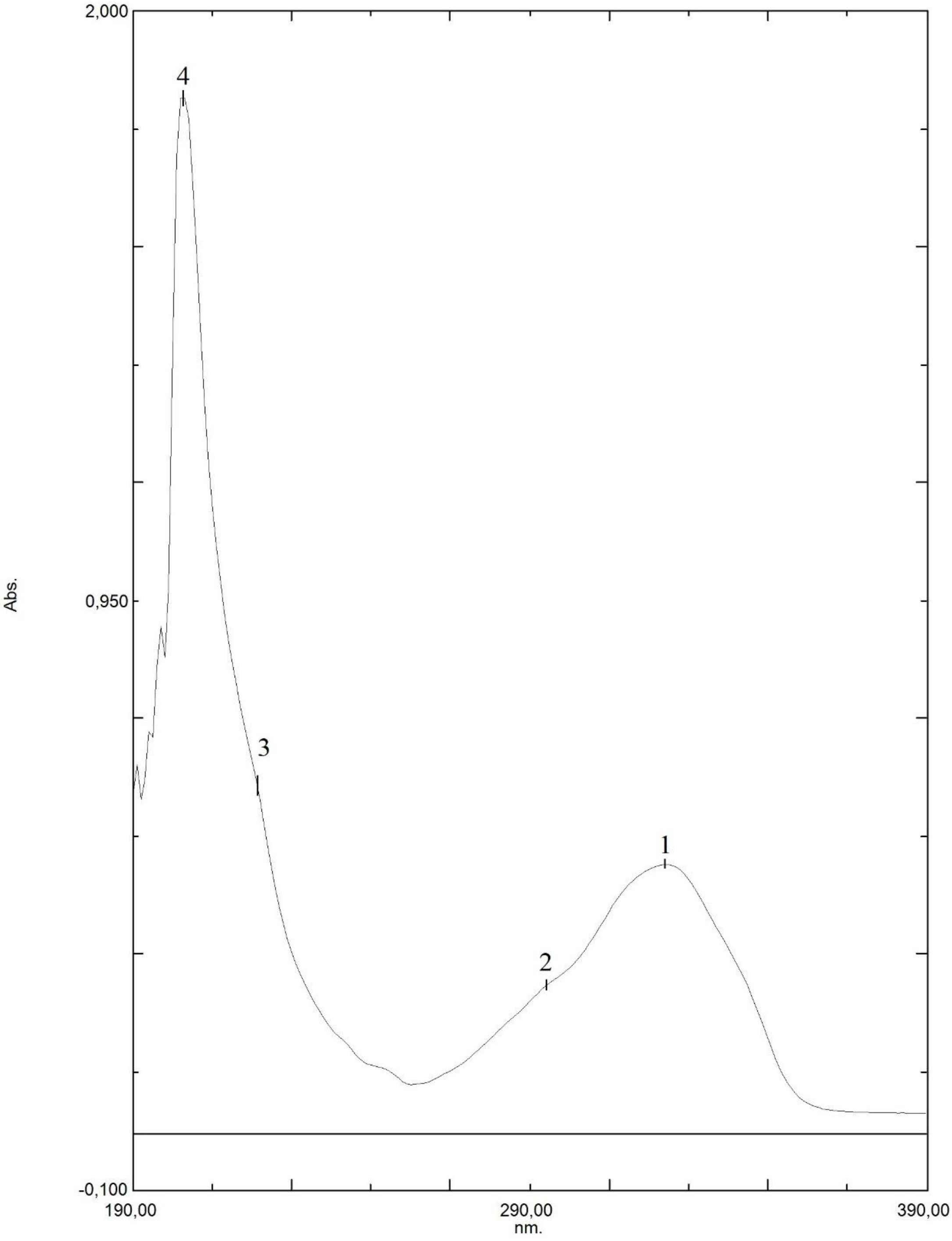

	Wavelength	Absorbance
1	324,00	0,478
2	294,00	0,262
3	221,00	0,630
4	202,00	1,845

Figure S108. UV spectrum of (*R*)-MTPA ester of samarcandin (**24**)

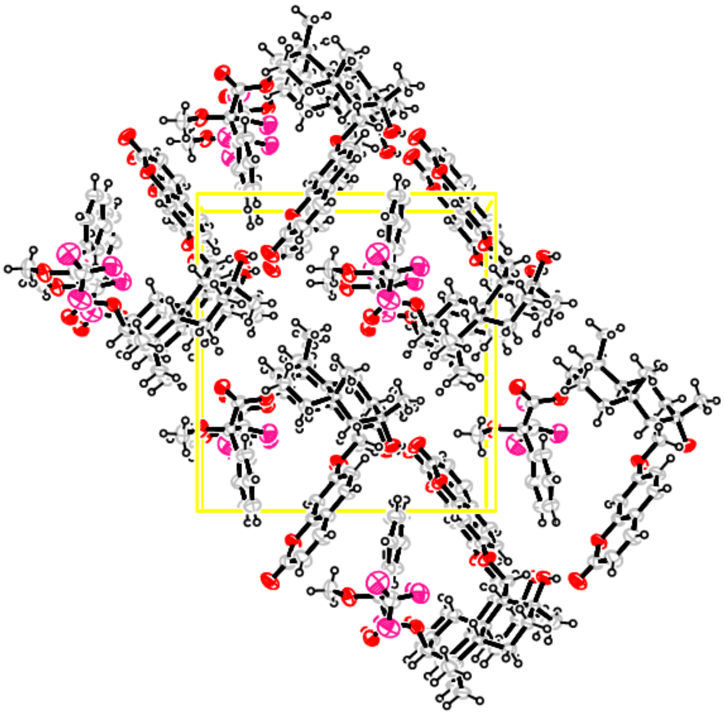

A

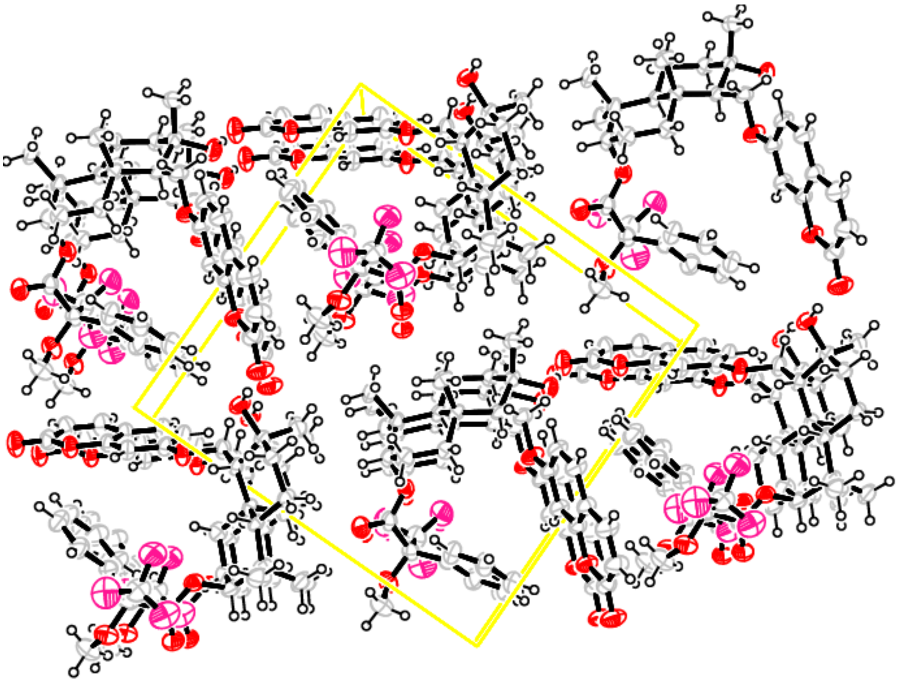

B

Figure S109. Packings of the (*R*)-MTPA ester of samarcandin (**24**) in unit cell

Table S2. Crystal data and details of the structure determination of (*R*)-MTPA ester of samarcandin (**24**)

Crystal formula	$C_{34} H_{39} F_3 O_7$
Formula weight	616.15
Crystal dimensions, [mm^3]	0.5x0.2x0.2
Temp, [K]	293(2)
Wavelength, [Å]	0.71073
Crystal system	Monoclinic
Space group; Z	P 2_1; 2
a, [Å]	7.31110(10)
b, [Å]	14.0819(4)
c, [Å]	14.9790(10)
β, [º]	97.977(6)
Volume [Å^3]	1527.23(11)
Dcalc [g.cm^{-3}]	1.341
F(000)	652.0
Abs. coefficient (μ, mm^{-1}]	0.104
Range of θ, [º]	3.10 to 27.47
Reflections collected	36935
Reflections used in refinement	6992
No. of refined parameters	413
Absorption correction	Multi-scan
Refinement method	Full matrix
R / R_w values	0.0379/ 0.1049
GOF	1.056
Final shift	0.000
$(\Delta\rho)_{min}$, $(\Delta\rho)_{max}$ (e Å^{-3})	0.252, - 0.203

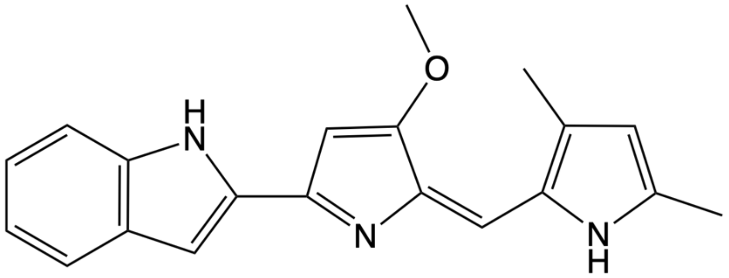

Obatoclax (Reference Compound for Bcl-XL & β-Catenin)

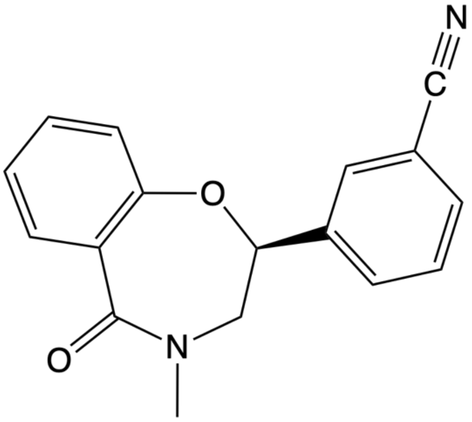

Compound 6 (Co-crystalized Ligand for β-Catenin)

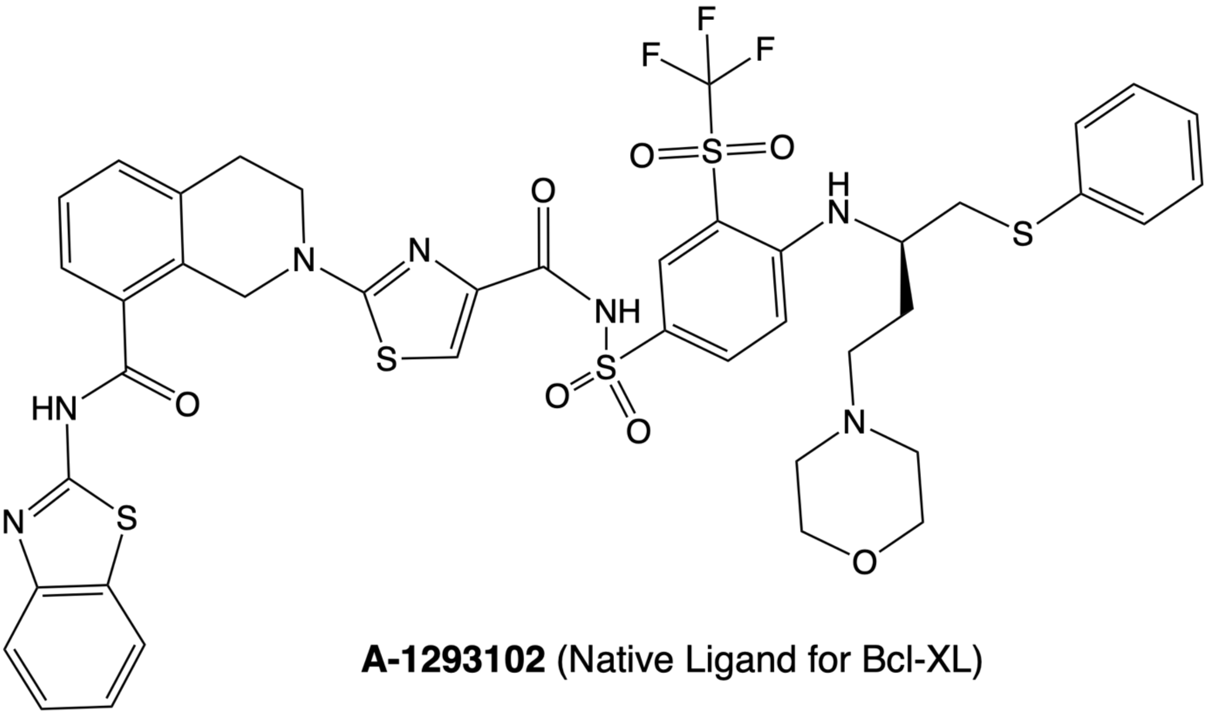

A-1293102 (Native Ligand for Bcl-XL)

Figure S110. Structures of Bcl-XL and β-Catenin ligands

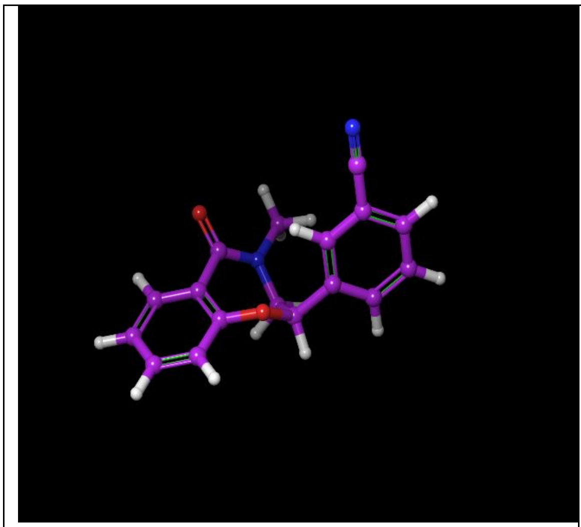	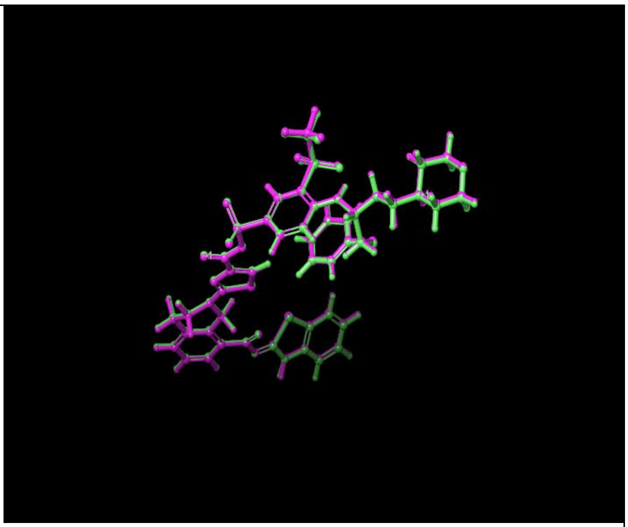
**RMSD 0.0429 Å**	**RMSD 0.5471 Å**

Figure S111. Validation of the docking models. Superposition of docked pose and experimental binding conformation (green) of Native Ligans in the binding pocket of 7AFW (left), 7LH7 (right)

References

1. Abd El-Razek, M.H.; Ohta, S.; Hirata, T. Terpenoid coumarins of the genus *Ferula*. *Heterocycles* **2003**, *60*(*3*), 689 - 716.
2. Ban'kovskii, A.I.; Ermatov, N.E.; Perel'son, M.E.; Bubeva-Ivanova, L.; Pavlova, N.S. Structure of the coumarins colladin and colladonin. II. *Chem. Nat. Compd.* **1970**, *6*, 170-176.
3. Aydogan, F.; Baykan, S.; Soliman, G.A.; Yusufoglu, H.; Bedir, E. Evaluation of the potential aphrodisiac activity of sesquiterpenoids from roots of *Ferula huber-morathii* Peşmen in male rats. *J. Ethnopharmacol.* **2020**, *257*, 112868.
